# Single Step Synthesis of Non-symmetric Azoarenes Using Buchwald–Hartwig Amination

**DOI:** 10.1021/acsomega.4c07485

**Published:** 2024-11-15

**Authors:** Martin Kocúrik, Pavlína Konopáčová, Lukáš Kolman, Pavel Kryl, Aleš Růžička, Jan Bartáček, Jiří Hanusek, Jiří Váňa

**Affiliations:** † Institute of Organic Chemistry and Technology, Faculty of Chemical Technology, University of Pardubice, Studentská 573, Pardubice 53210, The Czech Republic; ‡ Department of General and Inorganic Chemistry, Faculty of Chemical Technology, University of Pardubice, Studentská 573, Pardubice 53210, The Czech Republic

## Abstract

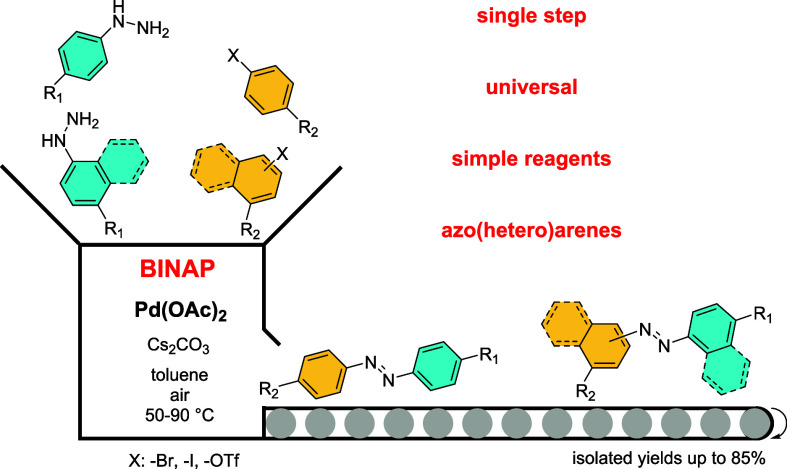

Aromatic azo compounds stand as a highly sought-after class of substances owing to their extensive array of applications across various fields. Despite their significance, their synthesis often presents challenges, requiring either multistep reactions or being restricted to specific substrate types. In this study, we are showing the universality and mechanistic aspects of a one-step approach for synthesis of nonsymmetrical azoarenes via the Buchwald–Hartwig amination reaction of (pseudo)haloaromatics with arylhydrazines, conducted in the presence of atmospheric oxygen. This reaction protocol yields products in up to 85% yield and is compatible with a wide class of substituents, making it highly adaptable. Notably, the inclusion of BINAP as a ligand plays a pivotal role in achieving favorable outcomes. This study not only offers a versatile solution to a long-standing synthetic challenge but also provides experimental and computational insights into the mechanisms driving the reaction.

## Introduction

Nonsymmetric aromatic azo compounds (diazenes) find extensive applications in both industry and academia. An example can be their traditional use as dyes, pigments, initiators, or more modern as chemosensors, molecular switchers, catalysts, and others.^1^ It can be said that they are currently one of the most interesting classes of compounds. Consequently, the synthesis of these compounds is assumed to be of paramount significance. It is therefore surprising that there is currently no universal direct method for their synthesis from simple reagents under mild conditions.^2^ Historically, the commonly used methods include azo-coupling and then Mills or Wallach reactions. Other, more modern approaches have been reviewed recently.^3^ The main disadvantage is the relatively narrow scope of all of these methods or their multistep character. For example, azocoupling is limited to electron-rich substrates, and the Mills reaction requires often problematic preparation of toxic and unstable nitroso compounds. Oxidative dehydrogenation of anilines^4^ or reductive coupling of nitroarenes^5^ suffers in controlling homo- and heterodimerized azo products. Recent Oestreich’s^6^ C(sp^2^)–N(sp^2^) cross-coupling or Li’s^7^ Cu-catalyzed C–N coupling requires the preparation of silylated aryl diazenes or phthalic hydrazides, respectively. For these reasons, our group focused on the synthesis of azo compounds using cross-coupling reactions of easily available materials.

The promising solution involves the in situ oxidation of diaryl hydrazines synthesized through the Buchwald–Hartwig amination reaction (BHA) of phenylhydrazines with “(pseudo)halogen” aromatics.

**Scheme 1:**
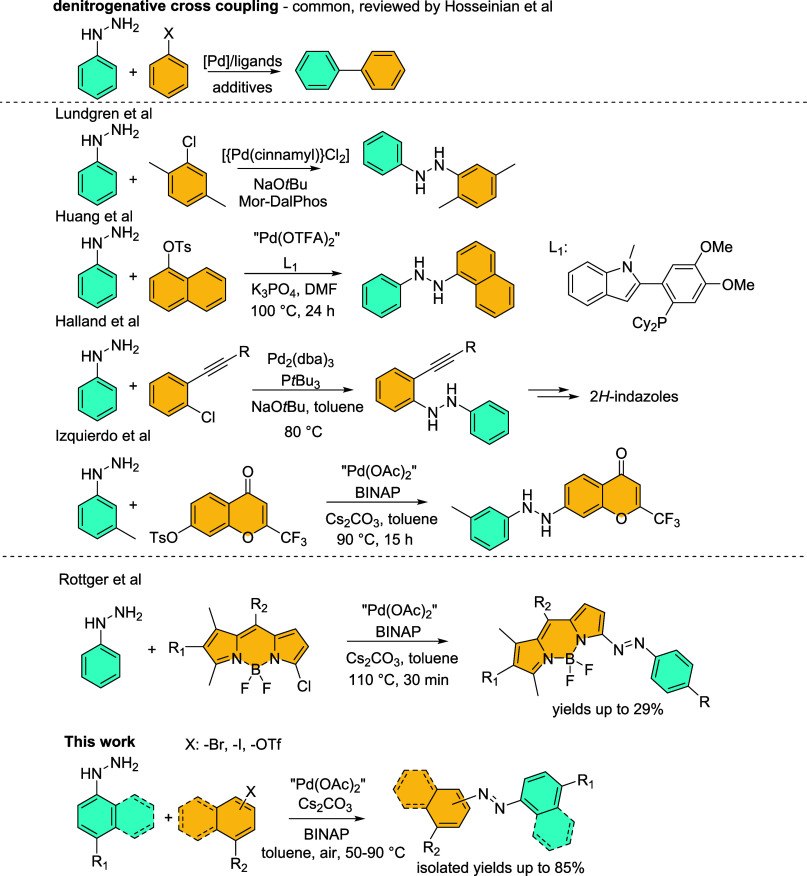
Summary of the Cross-Coupling Reactions Leading to 1,2-Diarylhydrazine Products

Nonetheless, this approach encounters two principal challenges. First, arylhydrazines typically undergo denitrogenation in combination with palladium and phosphine ligands and are thus an effective source of aryls for cross-coupling reactions (Scheme 1 top).^8^

Second, arylhydrazines exhibit reactivity at both nitrogen atoms, leading to the formation of both desired 1,2-diarylhydrazines and undesired 1,1-diarylhydrazines.^9^ Several strategies have been proposed to address this issue. The first approach involves the N1-protection of phenylhydrazines, albeit this elongates the overall reaction sequence.^10^ The second solution seeks to identify a suitable catalytic system that facilitates selective amination at the terminal N2 atom (Scheme 1). Notably, Lundgren et al. demonstrated that the reaction between 1-chloro-2,5-dimethylbenzene and phenylhydrazine, catalyzed by [{Pd(cinnamyl)Cl}_2_] with the Mor-DalPhos ligand, yields the corresponding 1,2-disubstituted hydrazine.^11^ Furthermore, Huang et al., who used a newly developed phosphine ligand having an indolylphenyl moiety, established that the reaction involving the sterically demanding 1-naphthyl tosylate and phenylhydrazine produces a 1,2-disubstituted hydrazine, in contrast to the phenyltosylates themselves.^9^ Next, Halland et al. observed the formation of *N*,*N*′-disubstituted hydrazines in a domino reaction leading to 2*H*-indazoles.^12^ Interestingly, in all these cases the (pseudo)halogen compound is *ortho*-substituted.^13^ Finally, Izquierdo et al. employed a combination of Pd(OAc)_2_ with BINAP for the amination of 4-chromenone-7-triflate,^14^ which is the only substrate having free *ortho* positions at the benzene nucleus.

One of the many routes allowing the oxidation of prepared 1,2-diarylhydrazines to azo compounds is aerobic oxidation using palladium acetate and a suitable base.^4,15^ Such a principle could be used for the *in situ* oxidation of the formed diarylhydrazines to azo compounds.

Thus, this work aims to develop an efficient catalytic system that enables the direct formation of azo compounds during the BHA reaction between arylhydrazines and “(pseudo)halogen” aromatics. The key to success lies in ensuring that the BHA reaction proceeds more rapidly than the undesired denitrogenation process and is selective toward the N2 atom of arylhydrazine. During the finalization of the manuscript, Röttger et al.^16^ published a protocol using almost identical conditions for the synthesis of phenylazo-BODIPYs from chloroderivatives (Scheme 1) in yields reaching 29%. Our work thus shows the possible breadth of application of this approach with yields reaching up to 85% and also brings important findings regarding the mechanistic rationale.

## Results and Discussion

### Preliminary Experiments

Inspired by the work of Izquierdo et al.,^14^ we first tried to adapt their protocol for the synthesis of 4-substituted phenanthrenes and their analogues.^17^ Thus, we run the reaction of triflates with arylhydrazines catalyzed by a palladium(II)acetate/BINAP system with cesium carbonate in toluene under an inert atmosphere (Scheme S1). We observed that during the reaction mixture workup, the initially formed diarylhydrazines undergo spontaneous oxidation to desired azocompounds in low to moderate yields.

### Optimization Experiments

Encouraged by these preliminary results, we tried to adapt the protocol for the synthesis of simpler 1-naphtylhydrazines from 1-bromonaphthalene (**1a**) and 3,5-dimethylhydrazine hydrochloride (**2a·HCl**) (Entry 1, Table 1). However, after the initial degassing of the reaction mixture, we maintained the reaction under air instead of inert atmosphere. To our delight, the GC–MS analysis showed the formation of the desired azocompound (**3a**) in a promising amount of 26%. Next to the desired product and starting 1-bromonaphthalene (35%), we observed product of denitrogenative cross-coupling, i.e., 1-(3,5-dimethylphenyl)naphthalene (**4a**) 19%, 1,1′-binaphthalene (**5a**) 7% (product of 1-bromonaphthalene homocoupling), and 3,3′,5,5′-tetramethyl-1,1′-biphenyl (**6a**) 13% (product of denitrogenative homocoupling).

**Table 1:**
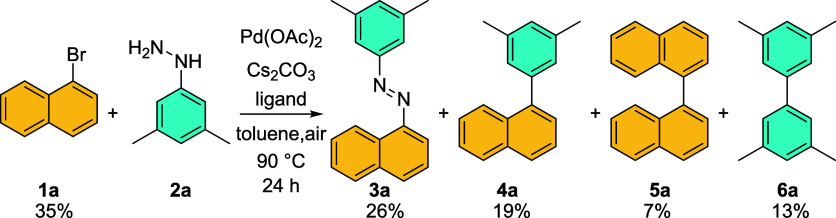
GC–MS Relative Abundances of Typical Products Formed during Screening Reactions

					GC–MS ratio				
entry	Pd(OAc)_2_ (mol %)	PH (equiv)	catalyst	solvent	**1a**	**6a**	**4a**	**5a**	**3a**
1	2.5	1.5 *HCl	BINAP	toluene	35	13	19	7	26
2	5	1.5 *HCl	BINAP	toluene		4	26	12	58
3	5 precomplexed	1.5 *HCl	BINAP	toluene					78% isol
4	5 precomplexed	1.5	BINAP	toluene		12			88 (85% isol)
5	5 precomplexed	1.5	PPh_3_	toluene		34	40	25	n.d.
6	5 precomplexed	1.5	Xanthphos	toluene		only			n.d.
7	5 precomplexed	1.5	dppf	toluene		13	58	30	n.d.
8	5 precomplexed	1.5	DPEphos	toluene		28	24	48	n.d.
9	5 precomplexed	1.5	dppp	toluene	28	33	25	5	8
10	5 precomplexed	1.5	(R)-MeOBIPHEP	toluene		13	24	11	51 (46% isol)
11	5 precomplexed	1.5	dppb	toluene	10	34	41	15	n.d.
12	5 precomplexed	1.5	dppm	toluene	12	33	40	16	n.d.
13	5 precomplexed	1.5	PPh_3_	NMP	93			7	n.d.
14	5 precomplexed	1.5	BINAP	NMP	98			2	n.d.

**Scheme 2:**
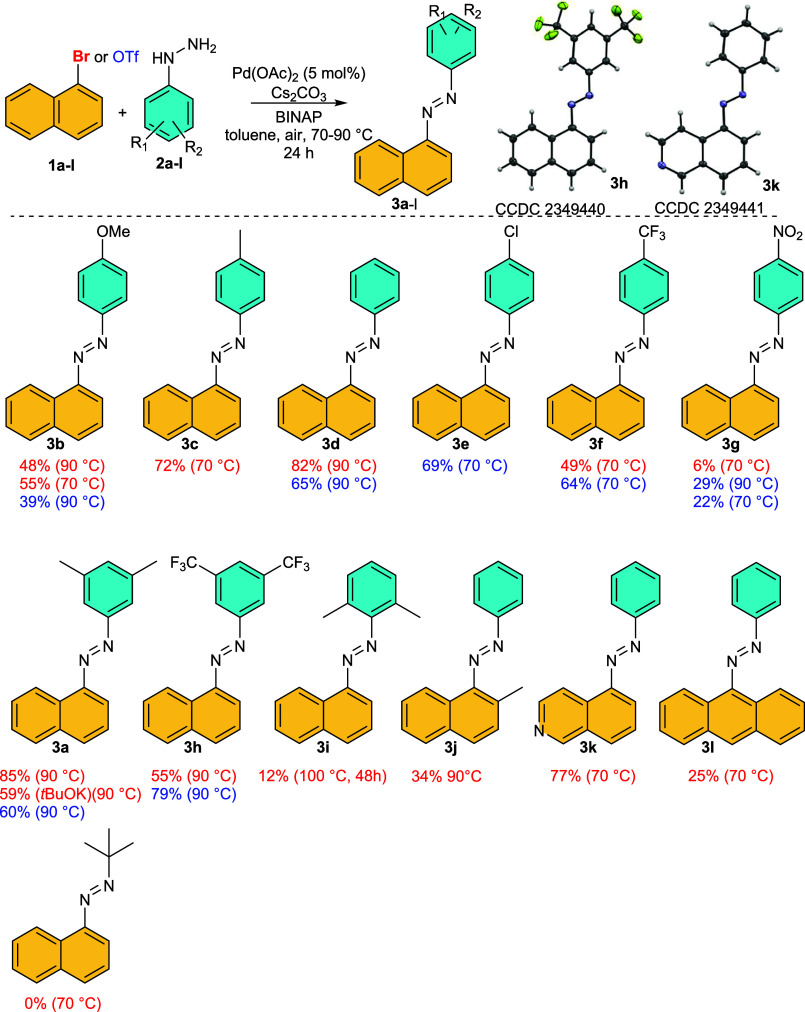
Substrate Scope: “*ortho*-” Substituted Substrates and Determined Molecular Structures of Compounds **3h** and **3k**

Encouraged by this success, we optimized the reaction conditions (Table 1). Next to the varying molar ratios of palladium (Entry 2), we investigated the role of the order of additions of individual components.^18^ The experiments showed that the best yields were achieved when palladium and BINAP were mixed first to create the precomplex under an inert atmosphere (Entry 3). Next, we used free phenylhydrazine **2a** instead of its hydrochloride **2a·HCl** (Entry 4). This improved the isolated yield of **3a** to 85%, and the reaction mixture contained only a negligible amount of undesired product of denitrogenative homocoupling **6a**. Next, we changed the base to *t*-BuOK, which gives only a slightly lower isolated yield (59%) (Scheme 2). Finally, we explored the roles of different ligands. However, most of the common and commercially available P-ligands (PPh_3_, XPhos, DPPF, DPEphos, or Xanthphos) failed (Entries 5–8). Therefore, we further tested structurally close ligands dppm, dppp, dppb, and MeOBIPHEP (Entries 9–12). In the case of dppp and MeOBIPHEP, the product was obtained but in lower yields. Furthermore, to verify the effect of the solvent, we examined the reaction with BINAP or PPh_3_ in NMP (Entries 13, 14), which were used in denitrogenative cross-couplings.^19^ In this case, we did not observe the formation of product **3a** and obtained only the expected denitrogenative cross-coupling product **4a** together with halogen derivate homocoupling **5a**. Another question is the amount of arylhydrazine. We found that a molar excess of it is needed, but the difference between 1.5 and 2 equiv is not significant. However, for substrates giving lower yields due to increased amounts of undesired denitrogenative cross-coupling products **6a**–**z**, a larger excess of hydrazine may be advantageous. Finally, we tried to substitute the bromine leaving group with trifluoromethanesulfonate (-OTf), but this modification did not fundamentally affect the yield.

### Substrate Scope

In the next part of this work, we examined the effects of the structure of arylhydrazines on the reactivity of both 1-bromo and 1-OTf naphthalenes (Scheme 2). Arylhydrazines bearing substituents with weak or moderate positive or negative electronic effects gave nice isolated yields in the range of (60–85%). On the other hand, employment of strong electron-donating or electron-withdrawing substituents in *para* positions of arylhydrazines causes a substantial reduction of isolated yields. Employment of doubly *ortho*-blocked 9-bromoanthracene **1l** and 2-methylnaphthalene **1j** gives 25% or 34% yields of **3l** and **3j**, respectively. The reduction of yield to 12% and the necessity of reaction time prolongation due to the steric hindrance of the arylhydrazine moiety illustrate employments of 2,6-dimethylphenylhydrazine **3i**. Finally, the reaction with aliphatic *t*-butylhydrazine failed, and starting material together with naphthalene homocoupling product **5a** was observed.

**Scheme 3:**
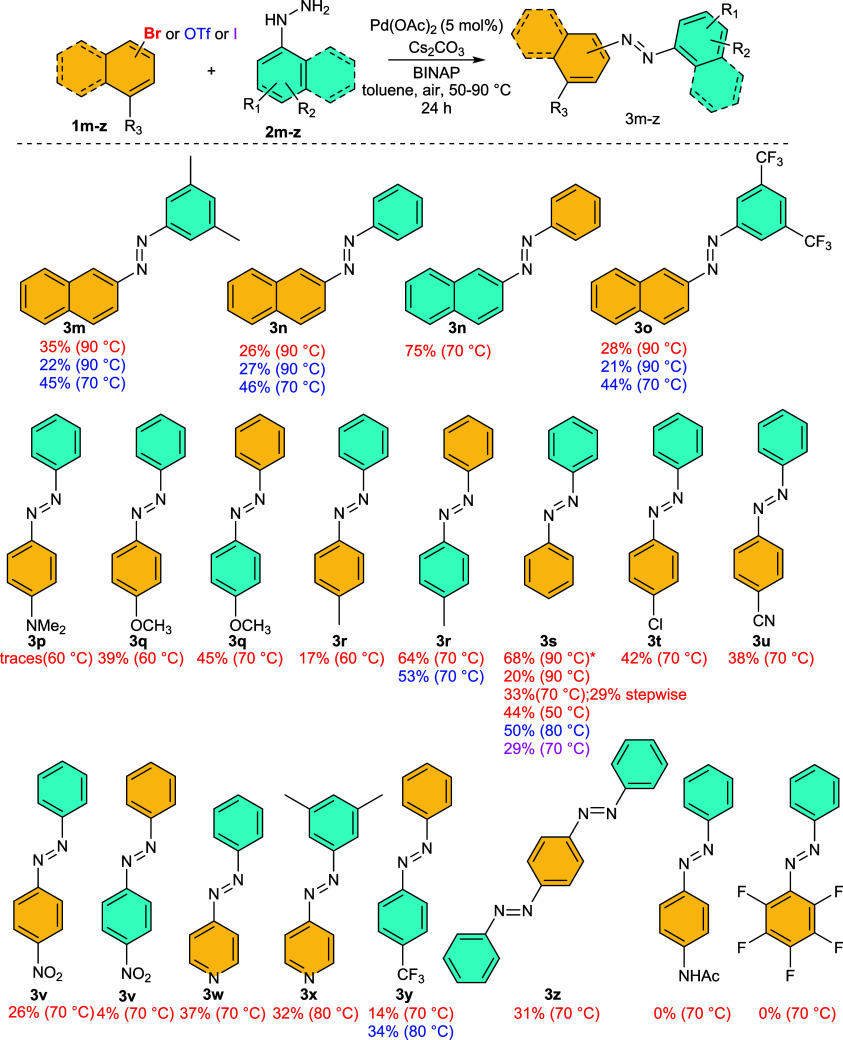
Substrate Scope: Substrates Bearing Unsubstituted *ortho* Positions. *Result of One Unrepeatable Experiment

Next, we focused on substrates bearing both *ortho*-positions free (Scheme 3). Hence, we explored the reactivity of 2-substituted (pseudo)halogenated naphthalenes and benzenes. For 2-substituted naphthalenes, the yields were notably lower (20–35%) compared to 1-naphthalenes, with a significant observation of the corresponding denitrogenative products **4**. To suppress the undesired denitrogenation pathway,^6^ we attempted to reduce the reaction temperature, resulting in improved yields of **3m**–**o** upon decreasing to 70 °C. Subsequent further temperature reduction primarily extended the reaction time while maintaining a similar ratio of products **3** vs **4**. Additionally, we endeavored to reverse the functionalities of the building blocks. Consequently, the reaction of 2-naphtylhydrazine with bromobenzene afforded product **3n** in a much-improved yield of 75% instead of 28%.

Finally, we examined the applicability of this protocol on simple (pseudo)halogen aromates, having the highest application potential (Scheme 3). The reaction temperature was again set in the range 60–70 °C. Initially, we varied the substituents on the synthetically/commercially more accessible (pseudo)halogen component in the reaction with phenylhydrazine. As depicted in Scheme 3, the isolated yields ranged approximately from 20 to 50%. The reduction in yields was mainly attributed to the formation of denitrogenative and homocoupling products.

No discernible trend in the electronic effects of substituents on the yield was observed; however, it was evident that strong electron-donating or electron-withdrawing substituents contributed to their decrease. Preferred activation of the C–Br bond over the C–Cl bond is demonstrated by the exclusive formation of product **3t** in the reaction of 4-bromochlorobenzene. Subsequently, we varied substituents on the phenylhydrazine moiety in the reaction with bromobenzene. The results indicated that this interchange of functionalities of reaction partners was advantageous in the case of electron-rich arylhydrazines, with, for instance, 4-methylphenylhydrazine, leading to a nice 64% isolated yield of **3r** (instead of 17%). Furthermore, other experiments showed that the protocol is compatible with heteroaromates **3k**, **3w**, and **3x**, or it can be used for double coupling, giving product **3z**. On the other hand, we found substrates such as 4-bromoacetanilide or pentafluorobromobenzene, which do not provide the desired products **3**.

### Reaction Mechanism

**Scheme 4:**
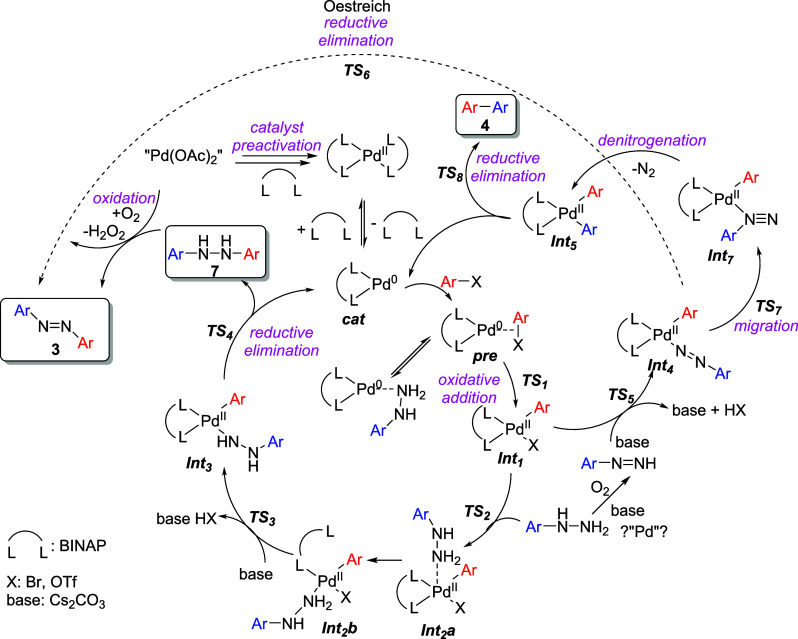
Plausible Reaction Mechanism

Based on literature precedents, a plausible reaction mechanism can be proposed (Scheme 4).^20^ Following the reduction of Pd(II) to Pd(0), preactivation of the catalyst to Pd(BINAP)^21^ and formation of the precomplex **pre**, oxidative addition into the aryl-(pseudo)halogen bond occurs, yielding intermediate **Int**_**1**_. The stability and reactivity of **Int**_**1**_ are one of the key factors that dictate the overall reaction efficiency. In the productive pathway, **Int**_**1**_ binds with arylhydrazine to (**Int**_**2a**_),^22^ followed by decoordination of one phosphorus atom from BINAP to (**Int**_**2b**_) (Figure S3 and ref (22)). Subsequently, base-assisted loss of HX takes place, resulting in the formation of **Int**_**3**_. Reductive elimination from **Int**_**3**_ yields the 1,2-diarylhydrazine product **7**. In the presence of oxygen and the palladium catalyst,^15^ 1,2-diarylhydrazine **7** undergoes oxidation to produce the azo product **3**.

In a parallel pathway, **Int**_**1**_ reacts with phenyldiazene to form intermediate **Int**_**4**_. Phenyldiazene is generated in the reaction mixture from arylhydrazine through the action of oxygen, base, and possibly Pd.^19,24^**Int**_**4**_ can potentially undergo two reactions. First, migration (**TS**_**7**_) and denitrogenation to (**Int**_**5**_) followed by reductive elimination (**TS**_**8**_) leads to the undesired biaryl compound **4**. Alternatively, following the pathway proposed by Oestreich,^6^**Int**_**4**_ can directly undergo reductive elimination (**TS**_**6**_) to yield the desired azo product **3**.

To validate this mechanism, we did several experiments and supported them with DFT calculations. Initially, to substantiate the existence of the diarylhydrazine intermediate, we performed a reaction between 1-bromonaphthalene and phenylhydrazine under an inert atmosphere. The GC–MS analysis of the reaction mixture after a 24 h duration reveals the coexistence of product **3d** and signals corresponding to the mass of diarylhydrazine **7** (Figure S1). After opening to atmosphere, this mixture exhibits stability at room temperature for 24 h. However, there is a reduction in the signals associated with arylhydrazines **7** and a concomitant increase in the signal corresponding to the product **3** upon its additional heating to 90 °C on the air. Thus, the oxidation of diarylhydrazine to azo product is facilitated or even conditioned by heating.

Thus, this experiment, together with the application of the two-step protocol described in the Preliminary Experiments session, shows that the diarylhydrazine intermediate is formed by the reaction, but it cannot be ruled out that the direct route proposed by Oestreich also in part applies. In any case, the mechanism suggested by Rötter et al.^16^ seems unlikely.

Furthermore, the undesired denitrogenation pathway requires the presence of oxygen. To suppress it, we ran the reaction under inert atmosphere for 24 h to generate the hydrazine intermediate, then opened the flask and ran the reaction for a further 24 h. However, this stepwise arrangement did not improve the isolated yield of **3s** (Scheme 3).

The important question connected with this oxidation is the ratio of formed (*E*)-/(*Z*)-isomers. The NMR analysis of crude reaction mixtures (Figure S2) shows almost exclusive formation of more stable (*E*)-isomers (typically more than 98%). The (*E*)-configuration of dominant products was confirmed by single-crystal X-ray diffraction of compounds **3h** and **k** (Scheme 2, and Supporting Information). This observation is in accordance with the literature.^15^

**Figure 1:**
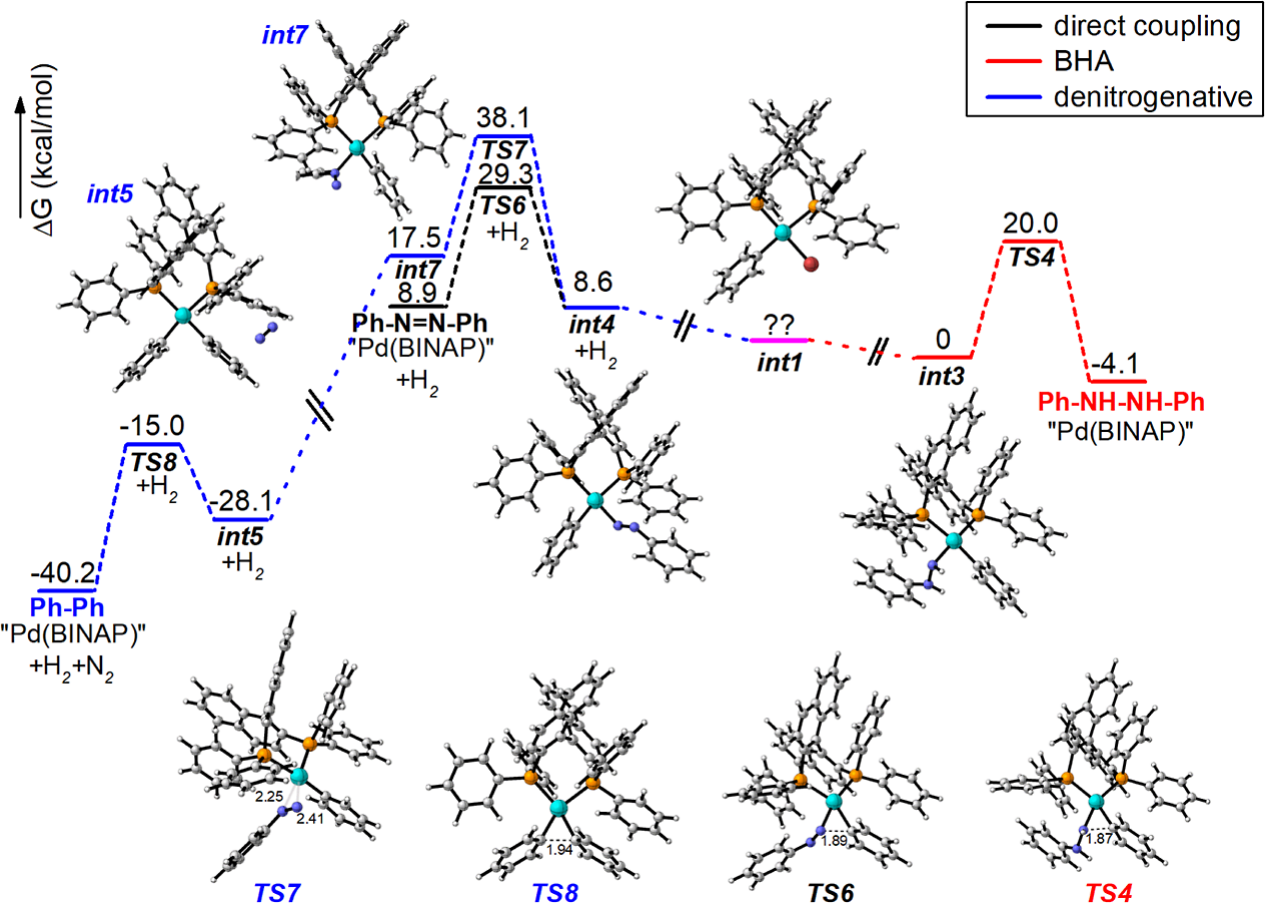
Comparison of reaction profiles (relative Gibbs energies in toluene (SMD) at 298 K in kcal mol^–1^ calculated at B3LYP/6-31+G*//6-311+G** for all atoms and SDD for the palladium level of theory with gd3bj empirical dispersion) of the reductive elimination steps: BHA to hydrazobenzene (red), Ostreichs direct coupling (black), and denitrogenative cross coupling (blue). The important bond lengths in TS are in Å.

Finally, we attempted to calculate the overall reaction mechanisms by DFT calculations. Unfortunately, we were not able to describe precisely the denitrogenation process because it most probably proceeds via dipalladium species^19,25^ that structure is very speculative. However, we were able to compare the relative activation barriers for the key reductive elimination steps. Computed results (Figure 1) show that both reductive eliminations from **Int**_**3**_ and **Int**_**4**_ giving hydrazobenzene (via **TS**_**4**_) and azobenzene (via **TS**_**6**_) have almost identical barriers (∼20 kcal/mol). On the other hand, in agreement with the work of Ananikov et al.,^26^ the barrier from **Int**_**5**_ leading to denitrogenative cross-coupling via **TS**_**8**_ is lover (∼13 kcal/mol). The calculated monopalladium barrier for denitrogenation, which precedes this elimination, is higher (30 kcal/mol). However, it can be assumed that involvement of the second palladium will reduce this barrier. Thus, it is clear that all reaction pathways have close barriers, and the formation of individual types of products is determined by small differences in the rate constants of individual steps caused by stereoelectronic effects together with the different oxidation rate of phenylhydrazines to phenyldiazenes.

A fundamental question remains unanswered as to why the BINAP ligand is crucial for the reaction when other (di)phosphine ligands fail and lead to the formation of denitrogenative cross-coupling or homocoupling products. Since BINAP does not deviate in any way from the range of (di)phosphine ligands for the BHA reaction,^23^ it is possible that the key parameter will be its bite angle.^27^ This confirms the formation of the product when using the structurally and angularly closest ligands, MEOBIPHEP and dppp.

## Conclusions

Through the utilization of the Buchwald–Hartwig amination reaction of pseudo(halo)aromatics with arylhydrazines in the presence of oxygen, we demonstrated the universality of a one-step approach to aromatic azo compounds from simple compounds under mild conditions.

First, this reaction protocol provides high yields of aromatic compounds with efficiencies of up to 85%, making it a viable and practical method for their synthesis. Furthermore, the protocol is not fundamentally limited by the effect of the substitution, demonstrating its versatility across a wide range of substrates, including heteroaromates or double coupling; however, the best yields are achieved for condensed aromates.

Importantly, the inclusion of BINAP as a ligand proved to be crucial for achieving favorable outcomes, highlighting the significance of ligand design in catalytic systems. Moreover, the proposed reaction mechanism, supported by both experimental findings and DFT calculations, offers insights into the underlying processes involved.

The development of such a method addresses long-standing challenges in the field and opens up new avenues for the exploration of aromatic azo compounds in various applications across the industry and academia. Further investigations into reaction optimization and mechanistic studies will undoubtedly enhance our understanding and utilization of this synthetic approach.

## Experimental Section

### Materials and Methods

All the chemicals and solvents were purchased from Acros Organics, Sigma-Aldrich, or Fluorochem and used as received. Palladium(II) acetate (recrystallized) was purchased from Sigma-Aldrich. High-resolution mass spectra were recorded on a MALDI LTQ Orbitrap XL equipped with a nitrogen UV laser (337 nm, 60 Hz, 8–20 μJ) in the positive ion mode or at micrOTOF-QIII, Bruker Daltonics Inc., in the APCI^+^ mode calibrated on an ESI-L Low Concentration Tuning Mix, Agilent Technologies. The NMR spectra were recorded on a Bruker Avance III 400 MHz or on a Bruker Ascend 500 MHz instrument. Chemical shifts δ are referenced to TMS (δ = 0 ppm), trifluorotoluene (−63.72 ppm), or solvent residual peaks δ(CDCl_3_) = 7.26 ppm (^1^H)/77.16 ppm (^13^C); δ(DMSO-*d*_6_) = 2.50 ppm (^1^H)/ 39.6 ppm (^13^C). FT-IR spectra were measured on a Nicolet (Thermo Scientific) iS50 spectrophotometer using the ATR (diamond) technique. The compounds were purified by the Biotage Selekt flash chromatography system. The microanalysis was determined using FISIONS EA 1108 CHN. Details of the DFT calculations as well as single crystal X-ray diffraction data are given in the Supporting Information.

### General Procedure for the Synthesis of Azoarenes

Pd(OAc)_2_ (8 mg, 0.0357 mmol, 5 mol %) and BINAP (45 mg, 0.0713 mmol, 10 mol %) were added to the Schlenk flask, followed by the addition of dry toluene (4 mL). The mixture was degassed by N_2_ for 10 min and subsequently heated to 80 °C for 15 min. After cooling down to room temperature, were added aryl halogenide or aryltrifluormethanesulfonate (0.7133 mmol, 1 equiv), arylhydrazine (freshly prepared free base, 1.070 mmol, 1.5 equiv), Cs_2_CO_3_ (256 mg, 0.784 mmol, 1.1 equiv), and dry toluene (4 mL), and the mixture was degassed by N_2_ for 10 min. Subsequently, the mixture was stirred at a certain temperature for 24 h in a Schlenk flask open to air. After 24 h, the mixture was filtered through a plug of silica and eluted with EtOAc (3 × 10 mL), and solvent was subsequently removed under reduced pressure. Crude products were purified by flash chromatography using hexane/dichloromethane (4:1).

## Supporting Information Available

Experimental details; mechanistic findings; spectra of compounds; calculated coordinates; X-ray analysis details; and references (PDF)
X-ray crystallographic data of **3h** (CIF)
X-ray crystallographic data of **3k** (CIF)

## Supplementary Material

ao4c07485_si_001.pdf

ao4c07485_si_002.cif

ao4c07485_si_003.cif

## Dedication

Dedicated to prof. Dr. Miloš Sedlák on the occasion of his 65th birthday.
