# Supplementary material for: Single Step Synthesis of Non-symmetric Azoarenes Using Buchwald–Hartwig Amination
Source: ACS Omega. 2024 Nov 15;9(47):47105–13. doi: 10.1021/acsomega.4c07485 (PMC11603238; doi:10.1021/acsomega.4c07485)
Supplement: Supplementary file 1 — ao4c07485_si_001.pdf [file ao4c07485_si_001.pdf]

Supporting Information

Single Step Synthesis of Non-symmetric Azoarenes Using Buchwald-Hartwig Amination

Martin Kocúrik, † *Pavlína Konopáčová,* †,§ *Lukáš Kolman,* † *Pavel Kryl,* † *Aleš Růžička* ‡ *Jan Bartáček,* † *Jiří Hanusek,* † *and Jiří Váňa* †*

†Institute of Organic Chemistry and Technology, Faculty of Chemical Technology, University of Pardubice, Studentská 573, 53210 Pardubice, The Czech Republic

‡ Department of General and Inorganic Chemistry, Faculty of Chemical Technology, University of Pardubice, Studentská 573, 53210 Pardubice, The Czech Republic

§ Current address: Institute for Nanomaterials, Advanced Technologies and Innovation, Technical University of Liberec, Studentská 1402/2, 461 17, Liberec 1, Czech Republic

Table of Contents

DFT Calculations 2

Preliminary experiments 3

Mechanistic findings 20

General procedure for aryltrifluoromethanesulfonates 24

General procedure for azo compounds 26

Single crystal X-ray difraction data 38

Spectra 44

Coordinates 119

References 154

DFT Calculations

All calculations were performed using the B3LYP density functional theory method as implemented in Gaussian16[1] with the D3 dispersion term using the Becke-Johnson damping function.[2] The basis set for geometry optimization was a combination of the SDD pseudopotential model for palladium[3] and 6-31+G* for all other atoms. The solvent was included by the SMD model.[4] The transition states are characterized by one and only one imaginary frequency. The so optimized geometries were used for the single point calculations at D3BJ-B3LYP/ 6-311+G** (SDD for Pd) level of theory including SMD solvation model. The free energy corrections from the lower level of theory frequency computations are added to the higher level of theory single-point energies to account for the entropic effects. Graphics were processed using the software CYLview.[5]

Preliminary experiments

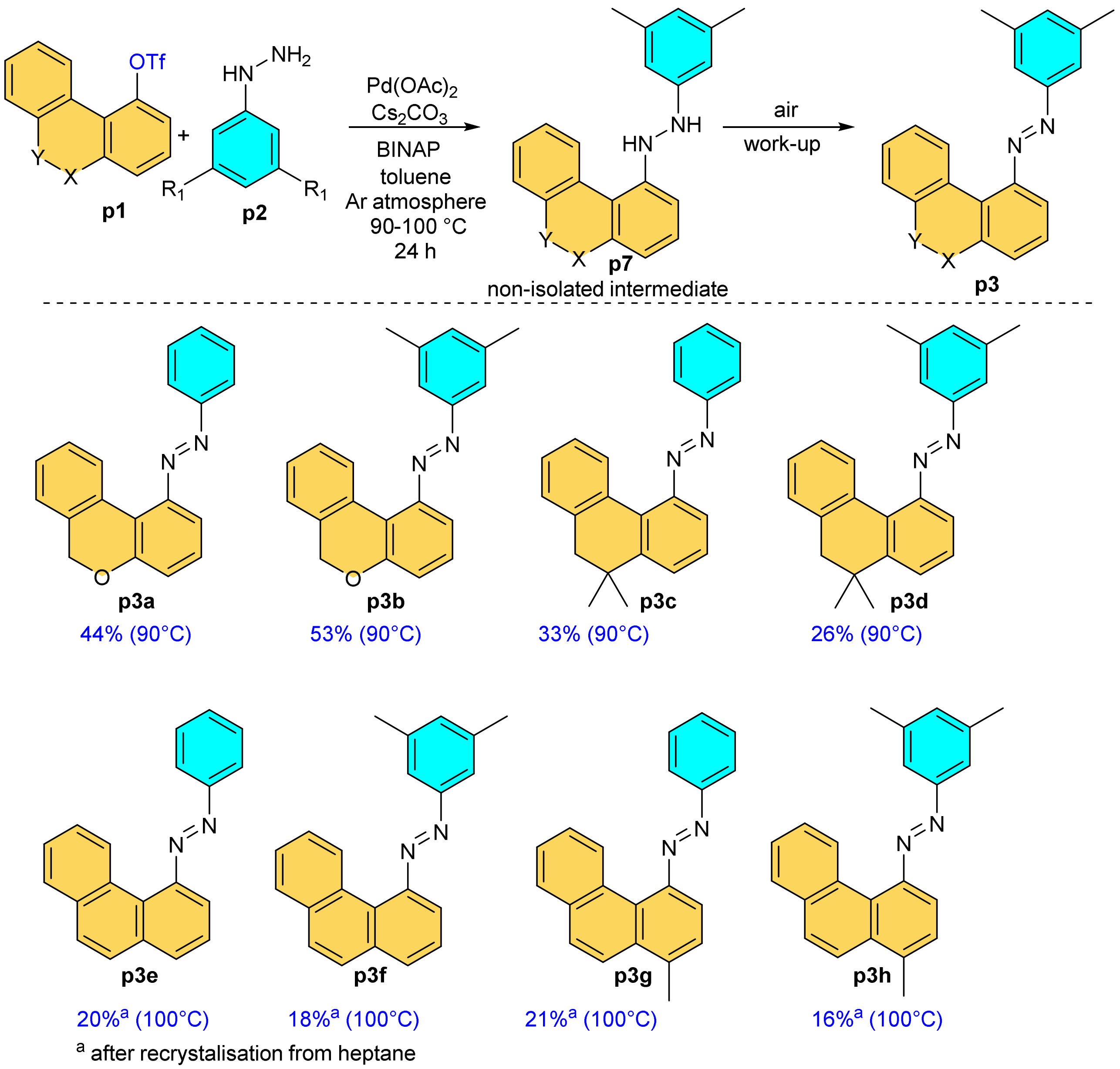

Scheme S1. Preliminary experiments using stepwise protocol inspired by Izquiredo et al.[6]

Sequence to 6H-benzo[c]chromen based azocompounds

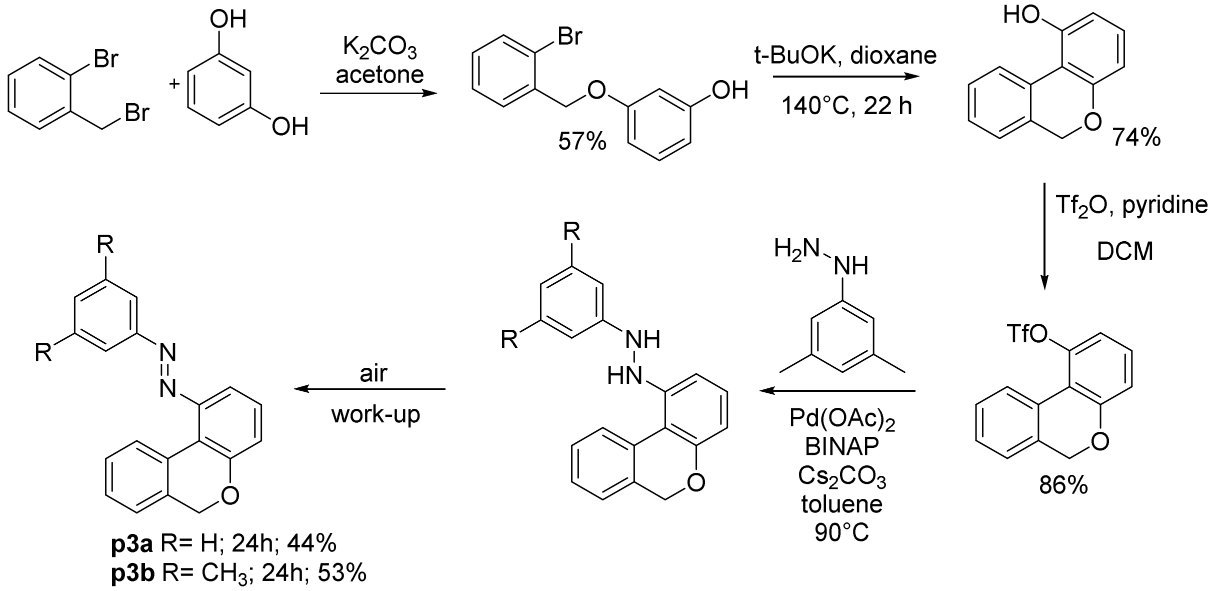

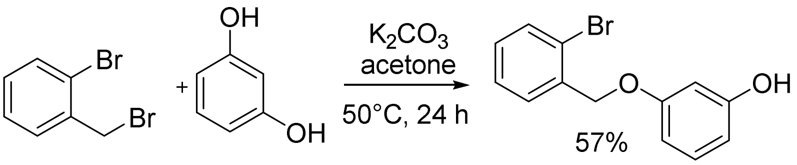

3-((2-Bromobenzyl)oxy)phenol[7]

In a 250 ml round-bottom flask, 4.95 g (45 mmol) of resorcinol was dissolved in 75 ml of acetone, followed by the addition of 2.1 g (15 mmol) of K_2CO_3. The mixture was stirred using a magnetic stirrer for 30 minutes at room temperature, after which 3.75 g (15 mmol) of 2-bromobenzyl bromide was added. The reaction mixture was heated to 50 °C and stirred for 24 hours. Subsequently, the reaction mixture was cooled and poured into 75 ml of a 2 M NaOH solution. Extraction was then performed using 3 × 50 ml of ethyl acetate, and the organic layers were combined, washed with 40 ml of brine, and dried over anhydrous Na_2SO_4. The solvent was evaporated, and the residue was subjected to flash chromatography on silica gel using a gradient elution of EtOAc/hexane (5/95 → 100/0) over 30 minutes. This yielded 2.4 g (57%) of desired compound as a brown oil.

^{1}H NMR (400 MHz, Chloroform-*d*) δ 7.56 (d, *J* = 8.6 Hz, 1H), 7.51 (d, *J* = 7.7 Hz, 1H), 7.35-7.26 (m, 2H), 7.21-7.09 (m, 3H), 6.57 (d, *J* = 8.3 Hz, 2H), 6.49 (s, 2H), 6.45 (d, *J* = 8.1 Hz, 1H), 5.18 (s, 2H)

^{13}C NMR (101 MHz, $CDCl_3$) δ 159.7, 156.6, 136.0, 132.6, 130.2, 129.2, 128.8, 127.5, 122.2, 108.3, 107.3, 102.5, 69.4.

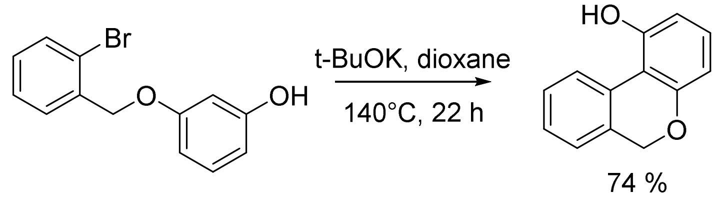

6H-Benzo[c]chromen-1-ol[8]

Into a pre-dried pressure vessel, 3-((2-bromobenzyl)oxy)phenol (1.7 g, 6.0 mmol), *t*-BuOK (1.7 g, 15 mmol), and dioxane (10 ml) were introduced. The vessel was purged with argon, sealed with a pressure cap, and the reaction mixture was stirred at room temperature for 5 minutes, followed by 2 hours in a silicone oil bath heated to 140 °C. After cooling to room temperature, the reaction mixture was poured into 2 M HCl (50 ml) and extracted with ethyl acetate (3 × 30 ml). The combined organic layers were washed with brine (40 ml) and dried over anhydrous Na_2SO_4. The solvent was evaporated and the residue was subjected to chromatographic separation on silica gel (dichloromethane/hexane 40:60), yielding 0.9 g (74%) of desired solid compound with a melting point of 167-168 °C.

^{1}H NMR (400 MHz, Chloroform-*d*) δ 8.04 (d, *J* = 7,9 Hz, 1H), 7,39 (dt, *J* = 27.3, 7.5 Hz, 2H), 7.28-7.17 (m, 2H), 7.04 (dd, *J* = 13.7, 8.3 Hz, 2H), 5.04 (s, 2H).

^{13}C NMR (101 MHz, $CDCl_3$) δ 159.7, 156.6, 136.1, 132.6, 130.2, 129.2, 128.8, 127.5, 122.2, 108.3, 107.3, 102.5, 69.4.

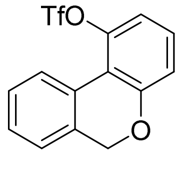

6H-Benzo[c]chromen-1-yl trifluoromethanesulfonate (p1a)

Was synthetized according to general protocol yielding 1.37 g (82 %) of yellowish oil

^{1}H NMR (400 MHz, Chloroform-*d*) δ 8.04 (d, *J* = 7.9 Hz, 1H), 7.39 (dt, *J* = 27.3, 7.5 Hz, 2H), 7.28-7.17 (m, 2H), 7.04 (dd, *J* = 13.7, 8.3 Hz, 2H), 5.04 (s, 2H).

^{13}C NMR (101 MHz, $CDCl_3$) δ 157.3, 146.5, 132.3, 129.0, 128.8, 128.7, 126.3, 126.2, 125.1, 123.3, 120.1, 117.9, 117.6, 117.0, 116.0, 113.8, 69.1.

Sequence to 9,10-dihydrophenanthrene based azocompounds

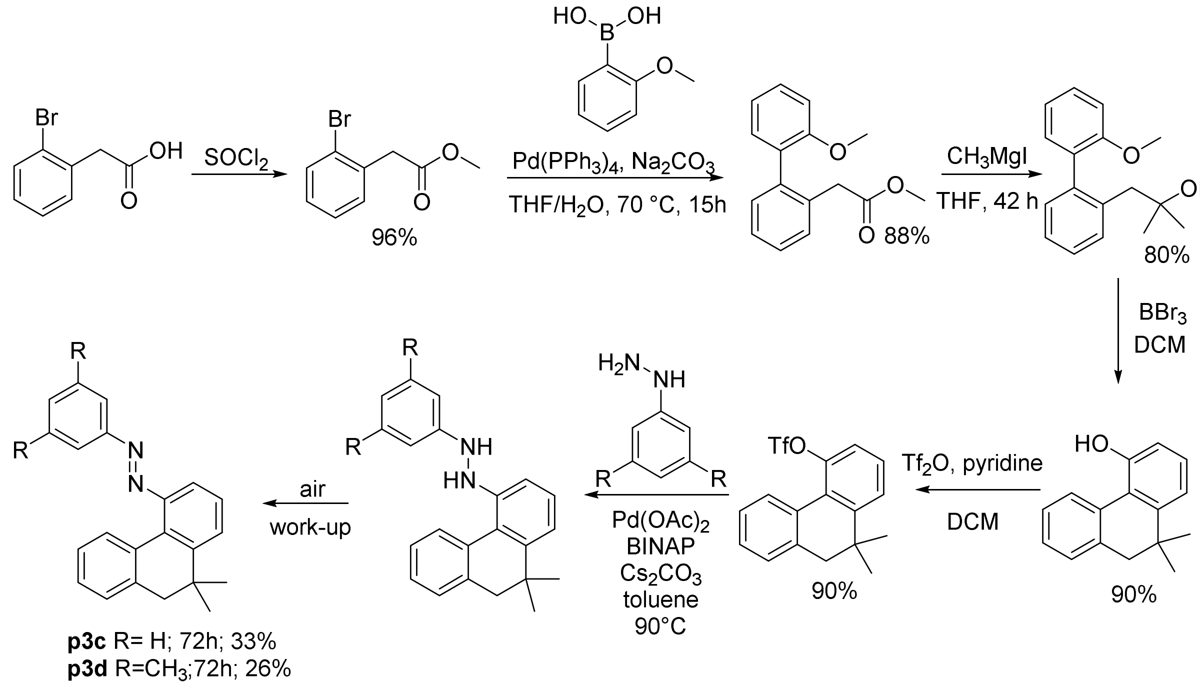

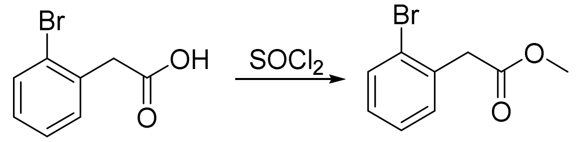

Methyl 2-(2-bromophenyl)acetate[9]

2-(2-bromophenyl)acetic acid (10.8 g, 50 mmol) was placed in a 250 ml flask and dissolved in methanol (75 ml). The mixture was cooled in an ice bath, and thionyl chloride (16.4 ml, 75 mmol) was added dropwise over a period of 10 minutes. The reaction mixture was stirred for 3 hours, after which methanol was distilled off, and 100 ml of water was added to the residue. The resulting emulsion was extracted 3 x 70 ml of ethyl acetate. The combined organic layers were washed with 50 ml of saturated aqueous $NaHCO_3$, dried over anhydrous Na_2SO_4, filtered, and evaporated. Thus, 11 g (96%) of pale yellow oil was obtained, which did not require further purification.

^{1}H NMR (500 MHz, Chloroform-*d*) δ 7.56 (d, *J* = 8.2 Hz, 1H), 7.31-7.23 (m, 2H), 7.17-7.10 (m, 1H), 3.79 (s, 2H), 3.71 (s, 3H).

^{13}C NMR (126 MHz, $CDCl_3$) δ 170.9, 134.1, 132.7, 131.4, 128.8, 127.5, 124.9, 52.1, 41.4.

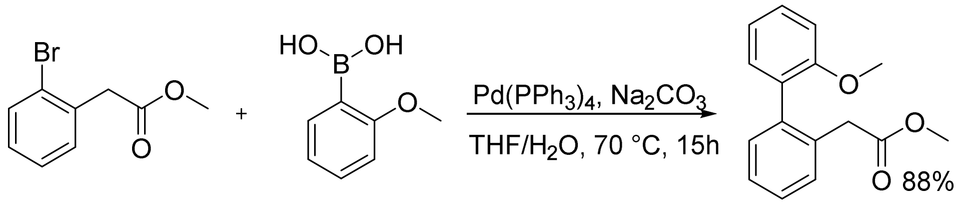

Methyl 2-(2'-methoxy-[1,1'-biphenyl]-2-yl)acetate[10]

Methyl 2-(2-bromophenyl)acetate (4 g, 17.3 mmol), 2-methoxyphenylboronic acid (3.2 g, 20.7 mmol), Na_2CO_3 (3.7 g, 35 mmol), and a mixture of HF/H_2O in a ratio of 3:1 (175 ml) were placed in a Schlenk flask. The reaction mixture was initially purged with argon for 30 minutes. Subsequently, $Pd(PPh_3)_4$ (1 g, 0.86 mmol) was added, and the mixture was further purged with argon for 5 minutes. The reaction mixture was heated to 70°C for 15 hours. After this period, the reaction mixture was cooled to room temperature, filtered through Celite, which was then washed with 100 ml of EtOAc. The filtrate was diluted with 150 ml of water and extracted 3 x 50 ml of EtOAc. The combined organic layers were washed with 100 ml of brine, dried over anhydrous Na_2SO_4, filtered, and evaporated. The product was purified by flash chromatography with a gradient elution of EtOAc/hexane 2:8 → 3:7 over 30 minutes. Thus, 3.9 g (88%) of desired compound was obtained as a yellow oil.

^{1}H NMR (500 MHz, Chloroform-*d*) δ 7.38-7.27 (m, *J* = 11.1, 6.4, 3.3 Hz, 4H), 7.23-7.18 (m, 1H), 7.17-7.11 (m, 1H), 6.99 (t, *J* = 7.2 Hz, 1H), 7.04-6.89 (m, 2H), 6.93 (d, *J* = 8.3 Hz, 1H), 3.70 (s, 3H), 3.55 (s, 3H), 3.48 (s, 2H).

^{13}C NMR (126 MHz, $CDCl_3$) δ 172.1, 156.4, 138.8, 133.0, 131.4, 130.4, 129.9, 129.8, 129.0, 127.5, 126.9, 120.5, 110.6, 55.2, 51.6, 38.8.

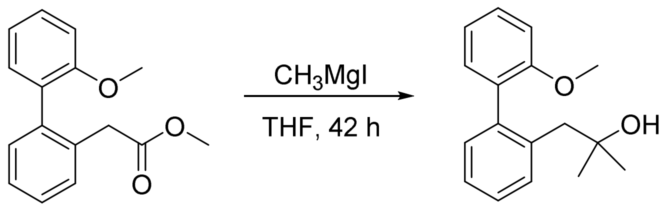

1-(2'-Methoxy-[1,1'-biphenyl]-2-yl)-2-methylpropan-2-ol[11]

Dry diethylether (50 ml) was added under argon flow to magnesium (6.5 g, 270 mmol). Carefully, methyl iodide (11.2 ml, 180 mmol) was added to it in small portions. The mixture was then refluxed for 1 hour. After cooling to room temperature, the resulting solution of methylmagnesium iodide was prepared for further use.

Methyl 2-(2'-methoxy-[1,1'-biphenyl]-2-yl)acetate (3.5 g, 13.6 mmol) was placed in a flask filled with argon and dissolved in dry THF (250 ml). After dissolution of the ester, 23 ml of the

prepared CH_3MgI solution was added to the reaction, and the mixture was further stirred for 16 hours at room temperature. Subsequently, 16 ml of CH_3MgI solution was added, and the reaction mixture was heated to reflux for 7 hours under a reflux condenser. After this period, another 16 ml of the prepared CH_3MgI solution was added to the reaction, and the mixture was refluxed for an additional 19 hours. The contents of the flask were then poured into 300 ml of saturated NH_4Cl solution and extracted 5 times with 100 ml portions of EtOAc. The combined organic layers were washed twice with 100 ml brine, dried over anhydrous Na_2SO_4, filtered, and evaporated. The resulting product was purified by flash chromatography with a gradient elution of hexane/EtOAc 5:1 → 2:1 over 30 minutes. Thus, 3.15 g (90%) of product was obtained as a yellow oil.

^{1}H NMR (500 MHz, Chloroform-*d*) δ 7.39-7.24 (m, 4H), 7.19 (d, *J* = 1.9 Hz, 1H), 7.14 (d, *J* = 1.8 Hz, 1H), 7.00 (t, *J* = 1.1 Hz, 1H), 6.96 (dd, *J* = 8.2, 1.1 Hz, 1H), 3.74 (s, 3H), 2.82 (d, *J* = 13.6 Hz, 1H), 2.66 (d, *J* = 13.6 Hz, 1H), 1.61-1.47 (m, 1H), 1.03 (s, 3H), 0.99 (s, 3H).
^{13}C NMR (126 MHz, $CDCl_3$) δ 156.2, 139.4, 136.4, 131.7, 131.1, 130.9, 128.7, 127.1, 126.2, 120.4, 110.8, 71.4, 55.4, 45.9, 29.5.

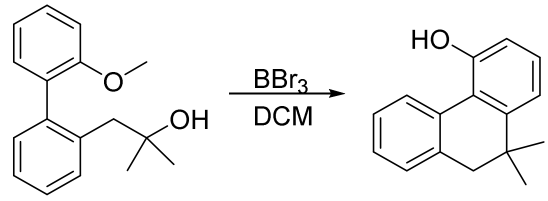

10,10-Dimethyl-9,10-dihydrophenanthren-4-ol[11]

1-(2'-Methoxy-[1,1'-biphenyl]-2-yl)-2-methylpropan-2-ol (3.85 g, 15 mmol) was dissolved under argon atmosphere in 200 ml of dichloromethane. To the solution, BBr_3 (3.1 ml, 33 mmol) was added dropwise, and the reaction mixture was stirred for 210 minutes. Subsequently, the mixture was poured into 300 ml of saturated $NaHCO_3$ solution and extracted 5 x 200 ml of CH_2Cl_2. The extract was then dried over anhydrous Na_2SO_4, filtered, and evaporated. The resulting product was purified by flash chromatography with a gradient elution of hexane/EtOAc 9:1 → 2:1 over 30 minutes. Thus, 1.9 g of compound was obtained as a brown oil with a yield of 56%.
^{1}H NMR (500 MHz, Chloroform-*d*) δ 8.14 (d, *J* = 7.8 Hz, 1H), 7.35-7.27 (m, 1H), 7.23 (dd, *J* = 10.9, 4.7 Hz, 2H), 7.15 (t, *J* = 7.9 Hz, 1H), 7.01 (d, *J* = 7.7 Hz, 1H), 6.79 (dd, *J* = 8.0, 0.7 Hz, 1H), 5.34 (s, 1H), 2.70 (s, 2H), 1.19 (s, 6H),

^{13}C NMR (126 MHz, $CDCl_3$) δ 152.7, 148.4, 137.2, 131.9, 128.8, 128.4, 127.1, 126.6, 126.3, 120.7, 116.7, 114.9, 44.4, 34.7, 27.5.

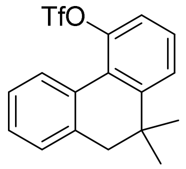

10,10-dimethyl-9,10-dihydrophenanthren-4-yl trifluoromethanesulfonate (p1c)

Was synthetized according to general protocol yielding 1.74 g (91 %) of yellowish oil.

^{1}H NMR (500 MHz, Chloroform-*d*) δ 7.96 (d, 1H), 7.43 (dd, J = 7.7, 1.1 Hz, 1H), 7.34-7.20 (m, 5H), 2.73 (s, 2H), 1.20 (s, 6H).

^{13}C NMR (126 MHz, $CDCl_3$) δ 149.8, 146.7, 137.2, 129.4, 128.6, 128.5, 128.3, 128.0, 127.6, 126.8, 124.3, 120.6, 119.8, 117.2, 44.0, 35.1, 27.3.

Sequence to phenanthrene based azocompounds

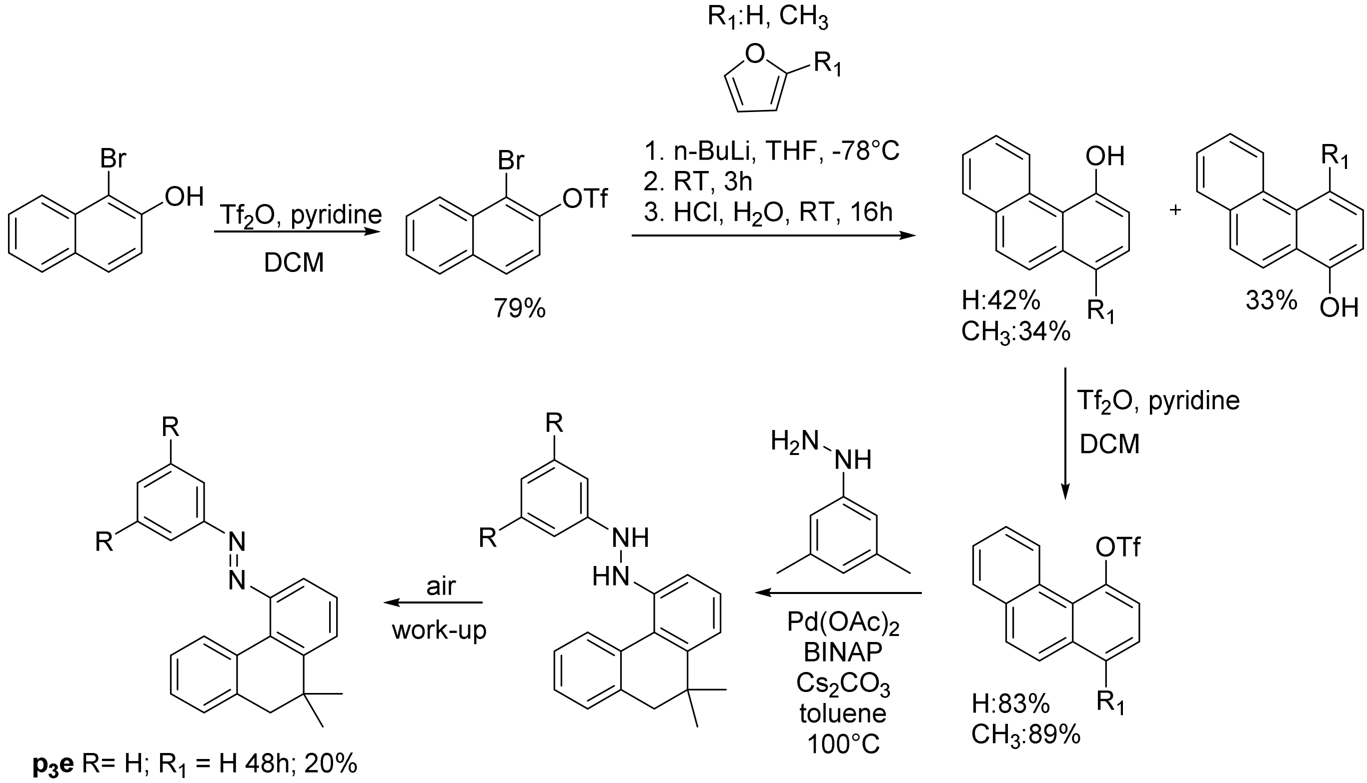

p3e R= H; R_1 = H 48h; 20%
p3f R= CH_3; R_1 = H 48h; 18%
p3g R= H; R_1 = CH_3 48h; 21%
p3h R= CH_3; R_1 = CH_3 48h; 16%

1-Bromonaphthalen-2-yl trifluoromethanesulfonate[12]

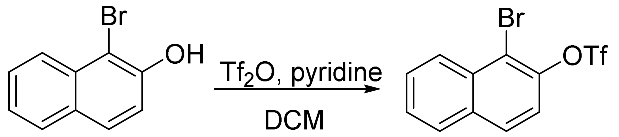

1-Bromonaphthalene-2-ol (5 g, 22.4 mmol) was weighed into a flame-dried three-necked flask equipped with a septum, stopper, and connected to a vacuum-inert gas line. The flask was evacuated and purged with argon several times, then 3.6 g (49.3 mmol) of dry pyridine in 40 ml of dry DCM was added under constant argon flow. The mixture was stirred in an ice bath for 30 minutes, followed by the dropwise addition of 6.4 g (22.4 mmol) of trifluoromethanesulfonic anhydride, and the solution was stirred for 1 hour at 0 °C. The reaction was quenched by slow addition of 60 ml of 0.5 M aqueous HCl followed by extraction by DCM (3×50 ml). The organic phase was dried over anhydrous Na_2SO_4 and evaporated. The oily residue was purified by column chromatography (silica gel, PE:EtOAc 20:1). After evaporation, 6.3 g (79%) of pale yellow oily liquid was obtained.

^{1}H NMR (500 MHz, Chloroform-*d*) δ 8.28 (d, *J* = 10 Hz, 1H), 7.85 (d, *J* = 5 Hz, 2H), 7.66 (t, *J* = 5 Hz, 1H), 7.59 (t, *J* = 5 Hz, 1H), 7.41 (d, *J* = 10 Hz, 1H).

^{13}C APT NMR (126 MHz, $CDCl_3$) δ 145.0, 132.9, 132.6, 129.7, 128.7, 128.3, 127.7, 127.6, 119.9, 118.7 (q, *J* = 318 Hz); 116.2.

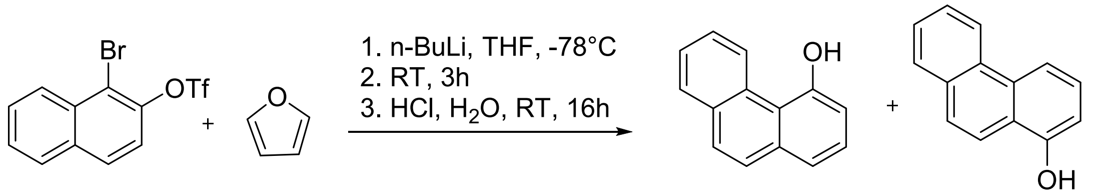

Phenanthren-1-ol[12]

1-Bromonaphthalen-2-yl-trifluoromethanesulfonate (7.8 g 22 mmol) was weighed into a three-necked flask, which was dried and heated under vacuum. The flask was then purged with argon, followed by the addition of 80 ml of dry THF and 9.8 g (10.5 ml, 144 mmol) of dry furan. The mixture was cooled to -78 °C, and 9.2 ml of *n*-BuLi (2.5M in hexane) was added dropwise. The reaction mixture was stirred for 30 minutes at -78 °C, then allowed to warm to room temperature, and stirred for an additional 3 hours. Under inert conditions, 50 ml of 2M aqueous HCl was added, and the reaction mixture was stirred for another 16 hours. The solution was poured into water (100 ml) and extracted with EtOAc (3×50 ml). The extract was washed with water (2×50 ml), and brine (2×50 ml), and dried over anhydrous Na_2SO_4. After evaporation, the mixture was purified by column chromatography (silica gel, PE:EtOAc 20:1). 1.8 g (42%) of phenanthren-1-ol was obtained as a yellow crystalline substance with a melting point of 153-155 °C. Another fraction isolated from the reaction mixture was phenanthren-4-ol.

^{1}H NMR (500 MHz, DMSO-*d6*) δ 10.20 (s, 1H), 8.74 (d, *J* = 10 Hz, 1H), 8.24 (d, *J* = 5 Hz, 1H), 8.12 (d, *J* = 10 Hz, 1H), 7.95 (d, *J* = 10 Hz, 1H), 7.76 (d, *J* = 10 Hz, 1H), 7.63 (t, *J* = 10 Hz, 1H), 7.48 (t, *J* = 10 Hz, 1H), 7.07 (d, *J* = 10 Hz, 1H).

^{13}C APT NMR (126 MHz, DMSO-*d6*) δ 151.9, 132.1, 131.9, 130.0, 128.6, 126.7, 126.6, 126.2, 123.1, 121.8, 119.9, 115.5, 110.6.

Phenanthren-4-ol[12]

Yield 1.4 g (33%) beige crystals melting at 109-111 °C.

^{1}H NMR (500 MHz, DMSO-*d6*) δ 9.64 (d, *J* = 10 Hz, 1H), 7.87 (d, *J* = 10 Hz, 1H), 7.71 (d, *J* = 5 Hz, 1H), 7.68 (d, *J* = 10 Hz, 1H), 7.65 (dt, *J* = 10 Hz, *J* = 1 Hz, 1H), 7.58 (dt, J_1 = 10 Hz, J_2 = 1 Hz, 1H), 7.49 (d, *J* = 10 Hz, 1H), 7.39 (t, *J* = 10 Hz, 1H), 6.93 (d, *J* = 5 Hz, 1H), 5.72 (s, 1H).

^{13}C APT NMR (126 MHz, DMSO-*d6*) δ 154.3, 134.9, 132.5, 130.3, 128.5, 128.2, 128.0, 127.0, 126.5, 126.3, 126.0, 121.7, 119.4, 113.2

1-Methylphenanthren-4-ol[12]

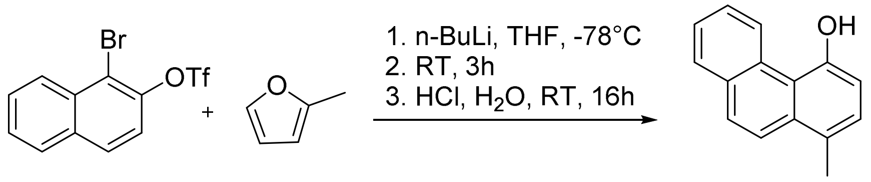

1-Methylphenanthren-4-ol was synthesized using the same procedure as phenanthren-1-ol and phenanthren-4-ol. For the synthesis, 4.5 g (12.7 mmol) of 1-bromonaphthalen-2-yl-trifluoromethanesulfonate, 45 ml of dry THF, 5.2 g (63.5 mmol) of dry 2-methylfuran, and 6.4 ml of n-BuLi (2.2 M in hexane, 14 mmol) were used. The mixture was separated by column chromatography (silica gel, PE:EtOAc 20:1). This yielded 0.9 g (34%) of yellow crystalline substance with a melting point of 102-103°C.

^{1}H NMR (500 MHz, DMSO-*d6*) δ 9.68 (d, *J* = 10 Hz, 1H), 7.89 (m, 1H), 7.88 (m, 1H), 7.77 (d, *J* = 10 Hz, 1H), 7.64 (dt, *J* = 10 Hz, *J* = 5 Hz, 1H), 7.58 (dt, *J*= 5 Hz, *J* = 2 Hz, 1H), 7.24 (t, *J* = 10 Hz, 1H), 6.85 (d, *J* = 10 Hz, 1H), 5.51 (s, 1H), 2.66 (s, 3H).

^{13}C APT NMR (126 MHz, DMSO-*d6*) δ 152.8, 133.0, 132.2, 130.6, 128.7, 128.1, 127.8, 127.4, 127.1, 126.4, 125.9, 123.0, 119.5, 112.6, 19.9.

Phenanthren-4-yl trifluoromethanesulfonate (p1e)

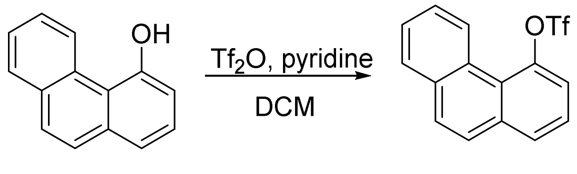

Phenanthrene-4-yltrifluoromethanesulfonate was prepared using the same procedure described for the synthesis of 1-naphthalen-2-yltrifluoromethanesulfonate. 1 g (5.1 mmol) of phenanthrene-4-ol, 0.95 g (12 mmol) of pyridine, 30 ml of DCM, and 1.5 g (5.2 mmol) of trifluoromethanesulfonic anhydride were used. After separation by column chromatography (silica gel, PE:EtOAc 20:1), 1.38 g (83%) of white crystalline substance with a melting point of 67-69 °C was obtained

^{1}H NMR (500 MHz, Chloroform-*d*) δ 9.18 (d, *J* = 10 Hz, 1H), 7.91 (d, *J* = 10 Hz, 1H), 7.87 (d, *J* = 10 Hz, 1H), 7.77 (d, *J* = 10 Hz, 1H), 7.69 (m, 3H), 7.62 (d, *J* = 5 Hz, 1H), 7.56 (t, *J* = 10 Hz, 1H).

^{13}C APT NMR (126 MHz, $CDCl_3$) δ 153.1, 147.2, 133.2, 129.2, 129.0, 128.9, 127.6, 127.4, 127.2, 127.1, 126.2, 126.1, 123.0, 120.5, 118.7 (q, *J* = 319 Hz).

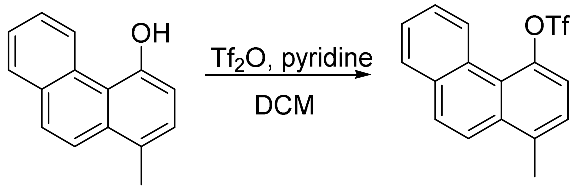

1-Methylphenanthrene-4-yltrifluoromethanesulfonate (p1g)

1-Methylphenanthrene-4-yltrifluoromethanesulfonate was prepared using the same procedure described for the synthesis of 1-naphthalen-2-yltrifluoromethanesulfonate. 0.85 g (4.1 mmol) of 1-methylphenanthrene-4-ol, 0.8 g (10 mmol) of pyridine, 20 ml of DCM, and 1.2 g (4.1 mmol) of trifluoromethanesulfonic anhydride were used. After separation by column chromatography (silica gel, PE:EtOAc 20:1), 1.24 g (89%) of white crystalline substance with a melting point of 49-51 °C was obtained

^{1}H NMR (500 MHz, Chloroform-*d*) δ 8.77 (d, *J* = 10 Hz, 1H), 7.95 (d, *J* = 10 Hz, 1H), 7.87 (m, 1H), 7.80 (d, *J* = 10 Hz, 1H), 7.60 (m, 2H), 7.40 (m, 2H), 3.03 (s, 3H).

^{13}C APT NMR (126 MHz, $CDCl_3$) δ 144.5, 136.2, 133.2, 131.8, 130.8, 130.3, 129.5, 126.1, 118.8(q, *J* = 318 Hz, CF_3), 118.7, 117.7, 27.1.

General procedure for the two-step synthesis of (modified)phenanthrene-derived azo compounds

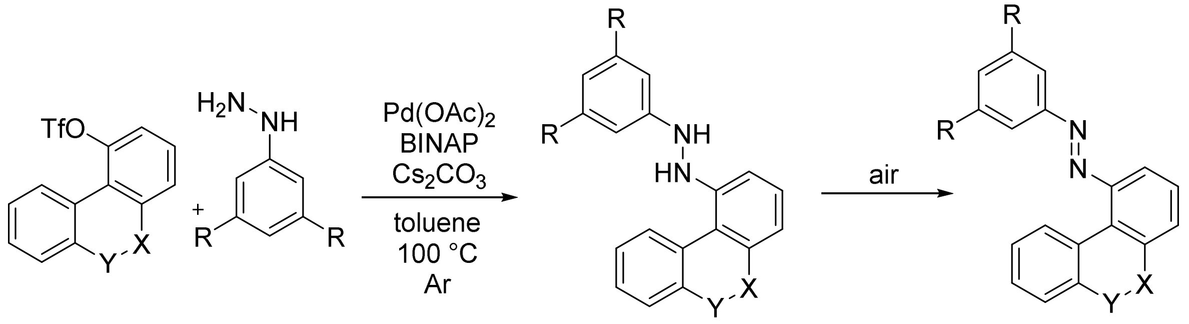

Into an argon-filled flask, dry toluene was introduced, and the corresponding arylhydrazine, triflate, Cs_2CO_3, and BINAP were suspended in it (quantities are provided subsequent Table). The suspension was bubbled with argon for 10 minutes. Subsequently, $Pd(OAc)_2$ was added to the mixture, and the mixture was further bubbled for an additional 5 minutes. The flask was placed in an oil bath, and the reaction mixture was heated to 100 °C for 24 to 72 hours, depending on the substrate's substitution. After complete consumption of the triflate, the mixture was cooled to room temperature and diluted with 20 ml of H_2O and 20 ml of EtOAc. Subsequently, extraction was performed 3 x 50 ml of EtOAc. The organic layers were combined, washed with brine, and filtered. During extraction, the oxidation process of the hydrazine to the corresponding diazene was initiated by atmospheric O_2. To achieve complete conversion, the mixture was stirred overnight in an open Erlenmeyer flask. The next day, the mixture was evaporated on a rotary evaporator. The product was purified on a silica gel column using a mixture of Et_2O/hexane (2:8) as the eluent or by flash chromatography with a gradient elution of Et_2O/hexane (1/99 → 10/90) over 30 minutes. The anthracene derivatives were purified by flash chromatography using PE/toluene 4:1 and crystalized from *n*-heptane.

Table S1.

compound	p1a [mmol]	p1a [mg]	p1c [mmol]	p1c [mg]	Ar-NH-NH2 [mg]	Cs_2CO_3 [mg]	BINAP [mg]	$Pd(OAc)_2$ [mg]	Toluene [ml]	Time [h]
**p3a**	4.39	1370	-	-	550	2044	247	45	45	24
**p3b**	2	700	-	-	350	1057	140	23.8	24	24
**p3c**	-	-	2.68	800	290	1106	145	25	25	72
**p3d**	-	-	2.68	800	365	1106	145	25	25	72

compound	p1e [mmol]	p1e [mg]	p1g [mmol]	p1g [mg]	Ar-NH-NH_2 [mg]	Cs_2CO_3 [mg]	BINAP [mg]	$Pd(OAc)_2$ [mg]	Toluene [ml]	Time [h]
**p3e**	1	330			130	530	630	23	20	48
**p3f**	1	330			160	530	630	23	20	48
**p3g**			1	340	130	530	630	23	20	48
**p3h**			1	340	160	530	630	23	20	48

(*E*)-1-(6*H*-Benzo[*c*]chromen-1-yl)-2-phenyldiazene (p3a)

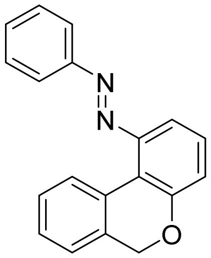

Reaction time 24 h; yield: 570 mg (44%) orange amorphous compound with m.p. 76.5-77.5 °C.

^{1}H NMR (500 MHz, Chloroform-*d*) δ 7.98 (d, *J* = 7.2 Hz, 2H), 7.63 (d, *J* = 7.4 Hz, 1H), 7.59-7.49 (m, 3H), 7.39-7.29 (m, 4H), 7.26 (d, *J* = 6.9 Hz, 1H), 7.17 (d, *J* = 6.2 Hz, 1H), 5.14 (s, 2H).

^{13}C APT NMR (126 MHz, $CDCl_3$) δ 156.6, 152.8, 150.3, 133.3, 131.3, 131.0, 129.3, 128.8, 128.7, 128.1, 127.7, 124.5, 123.5, 122.0, 119.3, 110.0, 69.1.

HRMS: Calculated for $C_{19}H_{15}N_2O$ [M+H$^+$]: 287.1184; found 287.1180.

Elemental anal.: Calc. for $C_{19}H_{14}N_2O$: C: 79.70; H: 4.93; N: 9.78; O: 5.59

Found: C: 79.94; H: 4.84; N: 9.48

(*E*)-1-(6*H*-Benzo[*c*]chromen-1-yl)-2-(3,5-dimethylphenyl)diazene (p3b)

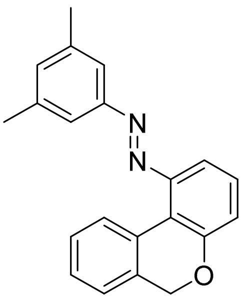

Reaction time 24 h; yield: 320 mg (53%) orange amorphous compound with m.p. 118-121 °C.

^{1}H NMR (500 MHz, Chloroform-*d*) δ 7.59 (d, *J* = 6.8 Hz, 3H), 7.40-7.24 (m, 5H), 7.16 (d, *J* = 9.7 Hz, 2H), 5.15 (s, 2H), 2.43 (s, 6H).

^{13}C APT NMR (126 MHz, $CDCl_3$) δ 156.5, 153.0, 150.5, 139.0, 133.3, 133.0, 131.0, 128.9, 128.7, 128.1, 127.7, 124.5, 121.7, 121.3, 119.0, 110.0, 69.1, 21.3.

HRMS: Calculated for $C_{21}H_{19}N_2O$ [M+H$^+$]: 315.1497; found 315.1500.

Elemental anal.: Calculated for $C_{21}H_{18}N_2O$ C: 80.23; H: 5.77; N: 8.91; O: 5.09

Found: C: 80.51; H: 5.63; N: 8.55

(*E*)-1-(10,10-Dimethyl-9,10-dihydrophenanthren-4-yl)-2-phenyldiazene (p3c)

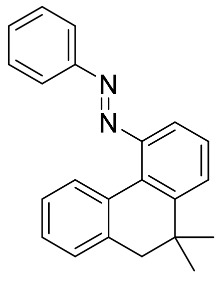

Reaction time 72 h; yield: 230 mg (33%) red resin.

^{1}H NMR (500 MHz, DMSO-*d6*) δ 7.92-7.84 (m, 2H), 7.66-7.55 (m, 3H), 7.52-7.42 (m, 3H), 7.38-7.23 (m, 4H), 2.85 (s, 2H), 1.28 (d, *J* = 13.5 Hz, 6H).

^{13}C APT NMR (126 MHz, $CDCl_3$) δ 152.8, 149.7, 148.0, 137.8, 132.8, 132.1, 131.8, 130.9, 129.2, 128.0, 127.9, 127.6, 126.1, 125.9, 123.4, 114.5, 44.4, 34.7, 27.6.

HRMS: Calculated for $C_{22}H_{21}N_2$ [M+H$^+$]: 313.1705; found 313.1700.

Elemental anal.: Calculated for $C_{22}H_{20}N_2$ C: 84.58; H: 6.45; N: 8.97

Found: C: 83.91; H: 6.53; N: 7.62.

(*E*)-1-(10,10-Dimethyl-9,10-dihydrophenanthren-4-yl)-2-(3,5-dimethylphenyl)diazene (p3d)

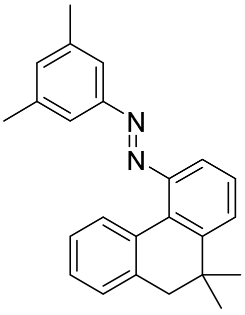

Reaction time 72 h; yield 180 mg (26%) red resin.

^{1}H NMR (500 MHz, DMSO-*d6*) δ 7.61 (dd, *J* = 6,2, 2,7 Hz, 1H), 7.49 (d, *J* = 9.0 Hz, 2H), 7.44 (d, *J* = 7.3 Hz, 2H), 7.42 – 7.34 (m, 1H), 7.34 – 7.29 (m, 2H), 7.29 – 7.23 (m, 1H), 7.22 (s, 1H), 2.84 (s, 2H), 2.40 – 2.39 (d, *J* = 4.0 Hz, 6H), 1.27 (d, *J* = 14.1 Hz, 6H),

^{13}C APT NMR (126 MHz, DMSO-*d6*) δ 153.4, 149.9, 149.5, 148.4, 139.2, 137.9, 133.1, 132.0, 131.2, 130.9, 128.5, 128.2, 126.7, 126.3, 125.7, 124.0, 121.1, 114.8, 44.3, 34.8, 27.6, 21.3.

HRMS: Calculated for $C_{24}H_{25}N_2$ [M+H$^+$]: 341.1973; found 341.2010.

Elemental anal.: Calculated for $C_{24}H_{24}N_2$ C: 84.67; H: 7.11; N: 8.23

Found: C: 84.09; H: 7.08; N: 7.54.

1-Phenyl-2-(phenanthren-4-yl)diazene (p3e)

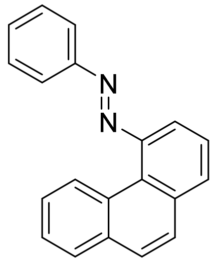

Yield: 56 mg (20%); red crystalline powder with m.p. 78–80 °C.

^{1}H NMR (500 MHz, Chloroform-*d*) δ 8.84 (d, *J* = 10 Hz, 1H), 8.12 (d, *J* = 5 Hz, 1H), 8.01 (d, *J* = 5 Hz, 1H), 7.94 (d, *J* = 5 Hz, 1H), 7.84 (d, *J* = 10 Hz, 1H), 7.81 (d, *J* = 10 Hz, 1H), 7.61 (m, 7H).

^{13}C APT NMR (126 MHz, $CDCl_3$) δ 153.0, 152.5, 134.0, 133.8, 131.5, 131.3, 131.1, 130.0, 129.4, 128.4, 128.1, 127.0, 126.7, 126.6, 126.5, 126.4, 123.6, 115.0.

HRMS calculated for $C_{20}H_{15}N_2$ [M+H$^+$]: 283.1235; found: 283.1231.

Elemental anal.: Calculated for $C_{24}H_{14}N_2$ C: 85.08; H: 5.00; N: 9.92

Found: C: 85.20; H: 4.89; N: 9.89.

1-(3,5-Dimethylphenyl)-2-(phenanthren-4-yl)diazene (p3f)

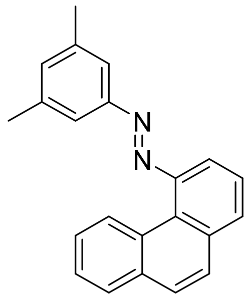

Yield: 55 mg (18%); red crystalline powder with m.p. 85–86°C.

^{1}H NMR (500 MHz, Chloroform-*d*) δ 8.81 (d, *J* = 5 Hz, 1H), 7.99 (dd, *J* = 5 Hz; *J* = 1 Hz, 1H), 7.93 (dd, *J* = 5 Hz; *J* = 1 Hz, 1H), 7.83 (d, *J* = 5 Hz, 1H), 7.81 (d, *J* = 5 Hz, 1H), 7.73 (bs, 2H), 7.65 (t, *J* = 10 Hz, 1H), 7.59 (m, 3H), 7.22 (bs, 1H), 2.48 (s, 6H).

^{13}C APT NMR (126 MHz, $CDCl_3$) δ 153.2, 152.6, 139.1, 133.9, 133.8, 133.0, 131.6, 130.9, 130.0, 128.4, 128.1, 127.0, 126.6, 126.5, 126.4, 121.4, 115.0, 21.4.

HRMS Calculated for $C_{22}H_{19}N_2$ [M+H$^+$]: 311.1548; found: 311.1547.

Elemental anal.: Calculated for $C_{22}H_{18}N_2$ C: 85.13; H: 5.85; N: 9.03

Found: C: 85.24; H: 5.74; N: 9.05.

1-Phenyl-2-(1-methylphenanthren-4-yl)diazene (p3g)

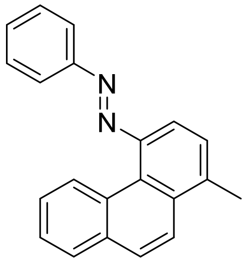

Yield: 63 mg (21%); red crystalline powder with m.p. 90–92 °C.

^{1}H NMR (500 MHz, Chloroform-*d*) δ 8.80 (d, *J* = 10 Hz, 1H), 8.10 (dt, *J* = 5 Hz, J_2 = 1 Hz; 2H), 8.02 (d, *J* = 10 Hz, 1H), 7.94 (dd, J_1 = 10 Hz, *J* = 2 Hz, 1H), 7.88 (d, *J* = 10 Hz, 1H), 7.58 (m, 7H), 2.83 (s, 3H).

^{13}C APT NMR (126 MHz, $CDCl_3$) δ 153.0, 151.0, 137.7, 133.5, 132.3, 131.9, 131.0, 130.3, 129.4, 128.2, 127.9, 127.8, 126.7, 126.5, 126.4, 123.5, 122.8, 114.3, 20.7.

HRMS Calculated for $C_{21}H_{17}N_2$ [M+H$^+$]: 297.1392; found: 297.1387.

Elemental anal.: Calculated for $C_{21}H_{16}N_2$ C: 85.11; H: 5.44; N: 9.45

Found: C: 85.20; H: 5.42; N: 9.38

1-(3,5-Dimethylphenyl)-2-(1-methylphenanthren-4-yl)diazene (p3h)

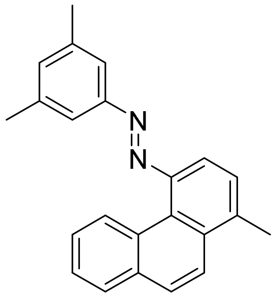

Yield: 52 mg (16%); red crystalline powder with m.p. 108–110 °C.

^{1}H NMR (500 MHz, Chloroform-*d*) δ 8.78 (d, *J* = 10 Hz, 1H), 8.01 (d, *J* = 5 Hz, 1H), 7.93 (d, *J* = 5 Hz, 1H), 7.87 (d, *J* = 10 Hz, 1H), 7.71 (bs, 2H), 7.60 (m, 2H), 7.51 (dd, *J* = 10 Hz, *J* = 5 Hz, 2H), 7.19 (s, 1H), 2.82 (s, 3H), 2.47 (s, 6H).

^{13}C APT NMR (126 MHz, $CDCl_3$) δ 153.3, 151.2, 139.0, 137.3, 133.5, 132.7, 132.3, 132.0, 130.3, 128.2, 127.9, 127.8, 126.7, 126.5, 126.4, 122.8, 121.4, 114.3, 21.3, 20.6.

HRMS Calculated for $C_{23}H_{21}N_2$ [M+H$^+$]: 325.1705; found: 325.1704.

Elemental anal.: Calculated for $C_{23}H_{20}N_2$ C: 85.15; H: 6.21; N: 8.63

Found: C: 85.07; H: 6.30; N: 8.58

Mechanistic findings

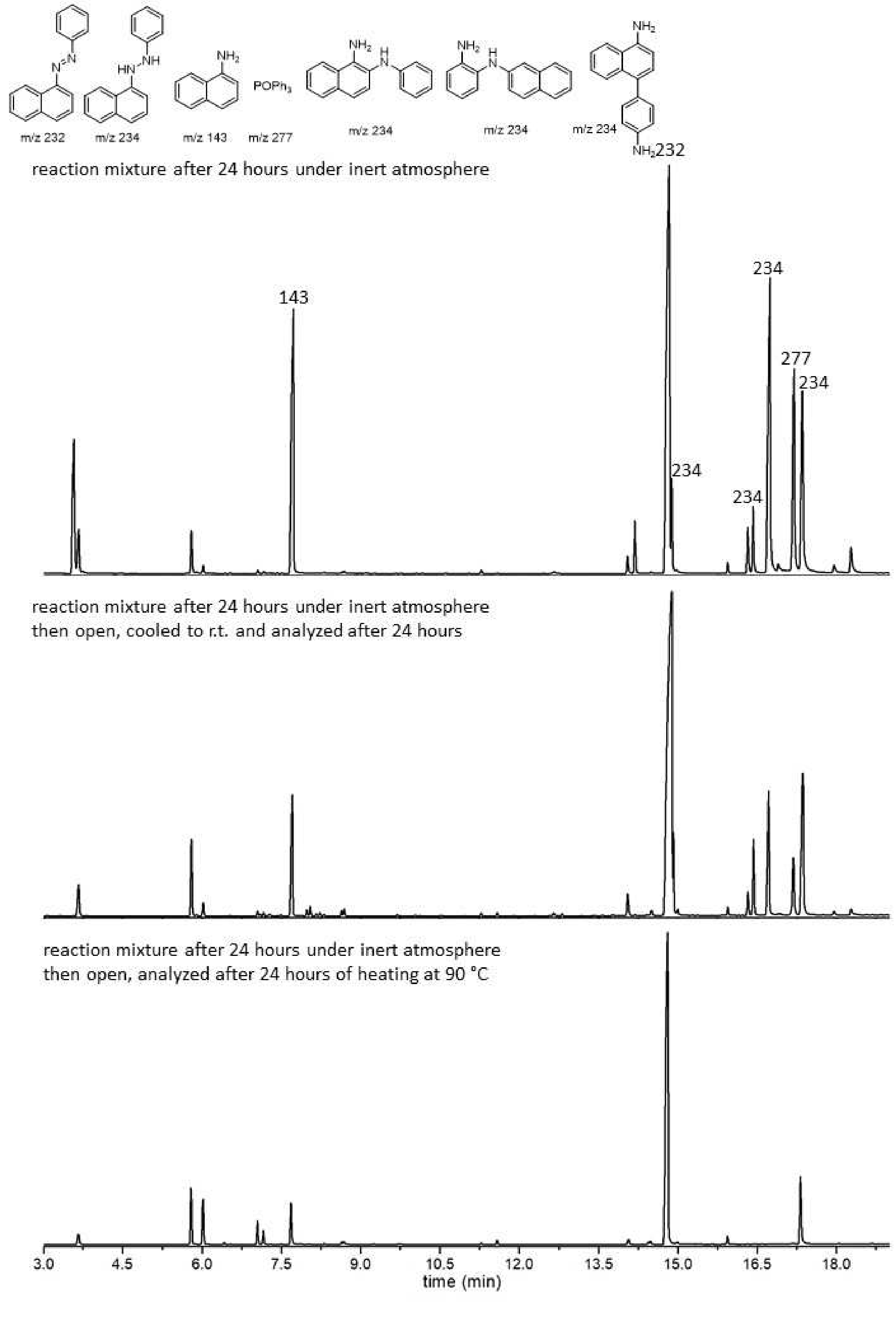

Figure S1. Evidence for the presence of 1,2-diarylhydrazine as a reaction intermediate.

GC-MS chromatogram of the reaction of 1-bromonaphthalene with phenylhydrazine running for all time under argon atmosphere (on the top) shows presence of compounds with masses corresponding to the azo (m/z 232) as well as hydrazo (m/z 234) compounds. The interpretation of this result must be cautious, as we have verified through independent experiments that during EI ionization both partial oxidation of hydrazine to an azo compound and rearrangements of hydrazine to other substances with the same mass occur (these compounds are only artefacts of EI ionization).

Spectrum in the middle is the same mixture analyzed after further 24 hours of standing opened on the air at the room temperature. This shows that the oxidation of hydrazine is slow at r. t.

Spectrum on the bottom is from the same reaction mixture that was heated to 90 °C for further 24 hours. It shows that oxidation of hydrazoarene was successfully completed after that time. Thus the elevated temperature is beneficial.

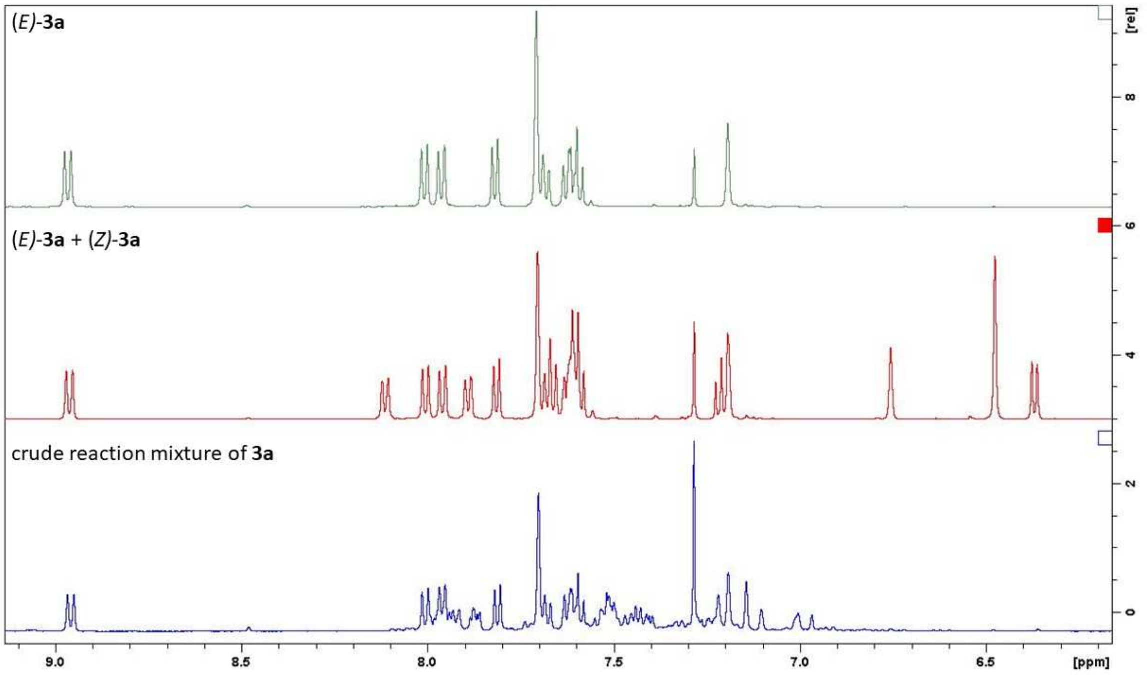

Figure S2. Comparison of ^{1}H NMR spectra ($CDCl_3$) of: isolated **3a** (top); **3a** irradiated by 380 nm LED for 30 min (middle); crude reaction mixture from the experiment with MeOBIPHEP catalyst (bottom).

Comparison of the spectra shows that there are no signals of **(Z)-3a** in the crude reaction mixture.

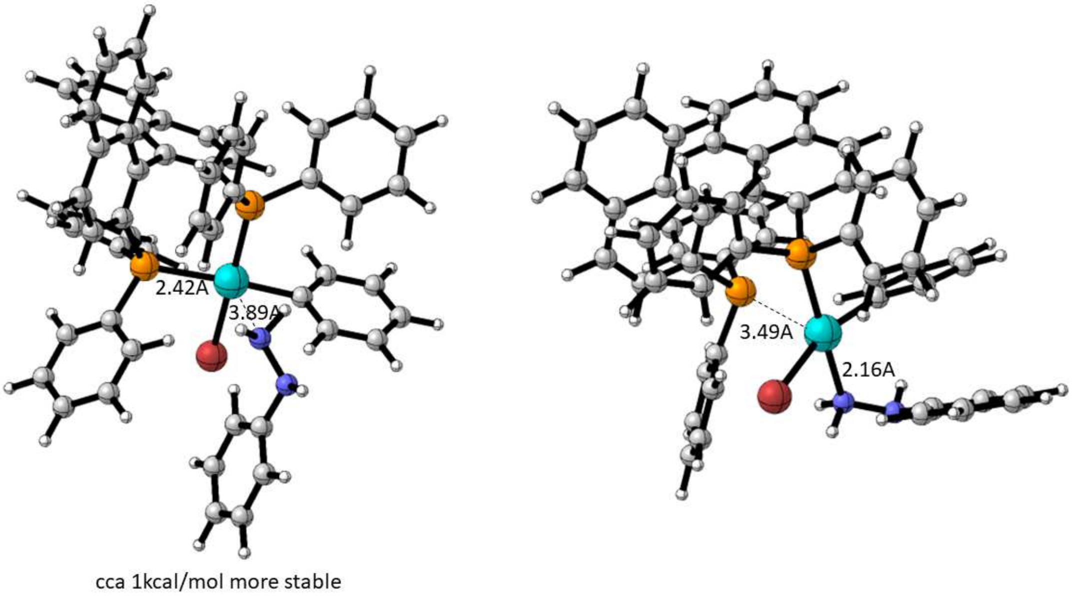

Figure S3. Comparison of calculated structures and important atom distances for **Int2a** and **Int2b**. D3BJ-B3LYP/ 6-311+G** (SDD for Pd). Geometry taken from the gas phase calculations.

General procedure for aryltrifluoromethanesulfonates

Corresponding phenol (5 g, 0.0346 mol) was dissolved in DCM (100 ml), followed by addition of pyridine (3.35 mL, 0.0416 mol). Triflic anhydride (8.75 mL, 0.0520 mol) was added dropwise to mixture cooled to 0 °C (ice bath), and let slowly warmed to room temperature and stirred overnight. Subsequently, mixture was washed with saturated solution of $NaHCO_3$ (100 ml) and aqueous phase was extracted with DCM (3 × 50 ml). Combined organic phases were dried over $NaSO_4$, and solvent was removed under reduced pressure. Crude products were purified by column chromatography with mobile phase Hexane:DCM (4:1).

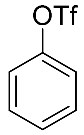

phenyl trifluoromethanesulfonate

colourless oil, 98%

^{1}H NMR (500 MHz, Chloroform-*d*) δ 7.46 (dd, *J* = 8.7, 7.0 Hz, 2H), 7.43 – 7.37 (m, 1H), 7.32 – 7.27 (m, 2H).

^{13}C{1H}NMR (126 MHz, $CDCl_3$) δ 149.80, 130.42, 128.55, 121.47, 118.8 (q, *J*= 319.7 Hz).

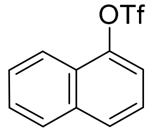

naphthalen-1-yl trifluoromethanesulfonate

colourless oil, 94%

^{1}H NMR (500 MHz, Chloroform-*d*) δ 8.12 – 8.08 (m, 1H), 7.95 – 7.91 (m, 1H), 7.90 – 7.85 (m, 1H), 7.69 – 7.63 (m, 1H), 7.63 – 7.58 (m, 1H), 7.52 – 7.46 (m, 2H).

^{13}C APT NMR (126 MHz, $CDCl_3$) δ 145.8, 135.0, 128.7, 128.2, 128.0, 127.5, 126.5, 125.3, 120.9, 118.8 (q, *J* = 321.1 Hz), 117.9.

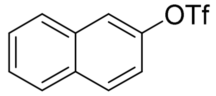

naphthalen-2-yl trifluoromethanesulfonate

colourless oil, 97%

^{1}H NMR (500 MHz, Chloroform-*d*) δ 7.93 (d, *J* = 9.0 Hz, 1H), 7.91 – 7.85 (m, 2H), 7.76 (d, *J* = 2.5 Hz, 1H), 7.62 – 7.55 (m, 2H), 7.38 (dd, *J* = 9.0, 2.5 Hz, 1H).

$^{13}C\{1H\}$NMR (126 MHz, $CDCl_3$) δ 147.2, 133.4, 132.5, 130.7, 128.1, 128.0, 127.7, 127.3, 119.6, 119.3, 118.9 (q, *J* = 320.3 Hz).

General procedure for azo compounds

$Pd(OAc)_2$ (8 mg, 0.0357 mmol, 5 mol%) and BINAP (45 mg, 0.0713 mmol, 10 mol%) were added to the Schlenk flask, followed by addition of dry toluene (4 mL). Mixture was degassed by N_2 for 10 min and subsequently heated to 80 °C for 15 min. After cooling down to room temperature was added aryl halogenide or aryltrifluormethanesulfonate (0.7133 mmol, 1 equiv.), arylhydrazine (1.070 mmol, 1.5 equiv.), Cs_2CO_3 (256 mg, 0.784 mmol, 1.1 equiv), dry toluene (4 mL) and mixture was degassed by N_2 for 10 min. Subsequently, mixture was stirred at certain temperature for 24 h in Schlenk flask open to air. After 24 h, mixture was filtered through plug of silica and eluted with EtOAc (3 × 10 mL) and solvent was subsequently removed under reduced pressure. Crude product were purified by flash chromatography using hexane/DCM (4:1).

General procedure for free base arylhydrazines

Arylhydrazine×HCl (0.0028 mol) was dissolved in DCM (15 mL) and washed with 2 M aqueous solution NaOH (40 mL), organic phase was subsequently washed with saturated solution of NaCl (30 mL), dried over $NaSO_4$, solvent was evaporated under reduced pressure and fresh arylhydrazines were used directly to azo-coupling reaction.

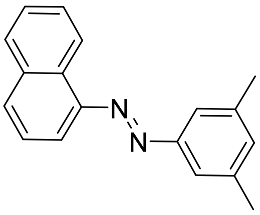

(*E*)-1-(3,5-dimethylphenyl)-2-(naphthalen-1-yl)diazene 3a

Red solid, MP: 99-100 °C

^{1}H NMR (500 MHz, Chloroform-*d*) δ 8.94 (d, *J* = 8.4 Hz, 1H), 7.98 (d, *J* = 8.1 Hz, 1H), 7.94 (d, *J* = 8.2 Hz, 1H), 7.79 (dd, *J* = 7.5, 1.2 Hz, 1H), 7.68 (d, *J* = 1.8 Hz, 2H), 7.67 – 7.64 (m, 1H), 7.62 – 7.54 (m, 2H), 7.17 (s, 1H), 2.47 (s, 6H).

^{13}C{1H}NMR (126 MHz, $CDCl_3$) δ 153.5, 148.1, 138.9, 134.4, 132.9, 131.3, 131.2, 128.1, 126.9, 126.6, 125.8, 123.6, 121.1, 111.9, 21.5.

FT-IR (ATR) cm^{-1}: 3058, 2916, 1604, 1504, 1342, 1201, 1118, 855, 799, 769, 582.

HRMS: Calculated for $C_{18}H_{17}N_2$ $[M+H]^+$: 261.13863; Found: 261.13891

Elemental anal.: Calc. for $C_{18}H_{16}N_2$: C: 83.04; H: 6.19; N: 10.76

Found: C: 83.27 ± 0.01 ; H: 6.88 ± 0.02; N: 10.33 ± 0.10

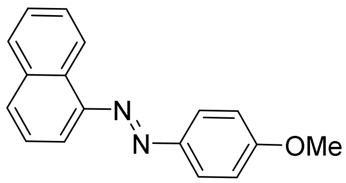

(*E*)-1-(4-methoxyphenyl)-2-(naphthalen-1-yl)diazene 3b

Red solid, MP: 62-63 °C

^{1}H NMR (500 MHz, Chloroform-*d*) δ 8.92 (d, *J* = 8.4 Hz, 1H), 8.09 – 8.04 (m, 2H), 7.96 (d, *J* = 8.1 Hz, 1H), 7.93 (d, *J* = 7.6 Hz, 2H), 7.80 (dd, *J* = 7.5, 1.1 Hz, 1H), 7.64 (ddd, *J* = 8.4, 6.8, 1.4 Hz, 1H), 7.61 – 7.54 (m, 2H), 7.09 – 7.05 (m, 2H), 3.92 (s, 3H).

^{13}C{1H}NMR (126 MHz, $CDCl_3$) δ 162.3, 148.0, 147.8, 134.4, 131.3, 130.7, 128.0, 126.7, 126.5, 125.8, 125.2, 123.6, 114.4, 111.8, 55.8.

FT-IR (ATR) cm^{-1}: 3050, 2836, 1597, 1498, 1386, 1247, 1136, 1027, 768, 516.

HRMS: Calculated for $C_{17}H_{15}N_2O$ $[M+H]^+$: 263.11789; Found: 263.11832

Elemental anal.: Calc. for $C_{17}H_{14}N_2O$: C: 77.84; H: 5.38; N: 10.68; O: 6.10

Found: C: 77.56 ± 0.04 ; H: 5.15 ± 0.15; N: 10.60 ± 0.04

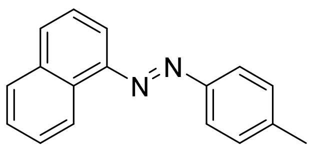

(*E*)-1-(naphthalen-1-yl)-2-(p-tolyl)diazene 3c

Orange solid, MP: 66-67 °C

^{1}H NMR (500 MHz, Chloroform-*d*) δ 8.96 – 8.91 (m, 1H), 7.98 (dd, *J* = 8.4, 2.3 Hz, 3H), 7.95 – 7.92 (m, 1H), 7.81 (dd, *J* = 7.5, 1.2 Hz, 1H), 7.65 (ddd, *J* = 8.3, 6.8, 1.4 Hz, 1H), 7.62 – 7.55 (m, 2H), 7.37 (d, *J* = 8.1 Hz, 2H), 2.47 (s, 3H).

^{13}C APT NMR (126 MHz, $CDCl_3$) δ 151.5, 147.9, 141.8, 134.4, 131.4, 131.1, 129.9, 128.0, 126.9, 126.8, 126.6, 125.8, 123.6, 123.3, 111.9, 21.7.

FT-IR (ATR) cm^{-1}: 3052, 2915, 1598, 1500, 1388, 1141, 1011, 769, 508.

HRMS: Calculated for $C_{17}H_{15}N_2$ $[M+H]^+$: 247.12298; Found: 247.1224

Elemental anal.: Calc. for $C_{17}H_{14}N_2$: C: 82.90; H: 5.73; N: 11.37

Found: C: 82.71 ± 0.45 ; H: 5.52 ± 0.20; N: 11.43 ± 0.13

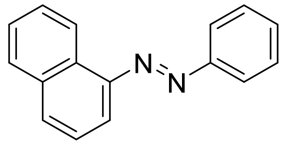

(*E*)-1-(naphthalen-1-yl)-2-phenyldiazene 3d

Red solid, MP: 66-67 °C

^{1}H NMR (500 MHz, Chloroform-*d*) δ 8.96 (d, *J* = 8.4 Hz, 1H), 8.08 (d, *J* = 7.7 Hz, 2H), 8.00 (d, *J* = 8.1 Hz, 1H), 7.95 (d, *J* = 8.1 Hz, 1H), 7.85 (d, *J* = 7.4 Hz, 1H), 7.67 (ddd, *J* = 8.4, 6.7, 1.3 Hz, 1H), 7.63 – 7.56 (m, 4H), 7.55 – 7.50 (m, 1H).

^{13}C APT NMR (126 MHz, $CDCl_3$) δ 153.3, 147.9, 134.4, 131.5, 131.4, 131.2, 129.3, 128.1, 126.9, 126.6, 125.8, 123.6, 123.3, 122.9, 111.9.

FT-IR (ATR) cm^{-1}: 3049, 2923, 1507, 1388, 1214, 1070, 922, 759, 683, 500.

HRMS: Calculated for $C_{16}H_{13}N_2$ $[M+H]^+$: 233.10733; Found: 233.10754

Elemental anal.: Calc. for $C_{16}H_{12}N_2$: C: 82.73; H: 5.21; N: 12.06

Found: C: 83.08 ± 0.01 ; H: 5.62 ± 0.12; N: 11.42 ± 0.13

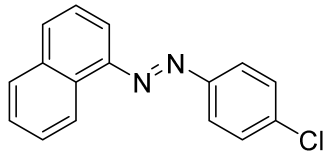

(*E*)-1-(4-chlorophenyl)-2-(naphthalen-1-yl)diazene 3e

Orange solid, MP: 124-125 °C

^{1}H NMR (500 MHz, Chloroform-*d*) δ 8.91 (dd, *J* = 8.3, 1.3 Hz, 1H), 8.03 – 7.97 (m, 3H), 7.94 (d, *J* = 8.1 Hz, 1H), 7.83 (dd, *J* = 7.6, 1.1 Hz, 1H), 7.66 (ddd, *J* = 8.4, 6.8, 1.3 Hz, 1H), 7.62 – 7.56 (m, 2H), 7.55 – 7.51 (m, 2H).

^{13}C APT NMR (126 MHz, $CDCl_3$) δ 151.7, 147.7, 137.1, 134.5, 131.8, 131.5, 129.5, 128.1, 127.1, 126.7, 125.7, 124.5, 123.5, 112.1.

FT-IR (ATR) cm^{-1}: 3049, 1571, 1481, 1342, 1219, 1088, 763, 501.

HRMS: Calculated for $C_{16}H_{12}ClN_2$ $[M+H]^+$: 267.06835; Found: 267.0699

Elemental anal.: Calc. for $C_{16}H_{11}ClN_2$: C: 72.05; H: 4.16; N: 10.50; Cl: 13.29

Found: C: 71.97 ± 0.02 ; H: 4.16 ± 0.01; N: 10.57 ± 0.08; Cl: 13.53 ± 0.34

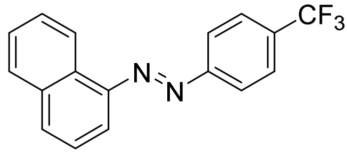

(*E*)-1-(naphthalen-1-yl)-2-(4-(trifluoromethyl)phenyl)diazene 3f

Orange solid, MP: 95-96 °C

^{1}H NMR (500 MHz, Chloroform-*d*) δ 8.96 – 8.90 (m, 1H), 8.16 – 8.10 (m, 2H), 8.08 – 8.02 (m, 1H), 7.98 – 7.94 (m, 1H), 7.89 – 7.85 (m, 1H), 7.85 – 7.81 (m, 2H), 7.73 – 7.66 (m, 1H), 7.64 – 7.56 (m, 2H).

^{13}C{1H}NMR (126 MHz, $CDCl_3$) δ 154.9, 147.6, 134.5, 132.5, 132.3 (q, *J*=32,7 Hz), 131.6, 128.2, 127.3, 126.8, 126.5 (q, *J*=3,8 Hz), 125.7, 124.1 (q, *J*=272,7 Hz), 123.4, 123.4, 112.2.

^{19}F{H} NMR (376 MHz, $CDCl_3$) δ -63.34

FT-IR (ATR) cm^{-1}: 3056, 1610, 1507, 1319, 1114, 850, 769, 507.

HRMS: Calculated for $C_{17}H_{12}F_3N_2$ $[M+H]^+$: 301.09471; Found: 301.0942

Elemental anal.: Calc. for $C_{17}H_{11}F_3N_2$: C: 68.00; H: 3.69; N: 9.33; F: 18.98

Found: C: 68.14 ± 0.21 ; H: 4.62 ± 0.08; N: 7.84 ± 0.13

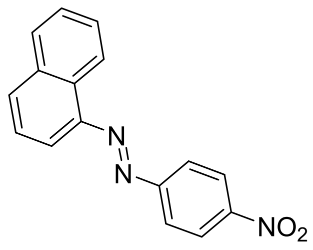

(*E*)-1-(naphthalen-1-yl)-2-(4-nitrophenyl)diazene 3g

Red solid, MP: 152-153 °C

^{1}H NMR (500 MHz, Chloroform-*d*) δ 8.94 (d, *J* = 8.4 Hz, 1H), 8.44 – 8.39 (m, 2H), 8.17 – 8.11 (m, 2H), 8.07 (d, *J* = 8.1 Hz, 1H), 7.96 (d, *J* = 8.2 Hz, 1H), 7.91 (dd, *J* = 7.6, 1.1 Hz, 1H), 7.70 (ddd, *J* = 8.4, 6.8, 1.3 Hz, 1H), 7.66 – 7.57 (m, 2H).

^{13}C APT NMR (126 MHz, $CDCl_3$) δ 156.2, 148.8, 147.5, 134.5, 133.2, 131.8, 128.3, 127.6, 126.9, 125.7, 124.9, 123.8, 123.3, 112.4.

FT-IR (ATR) cm^{-1}: 3096, 2854, 2441, 1827, 1606, 1524, 1333, 1211, 1102, 1008, 775, 596, 497, 413.

HRMS: Calculated for $C_{16}H_{12}N_3O_2$ $[M+H]^+$: 278.09240; Found: 278.09280

Elemental anal.: Calc. for $C_{16}H_{11}N_3O_2$: C: 69.31; H: 4.00; N: 15.15; O: 11.54

Found: C: 69.24 ± 0.33 ; H: 3.73 ± 0.11; N: 14.99 ± 0.15

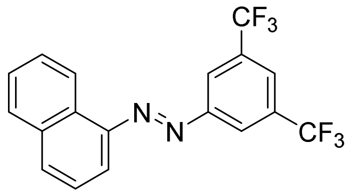

(*E*)-1-(3,5-bis(trifluoromethyl)phenyl)-2-(naphthalen-1-yl)diazene 3h

Orange solid, MP: 112-113 °C

^{1}H NMR (500 MHz, Chloroform-*d*) δ 8.92 (d, *J* = 8.5 Hz, 1H), 8.47 (s, 2H), 8.08 (d, *J* = 8.0 Hz, 1H), 8.01 (s, 1H), 7.97 (d, *J* = 8.2 Hz, 1H), 7.91 (dd, *J* = 7.6, 1.1 Hz, 1H), 7.72 (ddd, *J* = 8.4, 6.8, 1.3 Hz, 1H), 7.66 – 7.57 (m, 2H).

^{13}C APT NMR (126 MHz, $CDCl_3$) δ 153.4, 147.2, 134.5, 133.3, 132.9 (q, *J* = 33.8 Hz), 131.7, 128.3, 127.7, 126.9, 125.6, 123.9 - 123.8 (m), 123.3 (q, *J* = 273.2 Hz), 123.2, 112.7.

^{19}F{H} NMR (376 MHz, $CDCl_3$) δ -63.66

FT-IR (ATR) cm^{-1}: 3079, 1607, 1508, 1362, 1275, 1119, 896, 774, 680, 565.

HRMS: Calculated for $C_{18}H_{11}F_6N_2$ $[M+H]^+$: 369.08209; Found: 369.08271

Elemental anal.: Calc. for $C_{18}H_{10}F_6N_2$: C: 58.70; H: 2.74; N: 7.61; F: 30. 95

Found: C: 58.80 ± 0.04; H: 2.94 ± 0.10; N: 7.53 ± 0.02

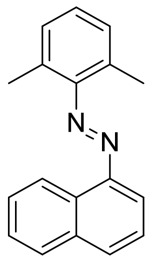

(*E*)-1-(2,6-dimethylphenyl)-2-(naphthalen-1-yl)diazene 3i

Dark red oil, 12 %

^{1}H NMR (500 MHz, Chloroform-*d*) δ 8.83 (d, *J* = 8.4 Hz, 1H), 8.01 (d, *J* = 8.1 Hz, 1H), 7.95 (d, *J* = 8.1 Hz, 1H), 7.80 (d, *J* = 7.5 Hz, 1H), 7.67 – 7.62 (m, 1H), 7.62 – 7.56 (m, 2H), 7.23 – 7.16 (m, 3H), 2.51 (s, 6H).

^{13}C APT NMR (126 MHz, $CDCl_3$) δ 153.1, 147.2, 133.0, 131.3, 129.9, 129.3, 128.9, 127.9, 127.8, 127.5, 127.1, 125.7, 123.7, 122.8, 19.3.

FT-IR (ATR) cm^{-1}: 3049, 2956, 2920, 1594, 1506, 1372, 1199, 1144, 1019, 809, 762, 687, 563.

Elemental anal.: Calc. for $C_{17}H_{14}N_2$: C: 82.90; H: 5.73; N: 11,37.

Found: C: 83.13 ± 0.01; H: 5.67 ± 0.03, N: 11.28 ± 0.01.

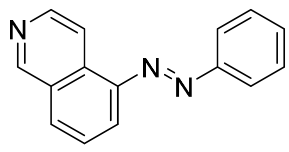

(*E*)-5-(phenyldiazenyl)isoquinoline 3k

Orange solid, MP:119-120 °C

^{1}H NMR (500 MHz, Chloroform-*d*) δ 9.36 (s, 1H), 8.73 – 8.63 (m, 2H), 8.11 (d, J = 8.1 Hz, 1H), 8.10 – 8.00 (m, 3H), 7.71 (t, J = 7.8 Hz, 1H), 7.63 – 7.47 (m, 3H).

^{13}C APT NMR (126 MHz, $CDCl_3$) δ 153.1, 152.7, 146.7, 144.0, 134.0, 131.7, 130.8, 129.5, 129.4, 127.2, 123.4, 116.4, 116.3.

FT-IR (ATR) cm^{-1}: 3047, 1620, 1573, 1380, 1213, 1069, 912, 834, 756, 681, 500.

HRMS: Calculated for $C_{15}H_{12}N_3$ $[M+H]^+$: 234.10257; Found: 234.1029

Elemental anal.: Calc. for $C_{15}H_{11}N_3$: C: 77.23; H: 4.75; N: 18.01

Found: C: 77.18 ± 0.14 ; H: 4.60 ± 0.09; N: 17.15 ± 0.11

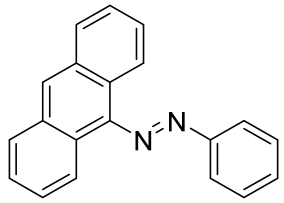

(*E*)-1-(anthracen-9-yl)-2-phenyldiazene 3l

Dark red solid, MP:121-122 °C

^{1}H NMR (500 MHz, Chloroform-*d*) δ 8.68 – 8.61 (m, 2H), 8.50 (s, 1H), 8.19 – 8.13 (m, 2H), 8.07 – 8.02 (m, 2H), 7.67 – 7.61 (m, 2H), 7.60 – 7.50 (m, 5H).

^{13}C APT NMR (126 MHz, $CDCl_3$) δ 153.5, 143.7, 131.9, 131.5, 129.4, 129.4, 128.4, 127.4, 125.9, 124.6, 124.5, 123.0.

FT-IR (ATR) cm^{-1}: 3046, 2916, 1620, 1523, 1440, 1303, 1071, 883, 758, 725, 612.

HRMS: Calculated for $C_{20}H_{15}N_2$ $[M+H]^+$: 283.12298; Found: 283.1233

Elemental anal.: Calc. for $C_{20}H_{14}N_2$: C: 85.08; H: 5.00; N: 9.92

Found: C: 85.81 ± 0.18 ; H: 5.01 ± 0.12; N: 9.41 ± 0.11

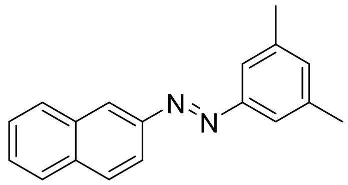

(*E*)-1-(3,5-dimethylphenyl)-2-(naphthalen-2-yl)diazene 3m

Yellow solid, MP: 97-98 °C

^{1}H NMR (500 MHz, Chloroform-*d*) δ 8.45 (d, *J* = 2.0 Hz, 1H), 8.06 (dd, *J* = 8.8, 1.9 Hz, 1H), 8.04 – 7.99 (m, 1H), 7.95 – 7.87 (m, 2H), 7.60 (s, 2H), 7.59 – 7.53 (m, 2H), 7.14 (s, 1H), 2.44 (s, 6H).

^{13}C APT NMR (126 MHz, $CDCl_3$) δ 153.0, 150.4, 138.9, 134.9, 133.6, 132.8, 129.5, 129.2, 128.1, 127.9, 127.6, 126.8, 120.8, 117.2, 21.4.

FT-IR (ATR) cm^{-1}: 3054, 2961, 2913, 2854, 1610, 1375, 1349, 1286, 1121, 955, 864, 748, 684, 477.

HRMS: Calculated for $C_{18}H_{17}N_2$ $[M+H]^+$: 261.13863; Found: 261.13885

Elemental anal.: Calc. for $C_{18}H_{16}N_2$: C: 83.04; H: 6.19; N: 10.76

Found: C: 82.73 ± 0.15 ; H: 6.75 ± 0.02; N: 9.90 ± 0.30

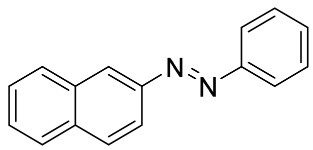

(*E*)-1-(naphthalen-2-yl)-2-phenyldiazene 3n

Yellow solid, MP: 81-82 °C

^{1}H NMR (500 MHz, Chloroform-*d*) δ 8.50 (d, *J* = 2.1 Hz, 1H), 8.10 (dd, *J* = 8.8, 1.9 Hz, 1H), 8.07 – 7.98 (m, 3H), 7.95 – 7.88 (m, 2H), 7.61 – 7.53 (m, 4H), 7.54 – 7.47 (m, 1H).

^{13}C APT NMR (126 MHz, $CDCl_3$) δ 152.9, 150.3, 134.9, 133.6, 131.1, 129.5, 129.3, 128.2, 128.1, 127.7, 126.9, 123.0, 117.2.

FT-IR (ATR) cm^{-1}: 3053, 1625, 1593, 1458, 1428, 1351, 1164, 1019, 819, 745, 687, 472.

HRMS: Calculated for $C_{16}H_{13}N_2$ $[M+H]^+$: 233.10733; Found: 233.10753

Elemental anal.: Calc. for $C_{16}H_{12}N_2$: C: 82.73; H: 5.21; N: 12.06

Found: C: 82.75 ± 0.07; H: 5.00 ± 0.05; N: 11.94 ± 0.04

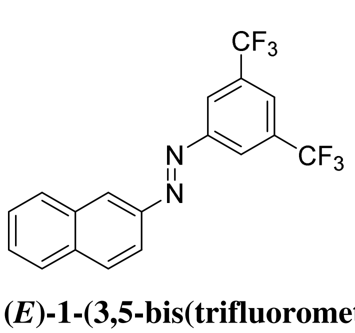

(*E*)-1-(3,5-bis(trifluoromethyl)phenyl)-2-(naphthalen-2-yl)diazene 3o

Yellow solid, MP: 99-100 °C

^{1}H NMR (500 MHz, Chloroform-*d*) δ 8.57 (d, *J* = 1.9 Hz, 1H), 8.42 (d, *J* = 1.6 Hz, 2H), 8.08 – 8.03 (m, 2H), 7.99 (s, 1H), 7.92 (dd, *J* = 10.8, 8.2 Hz, 2H), 7.64 – 7.58 (m, 2H).

^{13}C APT NMR (126 MHz, $CDCl_3$) δ 153.1, 149.8, 135.6, 133.5, 133.8 (q, *J* = 33.8 Hz), 130.6, 129.8, 129.6, 128.6, 128.2, 127.2, 123.8 (m), 123.2 (q, *J* = 272.7 Hz), 123.1 (q, *J* = 3.4 Hz), 116.3.

^{19}F{H} NMR (376 MHz, $CDCl_3$) δ -63.68

FT-IR (ATR) cm^{-1}: 3062, 1625, 1510, 1368, 1275, 1121, 903, 680.

HRMS: Calculated for $C_{18}H_{11}F_6N_2$ $[M+H]^+$: 369.08209; Found: 369.08274

Elemental anal.: Calc. for $C_{18}H_{10}F_6N_2$: C: 58.70; H: 2.74; N: 7.61; F: 30.95

Found: C: 58.60 ± 0.14 ; H: 2.77 ± 0.13; N: 7.58 ± 0.09

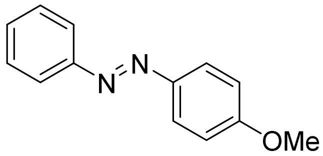

(*E*)-1-(4-methoxyphenyl)-2-phenyldiazene 3q

Orange solid, MP: 53-54 °C

^{1}H NMR (500 MHz, Chloroform-*d*) δ 7.96 – 7.92 (m, 2H), 7.92 – 7.86 (m, 2H), 7.53 – 7.48 (m, 2H), 7.47 – 7.42 (m, 1H), 7.04 – 7.00 (m, 2H), 3.90 (s, 3H).

^{13}C APT NMR (126 MHz, $CDCl_3$) δ 162.2, 152.8, 147.1, 130.5, 129.2, 124.9, 122.7, 114.3, 55.7.

FT-IR (ATR) cm^{-1}: 3085, 3039, 2960, 2839, 1600, 1495, 1439, 1246, 1028, 836, 759, 684, 553.

HRMS: Calculated for $C_{13}H_{13}N_2O$ $[M+H]^+$: 213.10224; Found: 213.1035

Elemental anal.: Calc. for $C_{13}H_{12}N_2O$: C: 73.56; H: 5.70; N: 13.20; O: 7.54

Found: C: 73.87 ± 0.13 ; H: 5.84 ± 0.06; N: 12.89 ± 0.09

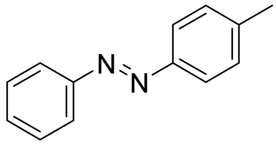

(*E*)-1-phenyl-2-(p-tolyl)diazene 3r

Yellow solid, MP: 69-70 °C

^{1}H NMR (500 MHz, Chloroform-*d*) δ 7.93 – 7.89 (m, 2H), 7.86 – 7.82 (m, 2H), 7.54 – 7.49 (m, 2H), 7.48 – 7.44 (m, 1H), 7.35 – 7.30 (m, 2H), 2.44 (s, 3H).

^{13}C APT NMR (126 MHz, $CDCl_3$) δ 152.8, 150.9, 141.7, 130.8, 129.9, 129.2, 123.0, 122.8, 21.6.

FT-IR (ATR) cm^{-1}: 3036, 2921, 1601, 1485, 1442, 1301, 1210, 1149, 1017, 821, 763, 681, 490.

HRMS: Calculated for $C_{13}H_{13}N_2$ $[M+H]^+$: 197.10733; Found: 197.1075

Elemental anal.: Calc. for $C_{13}H_{12}N_2$: C: 79.56; H: 6.16; N: 14.27

Found: C: 79.94 ± 0.12 ; H: 6.35 ± 0.15; N: 14.25 ± 0.09

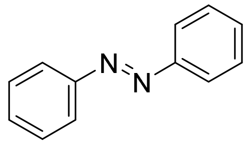

(*E*)-1,2-diphenyldiazene 3s

Orange solid, MP: 68-69 °C

^{1}H NMR (500 MHz, Chloroform-*d*) δ 7.97 – 7.91 (m, 4H), 7.56 – 7.51 (m, 4H), 7.51 – 7.45 (m, 2H).

^{13}C APT NMR (126 MHz, $CDCl_3$) δ 152.8, 131.1, 129.2, 122.9.

FT-IR (ATR) cm^{-1}: 3061, 1581, 1482, 1452, 1298, 1220, 1070, 924, 773, 685, 519.

HRMS: Calculated for $C_{12}H_{11}N_2$ $[M+H]^+$: 183.09167; Found: 183.0919

Elemental anal.: Calc. for $C_{12}H_{10}N_2$: C: 79.10; H: 5.53; N: 15.37

Found: C: 79.34 ± 0.16 ; H: 5.41 ± 0.08; N: 15.15 ± 0.05

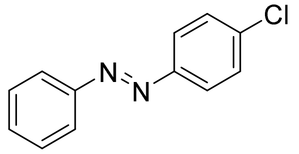

(*E*)-1-(4-chlorophenyl)-2-phenyldiazene 3t

Orange solid, MP: 87-88 °C

^{1}H NMR (500 MHz, Chloroform-*d*) δ 7.96 – 7.90 (m, 2H), 7.90 – 7.83 (m, 2H), 7.57 – 7.46 (m, 5H).

^{13}C APT NMR (126 MHz, $CDCl_3$) δ 152.6, 151.1, 137.0, 131.4, 129.5, 129.3, 124.3, 123.1.

FT-IR (ATR) cm^{-1}: 3058, 1573, 1479, 1296, 1210, 1147, 1084, 1004, 839, 762, 680, 523.

HRMS: Calculated for $C_{12}H_{10}ClN_2$ $[M+H]^+$: 217.05270; Found: 217.0529

Elemental anal.: Calc. for $C_{12}H_9ClN_2$: C: 66.52; H: 4.19; N: 12.93; Cl: 16.36

Found: C: 67.08 ± 0.24 ; H: 4.00 ± 0.02; N: 12.55 ± 0.05; Cl: 15.02 ± 0.16

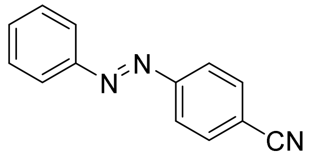

(*E*)-4-(phenyldiazenyl)benzonitrile 3u

Orange solid, MP: 121-122 °C

^{1}H NMR (500 MHz, Chloroform-*d*) δ 8.02 – 7.97 (m, 2H), 7.97 – 7.92 (m, 2H), 7.84 – 7.80 (m, 2H), 7.58 – 7.51 (m, 3H).

^{13}C APT NMR (126 MHz, $CDCl_3$) δ 154.6, 152.5, 133.4, 132.4, 129.4, 123.5, 123.5, 118.6, 114.1.

FT-IR (ATR) cm^{-1}: 3052, 2224, 1699, 1483, 1440, 1288, 1141, 1104, 923, 853, 771, 680, 561.

HRMS: Calculated for $C_{13}H_{10}N_3$ $[M+H]^+$: 208.08692; Found: 208.0870

Elemental anal.: Calc. for $C_{13}H_9N_3$: C: 75.35; H: 4.38; N: 20.28

Found: C: 75.51 ± 0.10 ; H: 4.15 ± 0.01; N: 19.59 ± 0.09

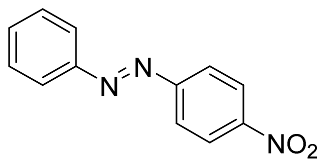

(*E*)-1-(4-nitrophenyl)-2-phenyldiazene 3v

Dark orange solid, MP: 135-136 °C

^{1}H NMR (500 MHz, Chloroform-*d*) δ 8.42 – 8.36 (m, 2H), 8.06 – 8.01 (m, 2H), 8.01 – 7.95 (m, 2H), 7.59 – 7.53 (m, 3H).

^{13}C APT NMR (126 MHz, $CDCl_3$) δ 155.8, 152.5, 148.8, 132.6, 129.5, 124.9, 123.6, 123.6.

FT-IR (ATR) cm^{-1}: 3090, 1587, 1521, 1442, 1340, 1215, 1104, 998, 857, 775, 686.

HRMS: Calculated for $C_{12}H_{10}N_3O_2$ $[M+H]^+$: 228.07675; Found: 228.0772

Elemental anal.: Calc. for $C_{12}H_9N_3O_2$: C: 63.43; H: 3.99; N: 18.49; O: 14.08

Found: C: 63.70 ± 0.09 ; H: 4.15 ± 0.03; N: 18.34 ± 0.01

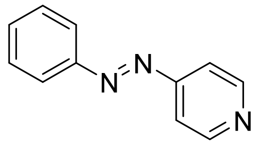

(E)-4-(phenyldiazenyl)pyridine 3w

Orange solid, MP: 96-97 °C

^{1}H NMR (500 MHz, Chloroform-*d*) δ 8.85 – 8.79 (m, 2H), 8.00 – 7.93 (m, 2H), 7.74 – 7.68 (m, 2H), 7.59 – 7.53 (m, 3H).

^{13}C APT NMR (126 MHz, $CDCl_3$) δ 157.3, 152.5, 151.5, 132.5, 129.4, 123.5, 116.4.

FT-IR (ATR) cm^{-1}: 3030, 1583, 1474, 1409, 1224, 1149, 930, 835, 765, 684, 562.

HRMS: Calculated for $C_{11}H_{10}N_3$ $[M+H]^+$: 184.08692; Found: 184.0876

Elemental anal.: Calc. for $C_{11}H_9N_3$: C: 72.11; H: 4.95; N: 22.94

Found: C: 72.34 ± 0.08 ; H: 5.17 ± 0.17; N: 22.41 ± 0.09

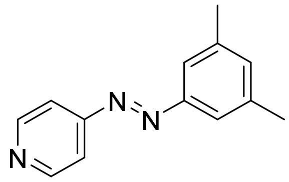

(*E*)-4-((3,5-dimethylphenyl)diazenyl)pyridine 3x

Orange solid, MP: 52-53 °C

^{1}H NMR (500 MHz, Chloroform-*d*) δ 8.82 – 8.78 (m, 2H), 7.70 – 7.68 (m, 2H), 7.58 (s, 2H), 7.19 (s, 1H), 2.43 (s, 6H).

^{13}C APT NMR (126 MHz, $CDCl_3$) δ 157.4, 152.7, 151.4, 139.1, 134.3, 121.3, 116.4, 21.4.

FT-IR (ATR) cm^{-1}: 3031, 2916, 2855, 1585, 1447, 1408, 1374, 1287, 1119, 855, 823, 680, 511.

HRMS: Calculated for $C_{13}H_{14}N_3$ $[M+H]^+$: 212.11822; Found: 212.1188

Elemental anal.: Calc. for $C_{13}H_{13}N_3$: C: 73.91; H: 6.20; N: 19.89

Found: C: 72.21 ± 0.03 ; H: 6.23 ± 0.02; N: 17.93 ± 0.27

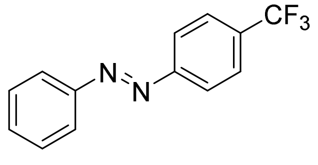

(*E*)-1-phenyl-2-(4-(trifluoromethyl)phenyl)diazene 3y

Yellow solid, MP: 95-96 °C

^{1}H NMR (500 MHz, Chloroform-*d*) δ 8.03 – 7.98 (m, 2H), 7.98 – 7.93 (m, 2H), 7.82 – 7.75 (m, 2H), 7.58 – 7.50 (m, 3H).

^{13}C APT NMR (126 MHz, $CDCl_3$) δ 154.5, 152.5, 132.3 (q, *J*=32.6 Hz), 131.9, 129.4, 126.4 (q, *J*=3,6 Hz), 124.1 (q, *J*=272,6 Hz), 123.3, 123.2, 120.5.

$^{19}F\{H\}$ NMR (376 MHz, $CDCl_3$) δ -63.36

FT-IR (ATR) cm^{-1}: 3067, 2920, 1694, 1609, 1486, 1411, 1321, 1220, 1150, 1101, 1063, 1008, 848, 768, 677, 599.

HRMS: Calculated for $C_{13}H_{10}F_3N_2$ $[M+H]^+$: 251.07906; Found: 251.0796

Elemental anal.: Calc. for $C_{13}H_9F_3N_2$: C: 62.40; H: 3.63; N: 11.20; F: 22.78

Found: C: 62.96 ± 0.05 ; H: 4.12 ± 0.07; N: 11.28 ± 0.01

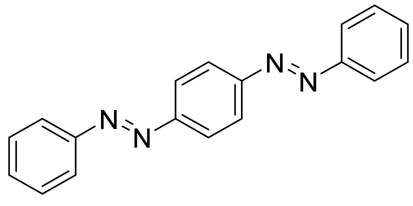

1,4-bis((E)-phenyldiazenyl)benzene 3z

Orange solid, MP: 168-169 °C

^{1}H NMR (500 MHz, Chloroform-*d*) δ 8.08 (s, 4H), 7.99 – 7.95 (m, 4H), 7.58 – 7.49 (m, 6H).

^{13}C APT NMR (126 MHz, $CDCl_3$) δ 153.8, 152.8, 131.6, 129.3, 123.9, 123.2.

FT-IR (ATR) cm^{-1}: 3037, 1586, 1483, 1440, 1304, 1209, 859, 762, 682, 506.

HRMS: Calculated for $C_{18}H_{15}N_4$ $[M+H]^+$: 287.12912; Found: 287.1290

Elemental anal.: Calc. for $C_{18}H_{14}N_4$: C: 75.50; H: 4.93; N: 19.57

Found: C: 75.46 ± 0.04 ; H: 4.88 ± 0.04; N: 19.56 ± 0.29

Single crystal X-ray difraction data

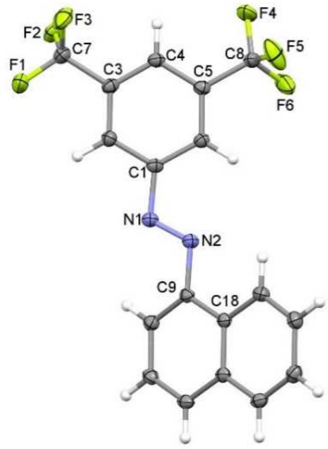

Figure S4. X-ray structure of **3h**, selected intermolecular distances and angles [Å, °]: N1—N2 1.2542(16), N1—C1 1.4287(16), N2—C9 1.4236(15), N2—N1—C1 113.49(10), N1—N2—C9 114.00(10).

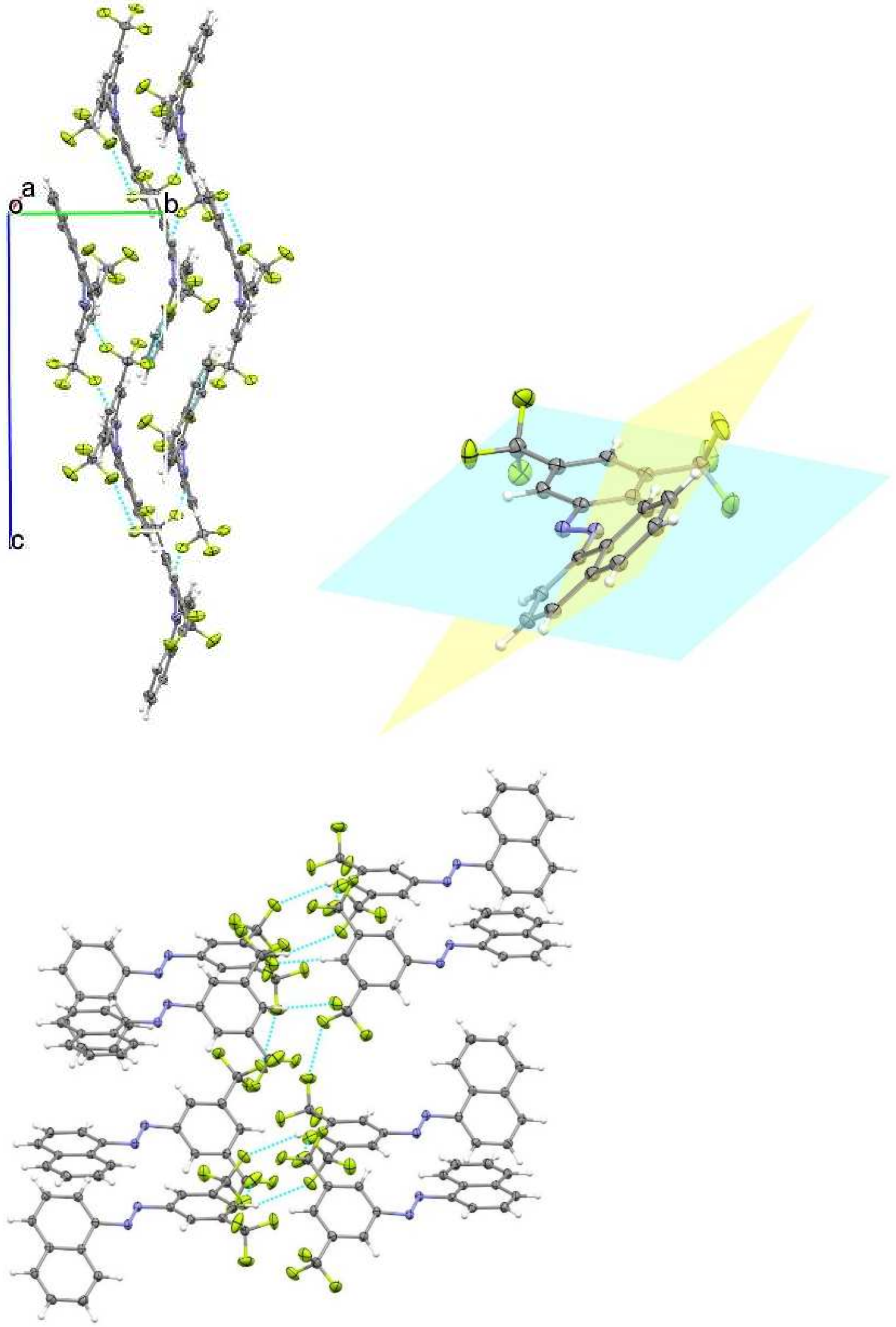

Figure S5. Crystal packing of **3h**, view of unit cell along *a*-axis, visualization of interplanar angle between phenyl and naphthyl moieties (40.69(12)°) and supramolecular architecture by weak C-H…F and F…F contacts.

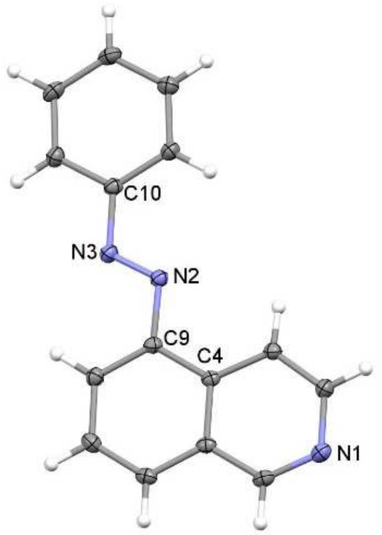

Figure S6. X-ray structure of **3k**, selected intermolecular distances and angles [Å, °]: N1—C2 1.3123(13), N1—C1 1.3693(12), N2—N3 1.2556(11), N2—C9 1.4266(11), N3—C10 1.4249(12), C2—N1—C1 116.73(8), N3—N2—C9 113.61(8), N2—N3—C10 114.84(8).

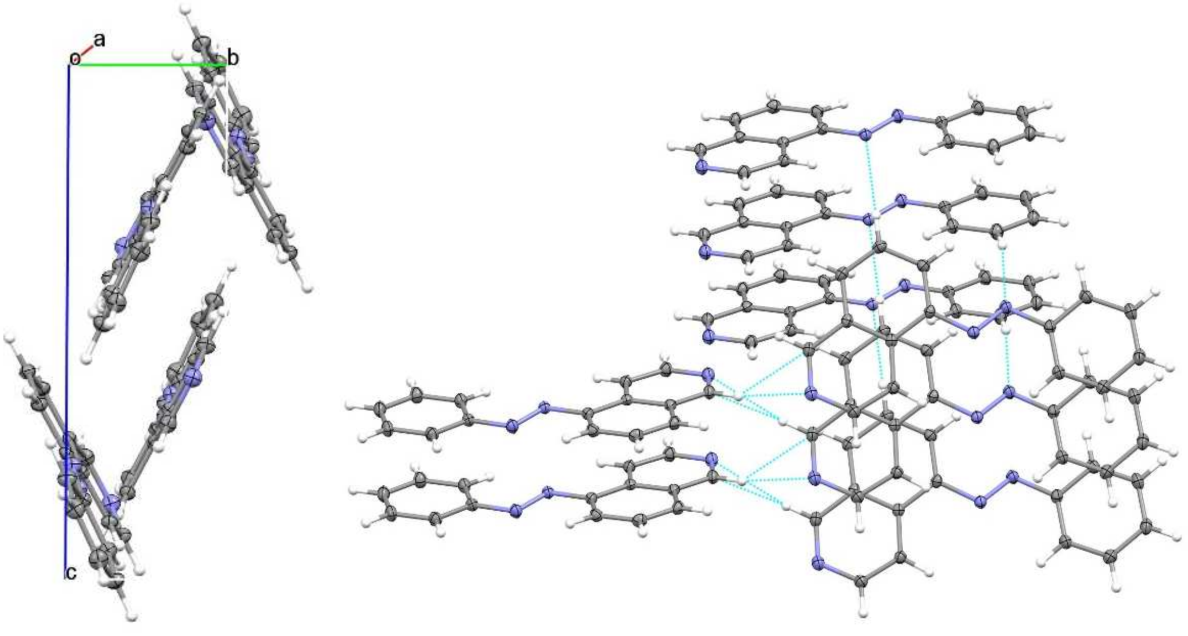

Figure S7. Crystal packing of **3k**, view of unit cell along *a*-axis, visualization of interplanar angle between phenyl and naphthyl moieties and supramolecular architecture by weak C-H…N (azenyl or quinolyl) contacts.

Table S2: Experimental details for **3h**

Crystal data	
Chemical formula	$C_{18}H_{10}F_6N_2$
M_r	368.28
Crystal system, space group	Monoclinic, $P2_1/c$
Temperature (K)	150
a, b, c (Å)	13.1240 (3), 7.3548 (2), 15.7871 (4)
β (°)	91.132 (1)
V (Å^3)	1523.54 (7)
Z	4
Radiation type	MoKα
μ (mm^{-1})	0.15
Crystal size (mm)	0.59 × 0.33 × 0.18
**Data collection**	
Diffractometer	Bruker D8 - Venture
Absorption correction	Multi-scan<br>*SADABS2016*/2 - Bruker AXS area detector scaling and absorption correction Reference: Krause, L., Herbst-Irmer, R., Sheldrick G.M. & Stalke D., J. Appl. Cryst. 48 (2015) 3-10.
T_{min}, T_{max}	0.724, 0.745
No. of measured, independent and observed [$I > 2\sigma(I)$] reflections	25070, 2898, 2718
R_{int}	0.034
$(\sin \theta/\lambda)_{max}$ (Å^{-1})	0.617
**Refinement**	
$R[F^2 > 2\sigma(F^2)]$, $wR(F^2)$, S	0.038, 0.103, 1.03
No. of reflections	2898
No. of parameters	236
No. of restraints	210
H-atom treatment	H-atom parameters constrained
$\Delta\rho_{max}$, $\Delta\rho_{min}$ (e Å^{-3})	0.27, −0.29

Computer programs: Bruker Instrument Service vV6.2.3, *APEX3* v2016.5-0 (Bruker AXS), *SAINT* V8.37A (Bruker AXS Inc., 2015), XT, VERSION 2014/5, *SHELXL2019*/1 (Sheldrick, 2019), *PLATON* (Spek, 2009).

Table S3: Experimental details for **3k**

Crystal data	
Chemical formula	$C_{15}H_{11}N_3$
M_r	233.27
Crystal system, space group	Monoclinic, $P2_1/c$
Temperature (K)	150
a, b, c (Å)	23.3180 (5), 3.8888 (1), 12.9751 (3)
β (°)	104.985 (1)
V (Å^3)	1136.56 (5)
Z	4
Radiation type	Mo$K\alpha$
μ (mm^{-1})	0.08
Crystal size (mm)	0.45 × 0.13 × 0.05
Data collection	
Diffractometer	Bruker D8 - Venture
Absorption correction	Multi-scan<br>*SADABS2016*/2 - Bruker AXS area detector scaling and absorption correction Reference: Krause, L., Herbst-Irmer, R., Sheldrick G.M. & Stalke D., J. Appl. Cryst. 48 (2015) 3-10.
T_{min}, T_{max}	0.722, 0.746
No. of measured, independent and observed [$I > 2\sigma(I)$] reflections	25211, 2791, 2476
R_{int}	0.030
(sin θ/λ)$_{max}$ (Å^{-1})	0.668
Refinement	
$R[F^2 > 2\sigma(F^2)]$, $wR(F^2)$, S	0.043, 0.126, 1.06
No. of reflections	2791
No. of parameters	163
No. of restraints	138
H-atom treatment	H-atom parameters constrained
$\Delta\rho_{max}$, $\Delta\rho_{min}$ (e Å^{-3})	0.28, −0.18

Computer programs: Bruker Instrument Service vV6.2.3, *APEX4* v2022.10-0 (Bruker AXS), *SAINT* V8.37A (Bruker AXS Inc., 2015), XT, VERSION 2014/5, *SHELXL2019*/1 (Sheldrick, 2019), *PLATON* (Spek, 2009).

The X-ray data for colorless crystals of **3h** and **3k** were obtained at 150K using Oxford Cryostream low-temperature device with a Bruker D8-Venture diffractometer equipped with Mo (Mo/K_α radiation; λ = 0.71073 Å) microfocus X-ray (IμS) source, Photon CMOS detector and Oxford Cryosystems cooling device was used for data collection. Obtained data were treated by XT-version 2014/5 and SHELXL-2017/1 software implemented in APEX4 v2019.1-0 (Bruker AXS) system.[13] $R_{int} = \sum|F_o^2 - F_{o,mean}^2|/\sum F_o^2$, $S = [\sum(w(F_o^2 - F_c^2)^2)/(N_{diffrs} - N_{params})]^{½}$ for all data, $R(F) = \sum||F_o| - |F_c||/\sum|F_o|$ for observed data, $wR(F^2) = [\sum(w(F_o^2 - F_c^2)^2)/(\sum w(F_o^2)^2)]^{½}$ for all data. Crystallographic data for all structural analysis have been deposited with the Cambridge Crystallographic Data Centre, CCDC nos. 2349440-2349441. Copies of this information may be obtained free of charge from The Director, CCDC, 12 Union Road, Cambridge CB2 1EY, UK (fax: +44-1223-336033; e-mail: deposit@ccdc.cam.ac.uk or www: http://www.ccdc.cam.ac.uk).

The frames were integrated with the Bruker SAINT software package using a narrow-frame algorithm. Data were corrected for absorption effects using the Multi-Scan method (SADABS). The structures were solved and refined using the Bruker SHELXTL Software Package.

Hydrogen atoms were mostly localized on a difference Fourier map, however to ensure uniformity of treatment of crystal, most of the hydrogen atoms were recalculated into idealized positions (riding model) and assigned temperature factors $H_{iso}(H)$ = 1.2 Ueq (pivot atom). H in aromatic rings were placed with C-H distances of 0.93 Å.

Spectra

^{1}H NMR (500 MHz, Chloroform-*d*)

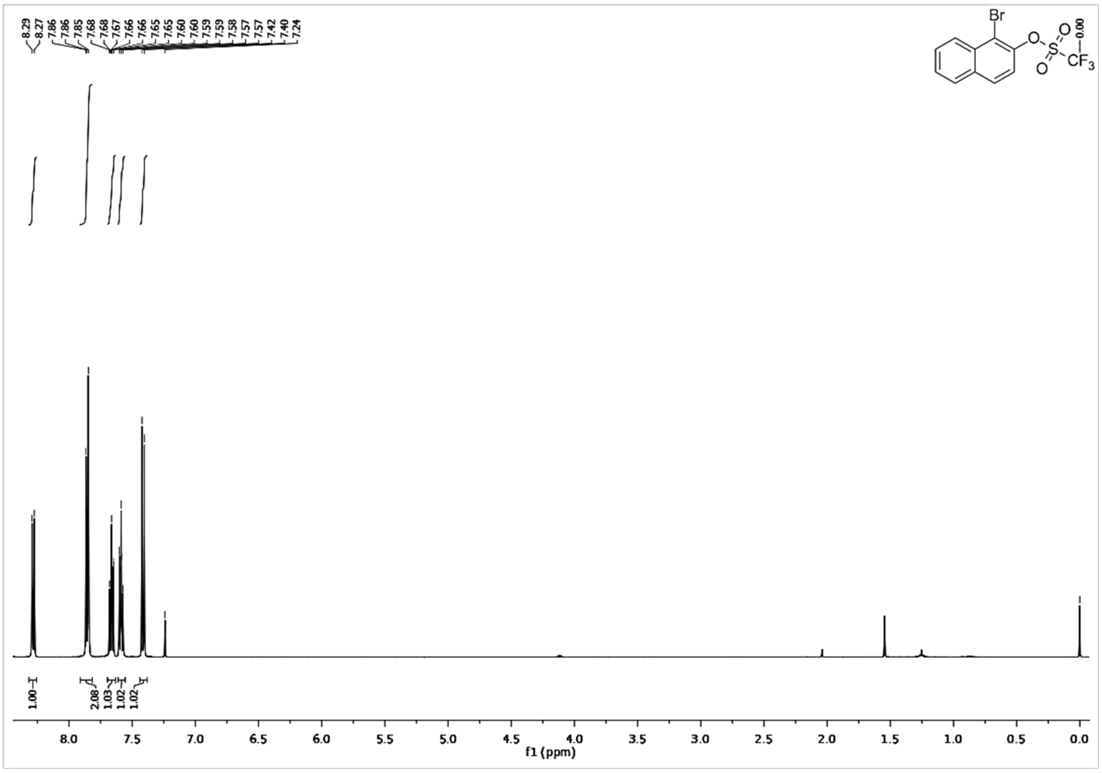

^{13}C APT NMR (126 MHz, $CDCl_3$)

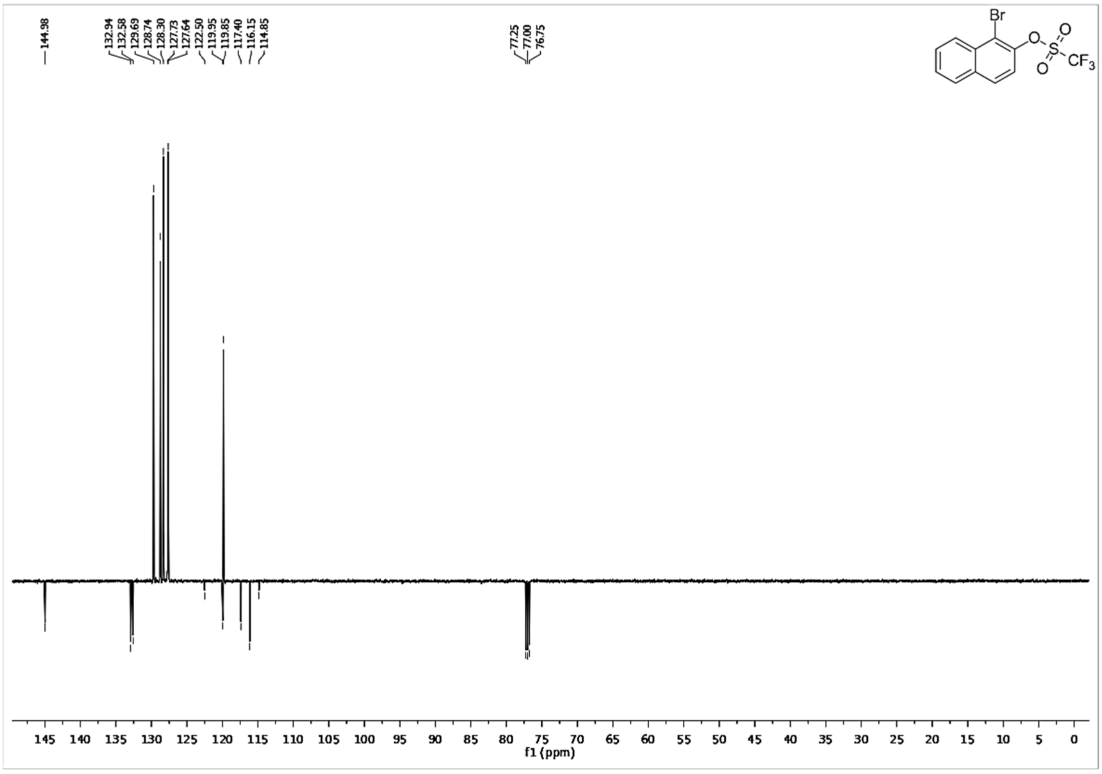

^{1}H NMR (500 MHz, DMSO-d6)

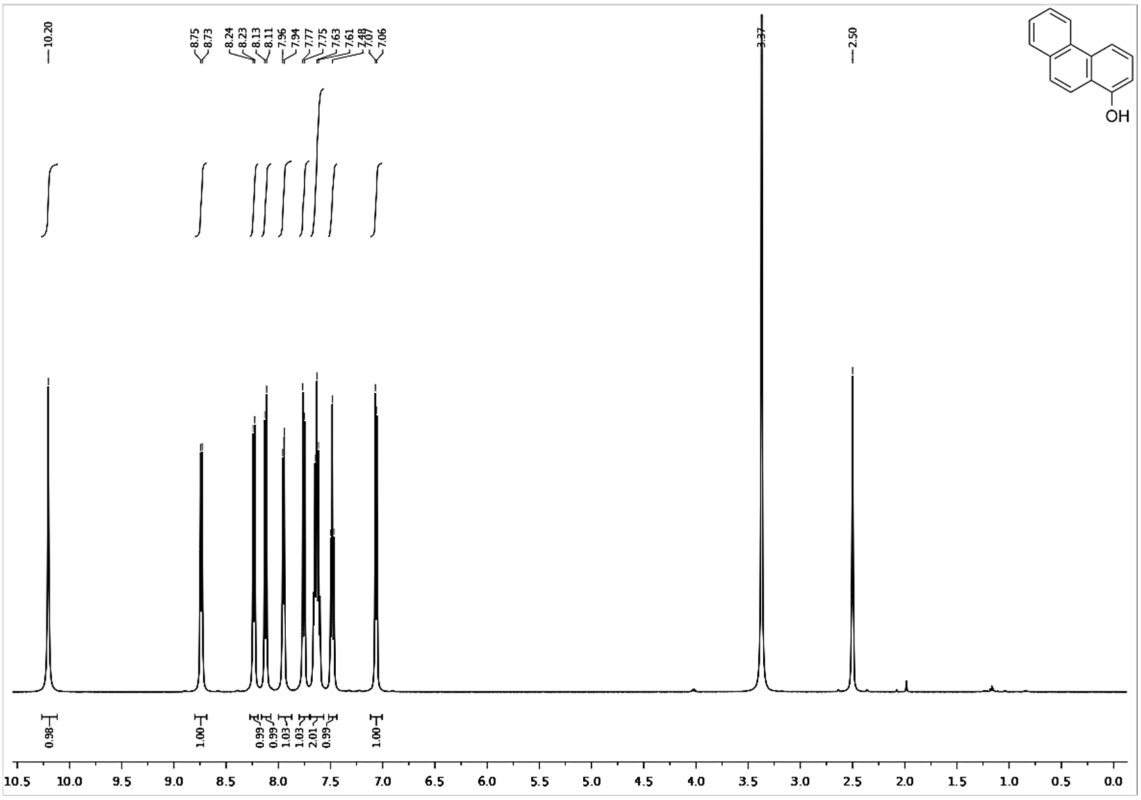

^{13}C APT NMR (126 MHz, DMSO-d6)

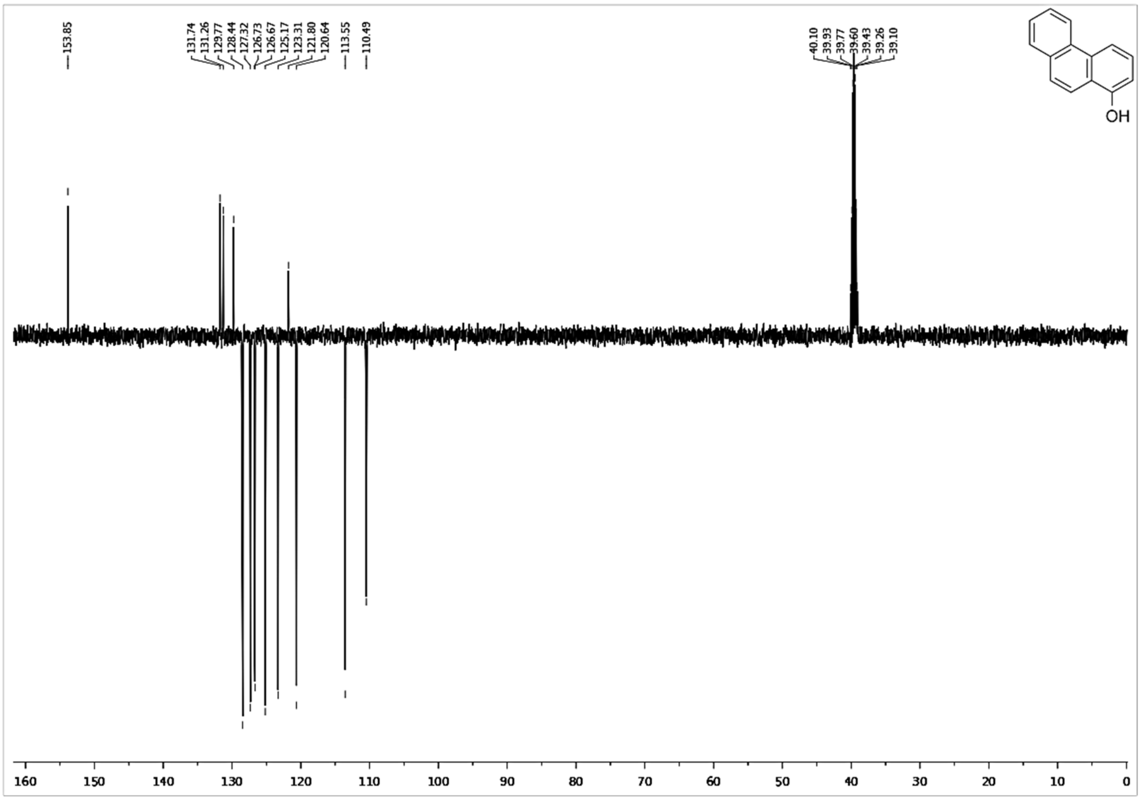

^{1}H NMR (500 MHz, Chloroform-*d*)

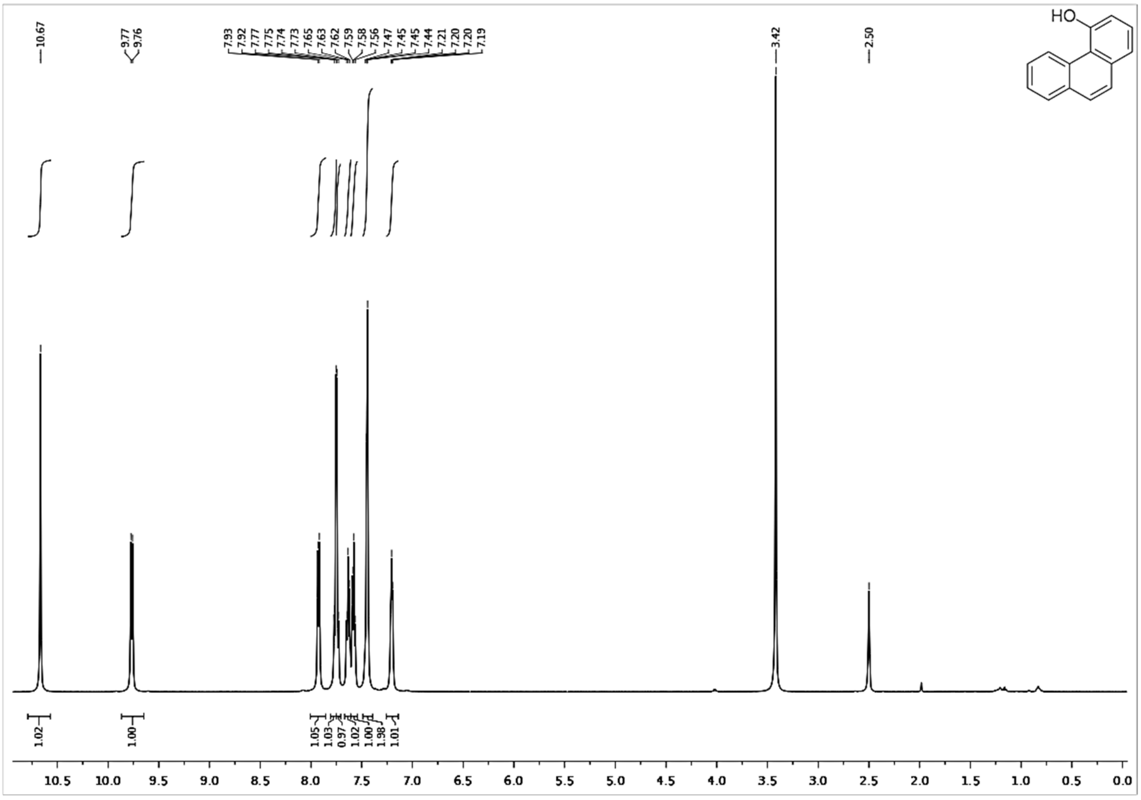

^{13}C APT NMR (126 MHz, DMSO-d6)

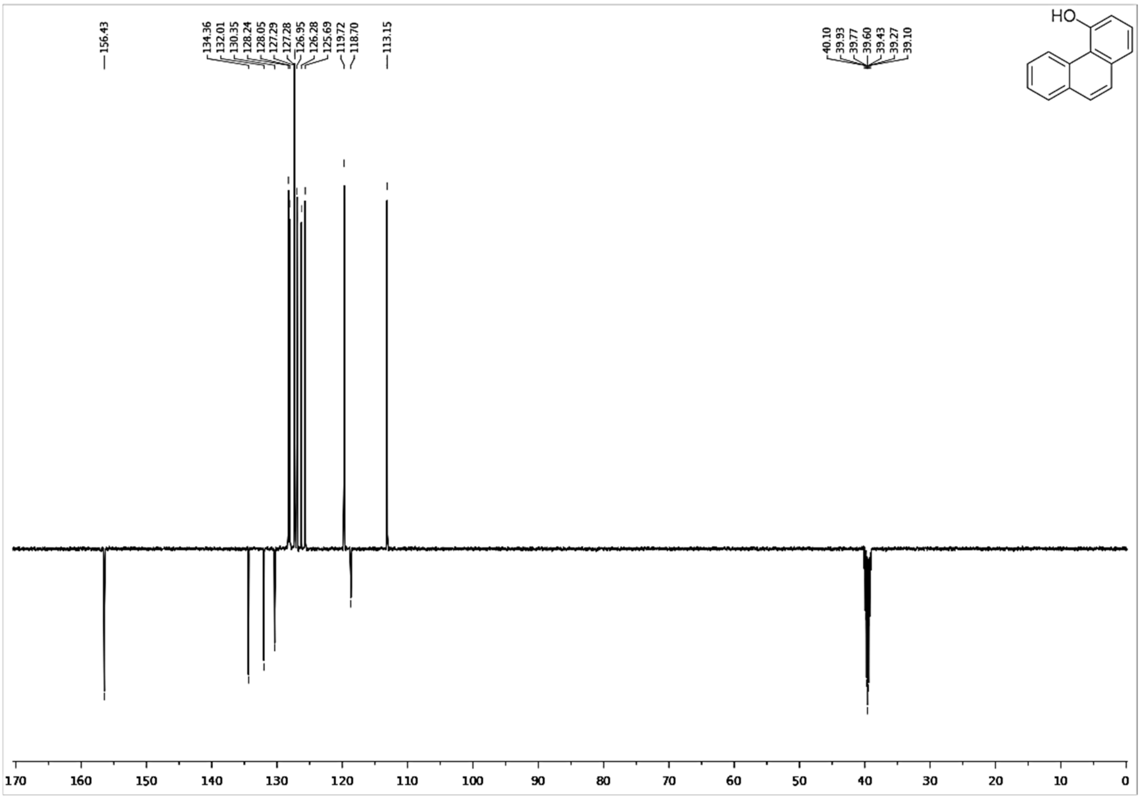

^{1}H NMR (500 MHz, Chloroform-*d*)

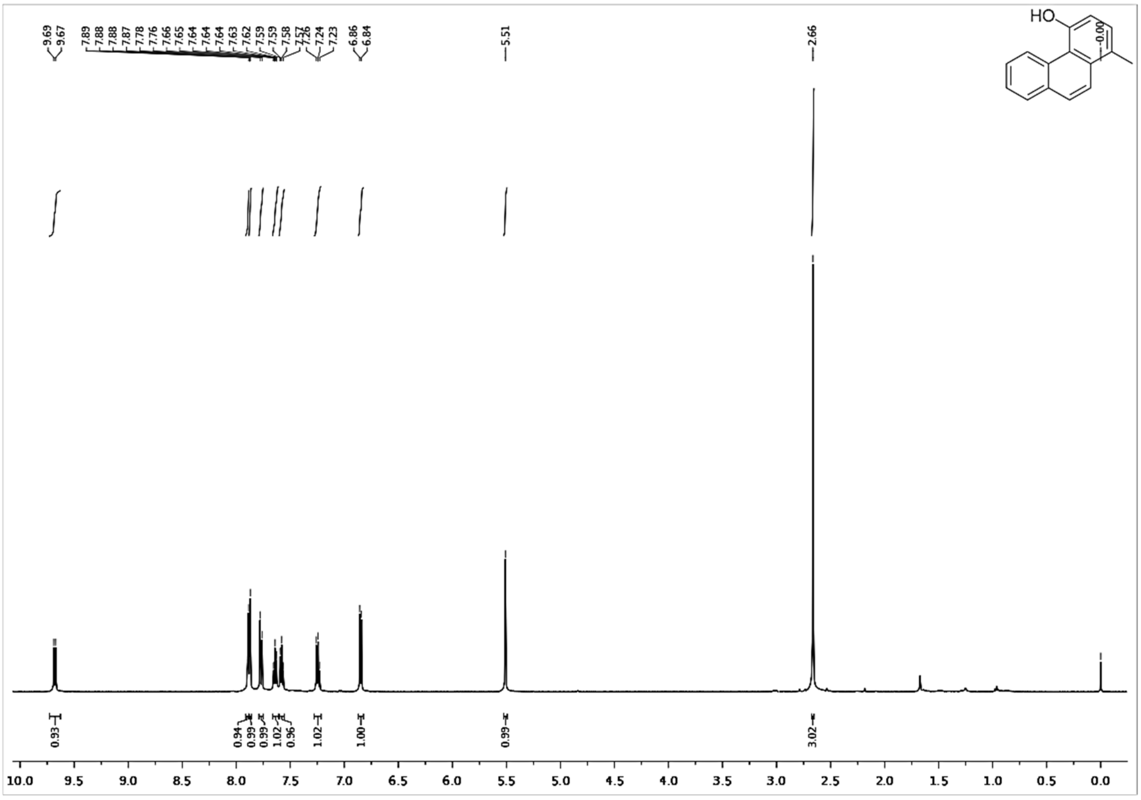

^{13}C APT NMR (126 MHz, Chloroform-*d*)

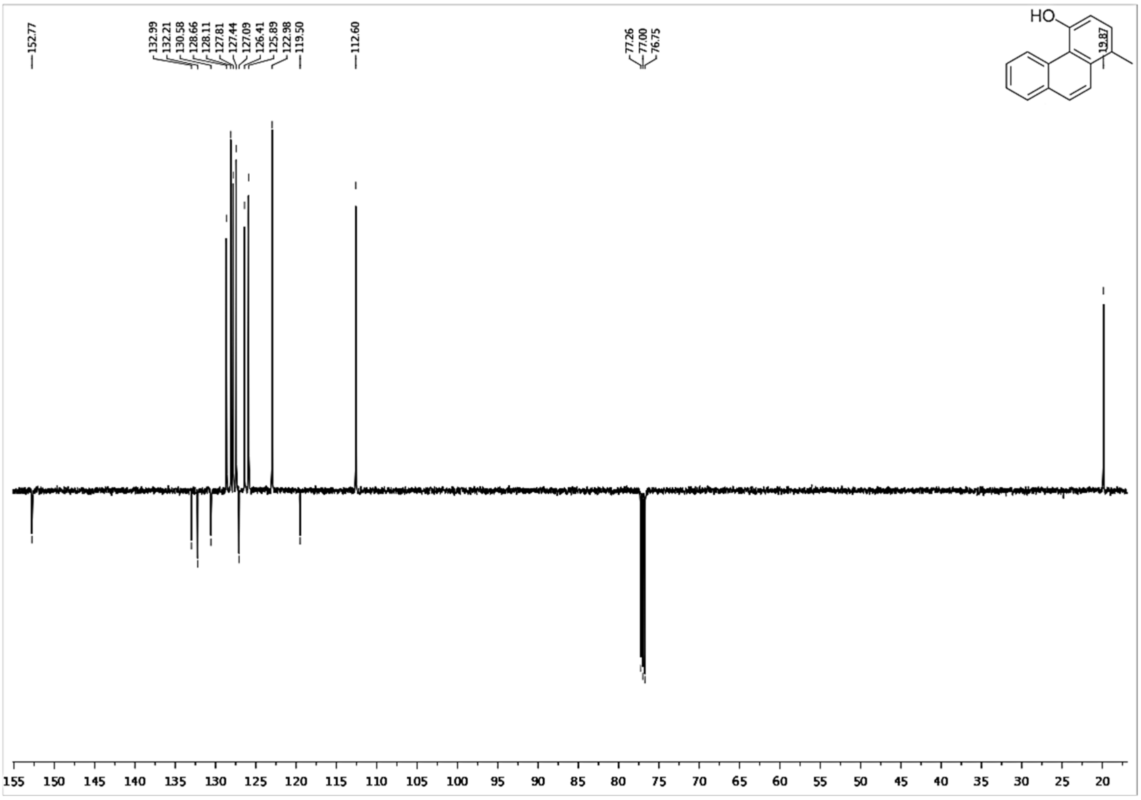

^{1}H NMR (500 MHz, Chloroform-*d*) of **p1e**

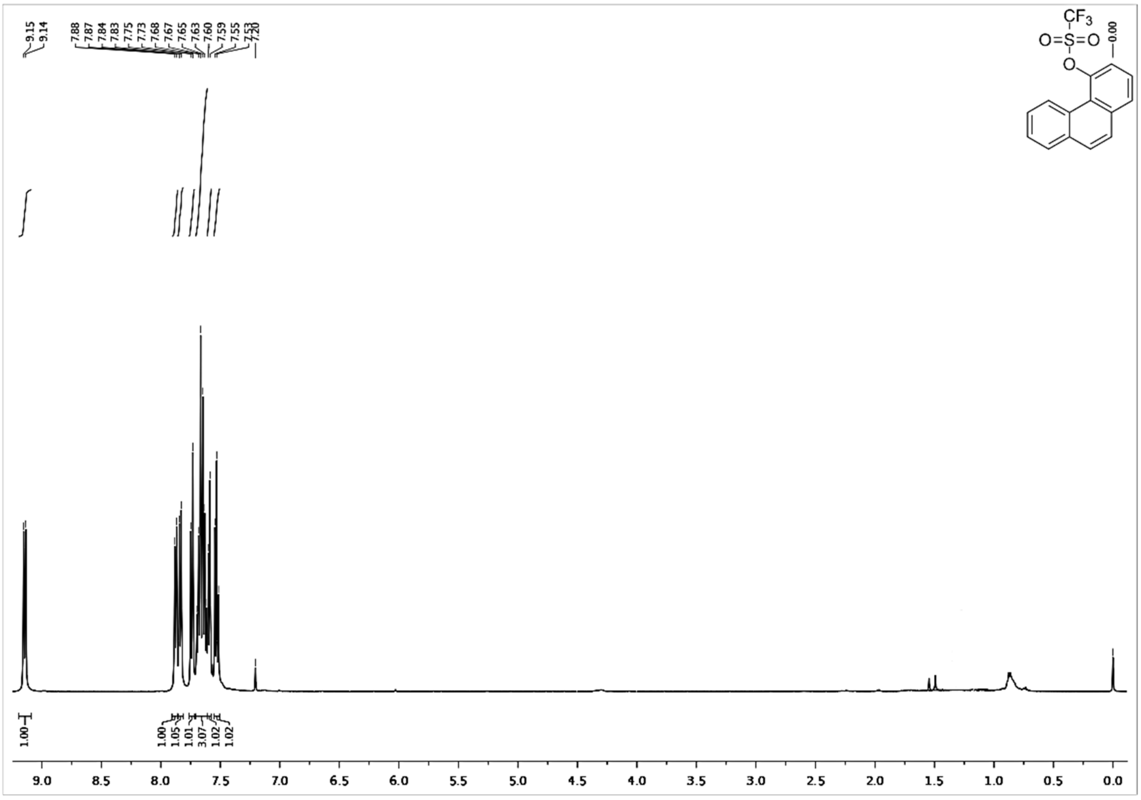

^{13}C APT NMR (126 MHz, Chloroform-*d*) of **p1e**

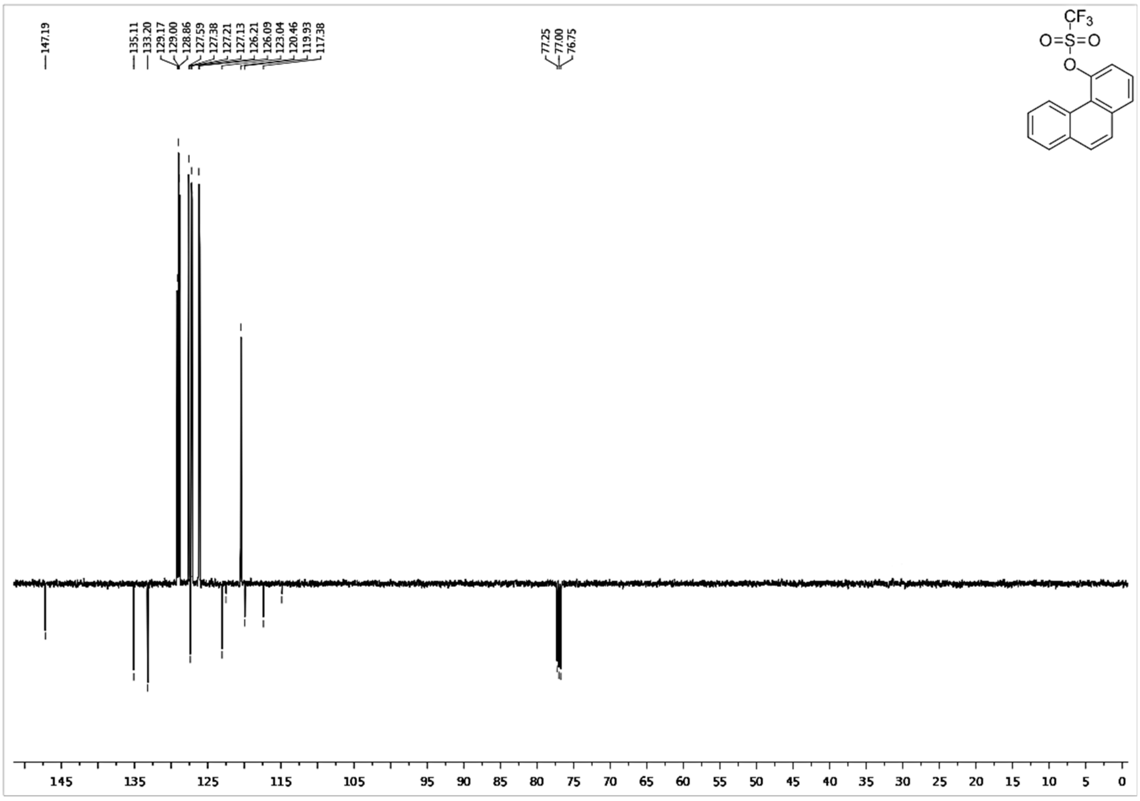

^{1}H NMR (500 MHz, Chloroform-*d*) of **p1g**

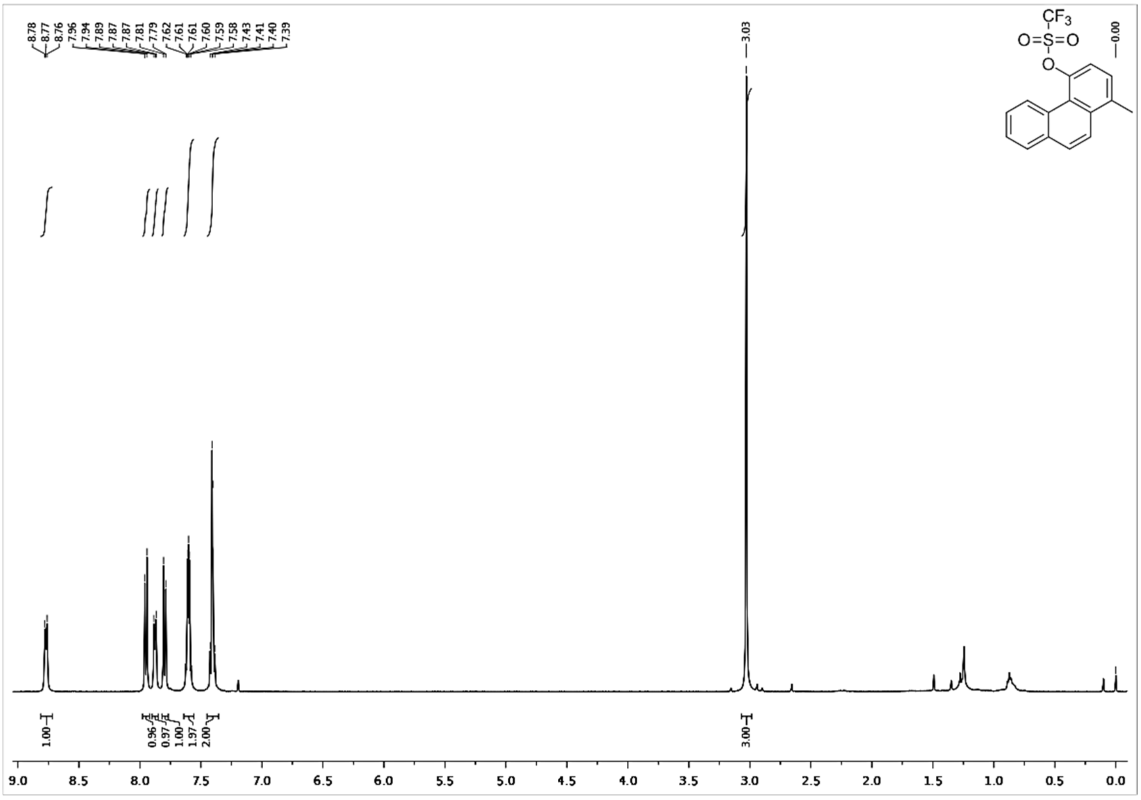

^{13}C APT NMR (126 MHz, Chloroform-*d*) of **p1g**

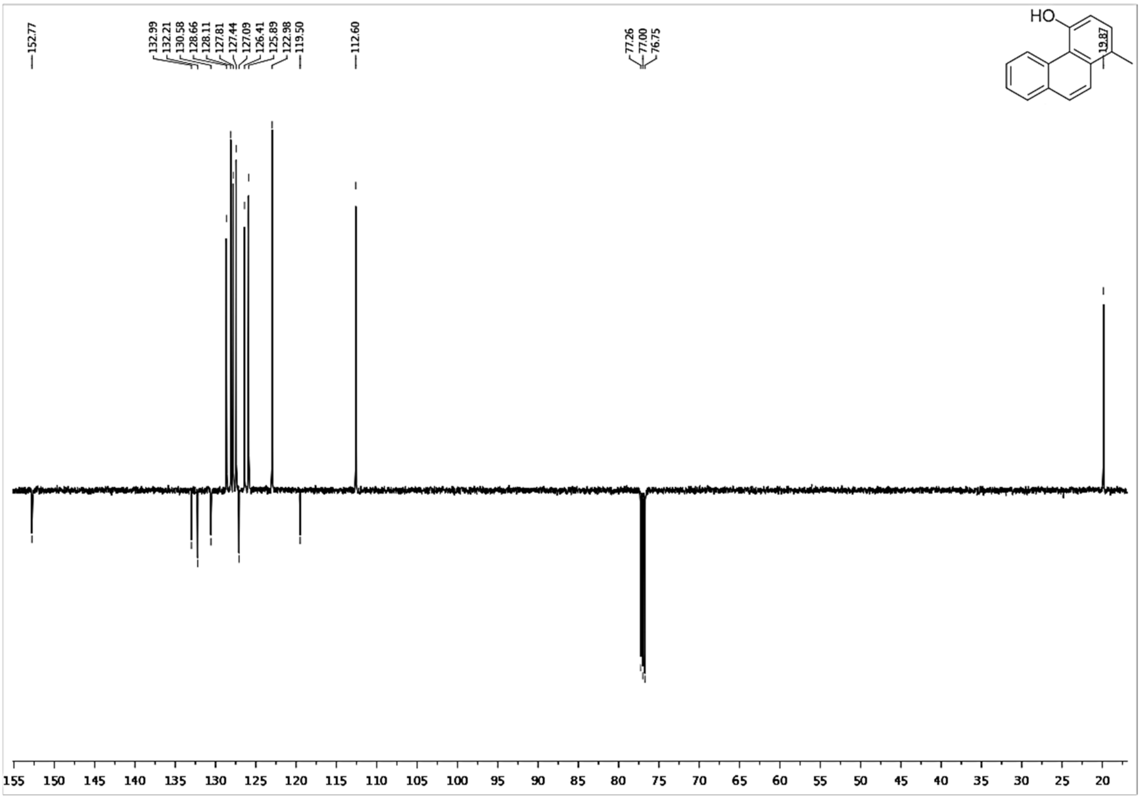

^{1}H NMR (500 MHz, Chloroform-*d*) of **p3e**

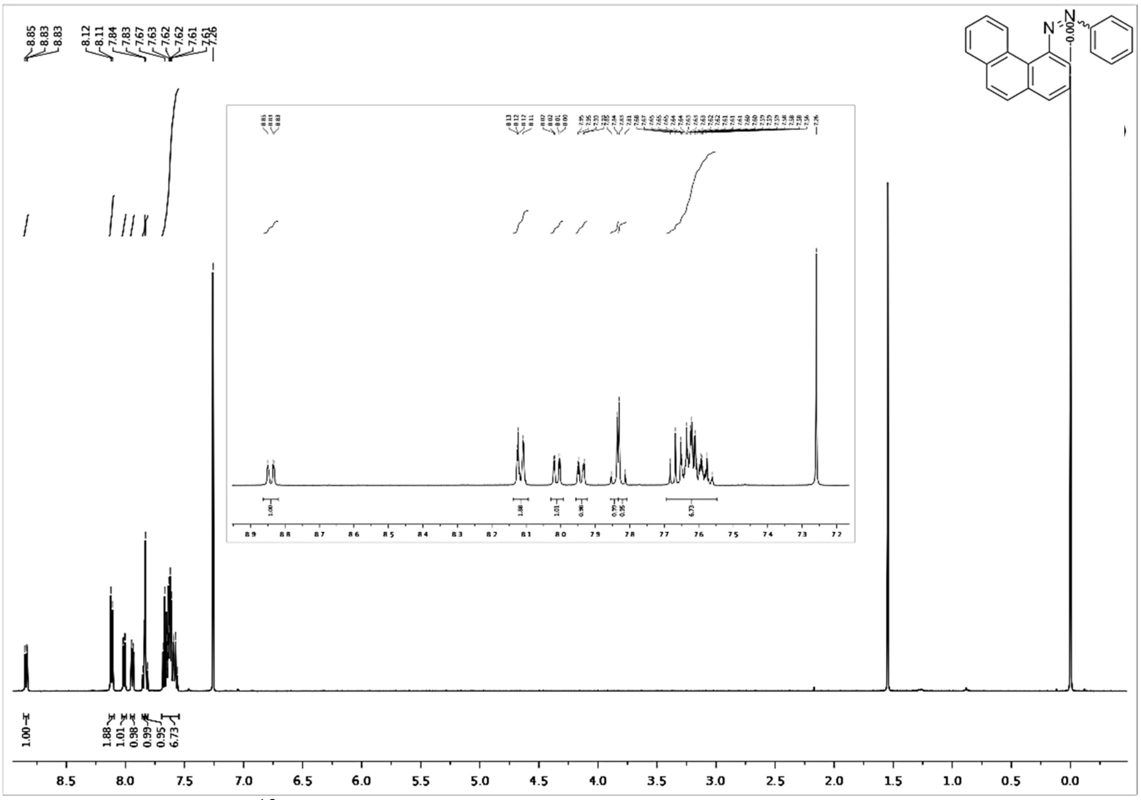

^{13}C APT NMR (126 MHz, Chloroform-*d*) of **p3e**

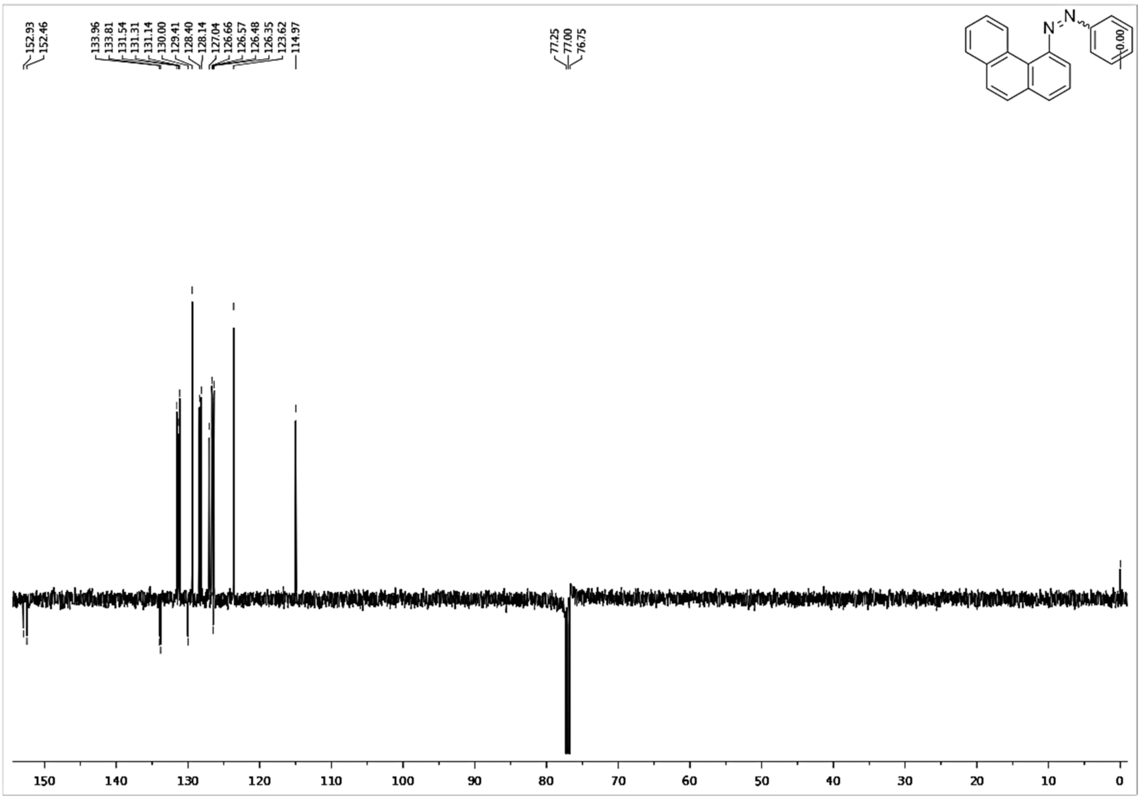

^{1}H NMR (500 MHz, Chloroform-*d*) of **p3f**

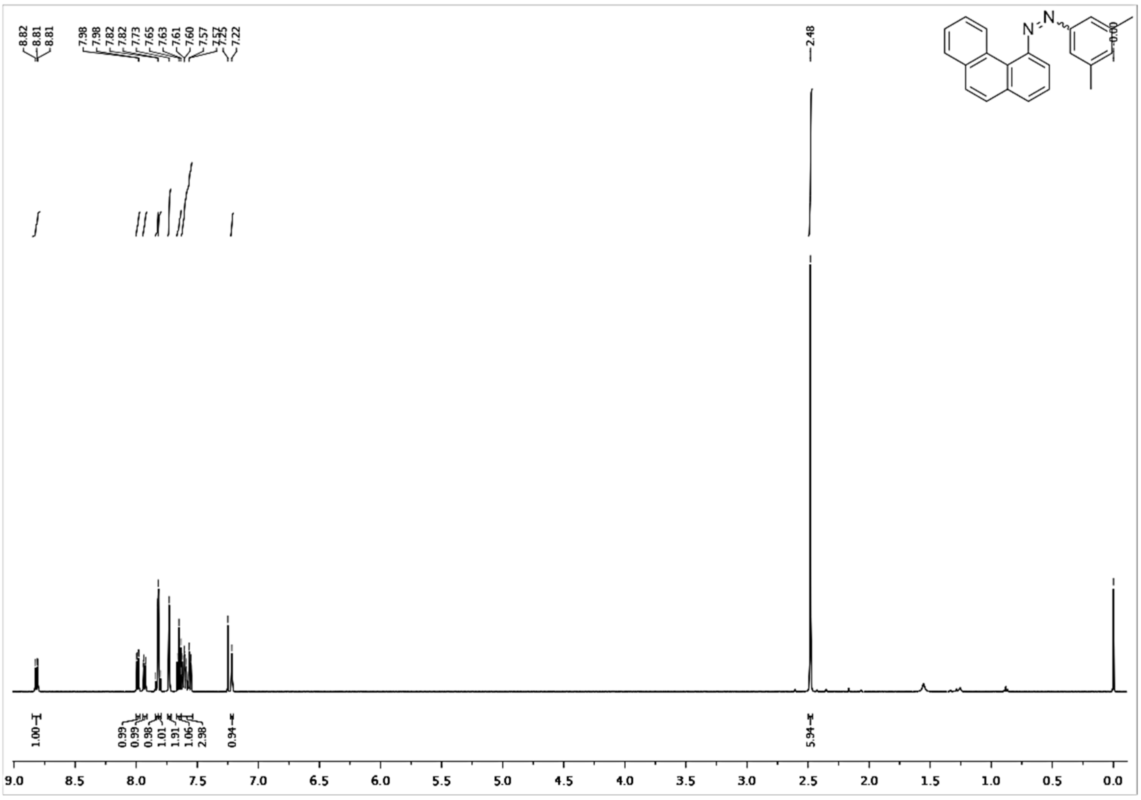

^{13}C APT NMR (126 MHz, Chloroform-*d*) of **p3f**

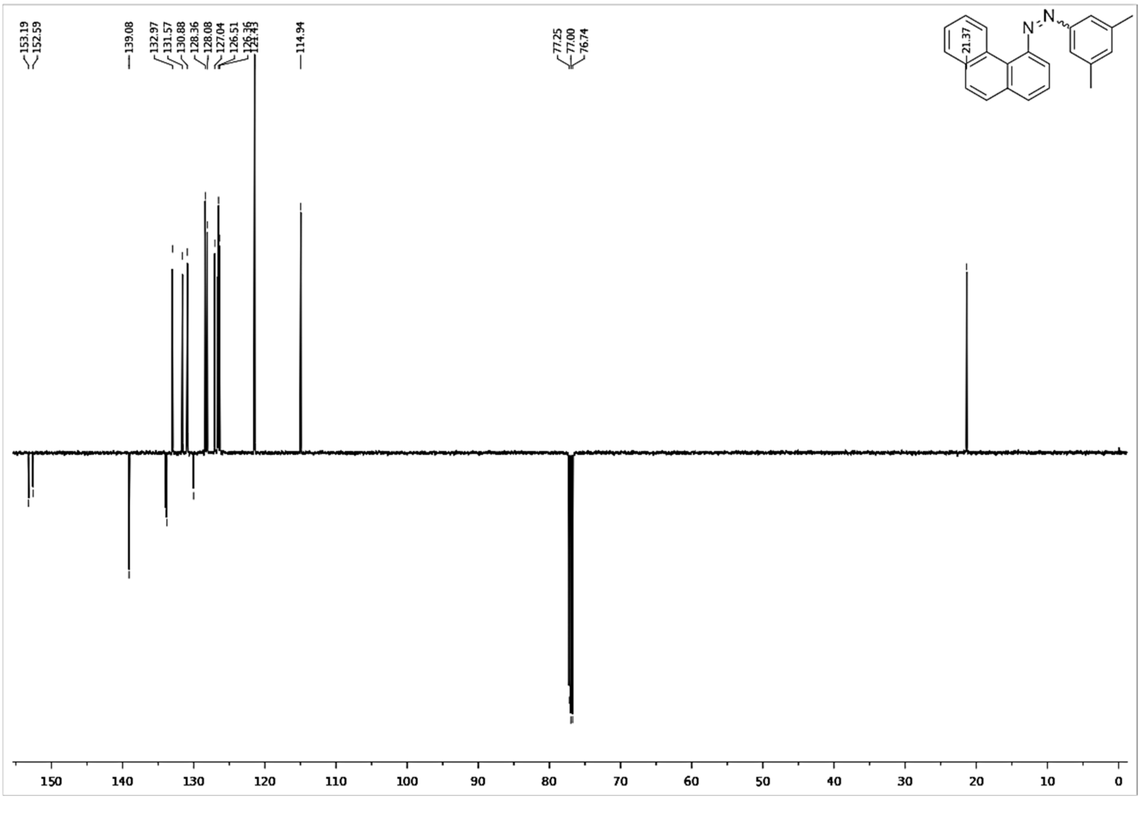

^{1}H NMR (500 MHz, Chloroform-*d*) of **p3g**

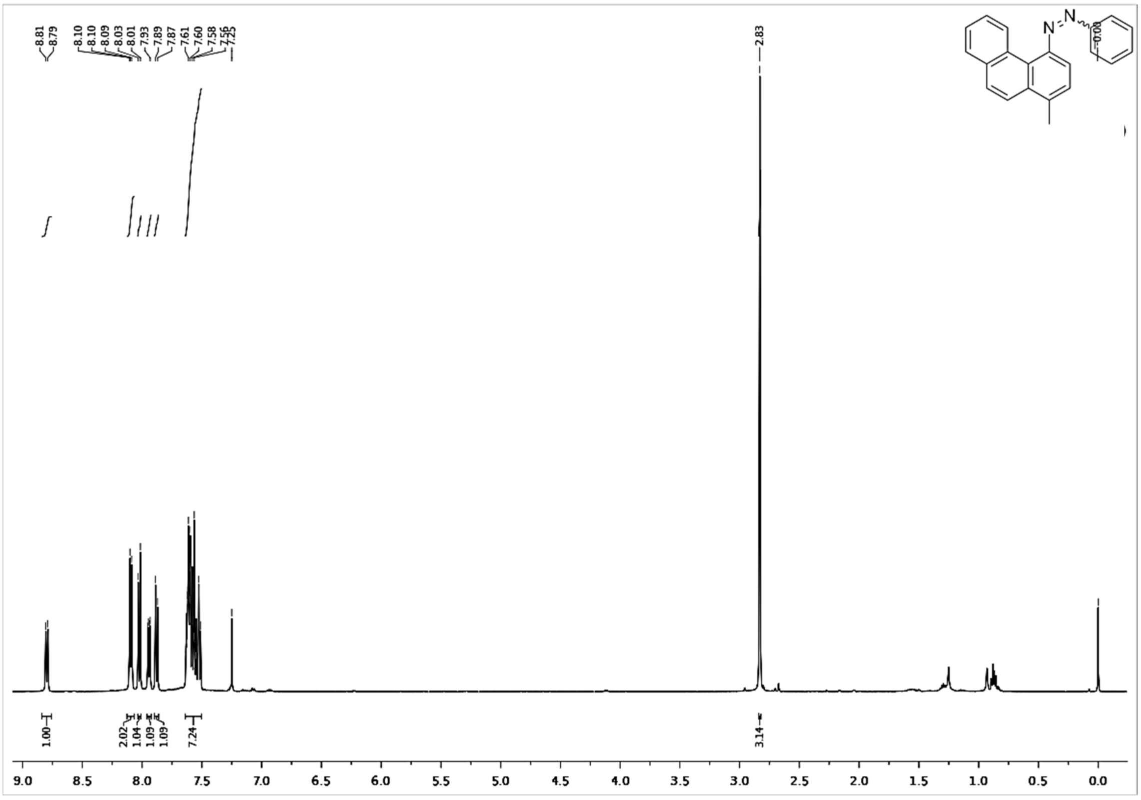

^{13}C APT NMR (126 MHz, Chloroform-*d*) of **p3g**

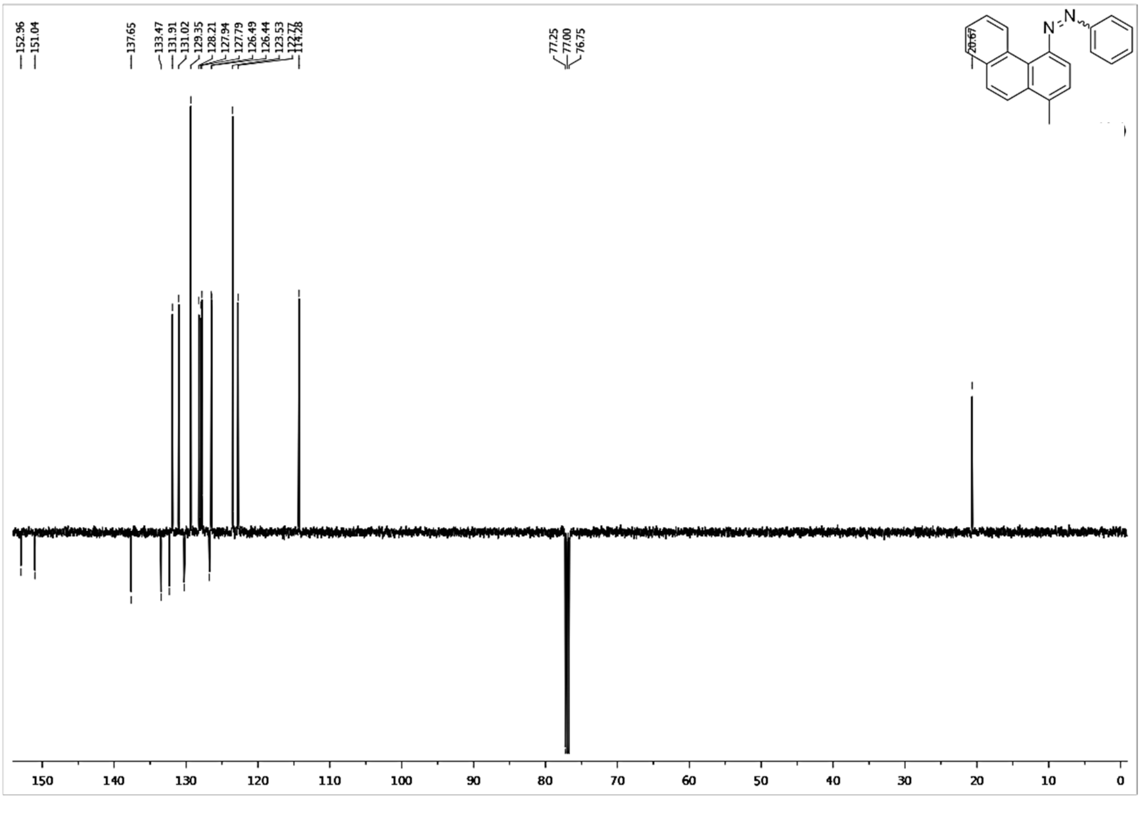

^{1}H NMR (500 MHz, Chloroform-*d*) of **p3h**

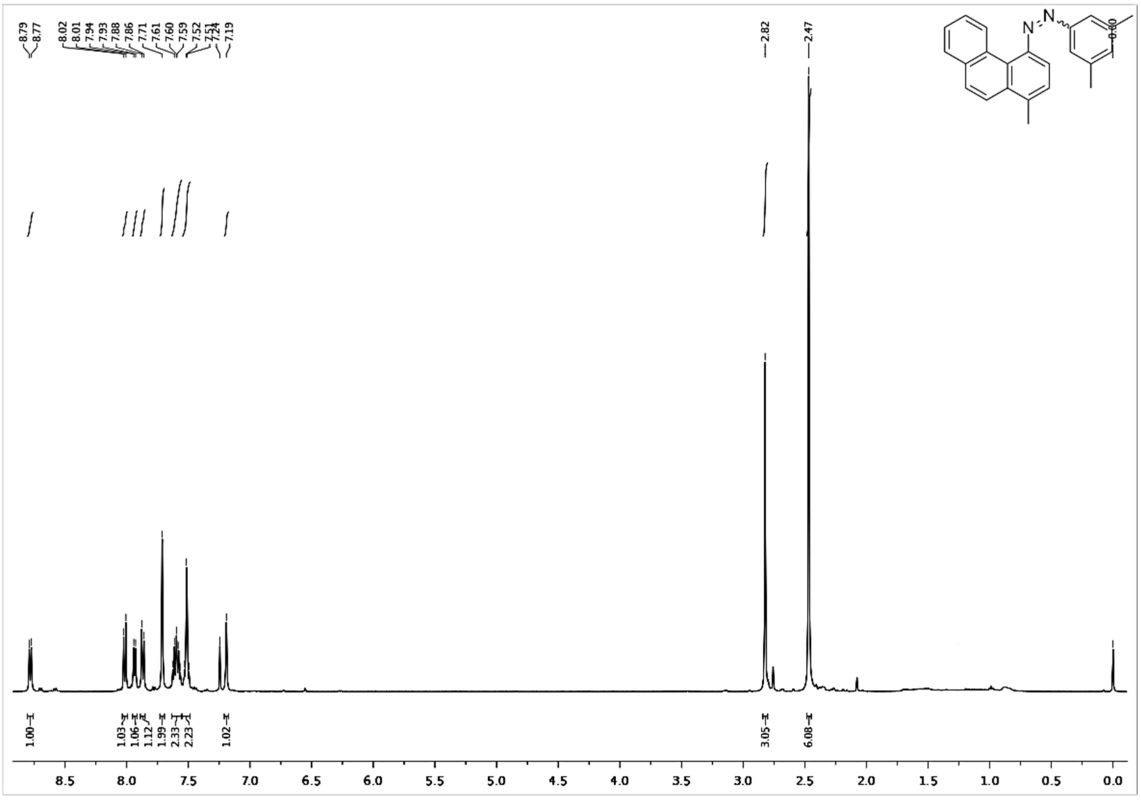

^{13}C APT NMR (126 MHz, Chloroform-*d*) of **p3h**

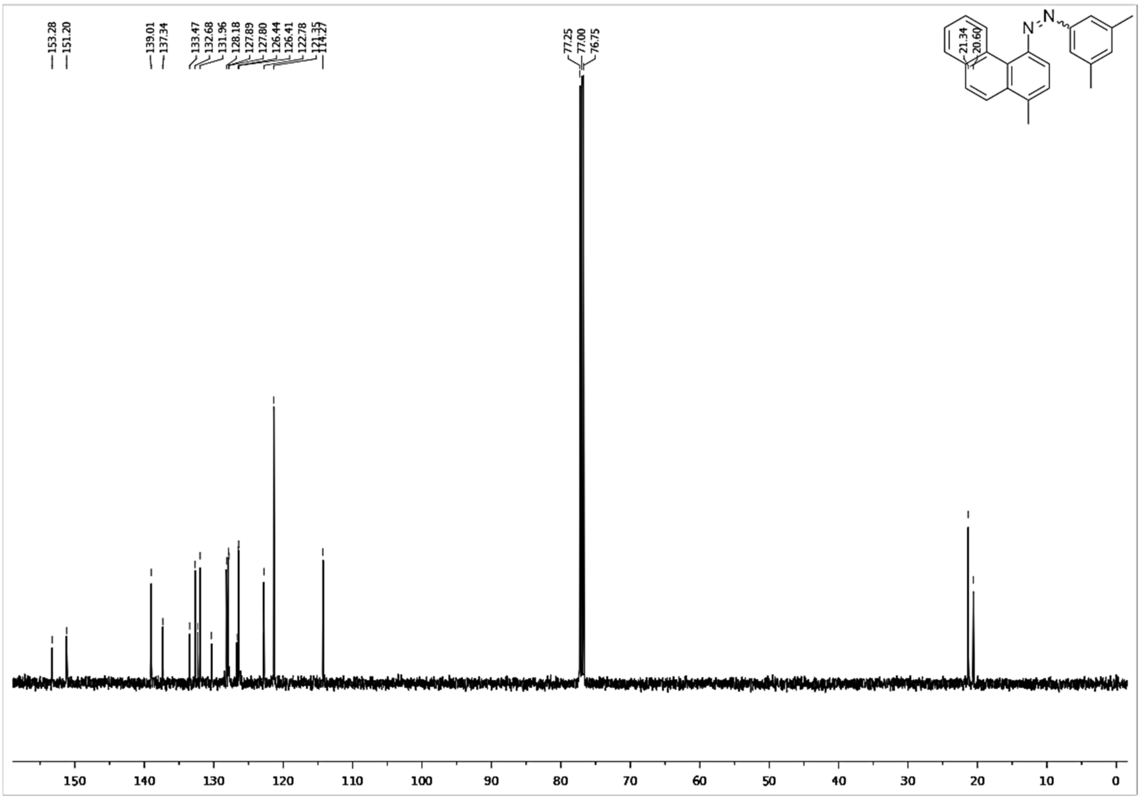

^{1}H NMR (500 MHz, Chloroform-*d*)

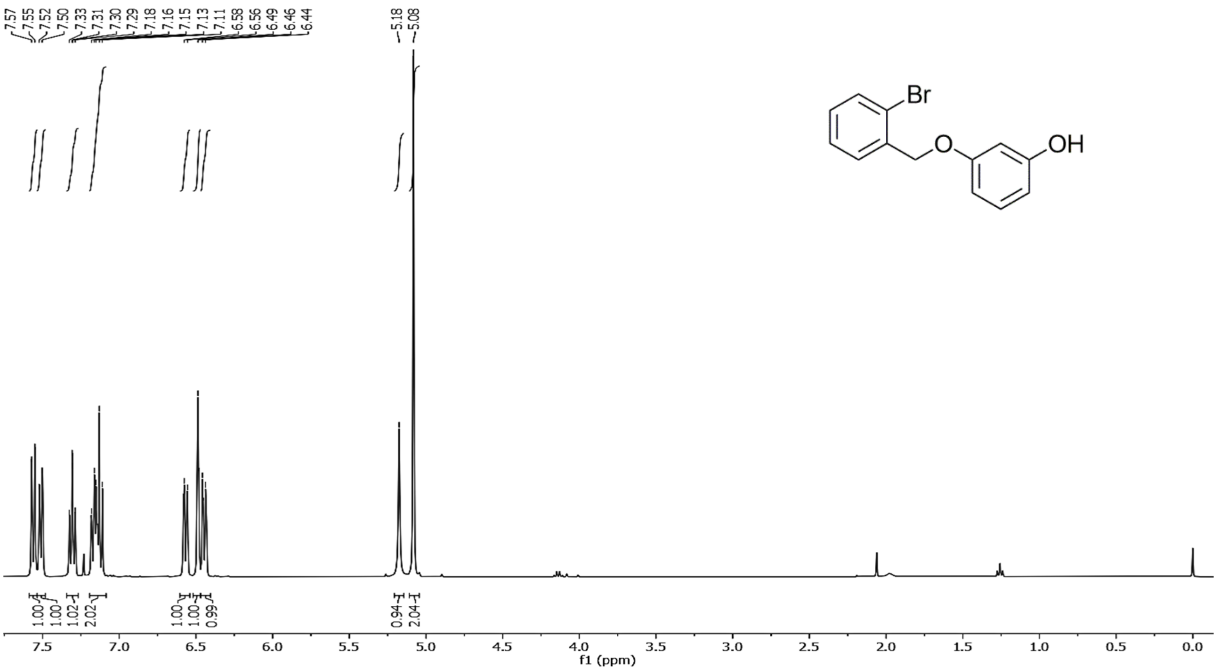

$^{13}C\{1H\}$NMR (126 MHz, Chloroform-*d*)

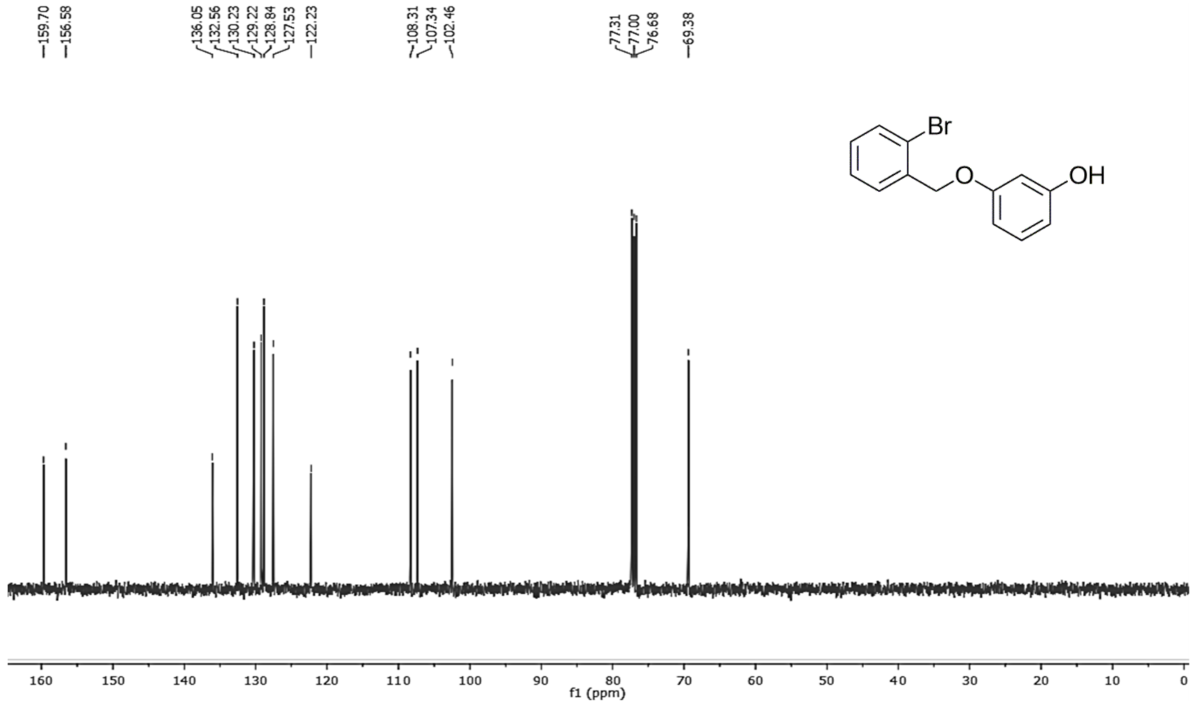

^{1}H NMR (500 MHz, Chloroform-d)

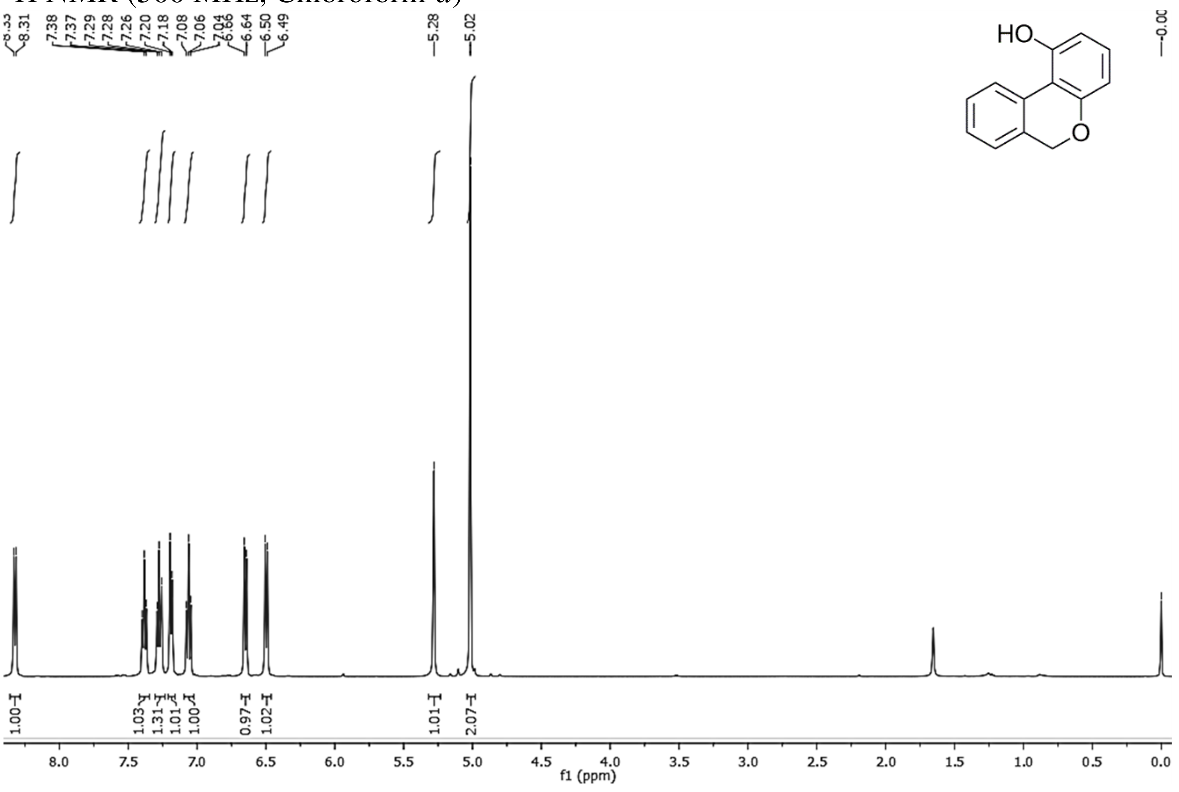

^{13}C APT NMR (126 MHz, Chloroform-d)

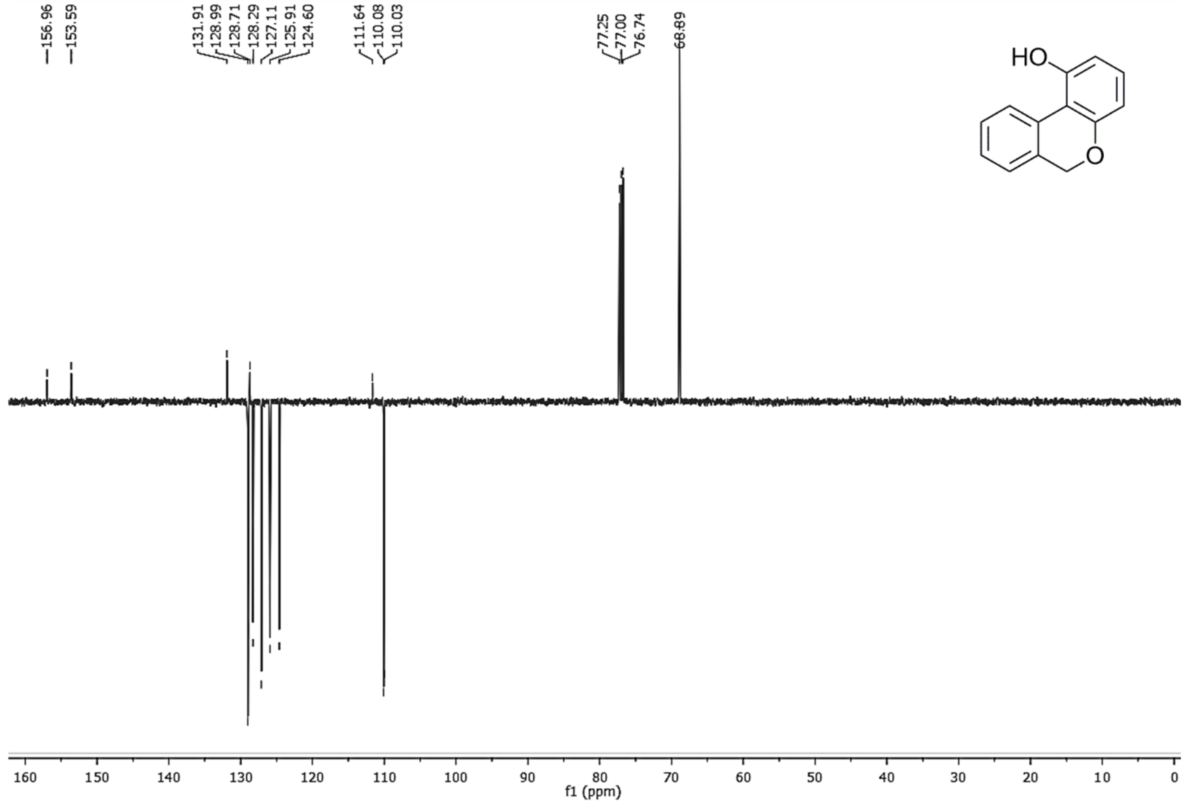

^{1}H NMR (500 MHz, Chloroform-*d*)

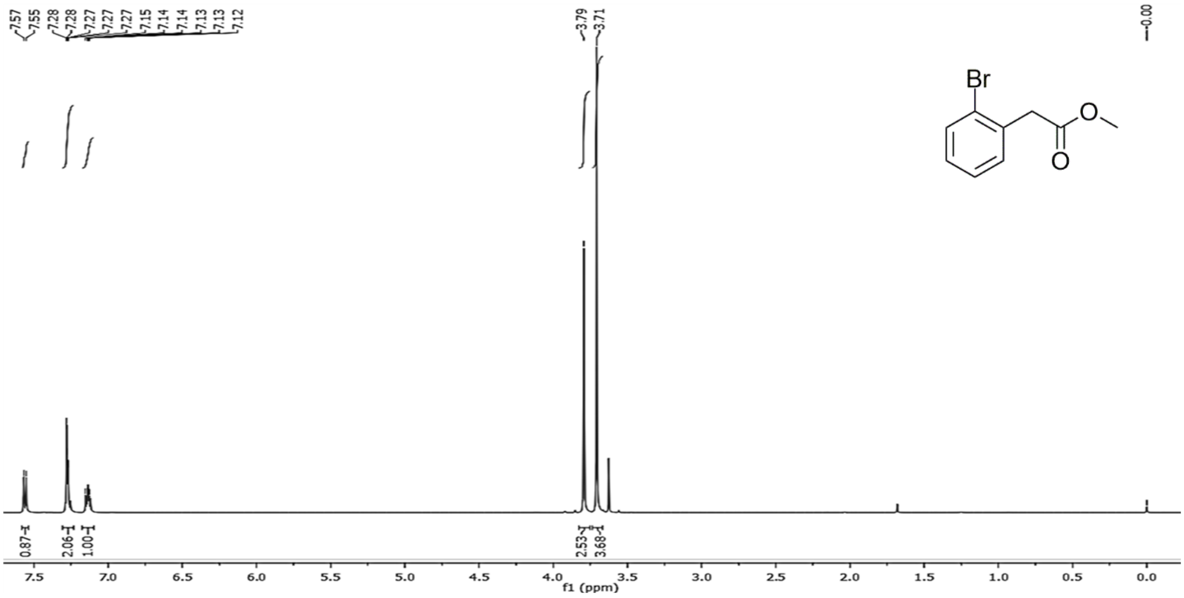

^{13}C APT NMR (126 MHz, Chloroform-*d*)

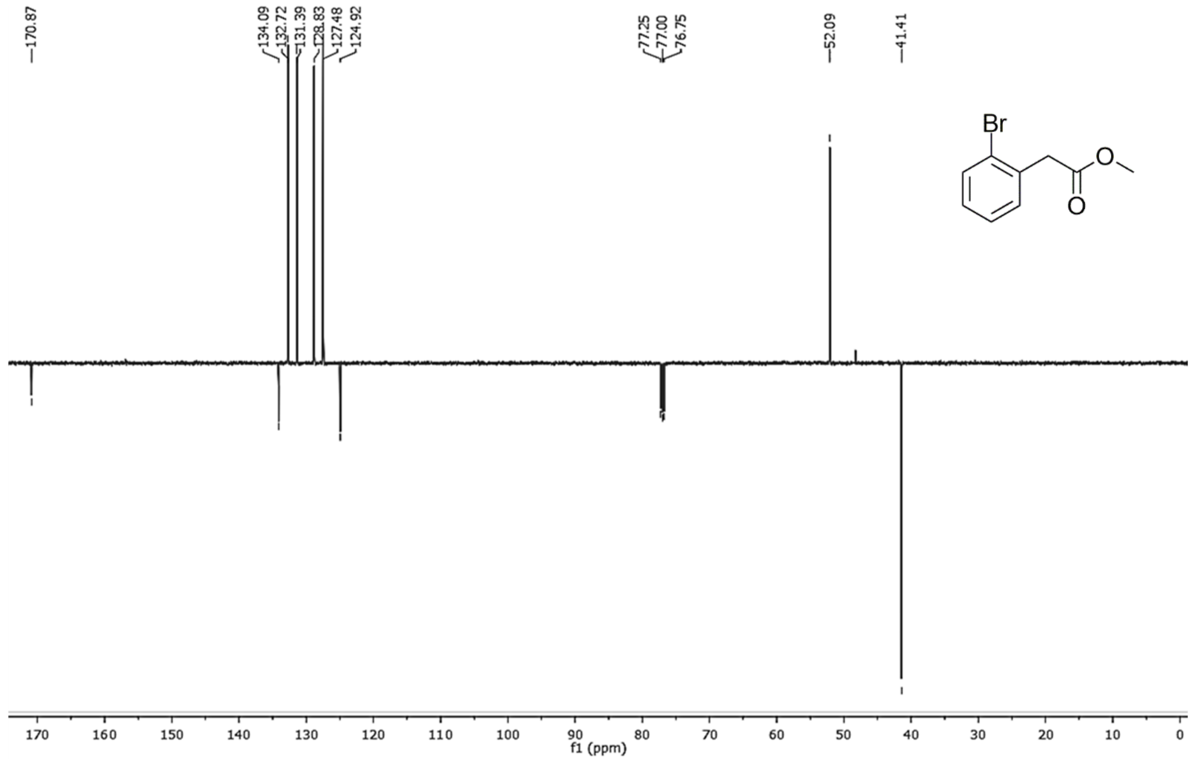

^{1}H NMR (500 MHz, Chloroform-*d*)

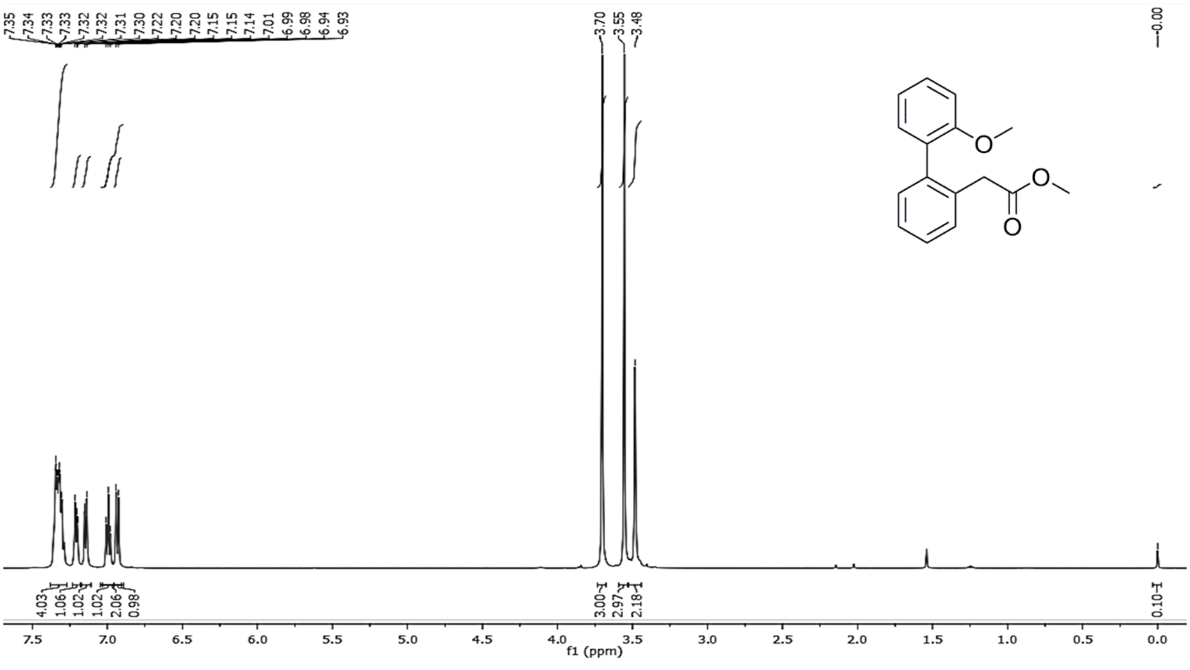

^{13}C APT NMR (126 MHz, Chloroform-*d*)

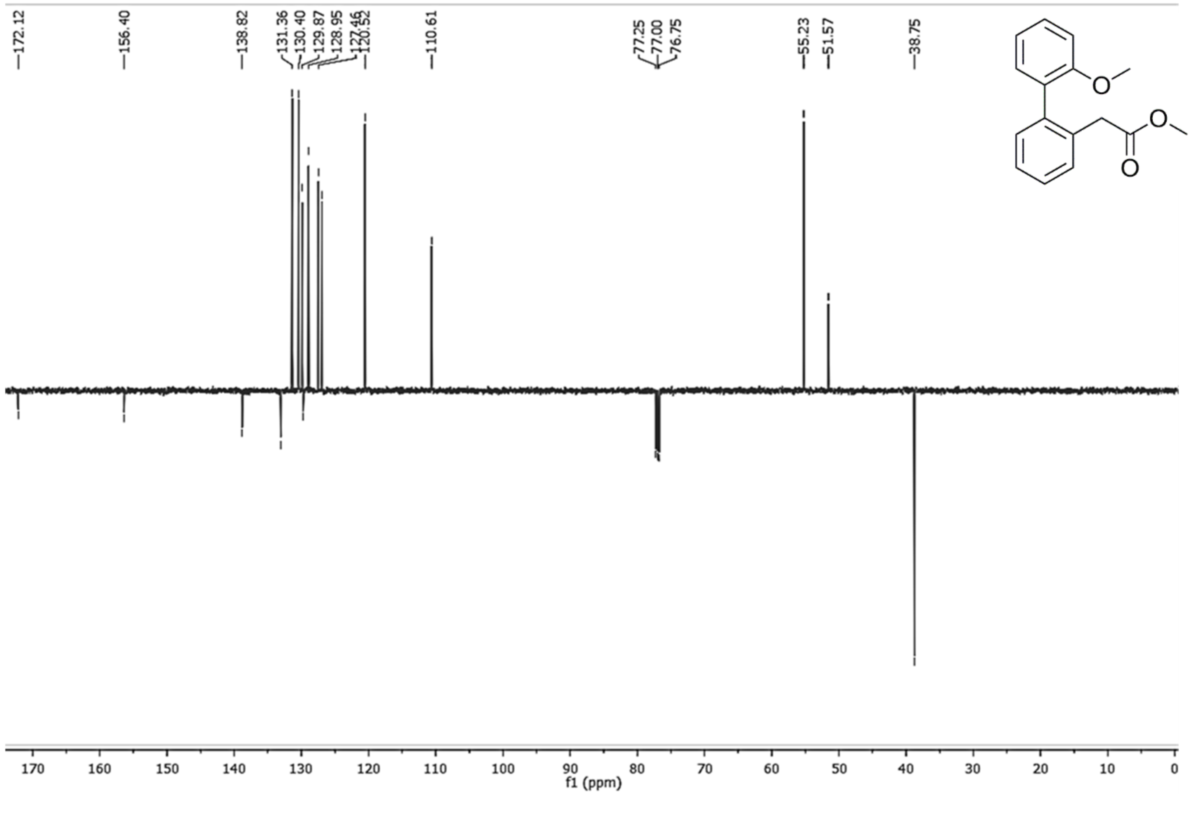

^{1}H NMR (500 MHz, Chloroform-*d*)

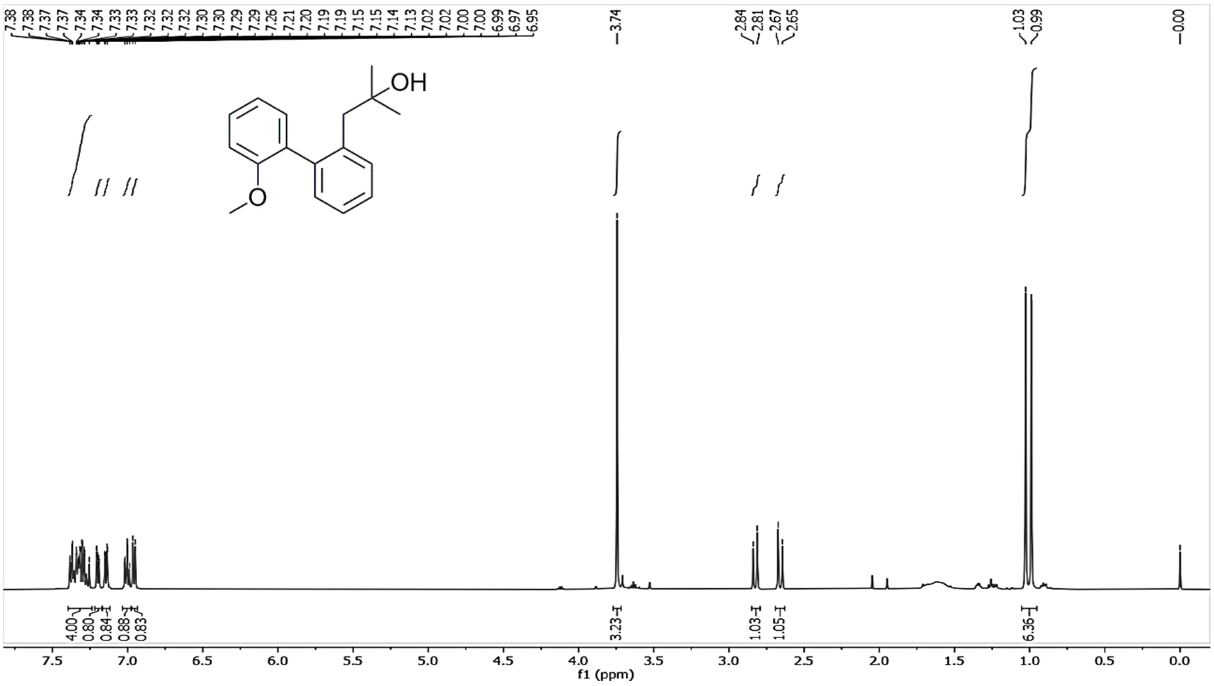

$^{13}C\{1H\}$NMR (126 MHz, Chloroform-*d*)

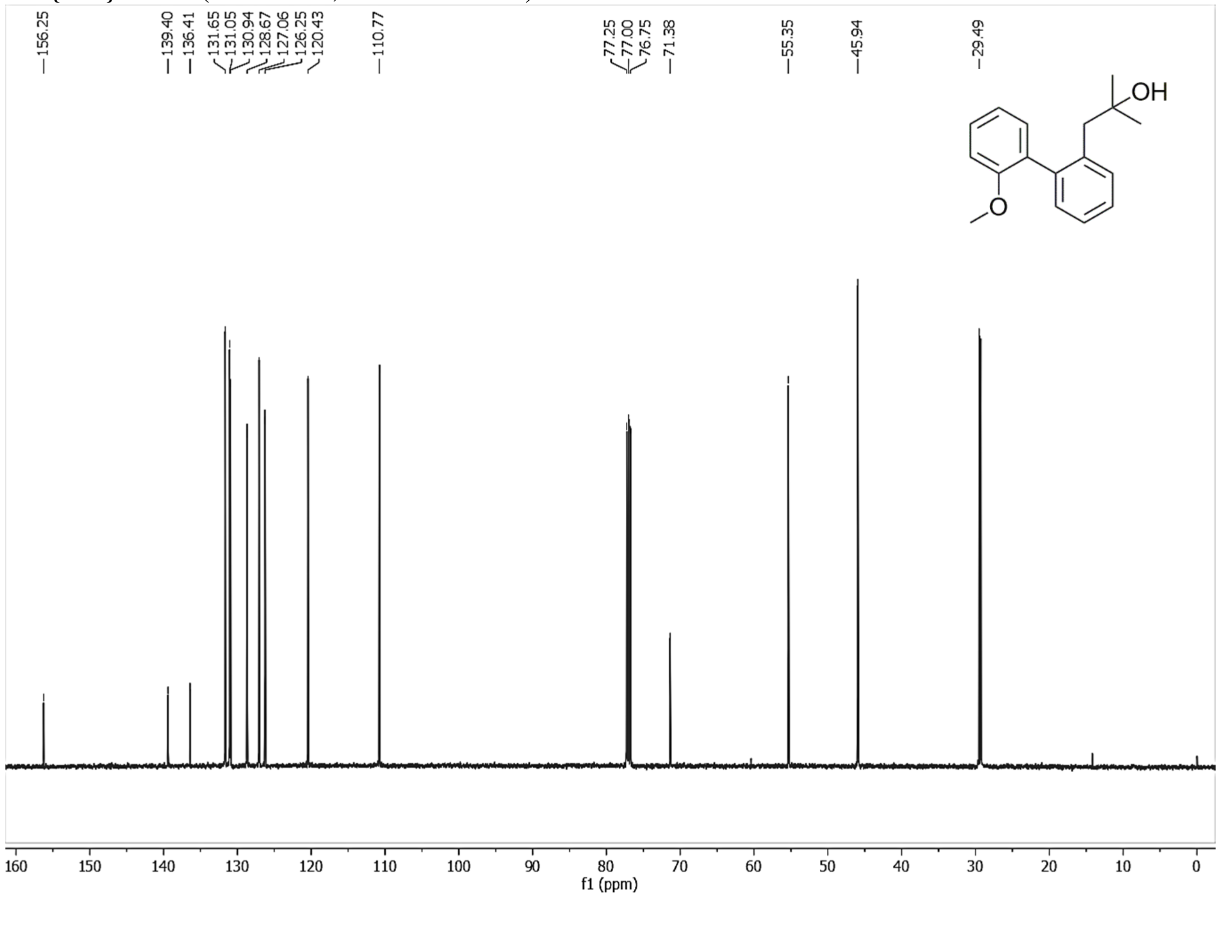

^{1}H NMR (500 MHz, Chloroform-*d*)

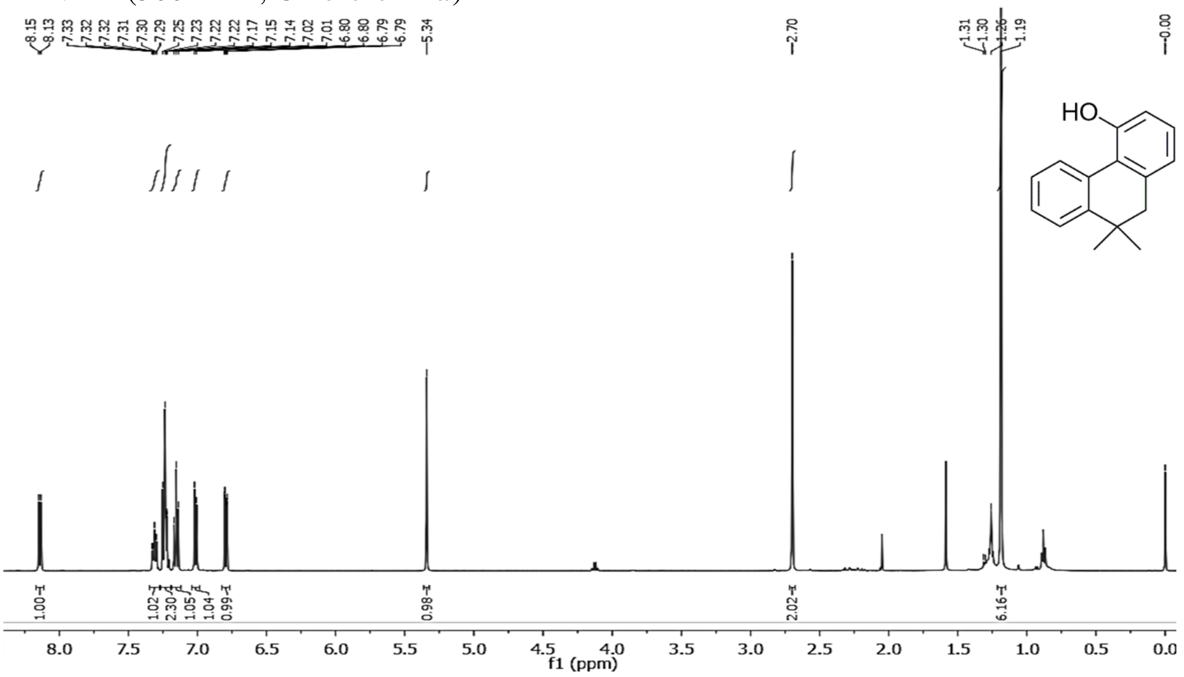

^{13}C APT NMR (126 MHz, Chloroform-*d*)

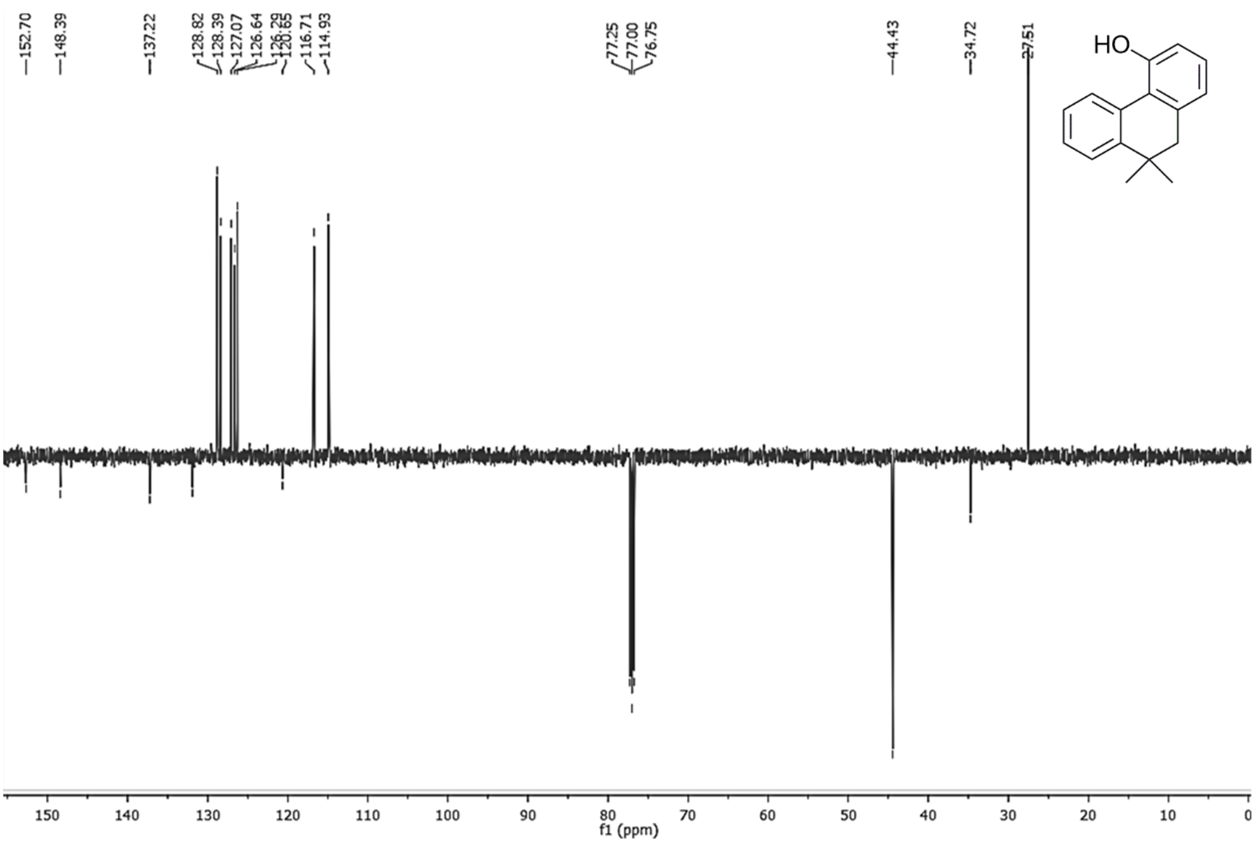

^{1}H NMR (500 MHz, Chloroform-*d*) of **p1a**

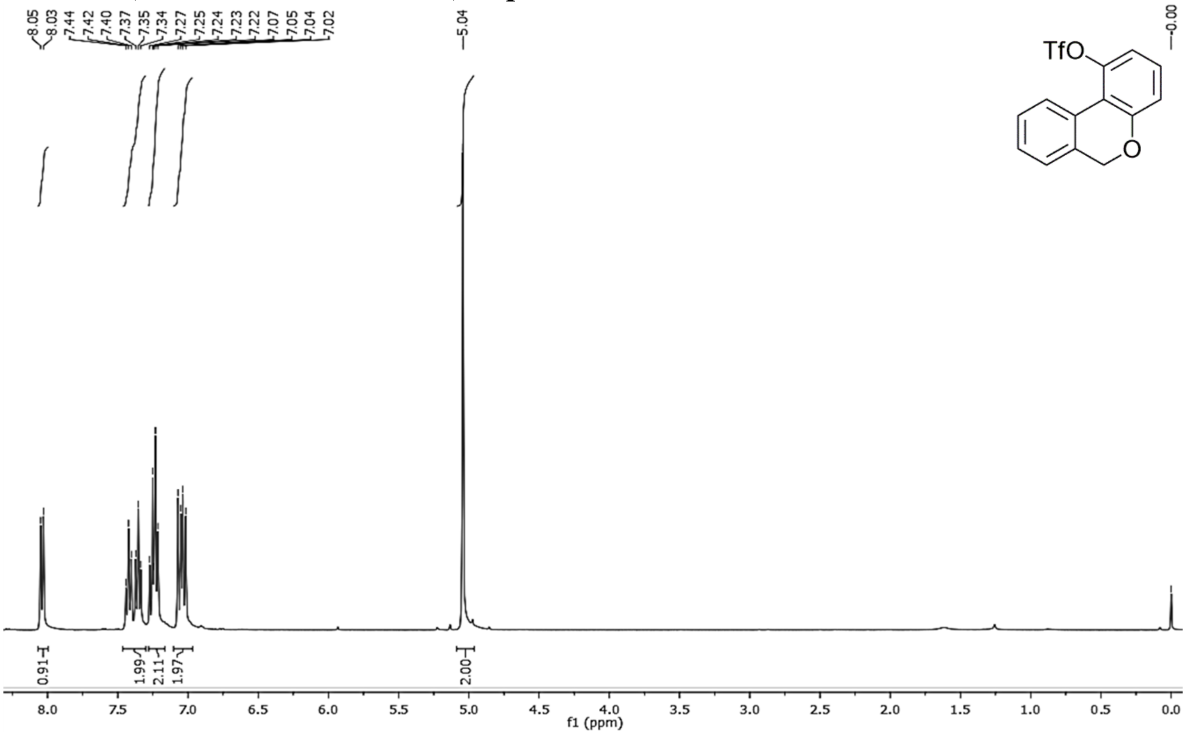

^{13}C{1H}NMR (126 MHz, Chloroform-*d*) of **p1a**

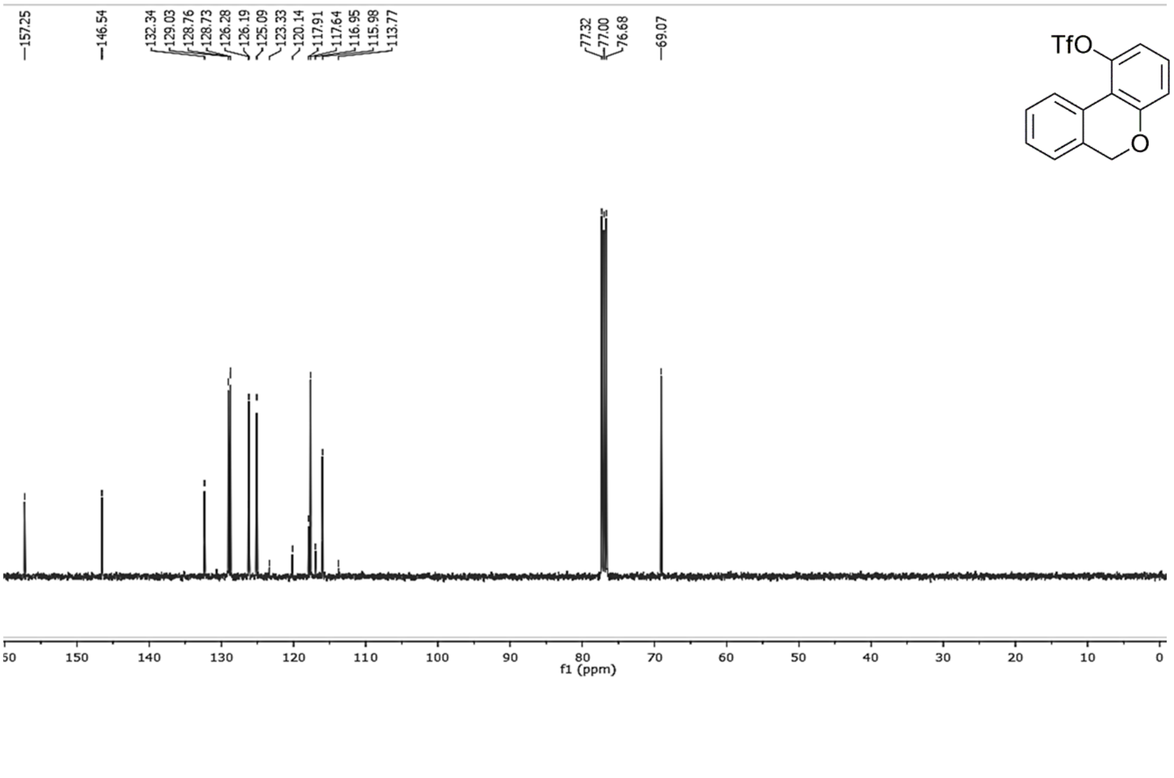

^{1}H NMR (500 MHz, Chloroform-*d*) of **p1c**

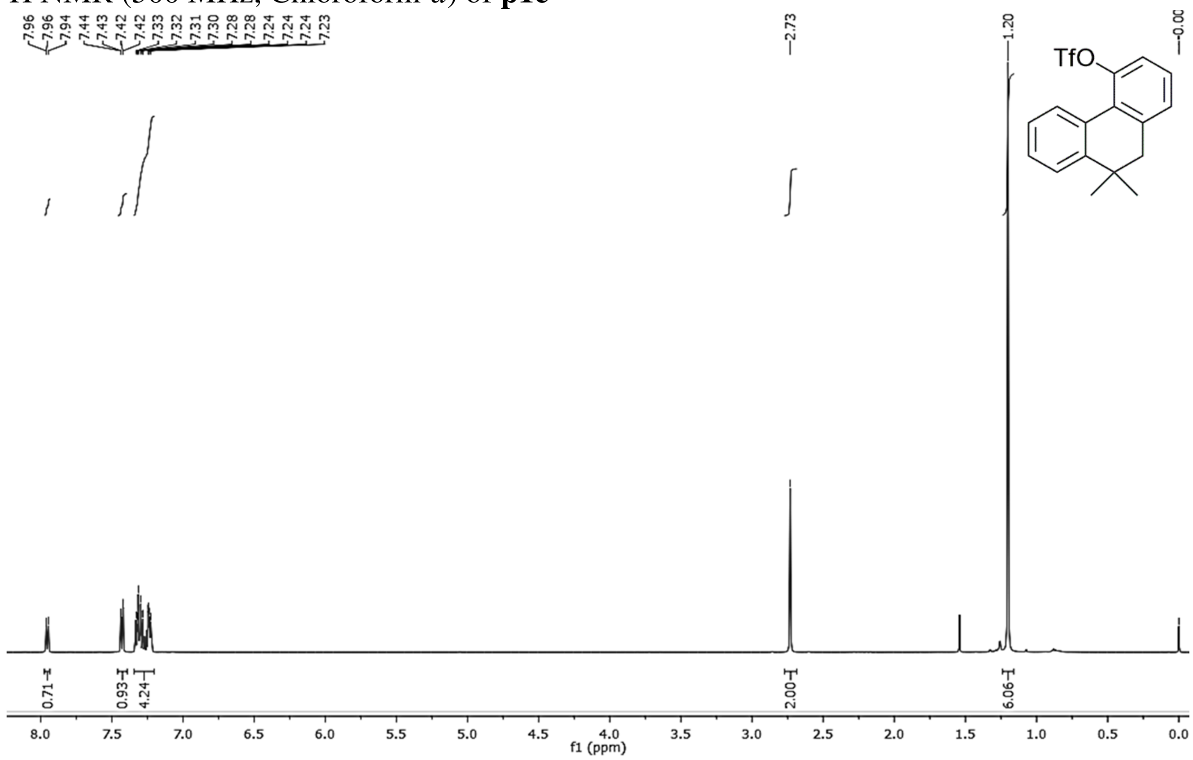

^{13}C APT NMR (126 MHz, Chloroform-*d*) of **p1c**

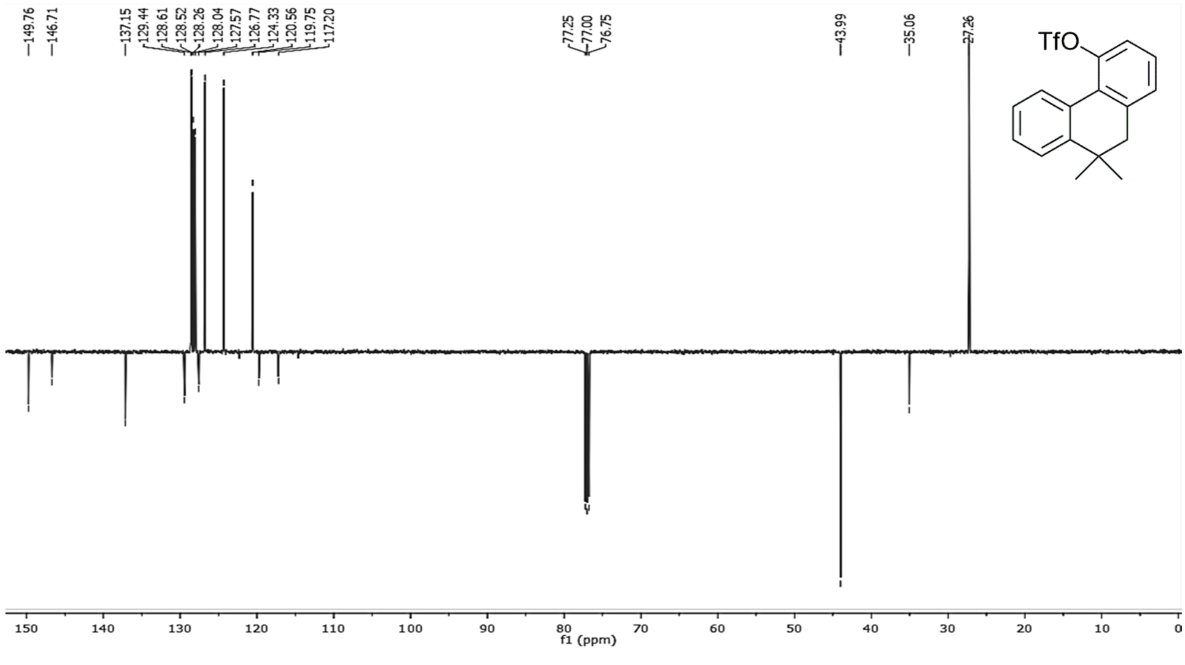

^{1}H NMR (500 MHz, Chloroform-*d*) of **p3a**

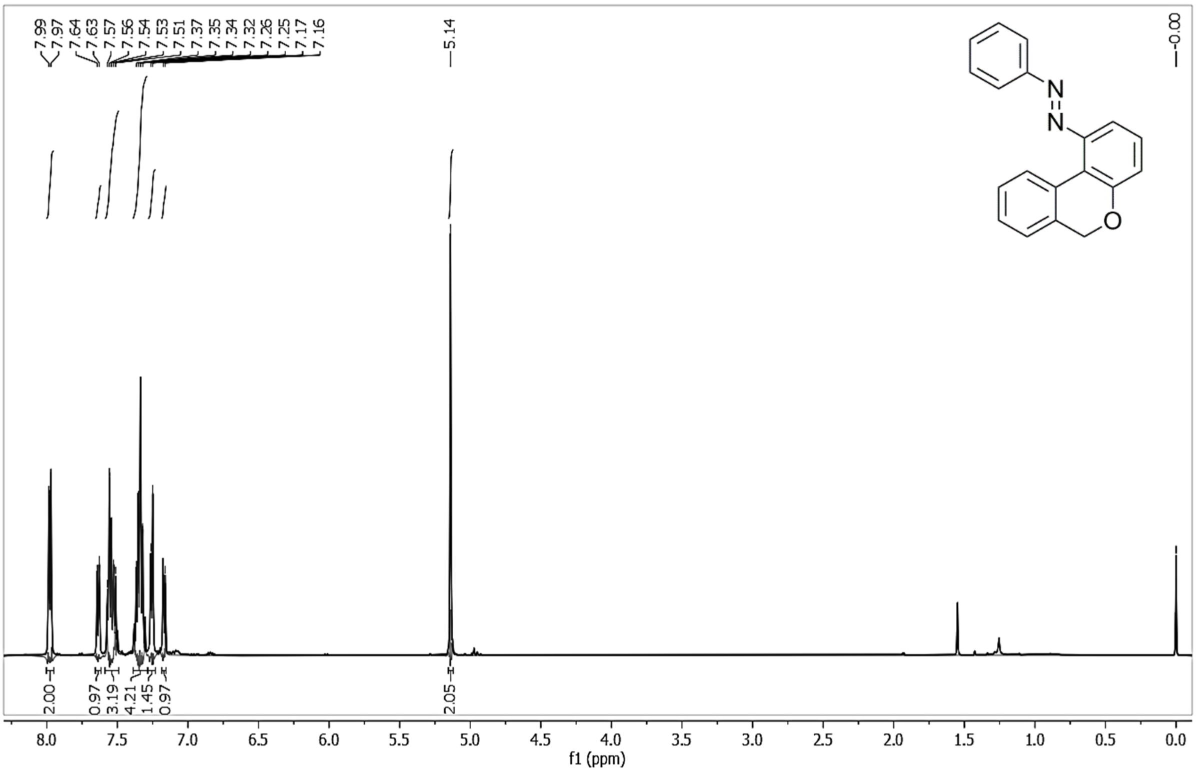

^{13}C APT NMR (126 MHz, Chloroform-*d*) of **p3a**

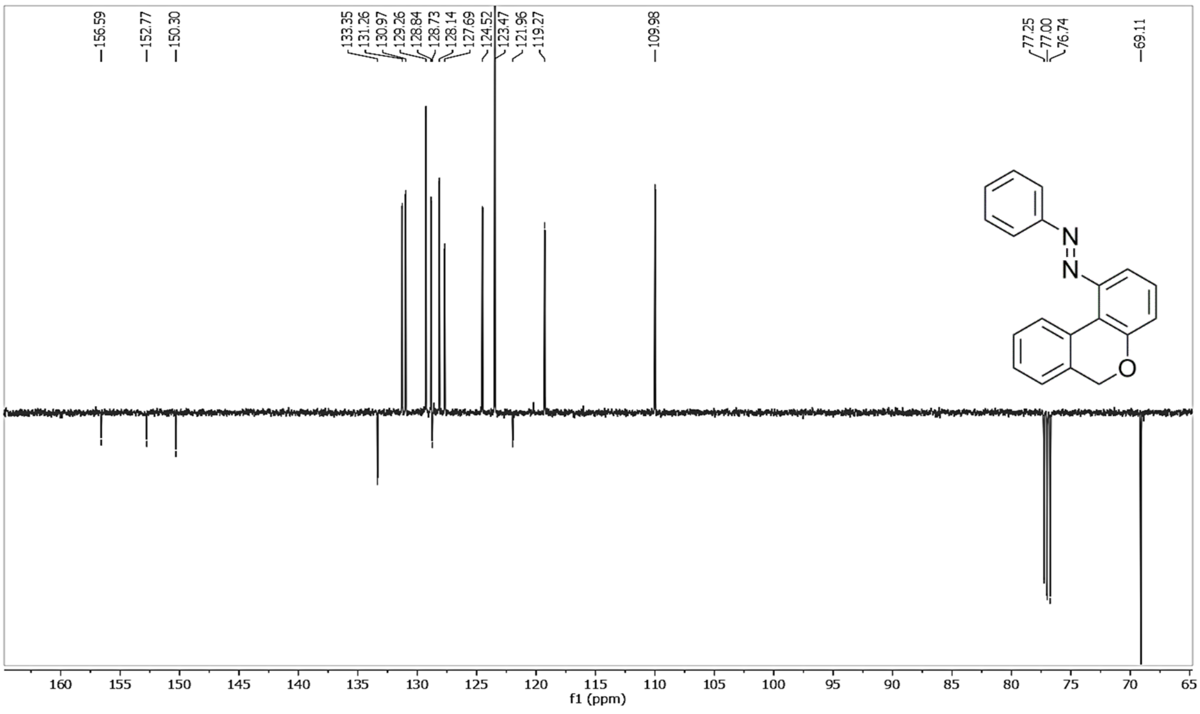

^{1}H NMR (500 MHz, Chloroform-*d*) of **p3b**

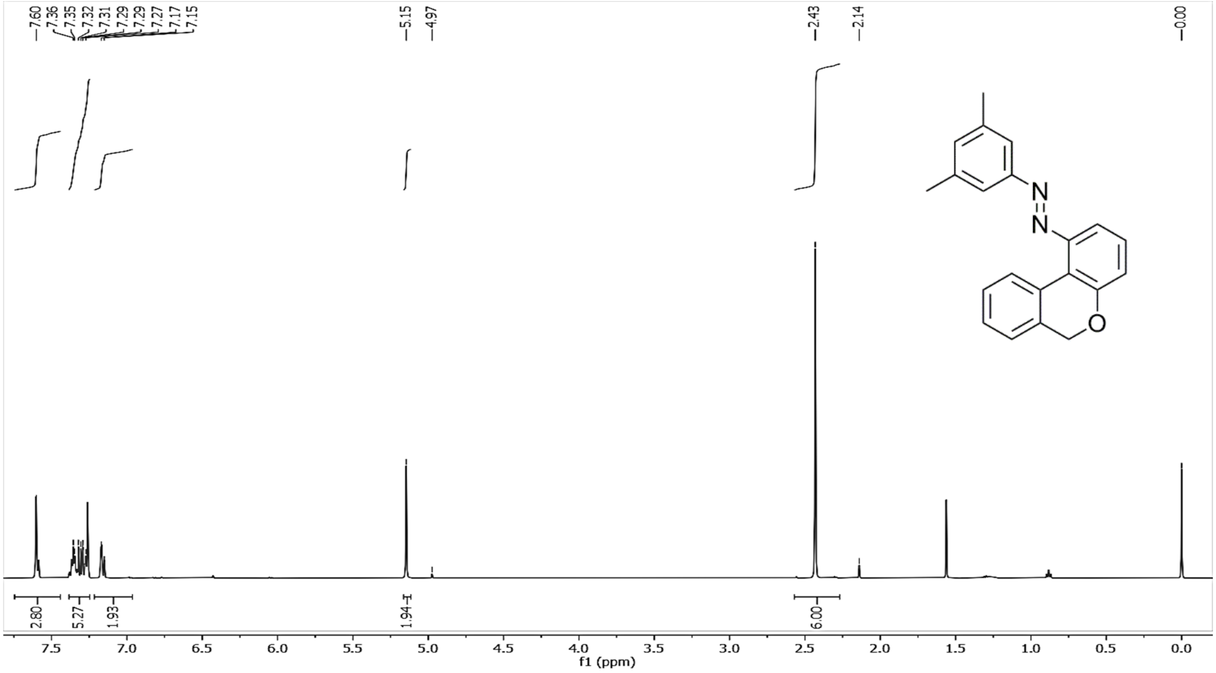

^{13}C APT NMR (126 MHz, Chloroform-*d*) of **p3b**

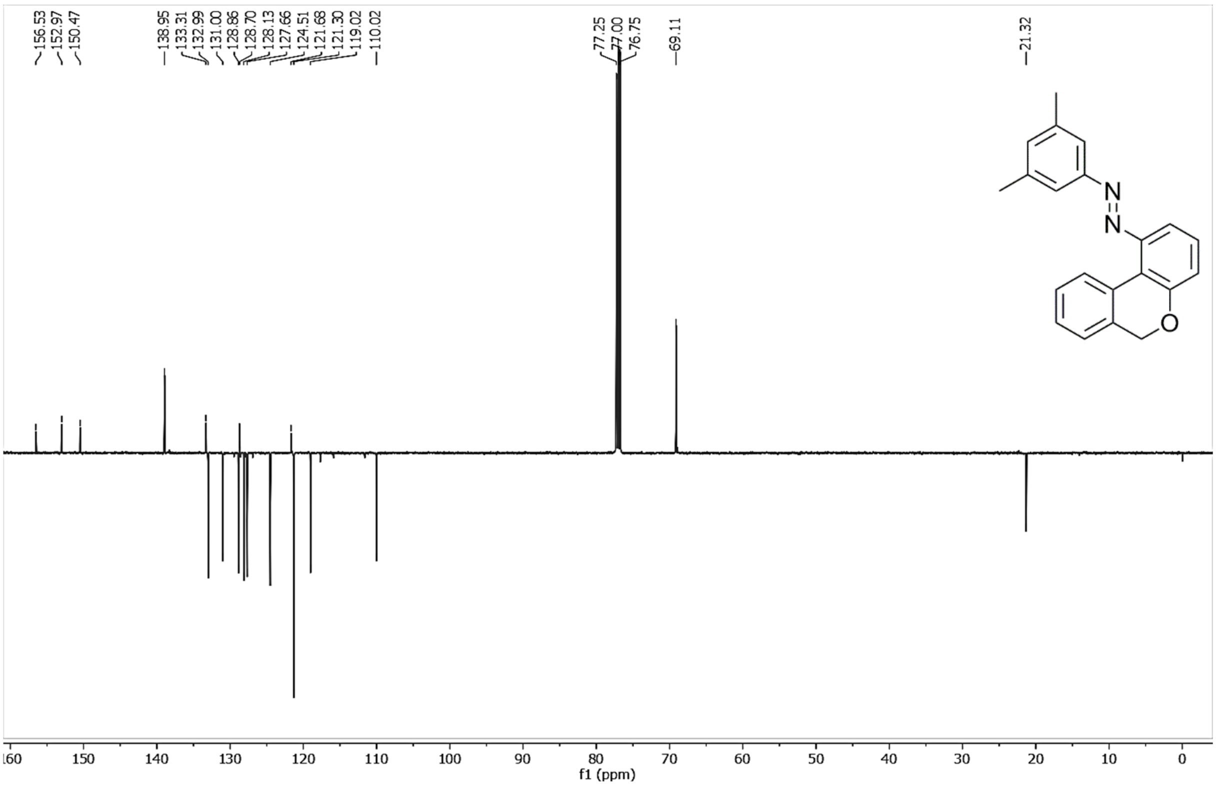

^{1}H NMR (500 MHz, Chloroform-*d*) of **p3c**

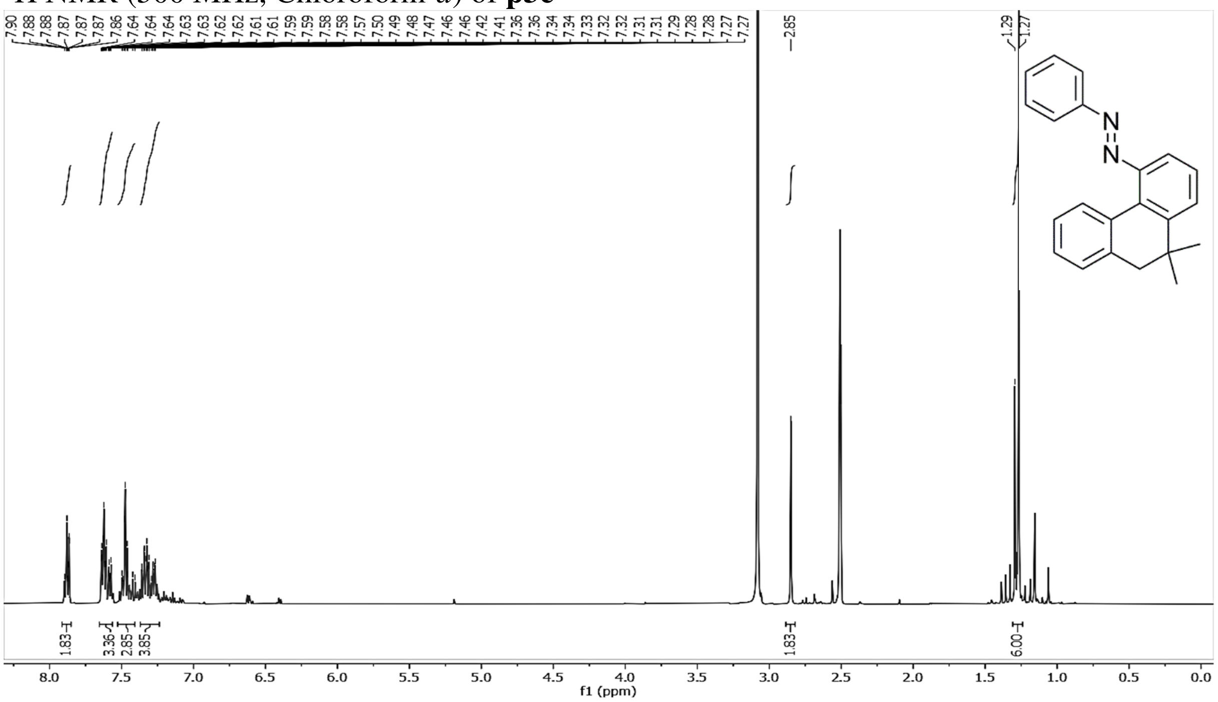

^{13}C APT NMR (126 MHz, Chloroform-*d*) of **p3c**

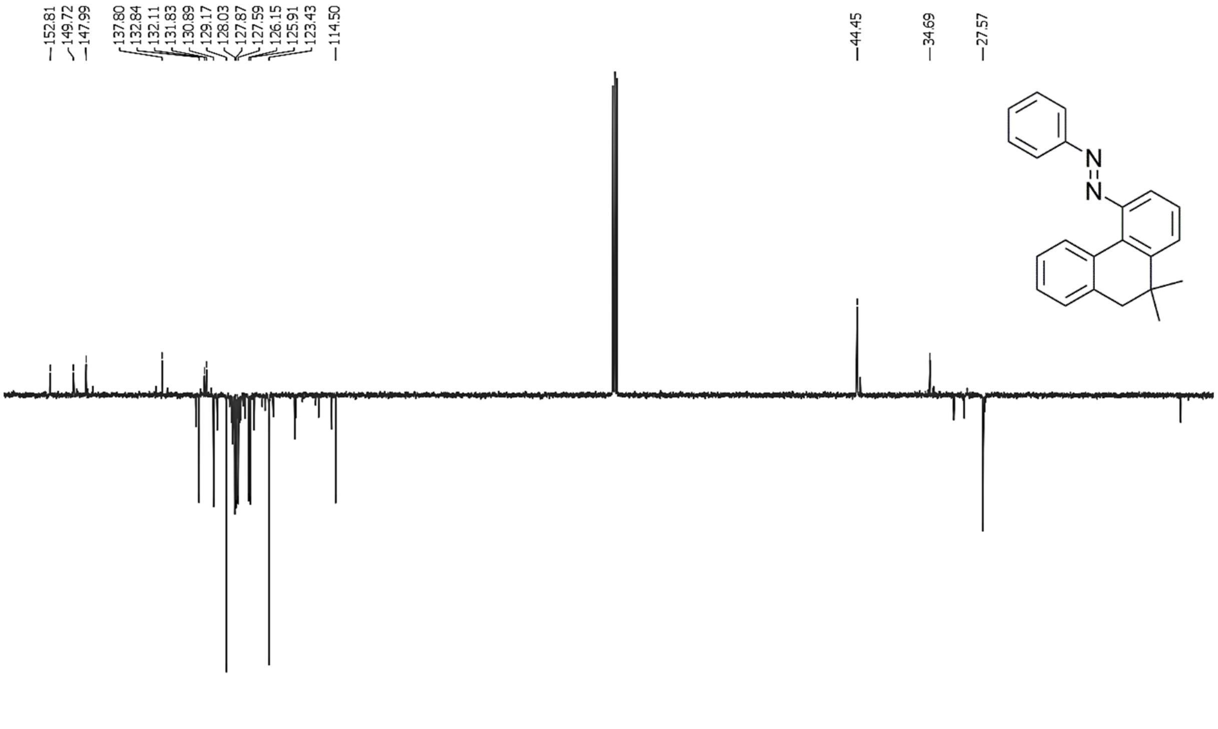

^{1}H NMR (500 MHz, Chloroform-*d*) of **p3d**

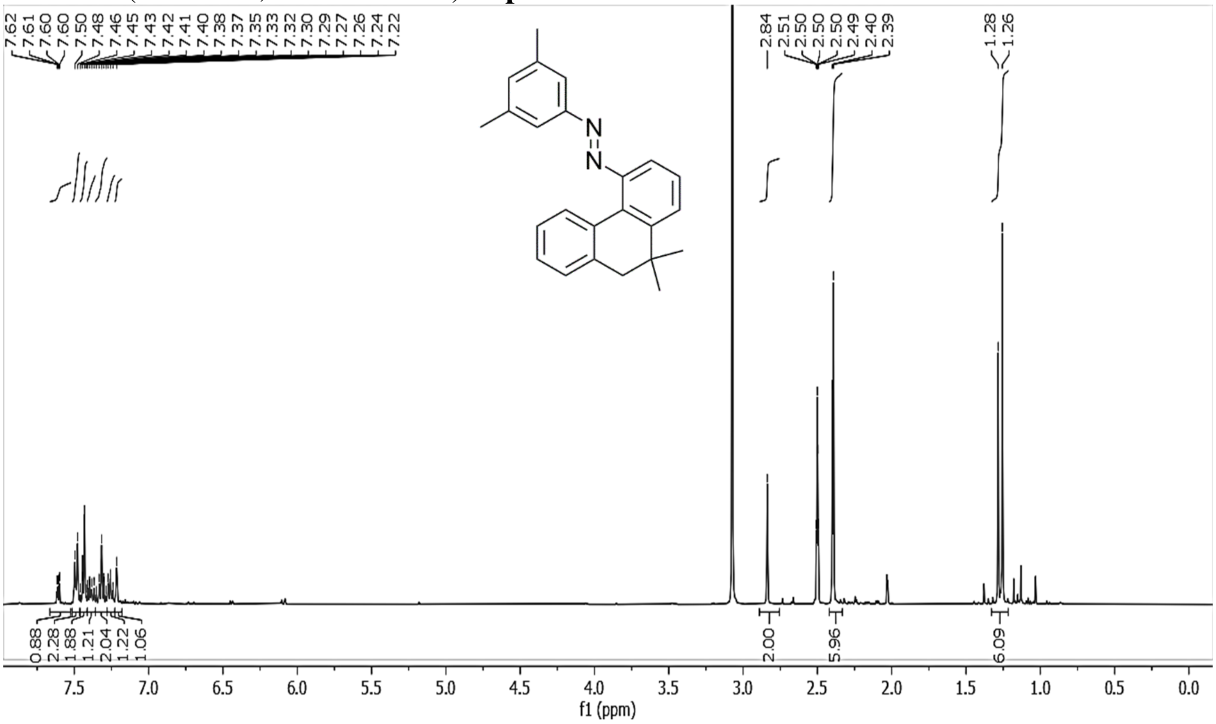

^{13}C APT NMR (126 MHz, Chloroform-*d*) of **p3d**

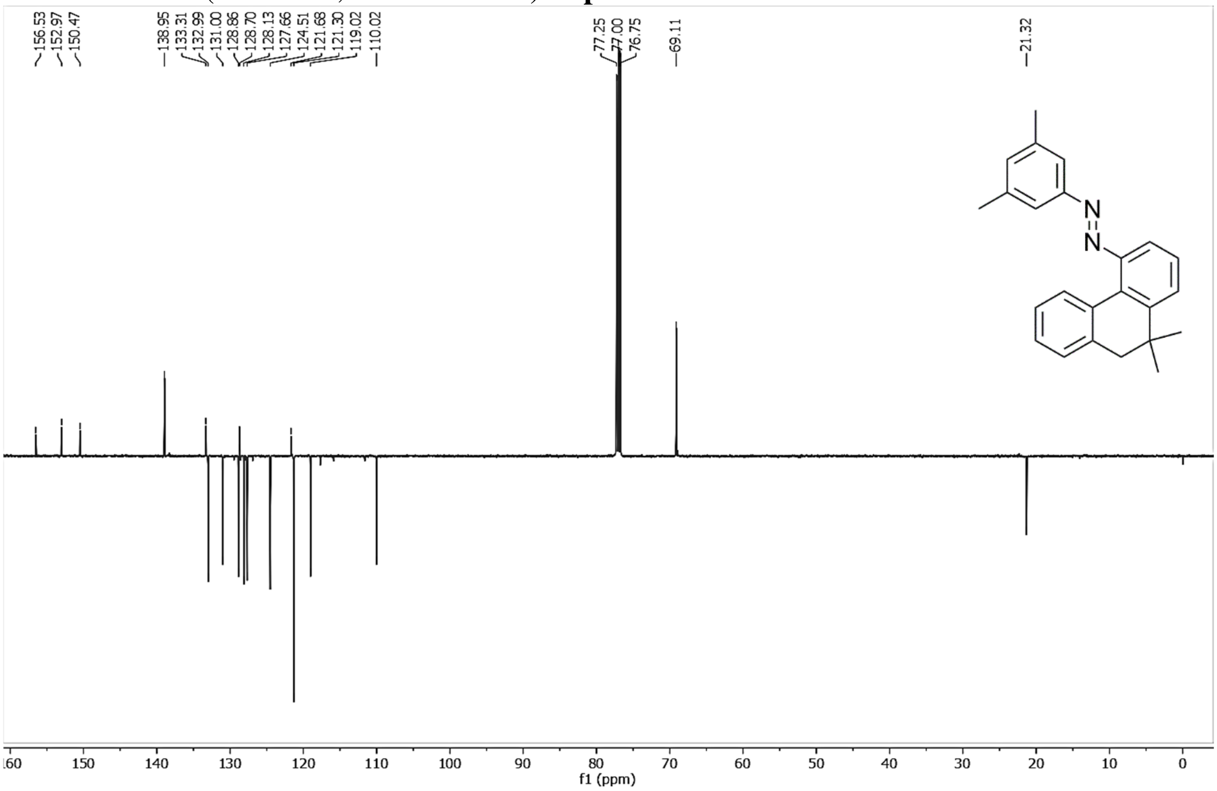

^{1}H NMR (500 MHz, Chloroform-*d*)

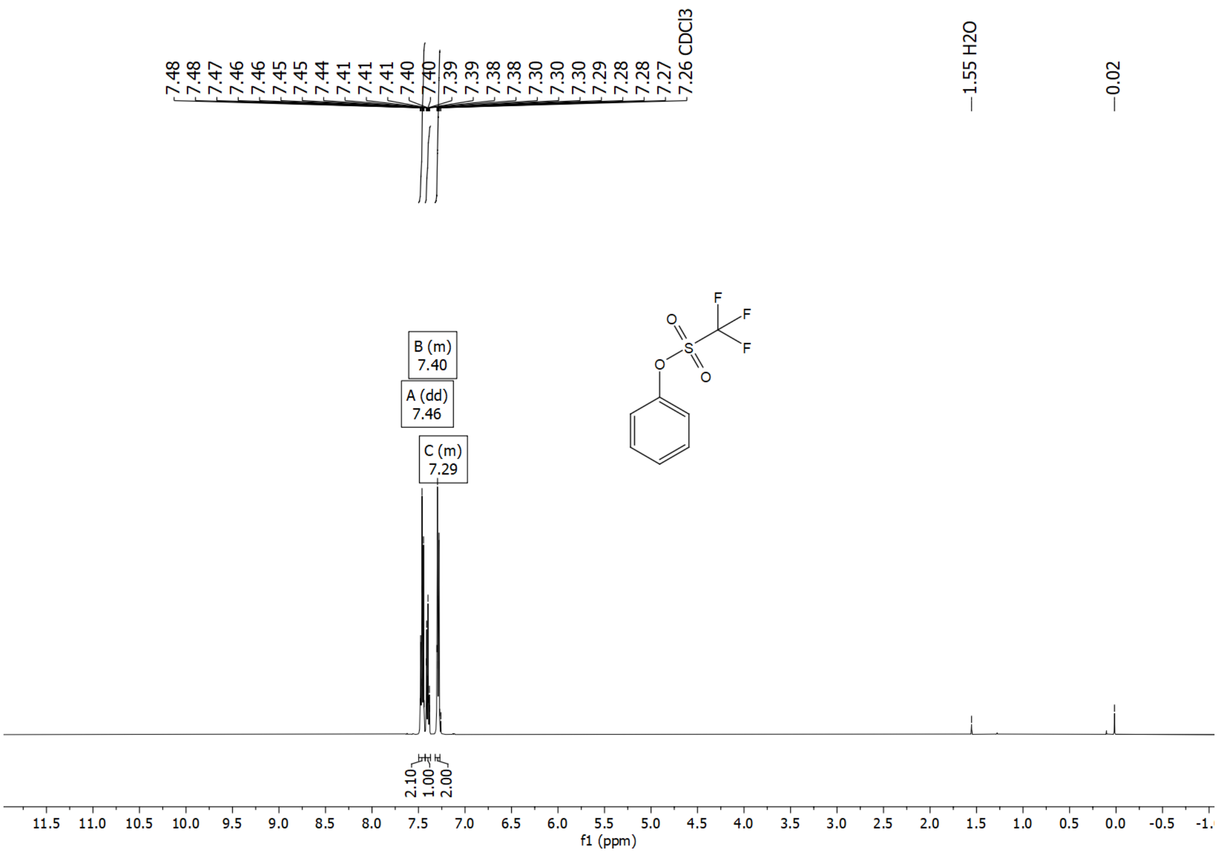

$^{13}C\{1H\}$NMR (126 MHz, $CDCl_3$)

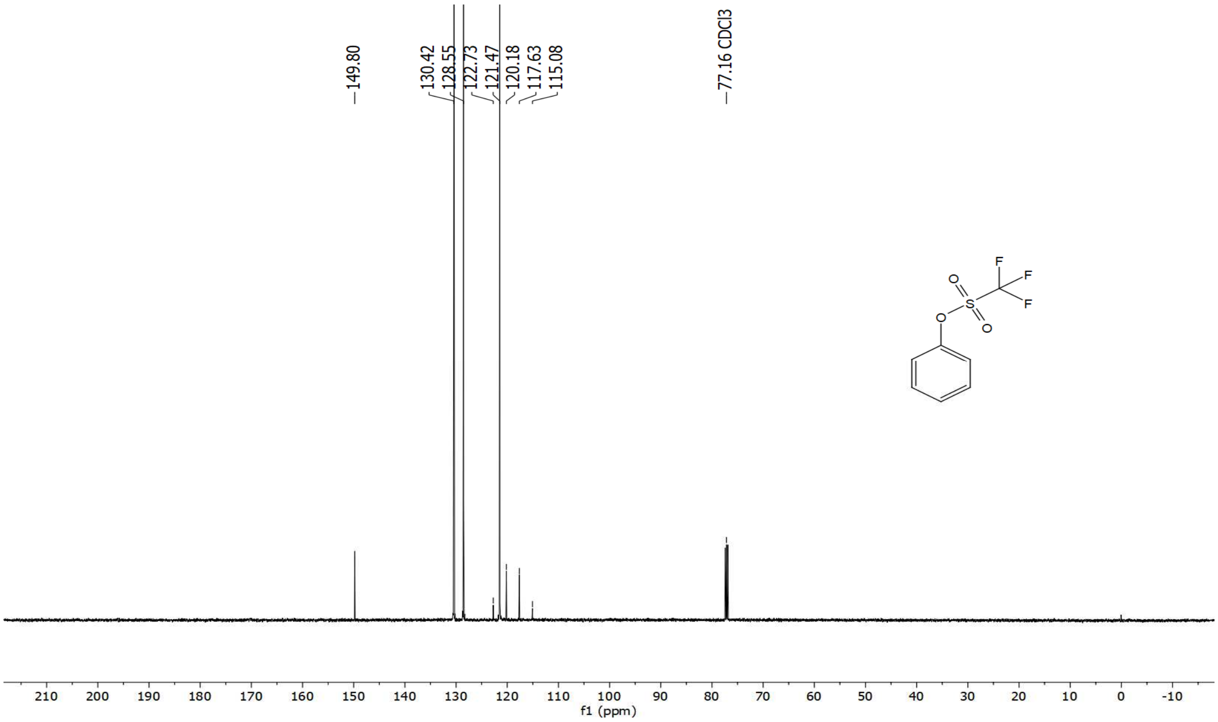

^{1}H NMR (500 MHz, Chloroform-*d*)

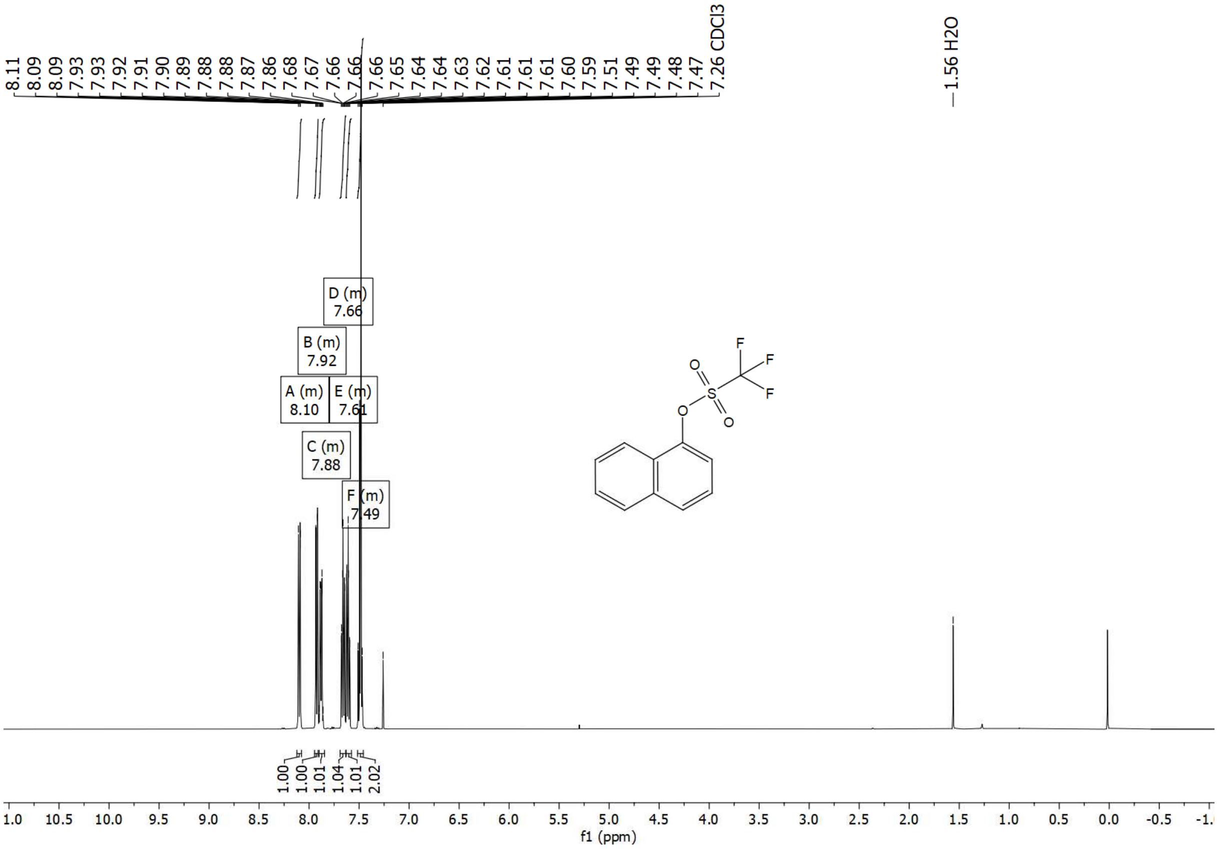

^{13}C APT NMR (126 MHz, $CDCl_3$)

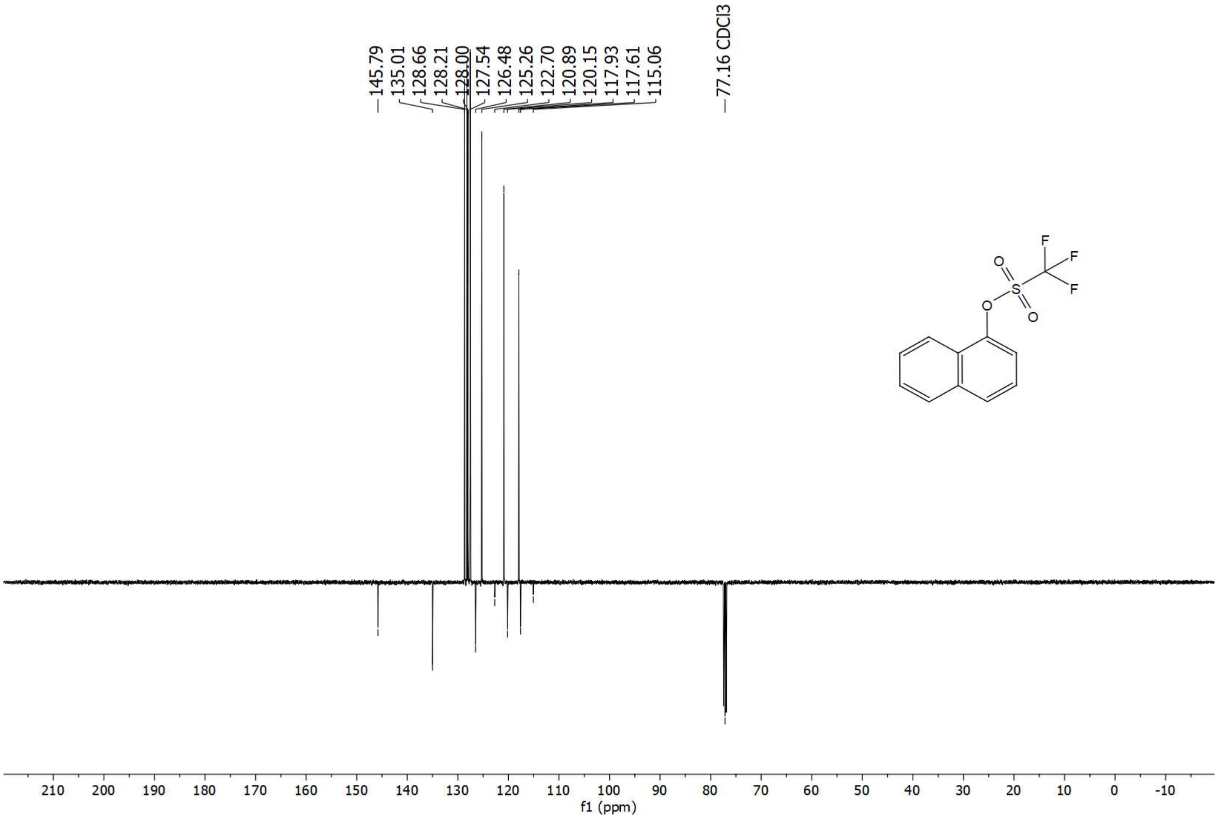

^{1}H NMR (500 MHz, Chloroform-*d*)

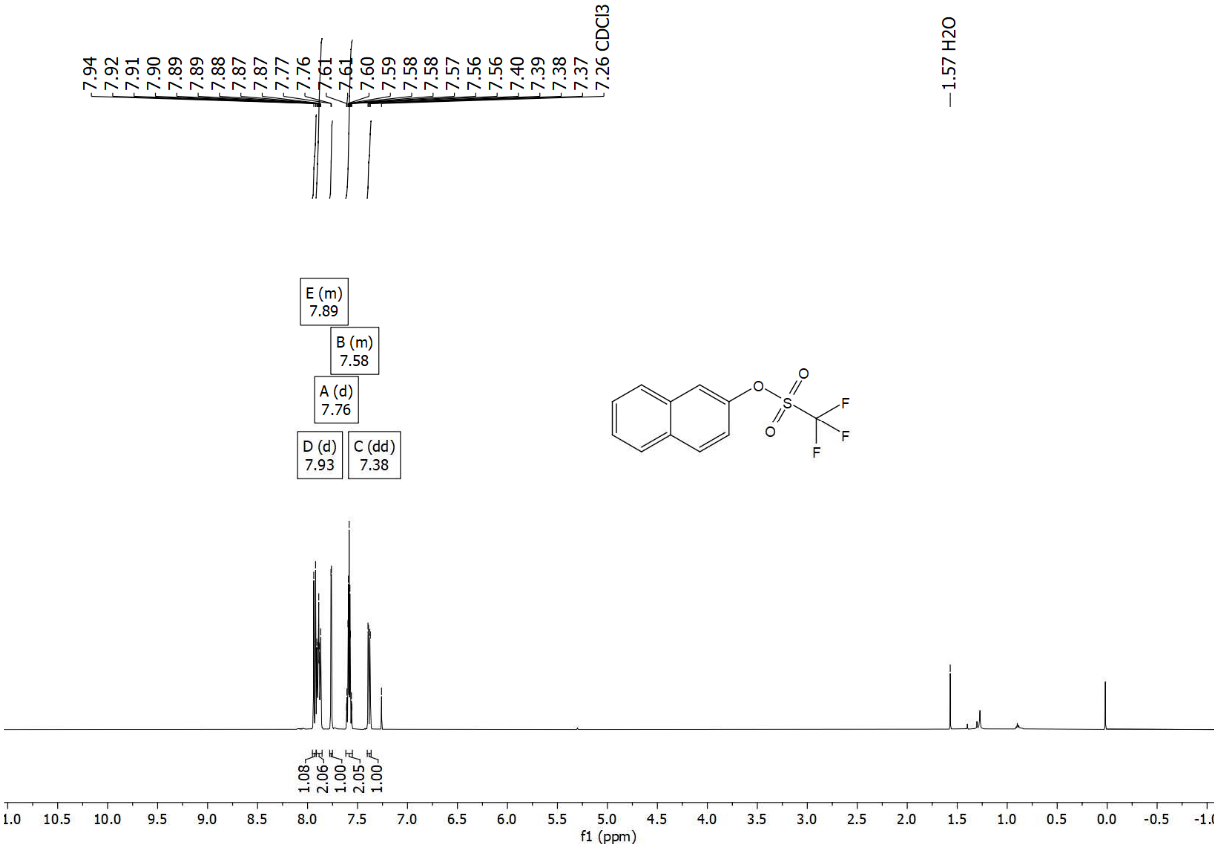

$^{13}C\{1H\}$NMR (126 MHz, $CDCl_3$)

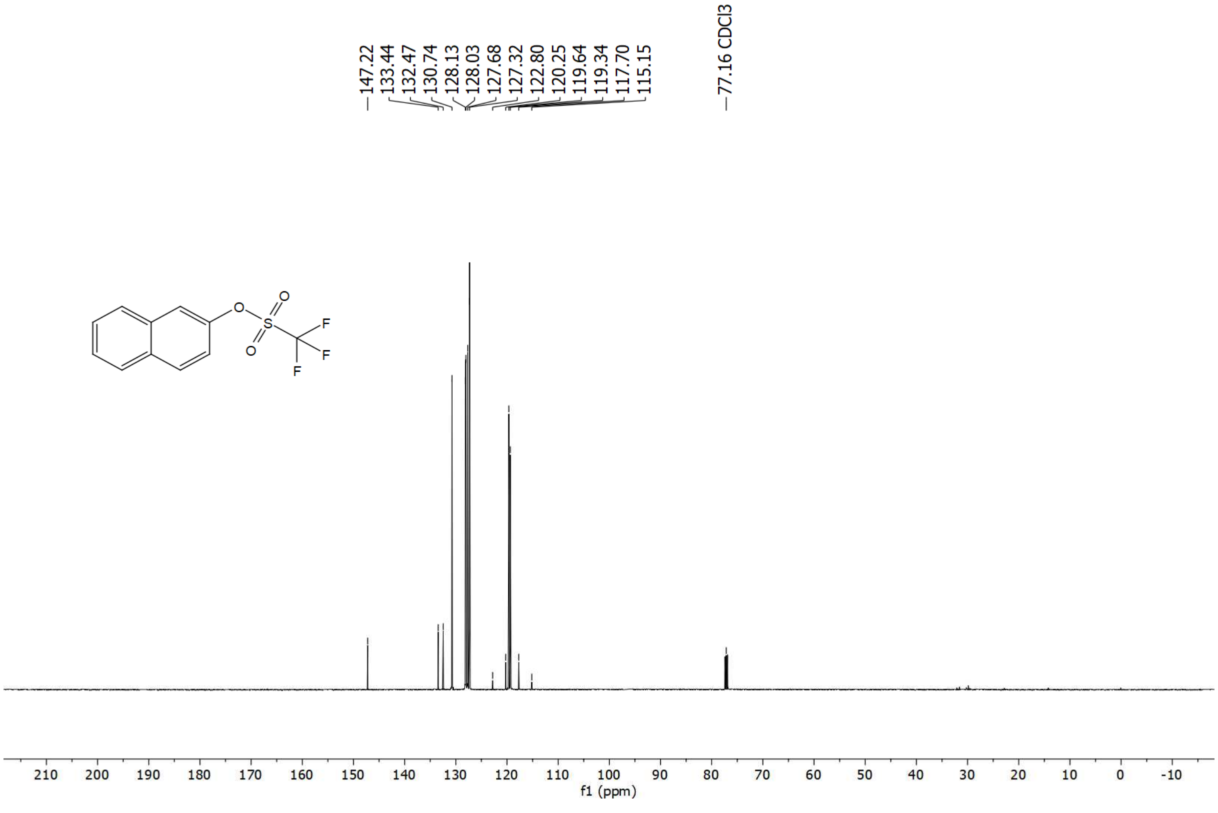

^{1}H NMR (500 MHz, Chloroform-*d*) of **3a**

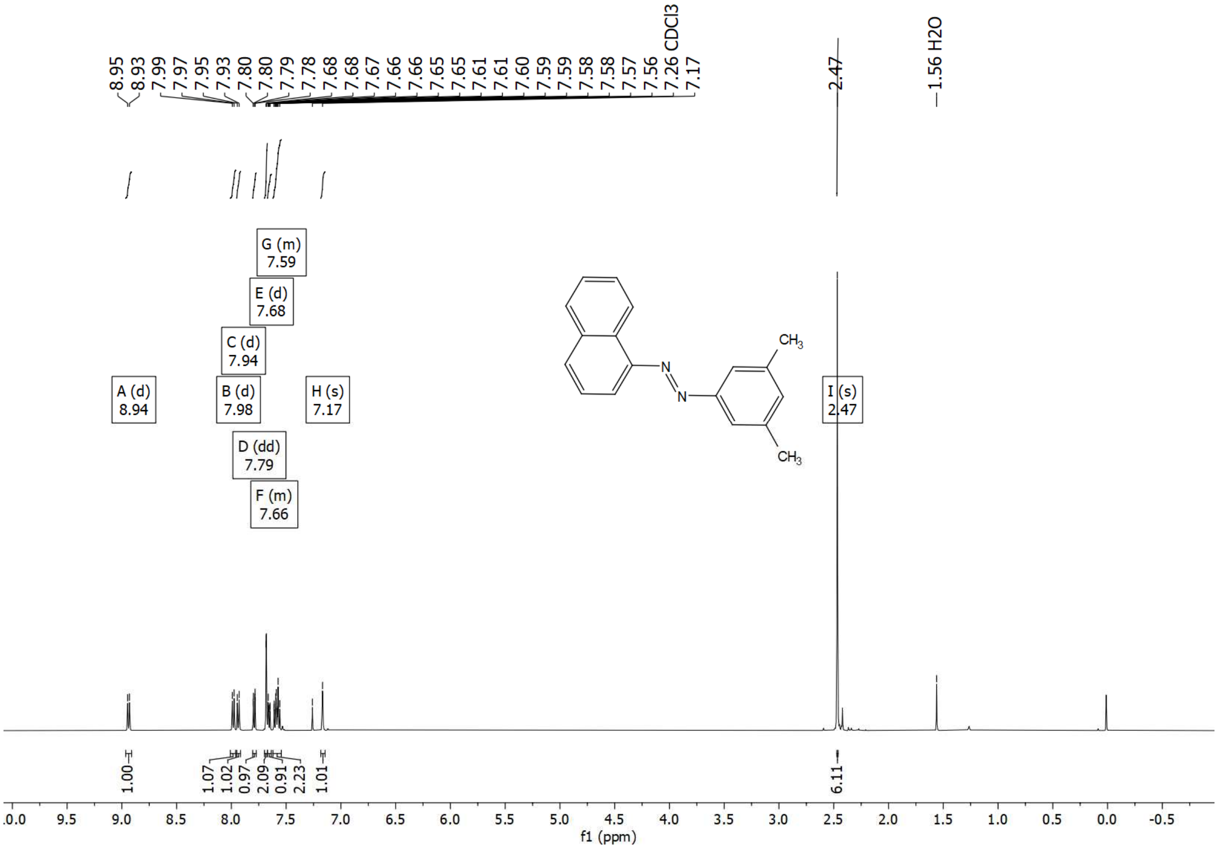

^{13}C{1H}NMR (126 MHz, $CDCl_3$) of **3a**

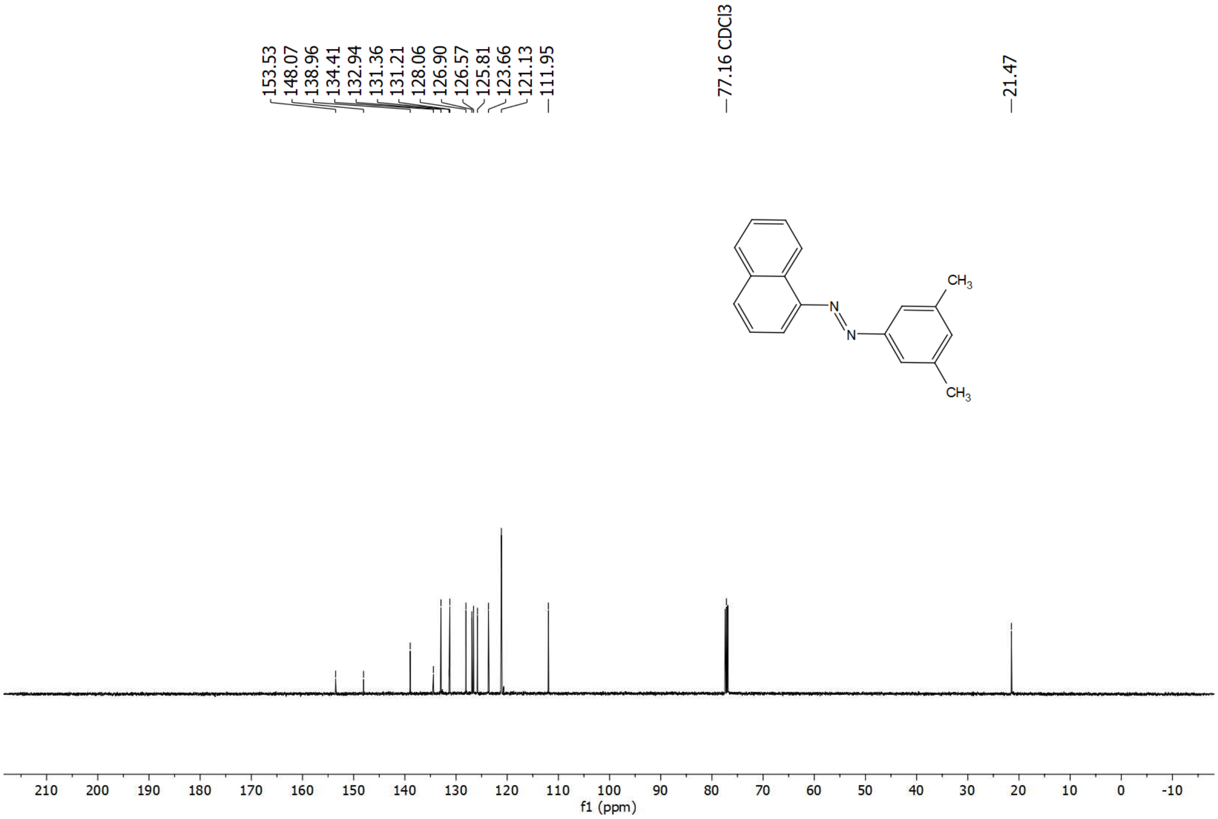

FT-IR diamond (ATR) of **3a**

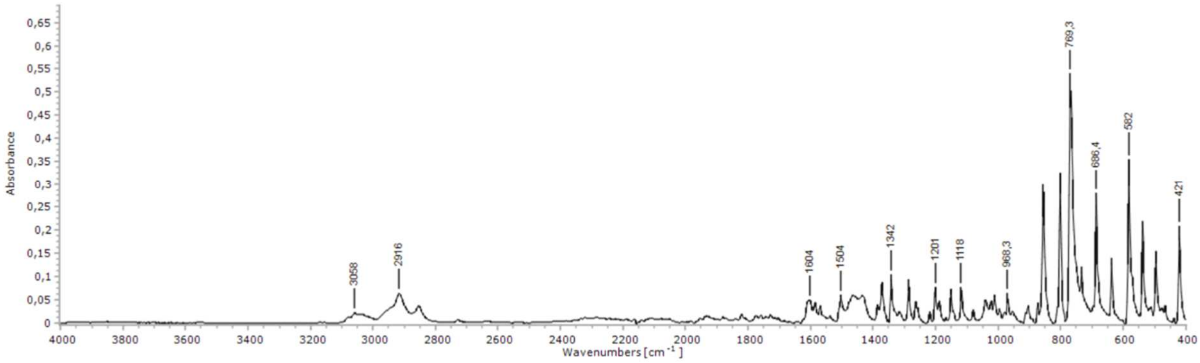

^{1}H NMR (500 MHz, Chloroform-*d*) of **3b**

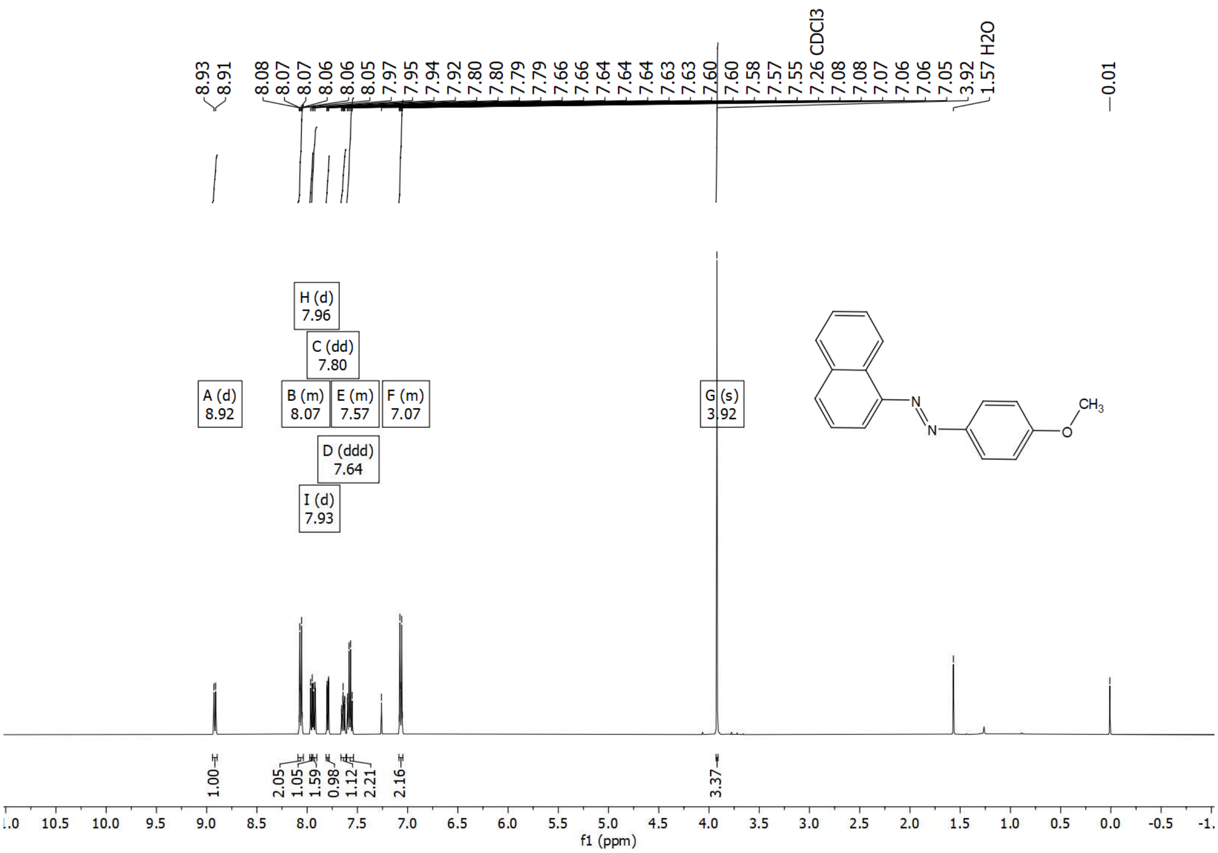

^{13}C{1H}NMR (126 MHz, $CDCl_3$) of **3b**

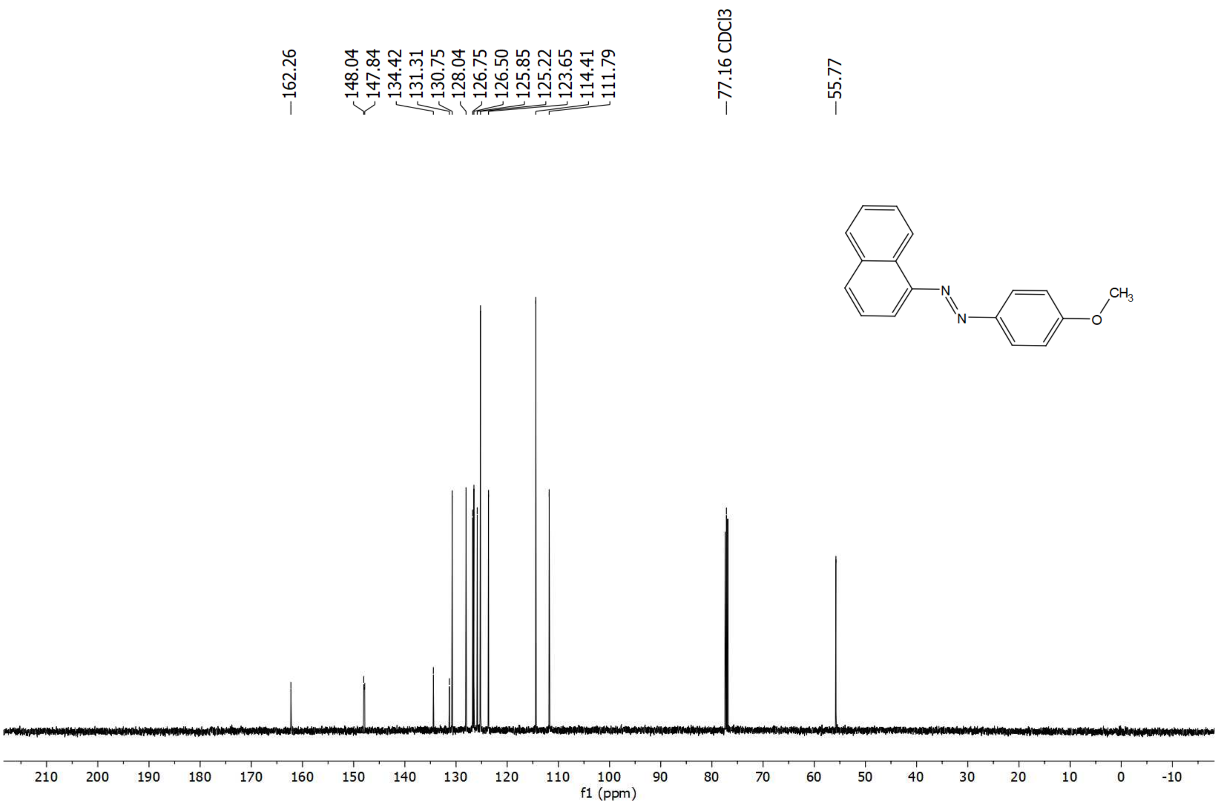

FT-IR diamond (ATR) of **3b**

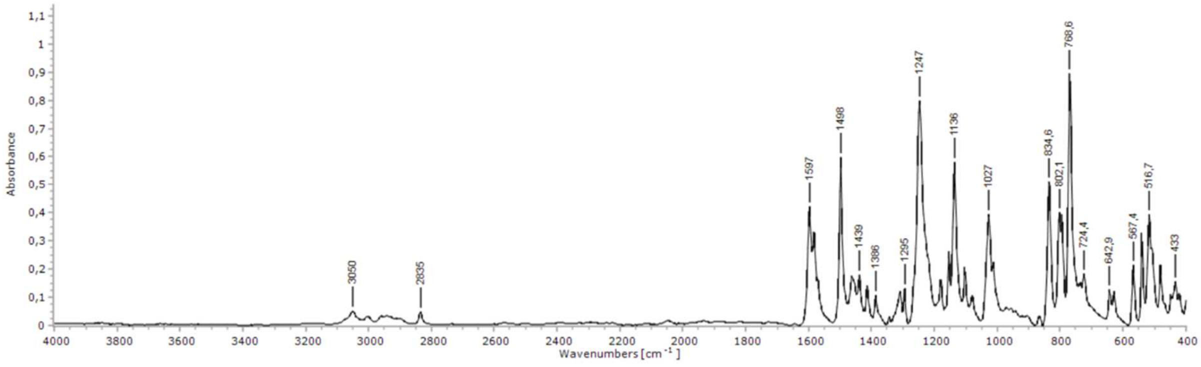

^{1}H NMR (500 MHz, Chloroform-*d*) of **3c**

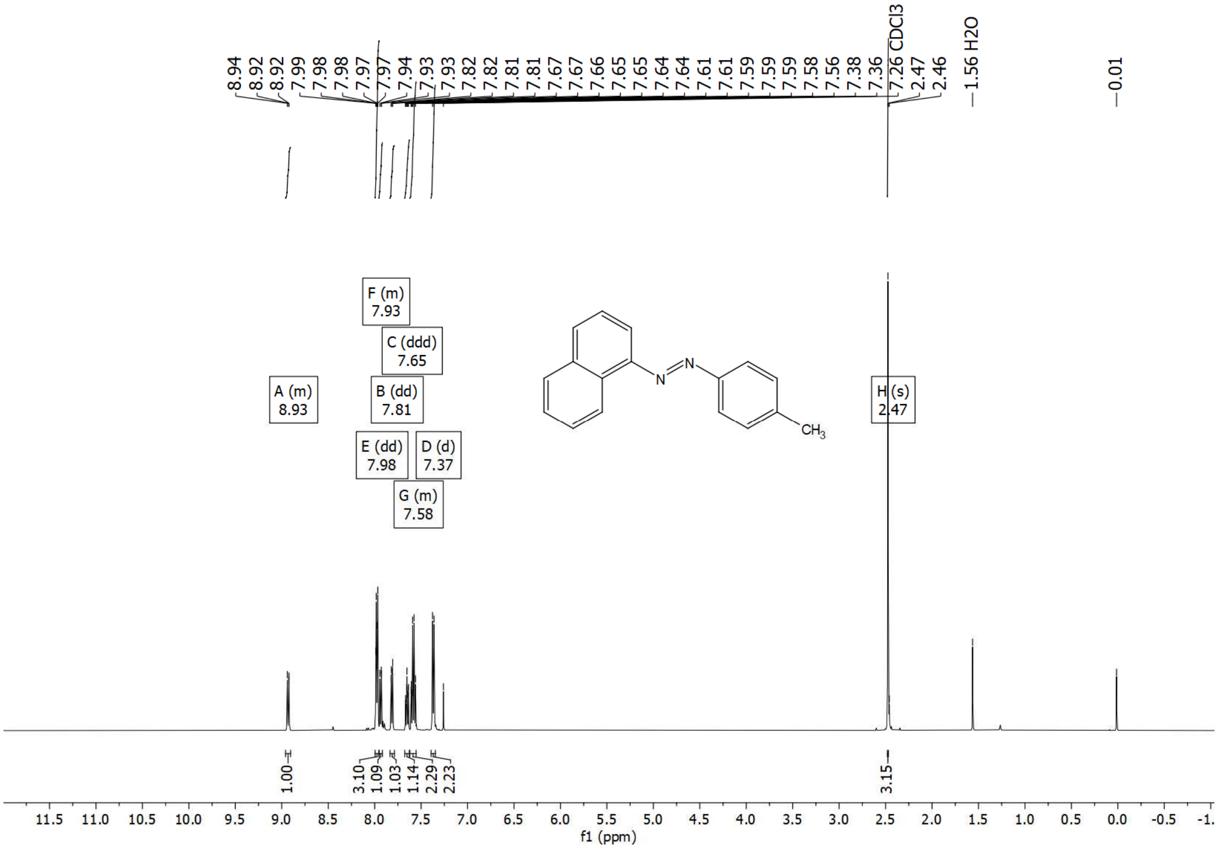

^{13}C APT (126 MHz, $CDCl_3$) of **3c**

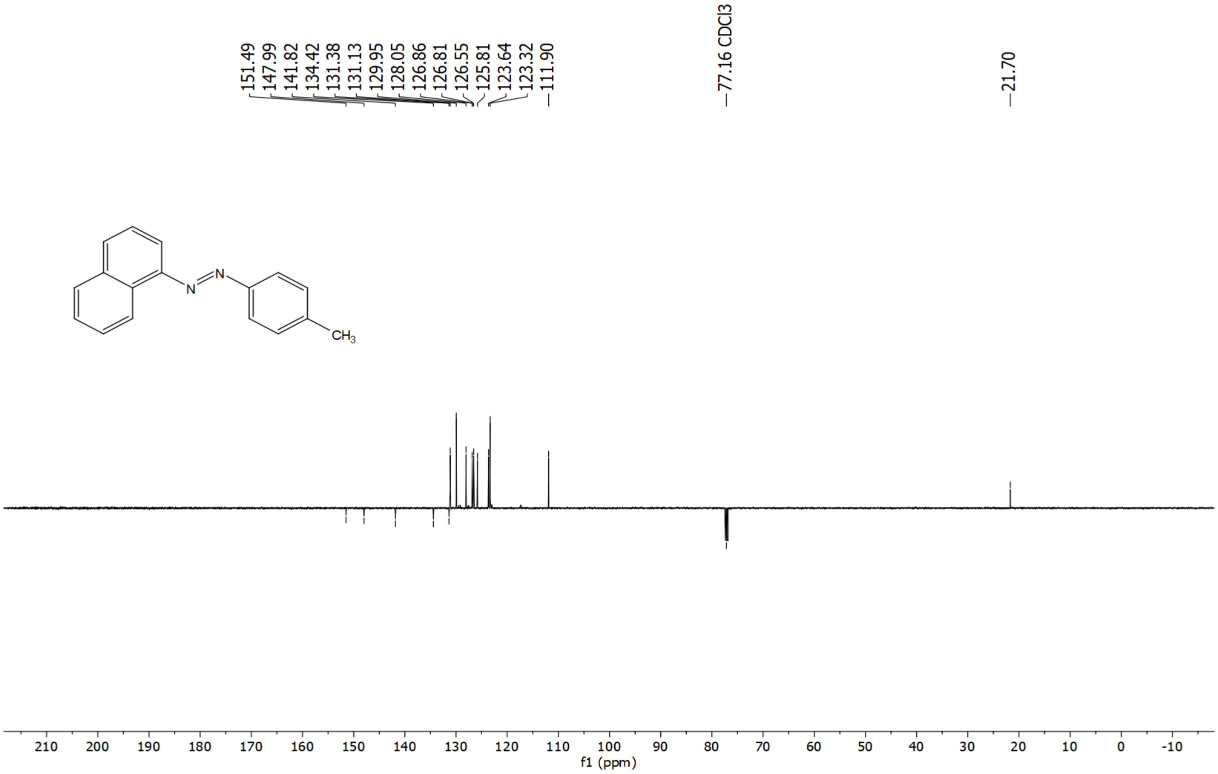

FT-IR diamond (ATR) of **3c**

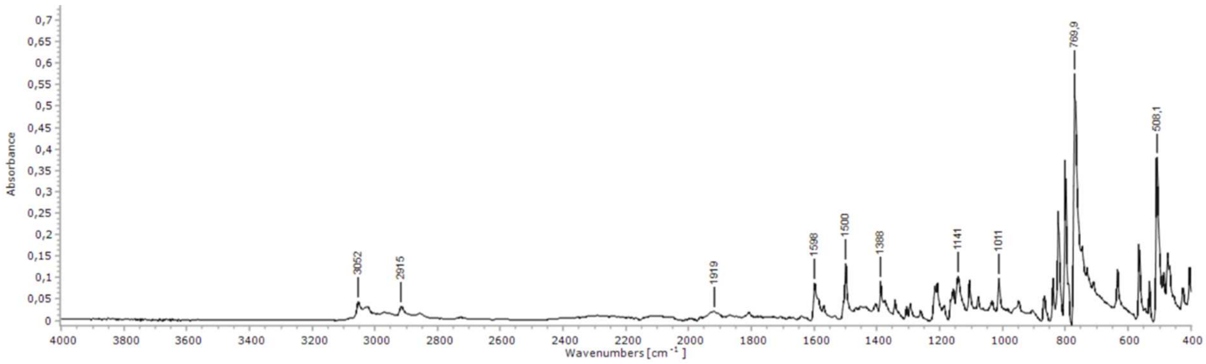

^{1}H NMR (500 MHz, Chloroform-*d*) of **3d**

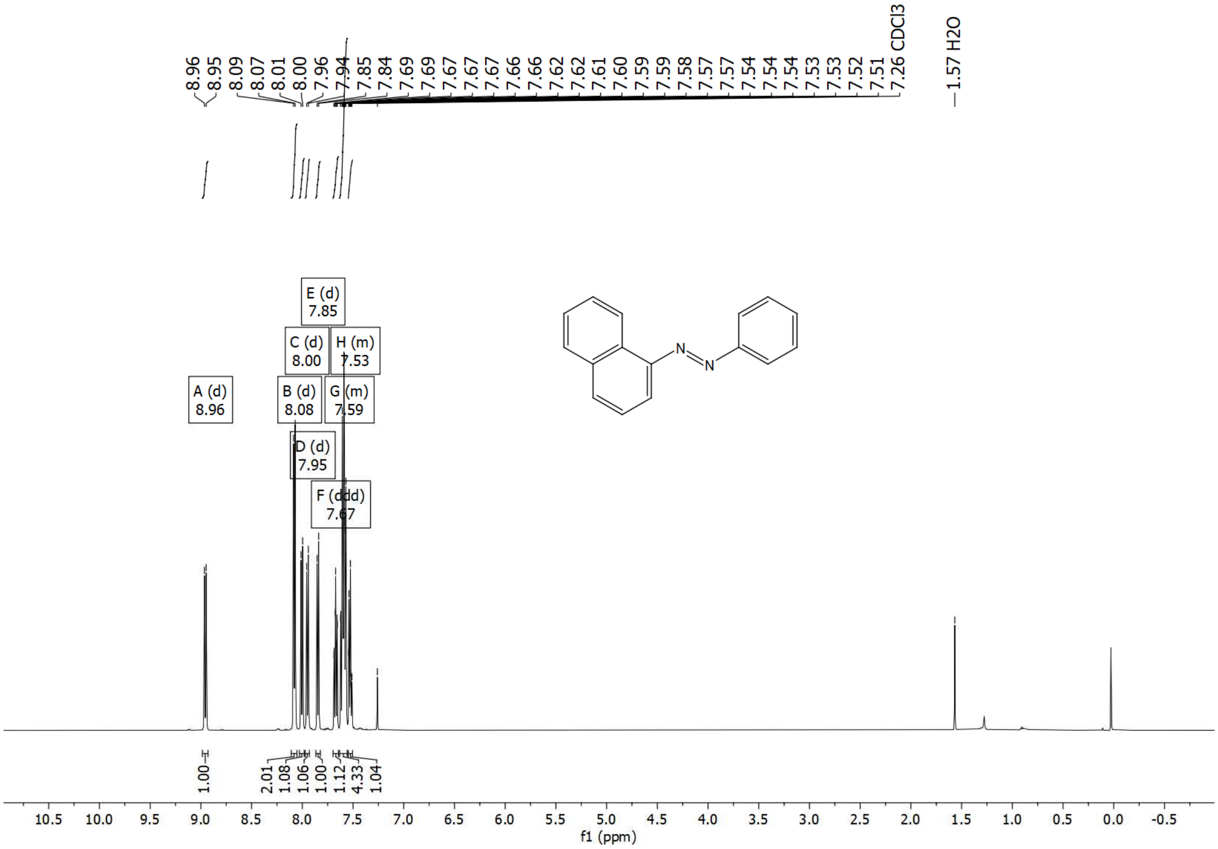

^{13}C APT NMR (126 MHz, $CDCl_3$) of **3d**

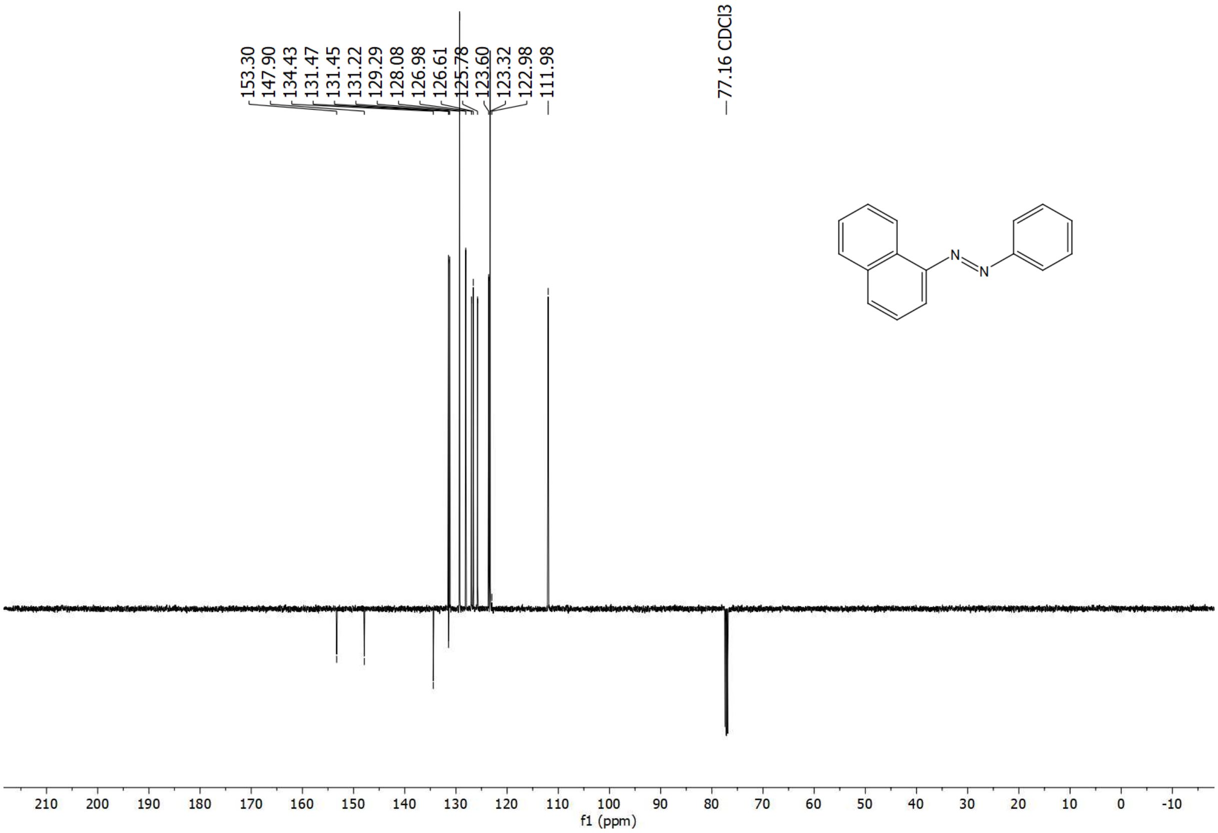

FT-IR diamond (ATR) of **3d**

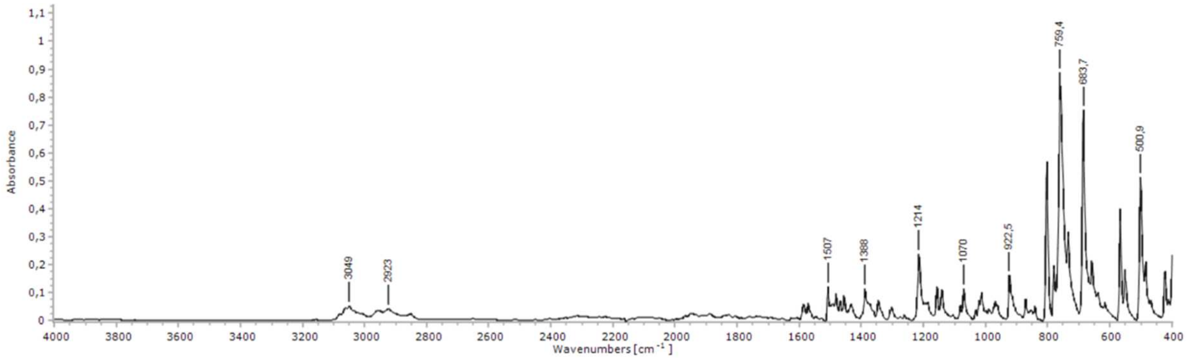

^{1}H NMR (500 MHz, Chloroform-*d*) of **3e**

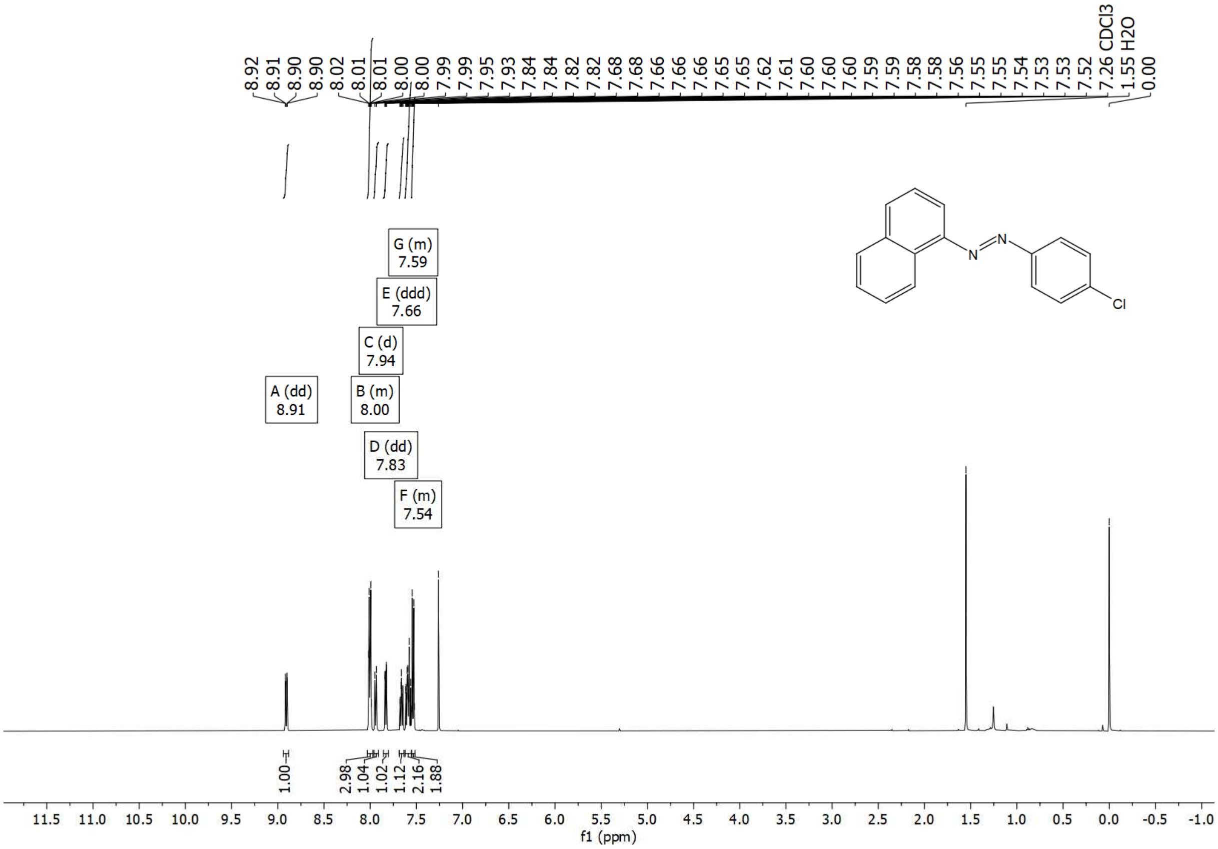

^{13}C APT NMR (126 MHz, $CDCl_3$) of **3e**

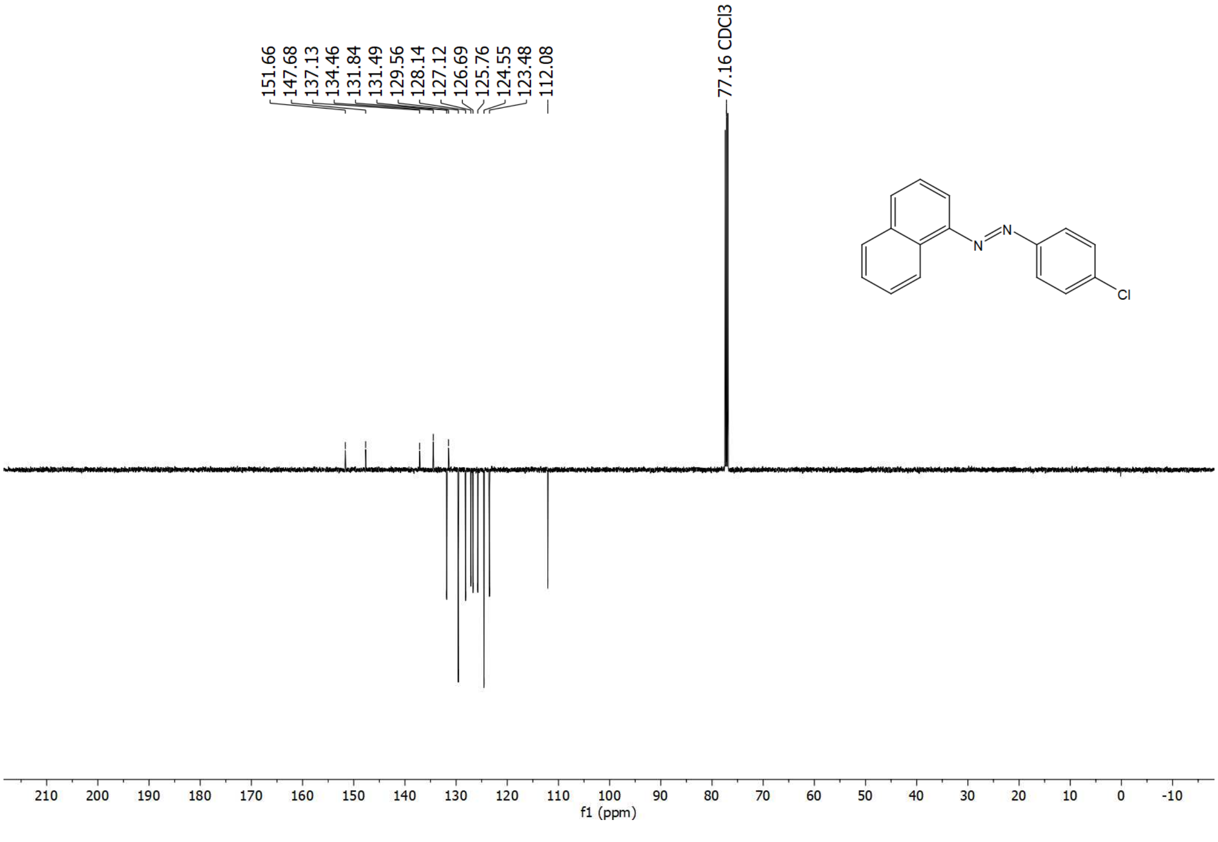

FT-IR diamond (ATR) of **3e**

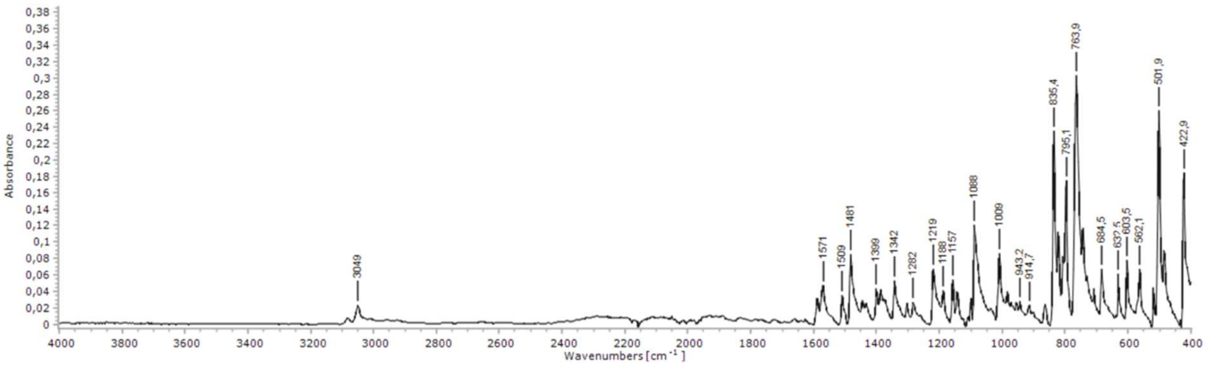

^{1}H NMR (500 MHz, Chloroform-*d*) of **3f**

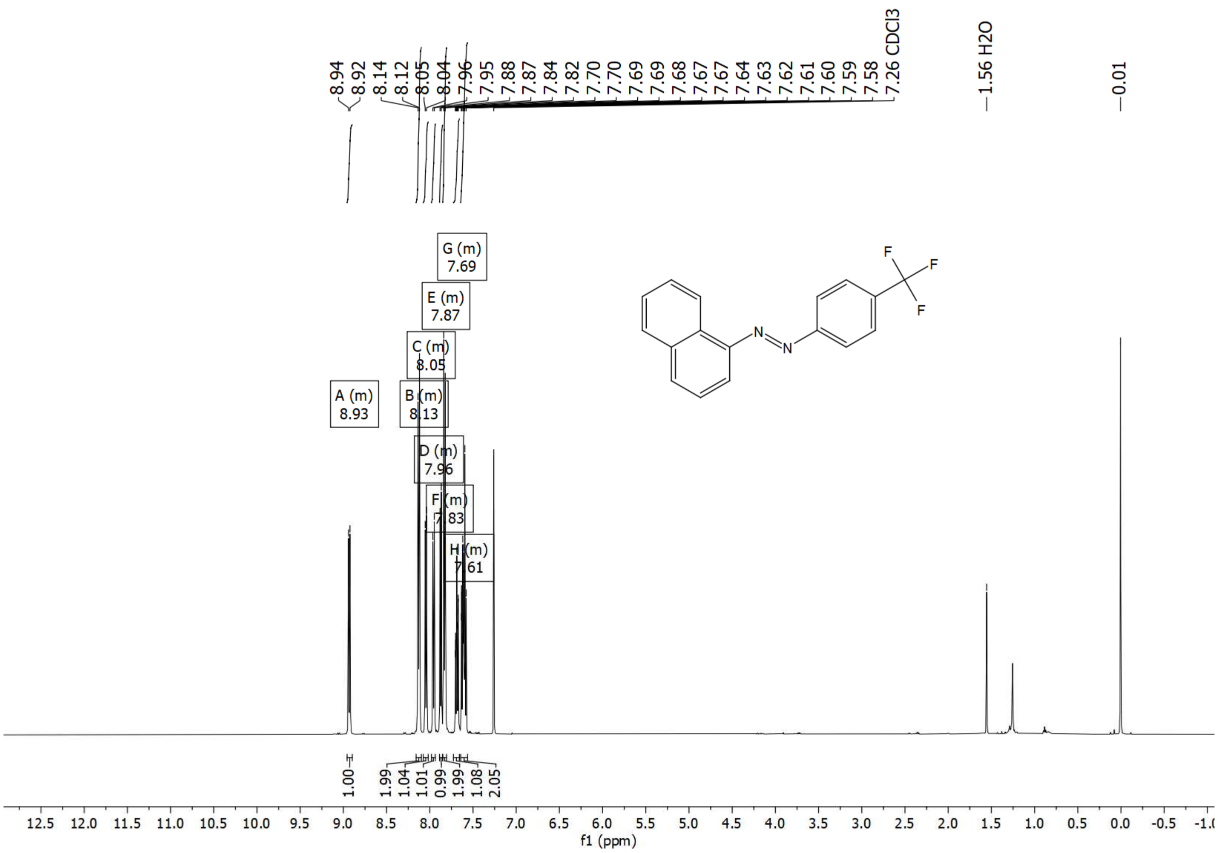

$^{13}C\{1H\}$NMR (126 MHz, $CDCl_3$) of **3f**

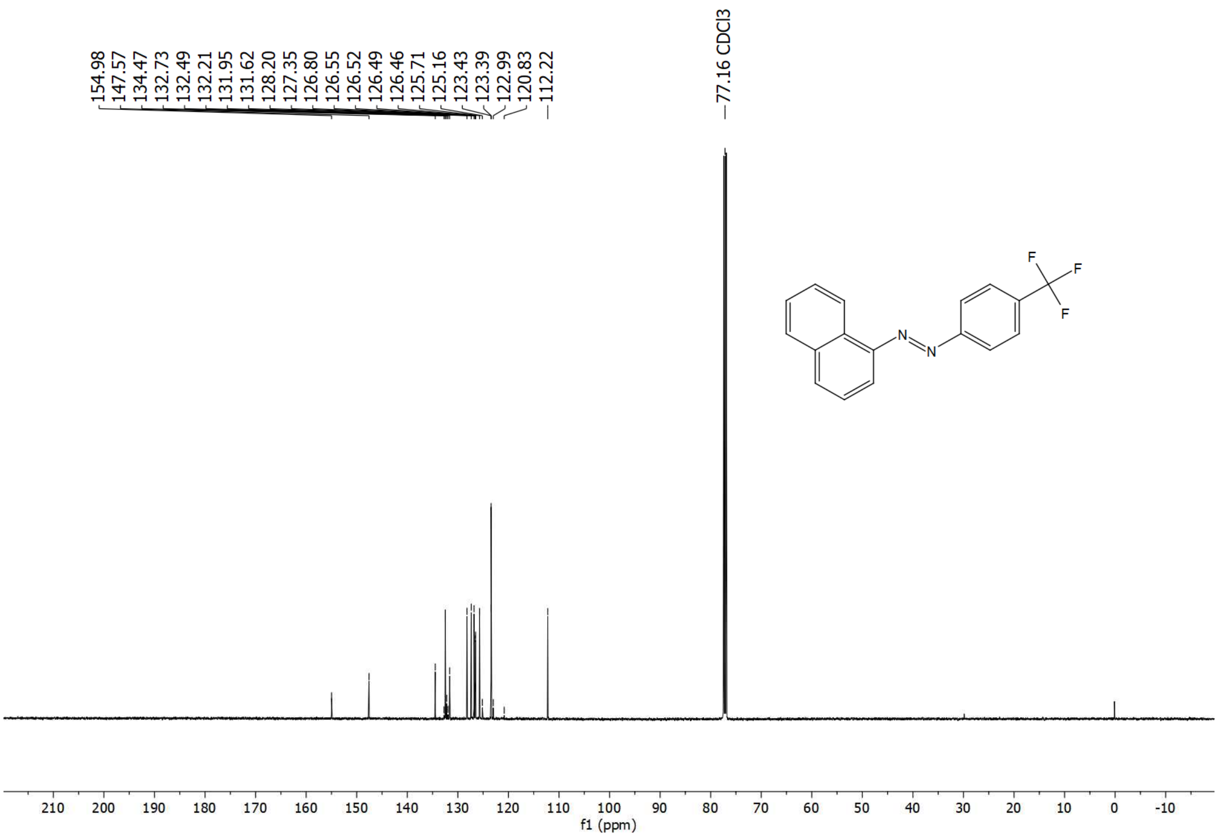

$^{19}F\{H\}$ NMR (376 MHz, $CDCl_3$) of **3f**

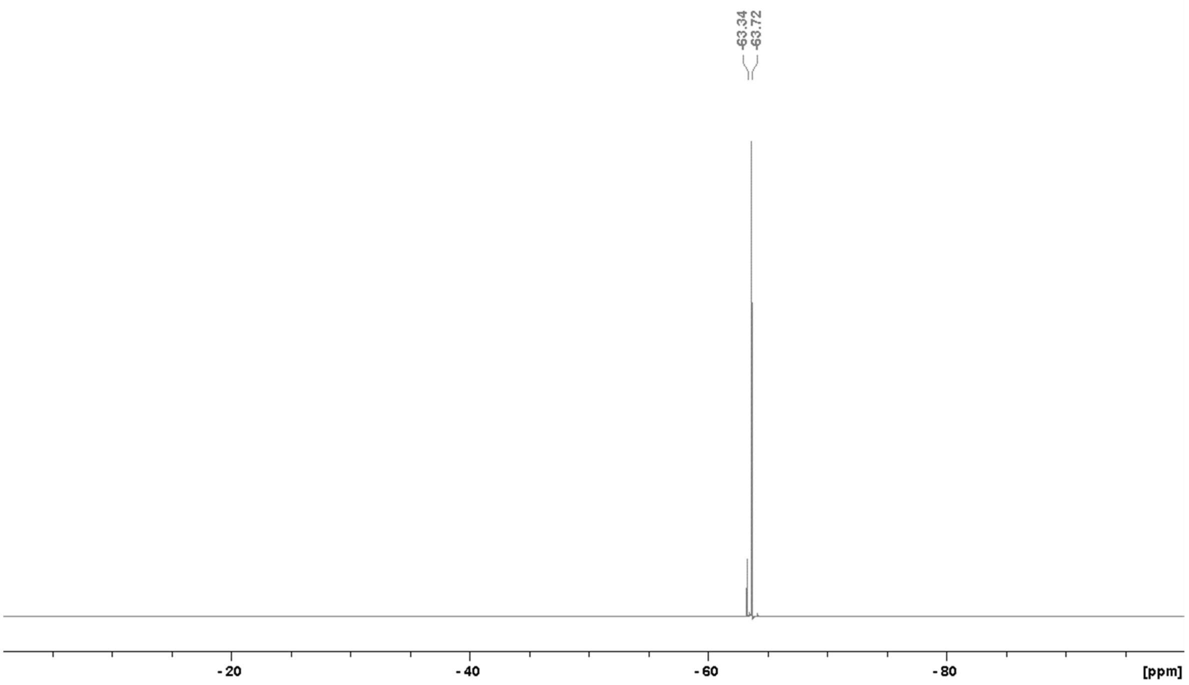

FT-IR diamond (ATR) of **3f**

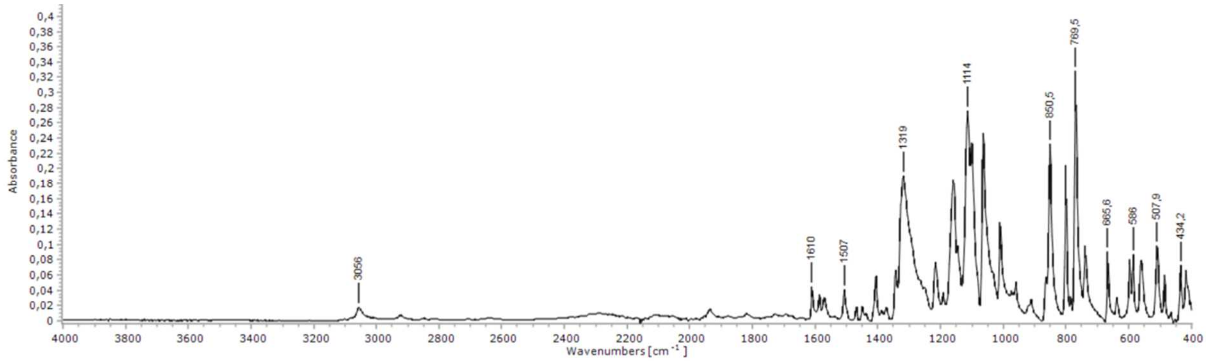

^{1}H NMR (500 MHz, Chloroform-*d*) of **3g**

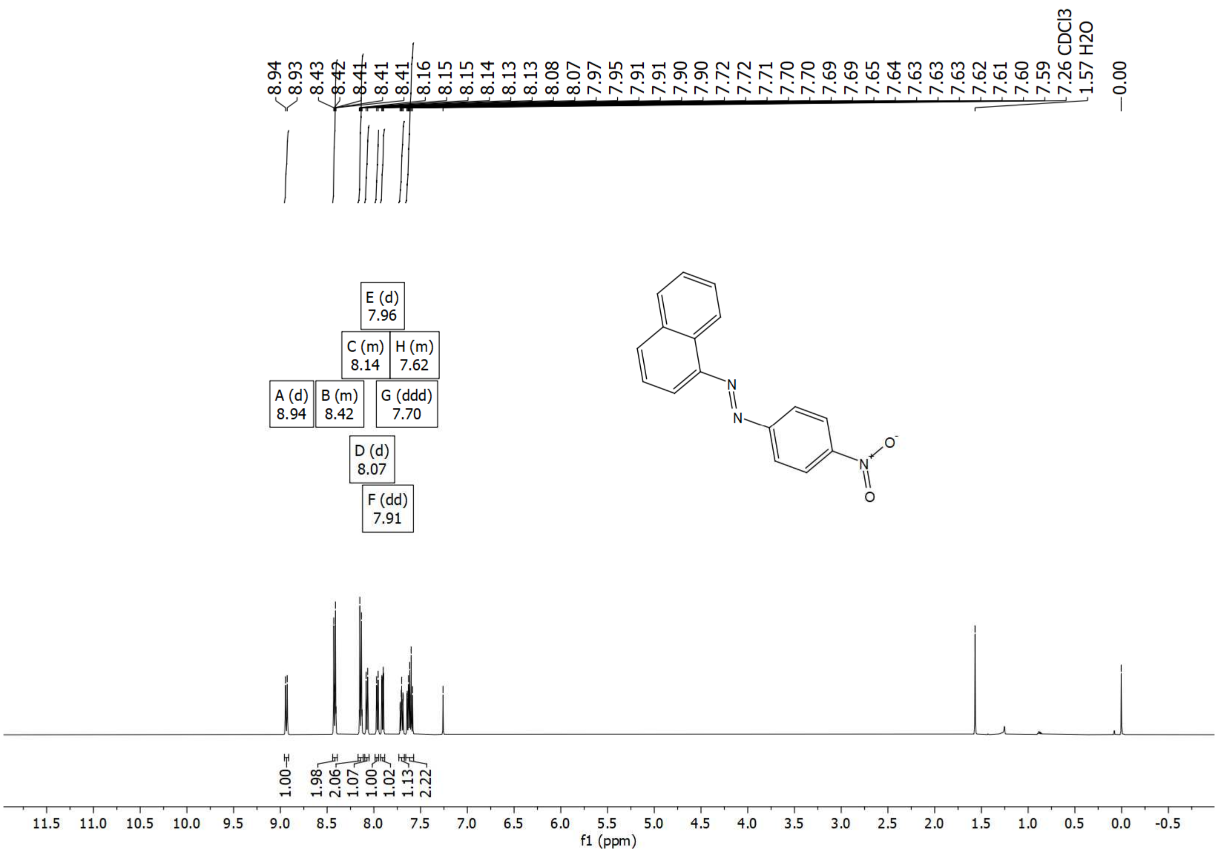

^{13}C APT NMR (126 MHz, $CDCl_3$) of **3g**

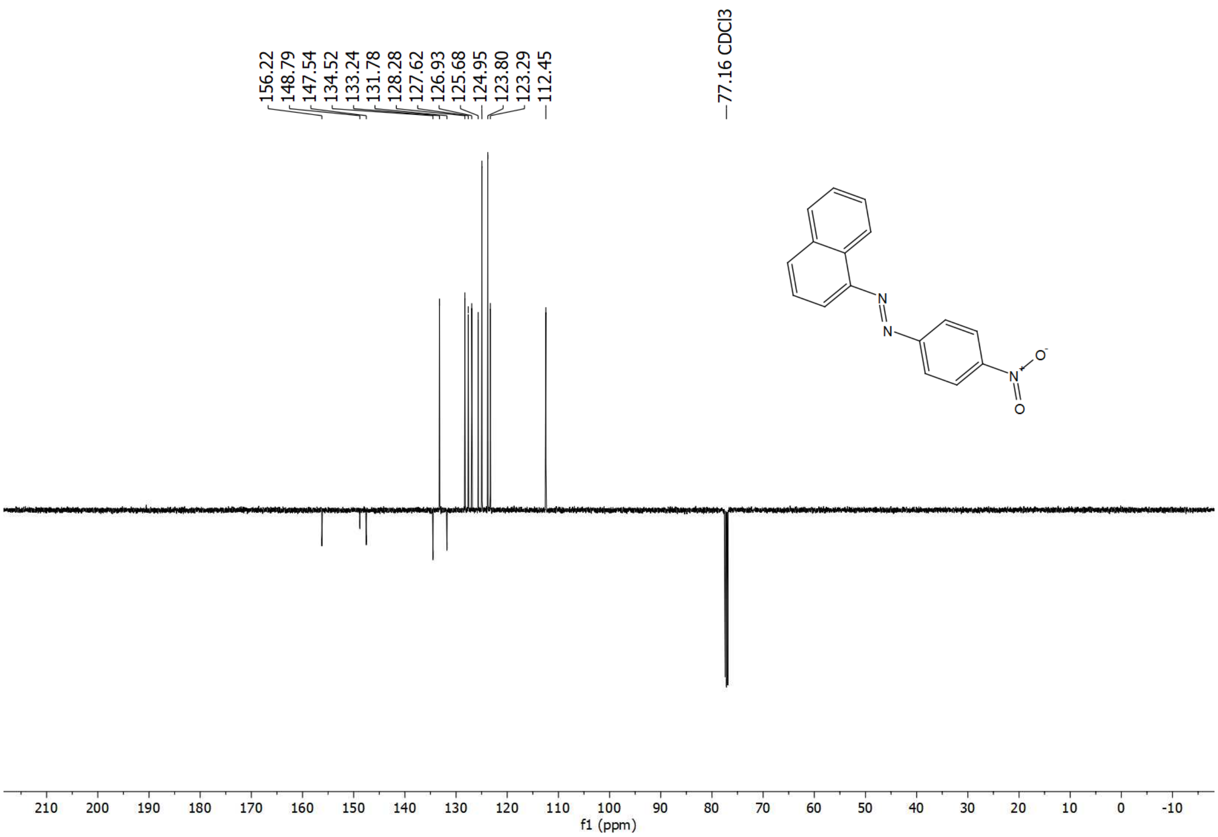

FT-IR diamond (ATR) of **3g**

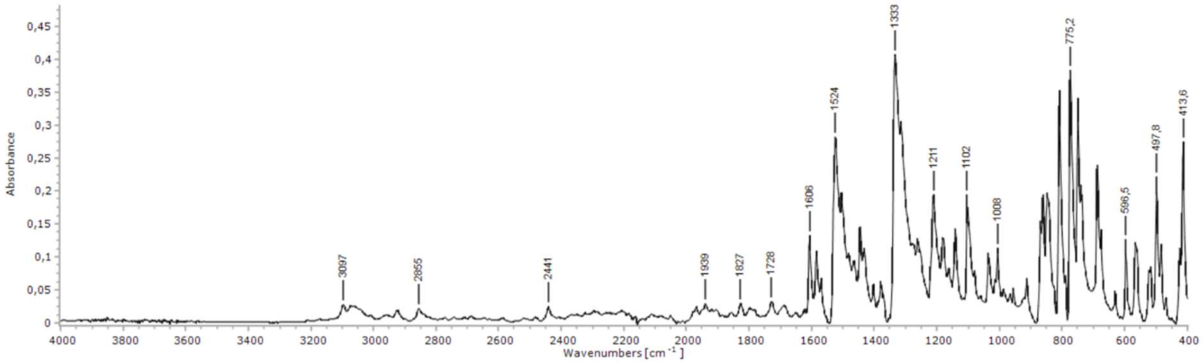

^{1}H NMR (500 MHz, Chloroform-*d*) of **3h**

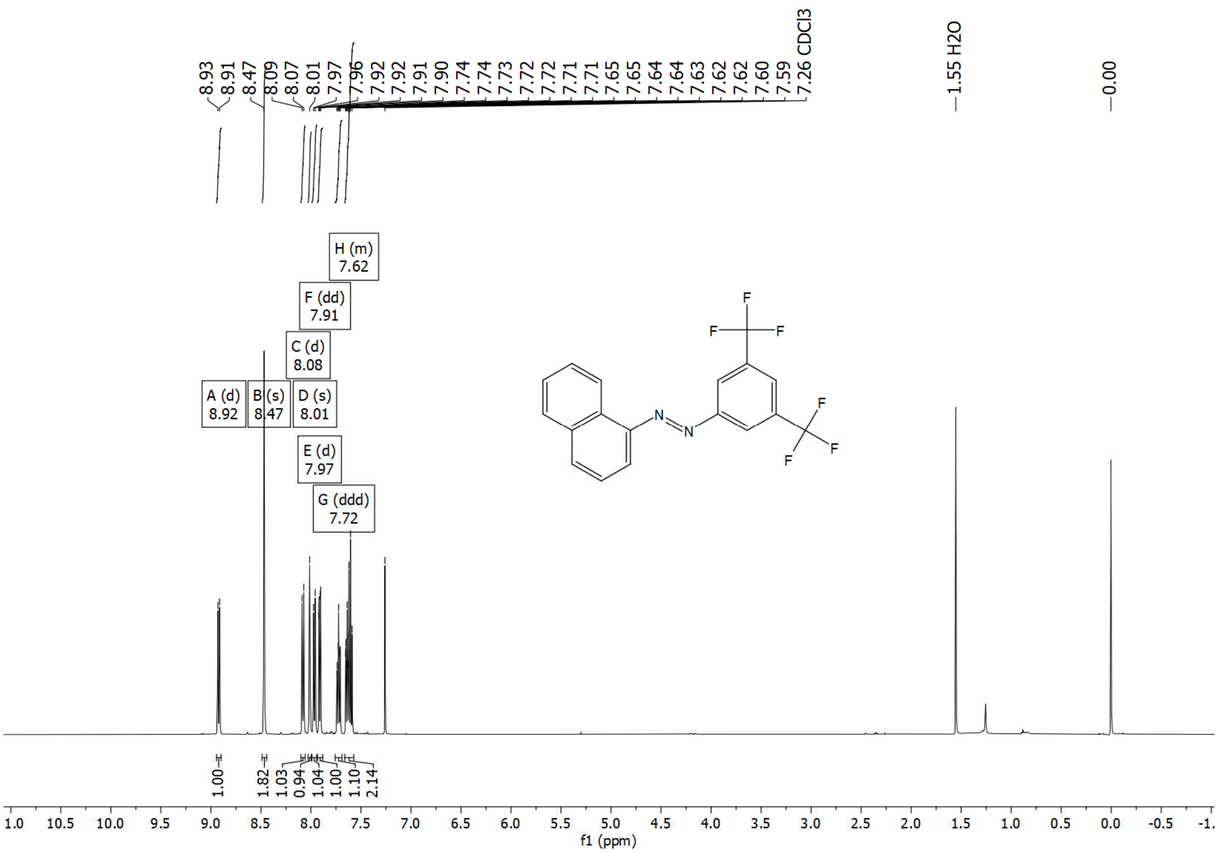

^{13}C APT NMR (126 MHz, $CDCl_3$) of **3h**

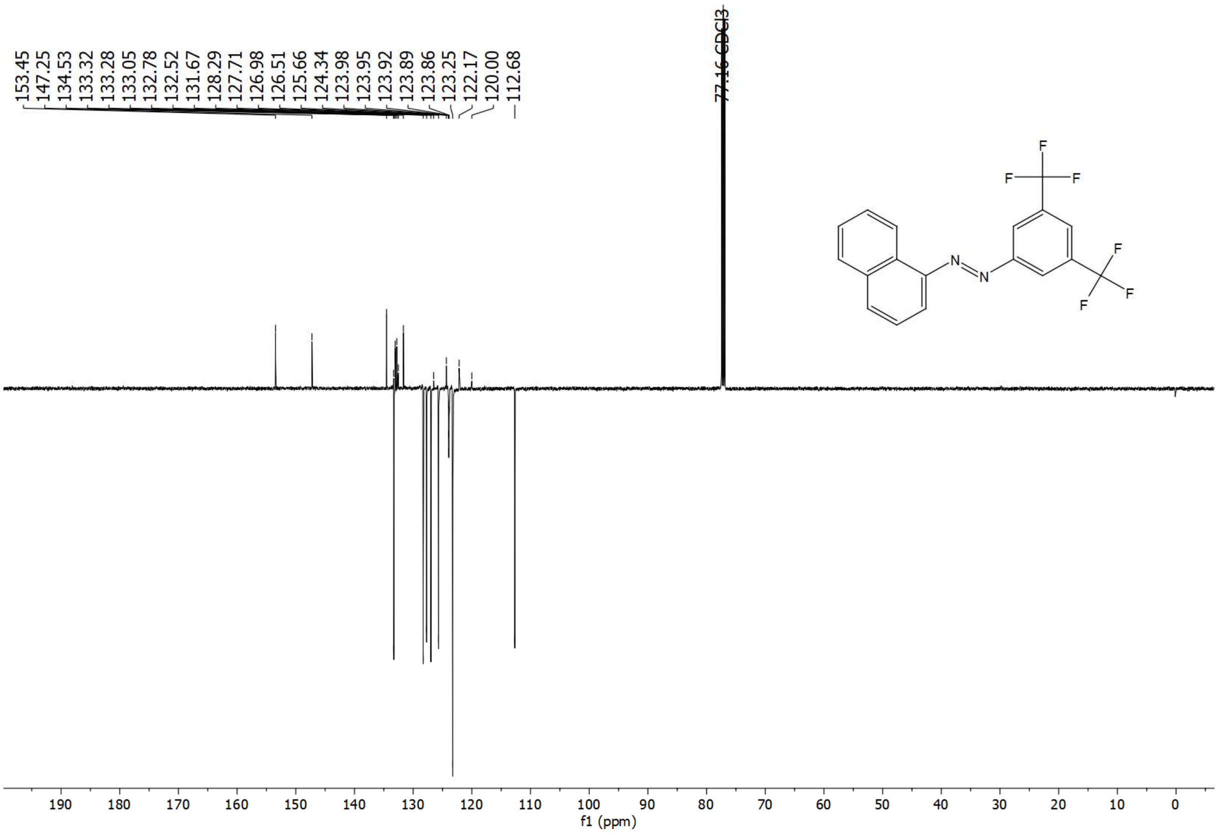

$^{19}F\{H\}$ NMR (376 MHz, $CDCl_3$) of **3h**

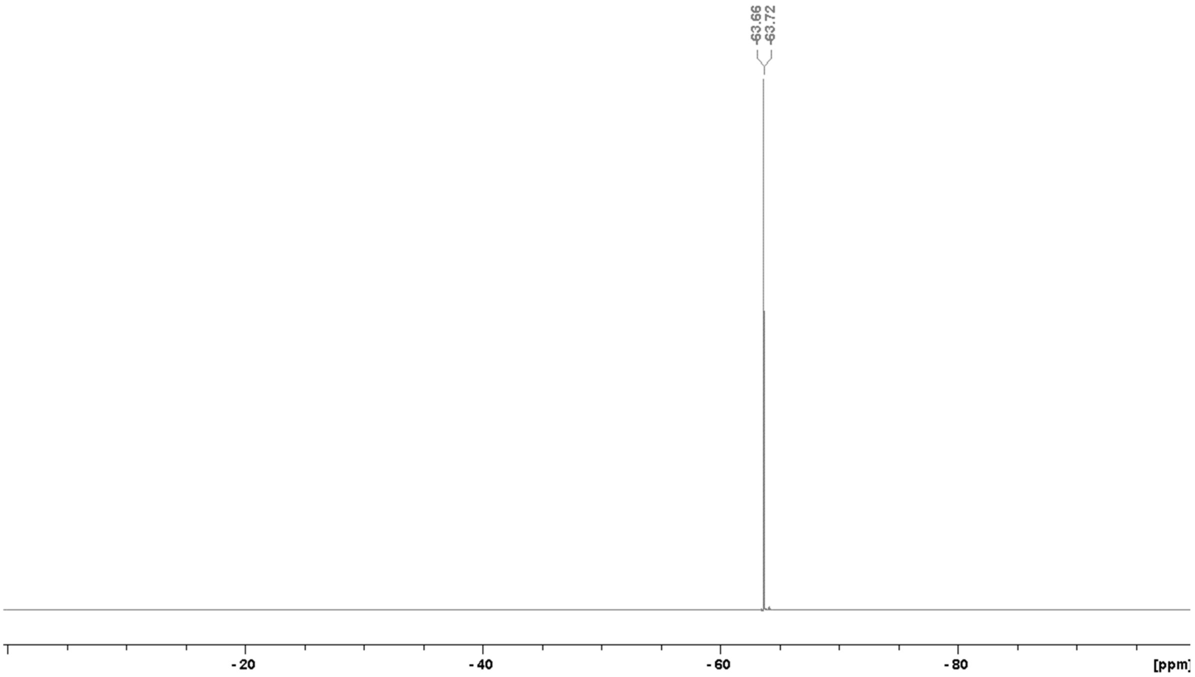

FT-IR diamond (ATR) of **3h**

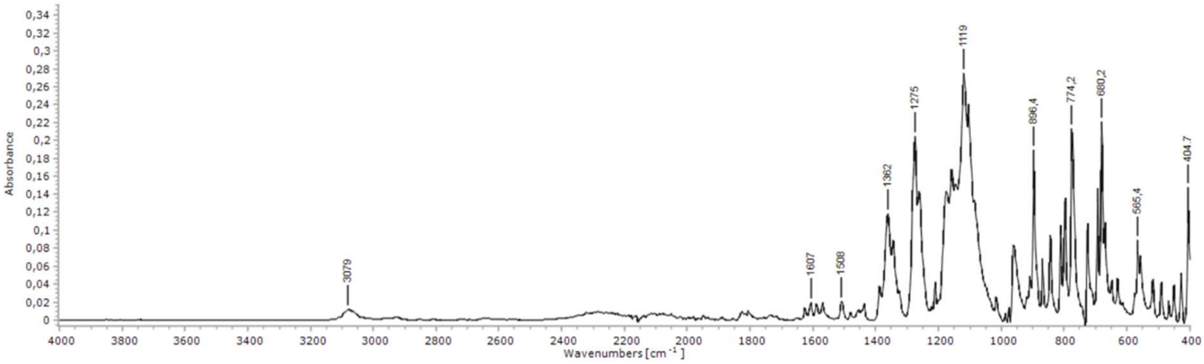

^{1}H NMR (500 MHz, Chloroform-*d*) of **3i**

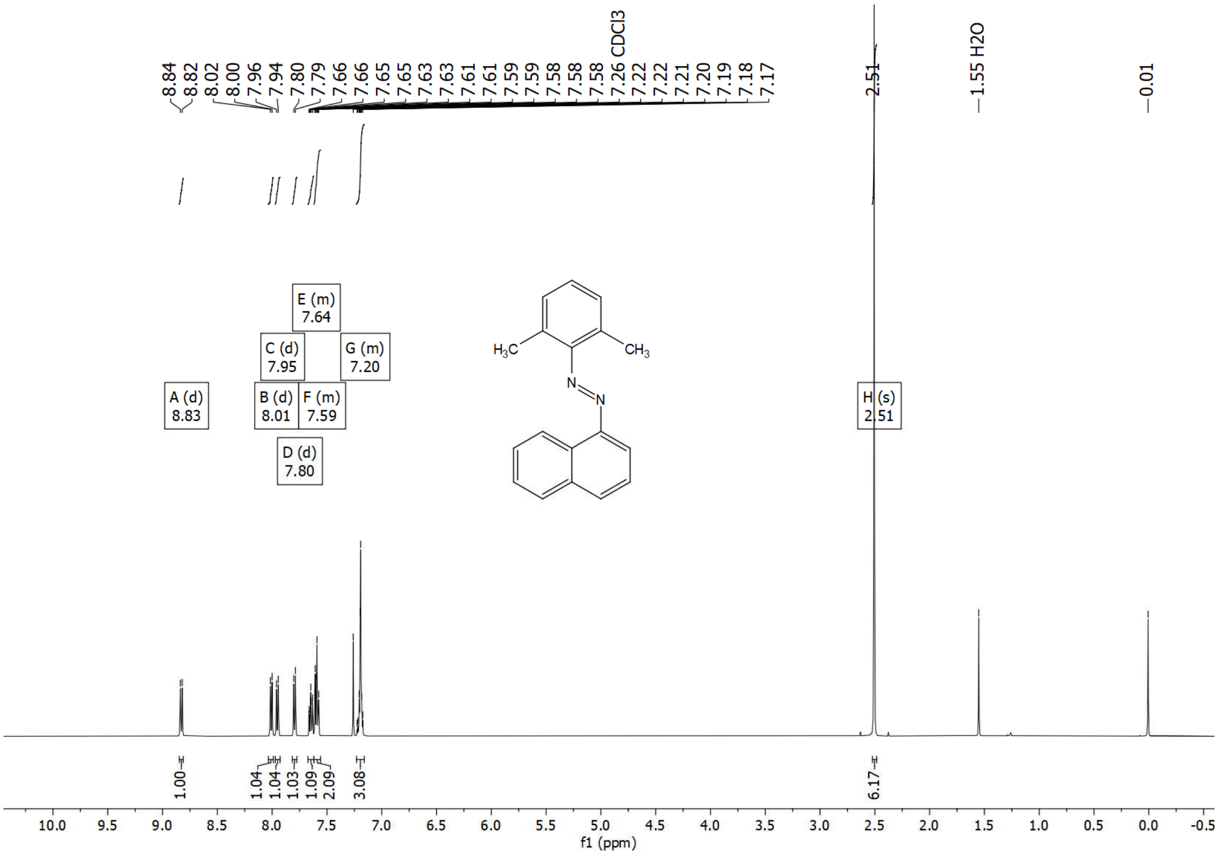

^{13}C APT NMR (126 MHz, $CDCl_3$) of **3i**

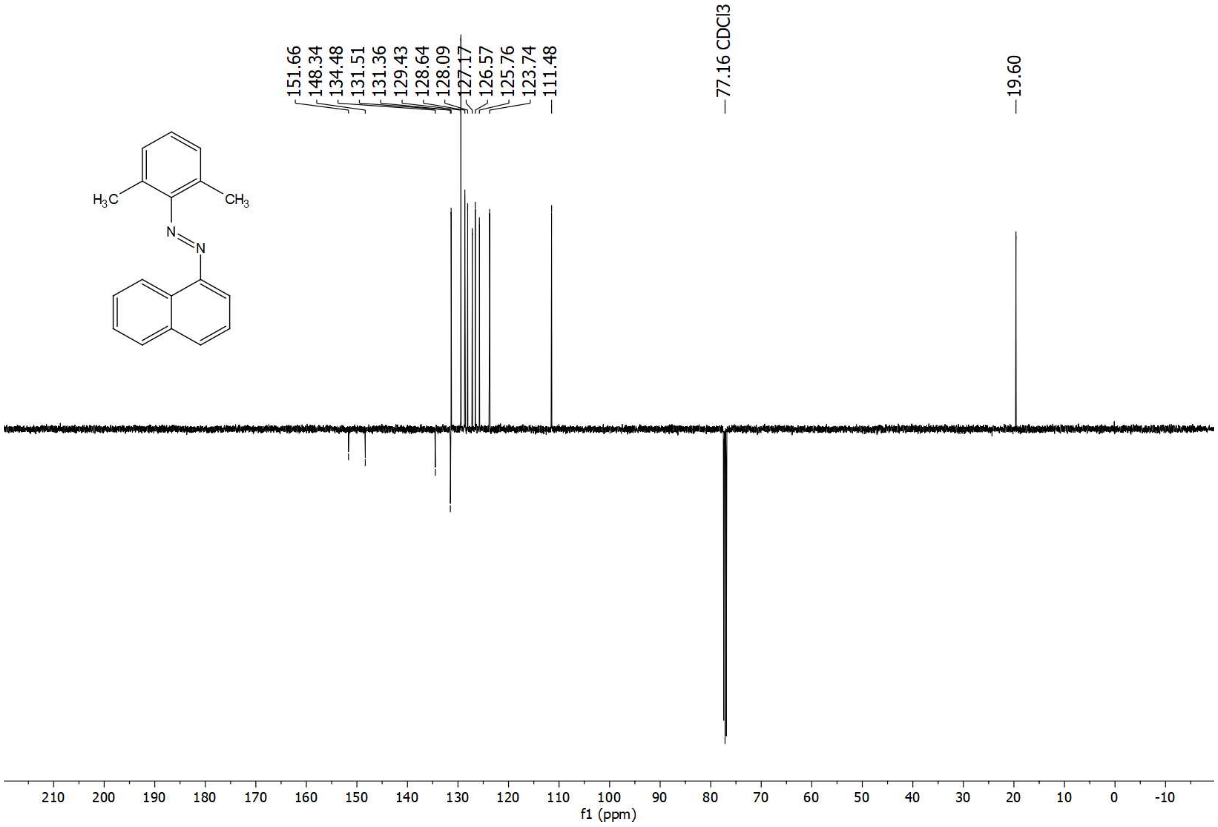

FT-IR (ATR) diamond of **3i**

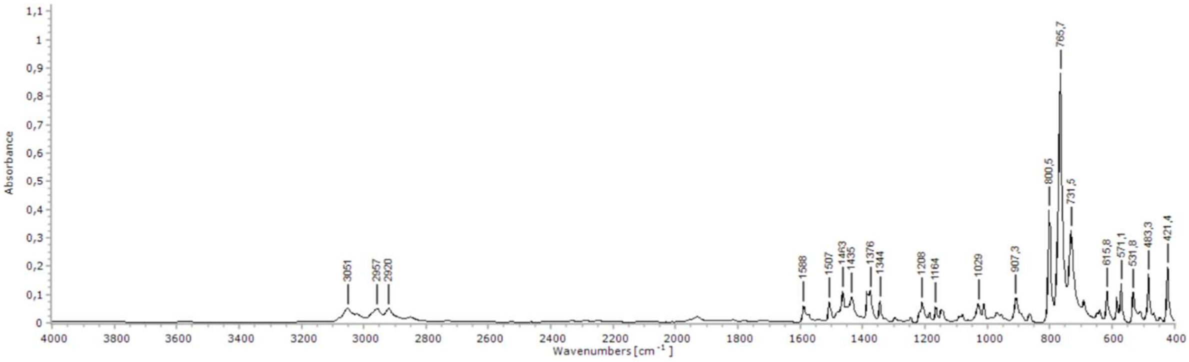

^{1}H NMR (500 MHz, Chloroform-*d*) of **3j**

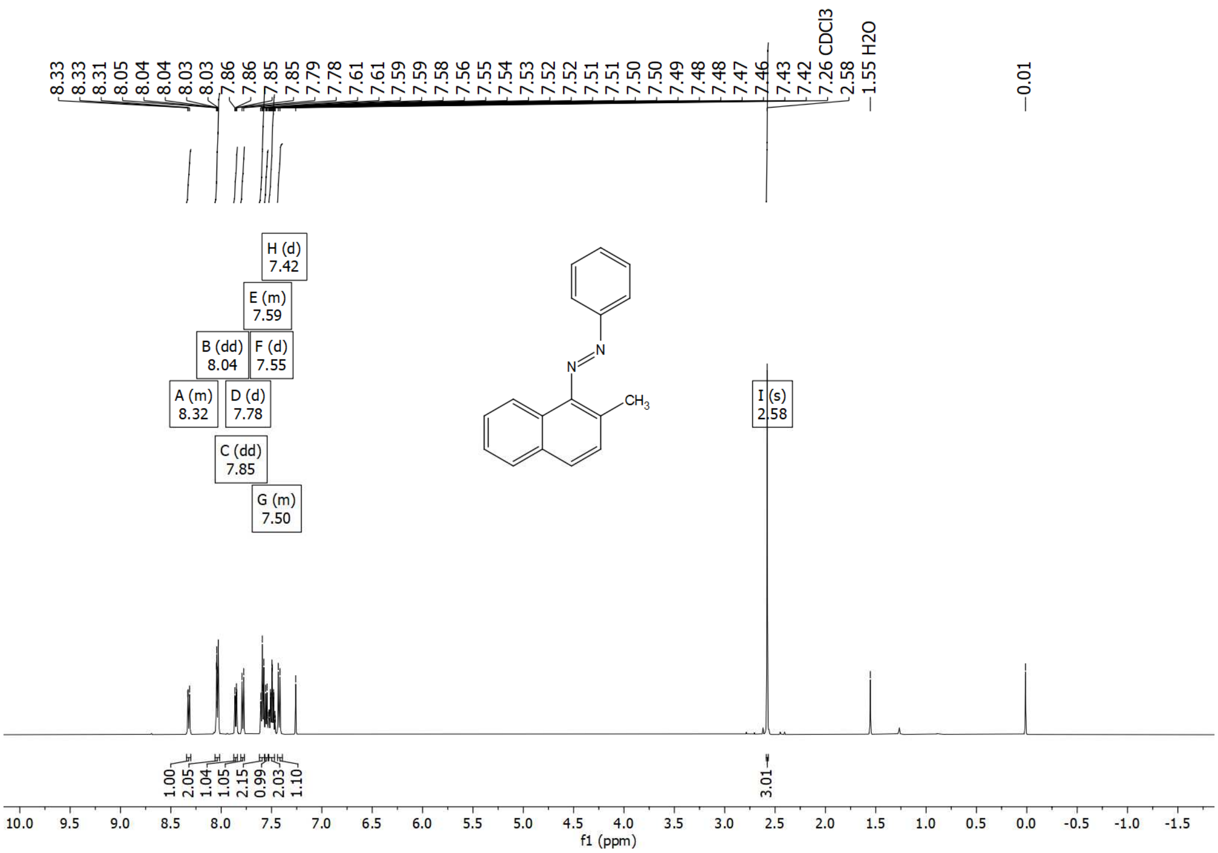

^{13}C APT NMR (126 MHz, $CDCl_3$) of **3j**

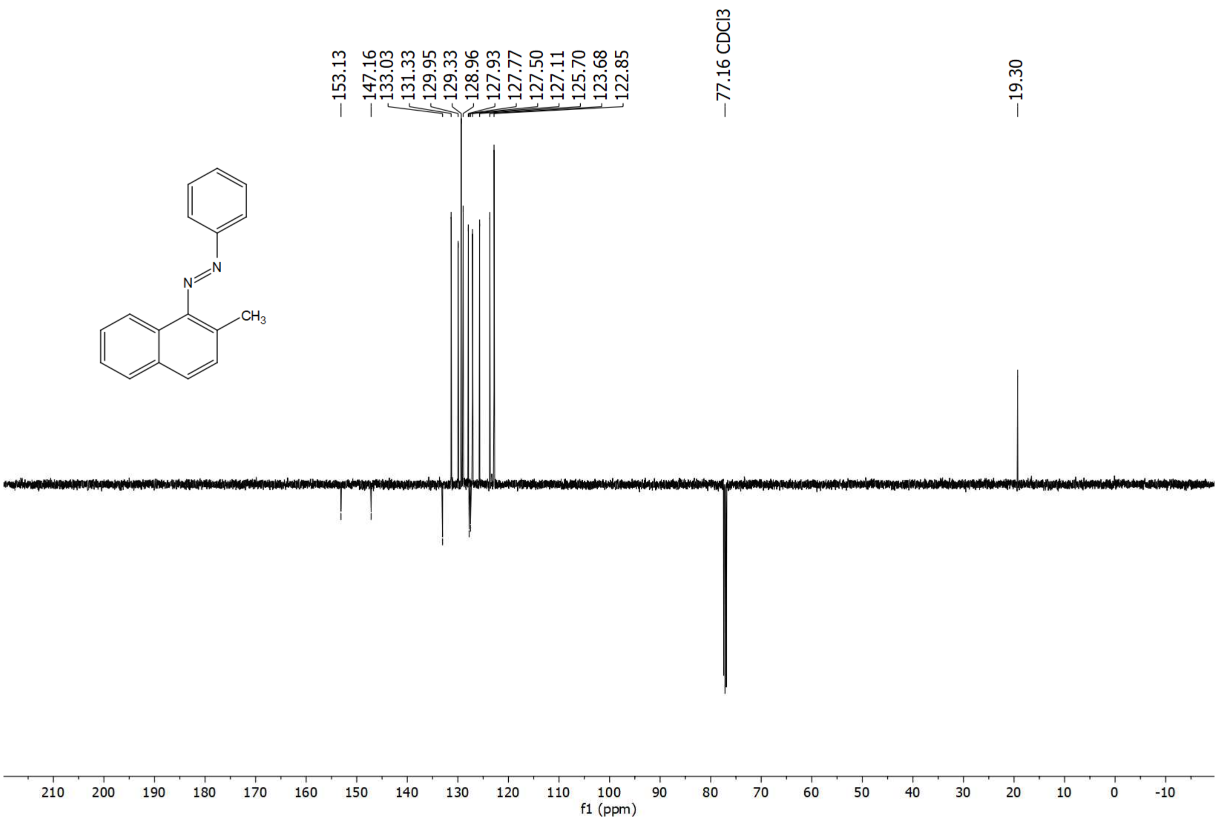

FT-IR (ATR) diamond of **3j**

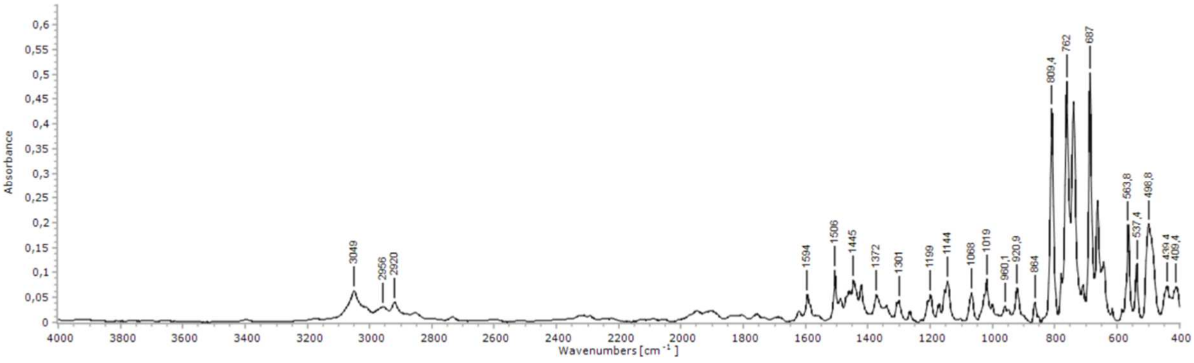

^{1}H NMR (500 MHz, Chloroform-*d*) of **3k**

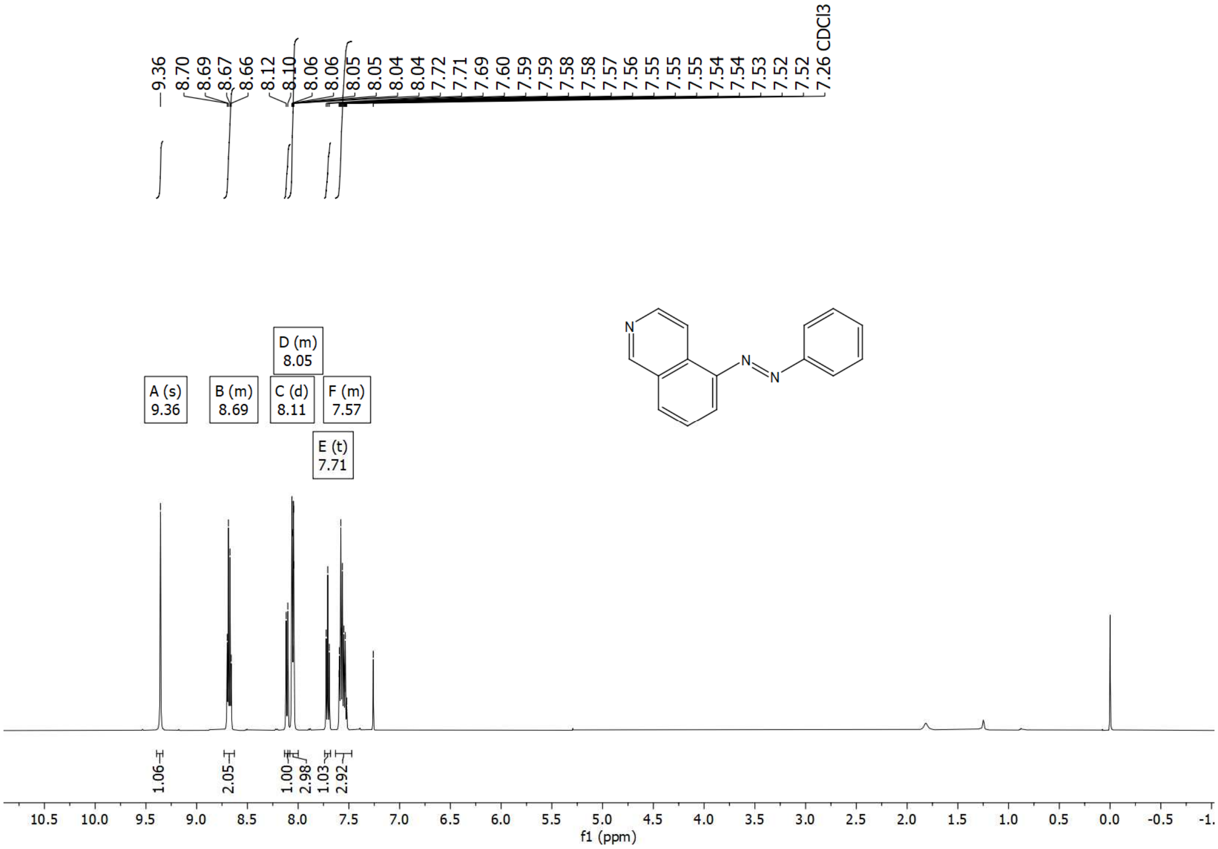

^{13}C APT NMR (126 MHz, $CDCl_3$) of **3k**

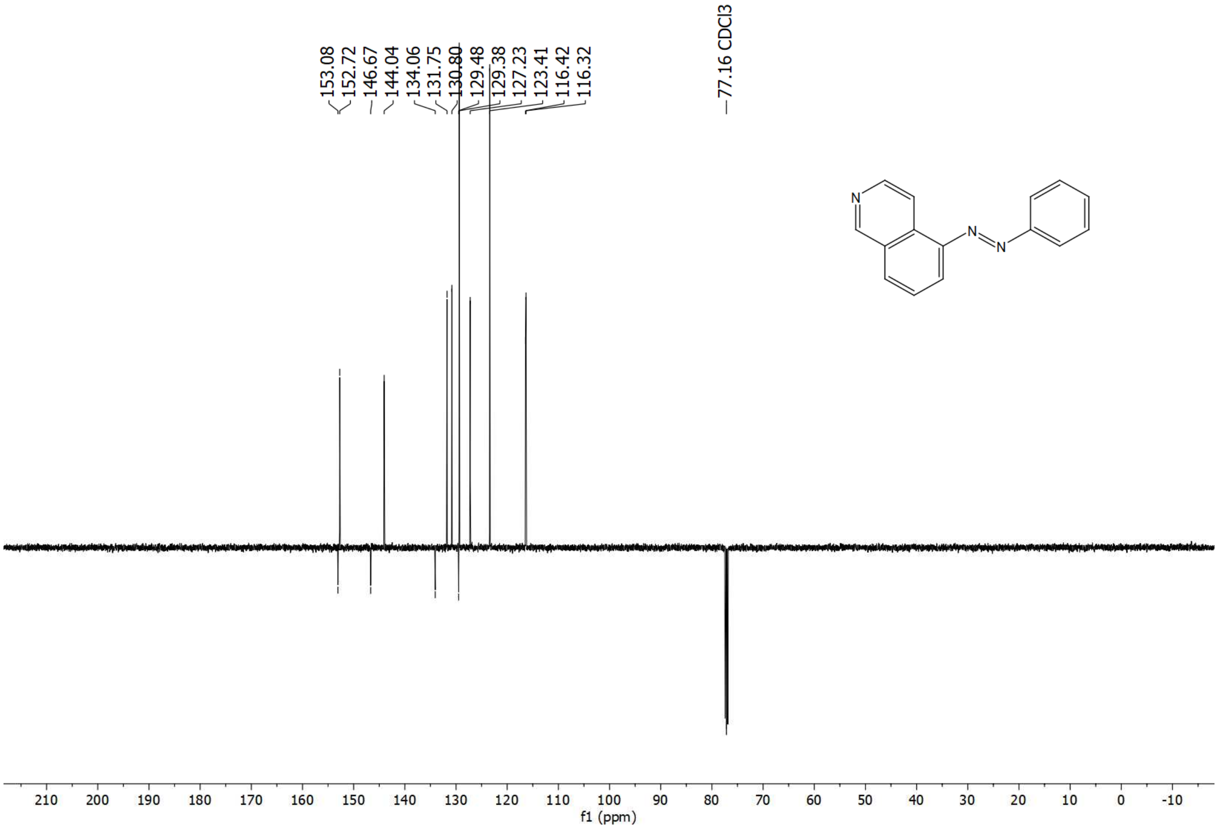

FT-IR (ATR) diamond of **3k**

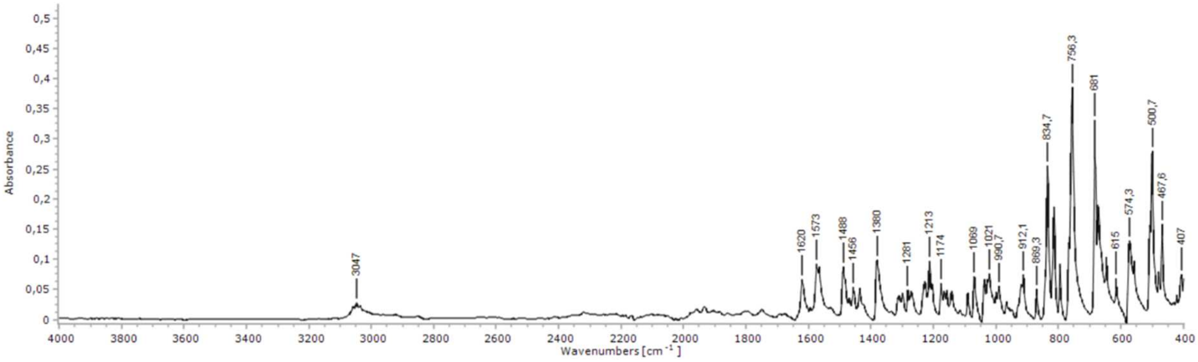

^{1}H NMR (500 MHz, Chloroform-*d*) of **3l**

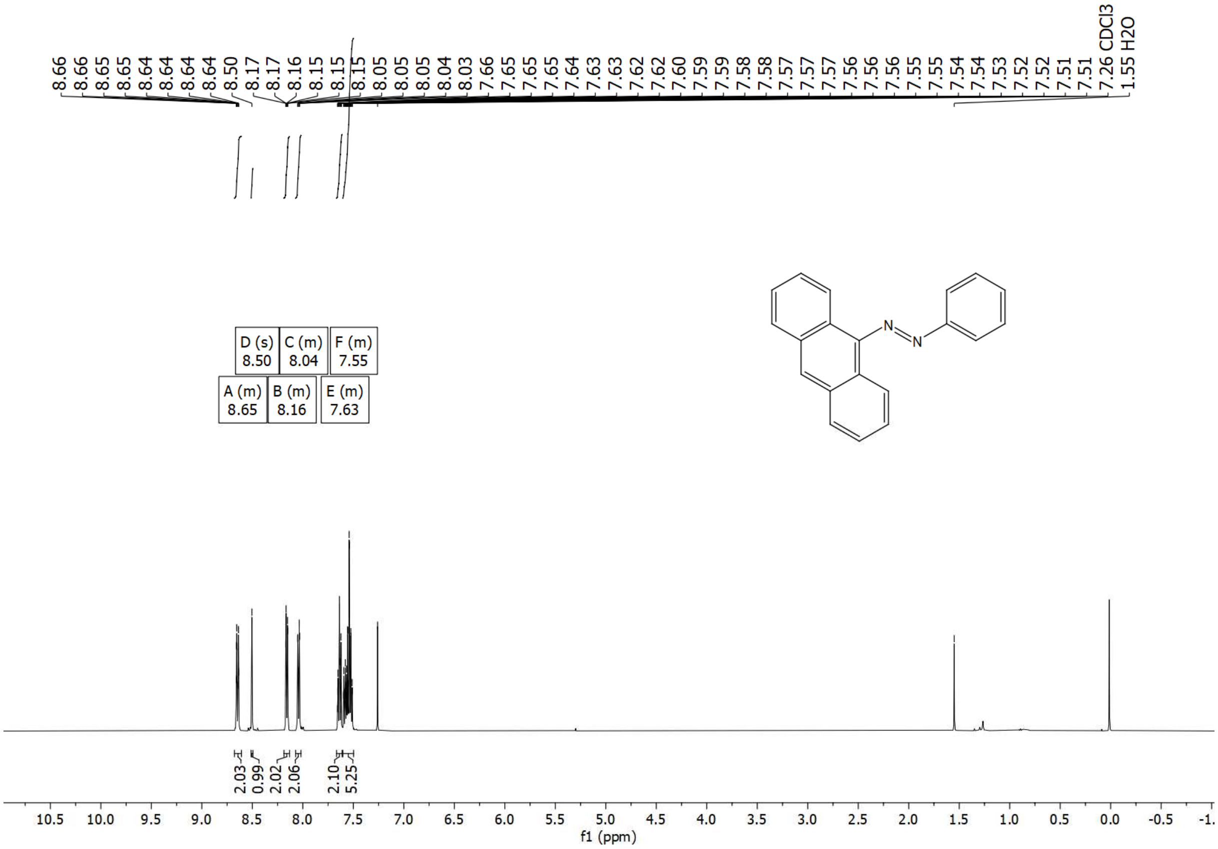

^{13}C APT NMR (126 MHz, $CDCl_3$) of **3l**

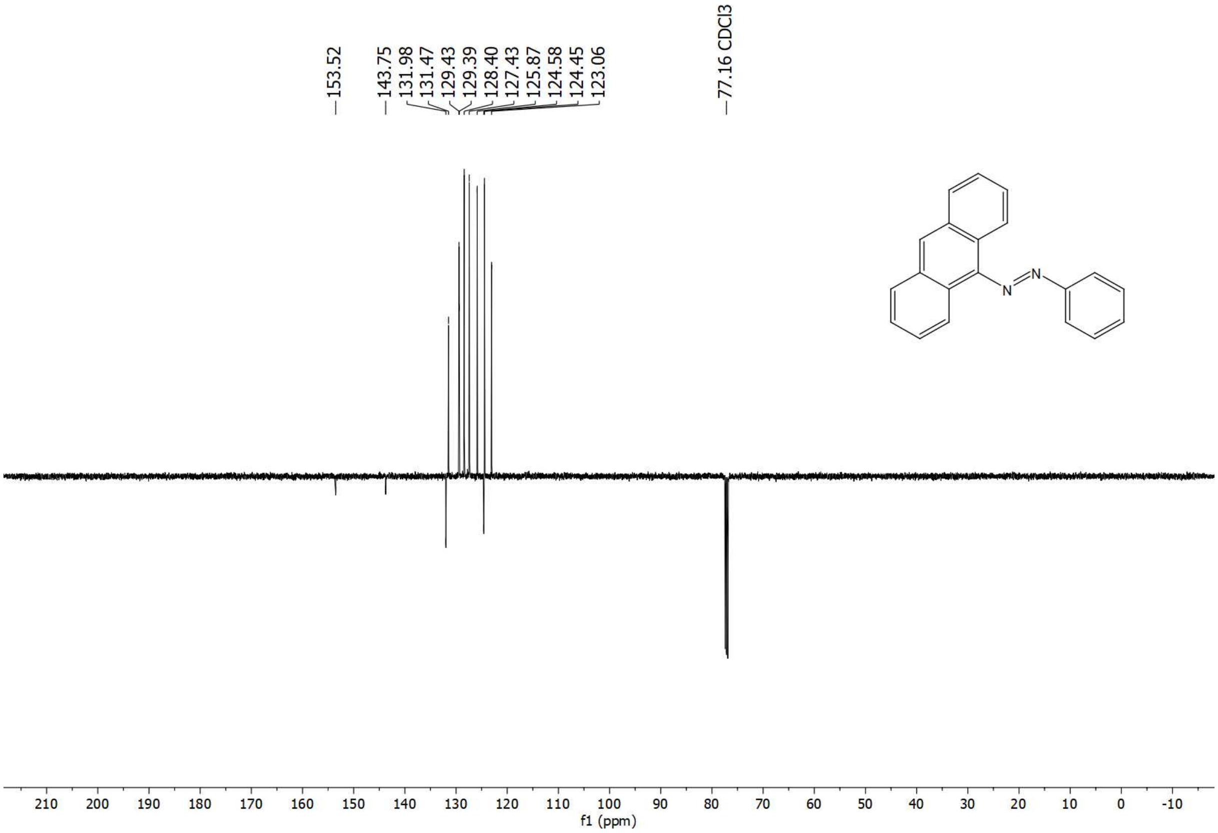

FT-IR (ATR) diamond of **3l**

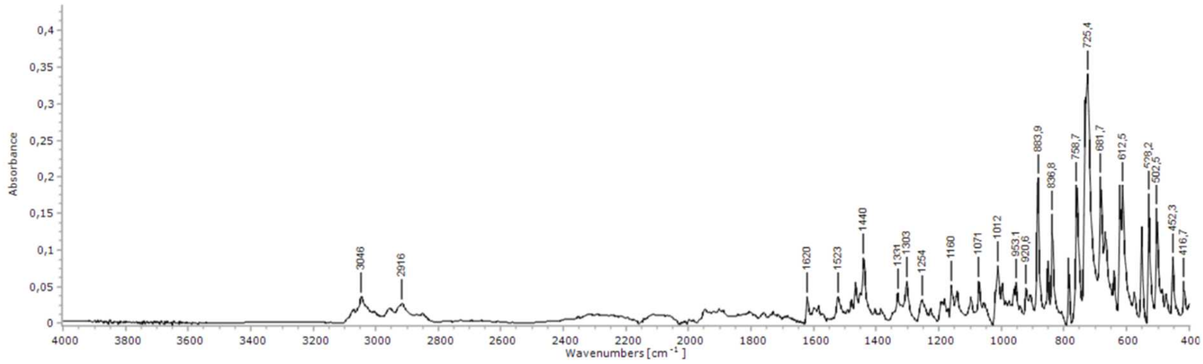

^{1}H NMR (500 MHz, Chloroform-*d*) of **3m**

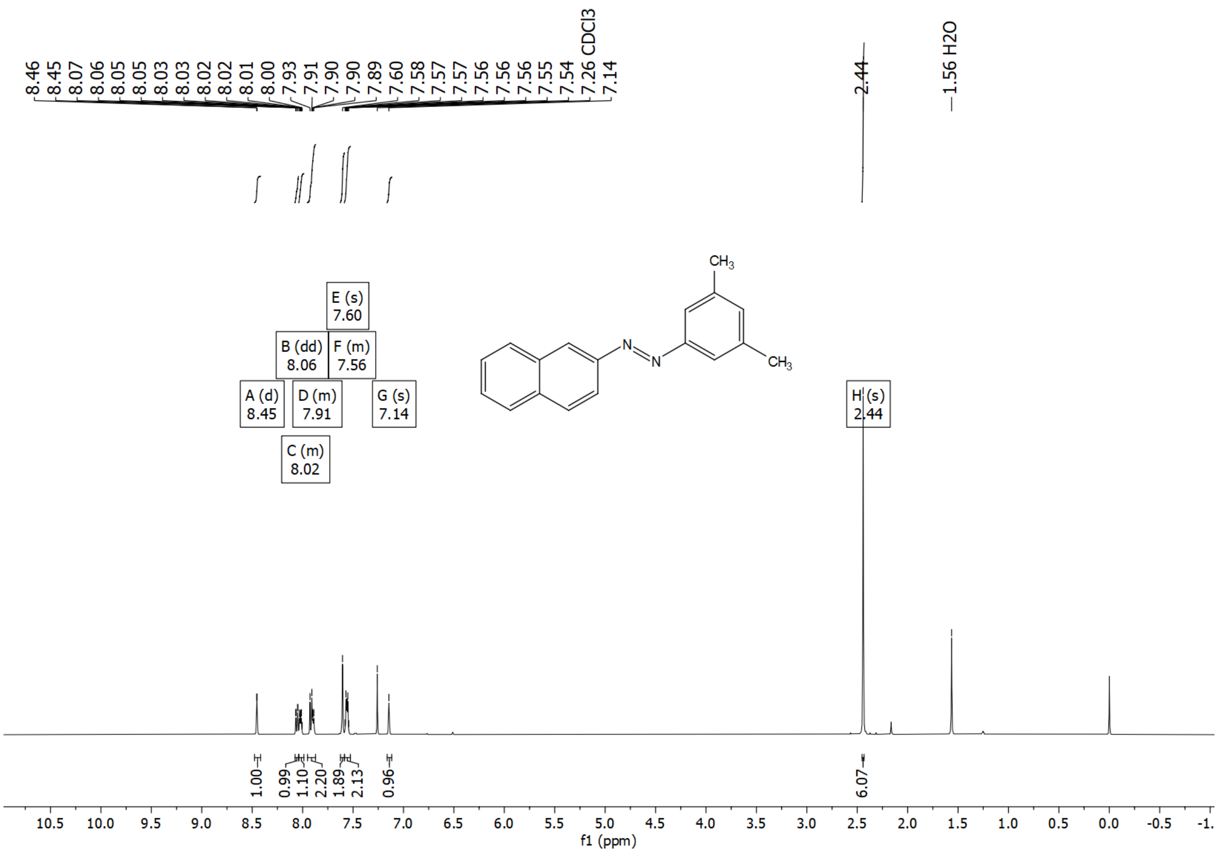

^{13}C APT NMR (126 MHz, $CDCl_3$) of **3m**

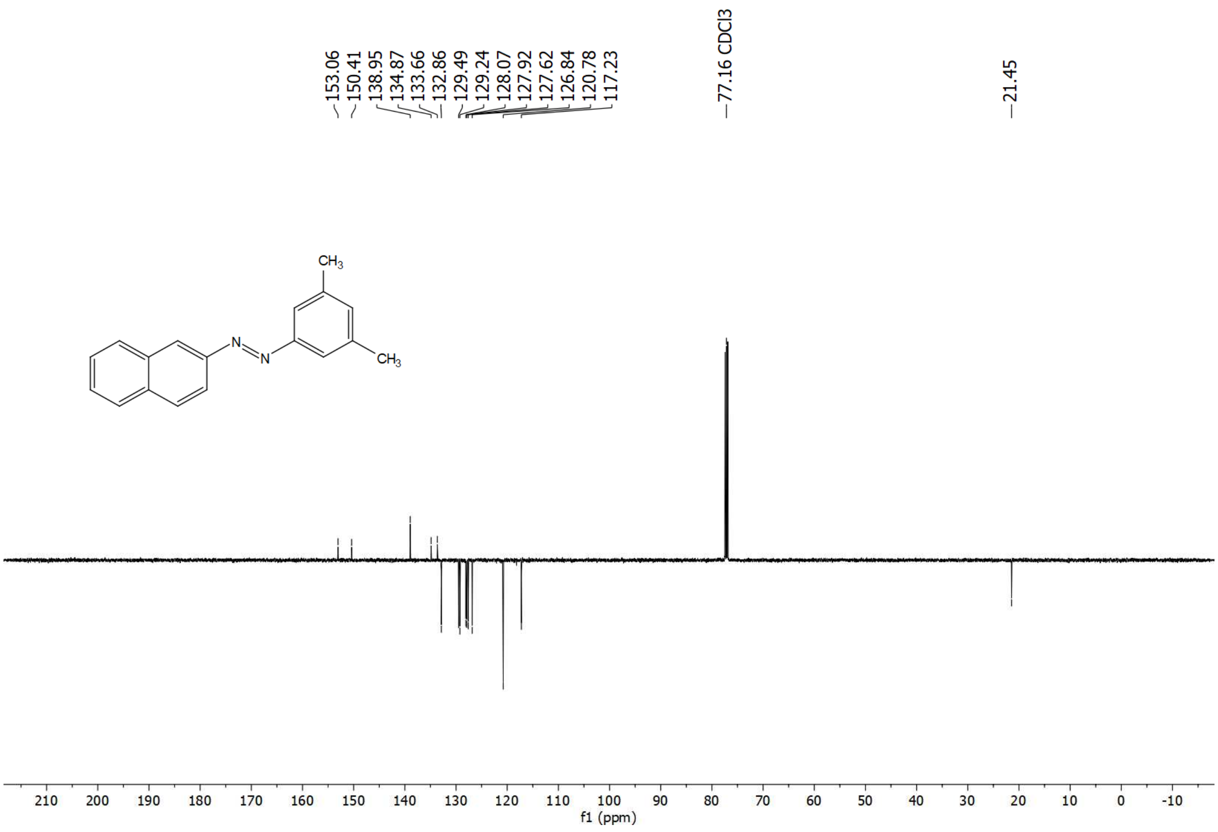

FT-IR (ATR) diamond of **3m**

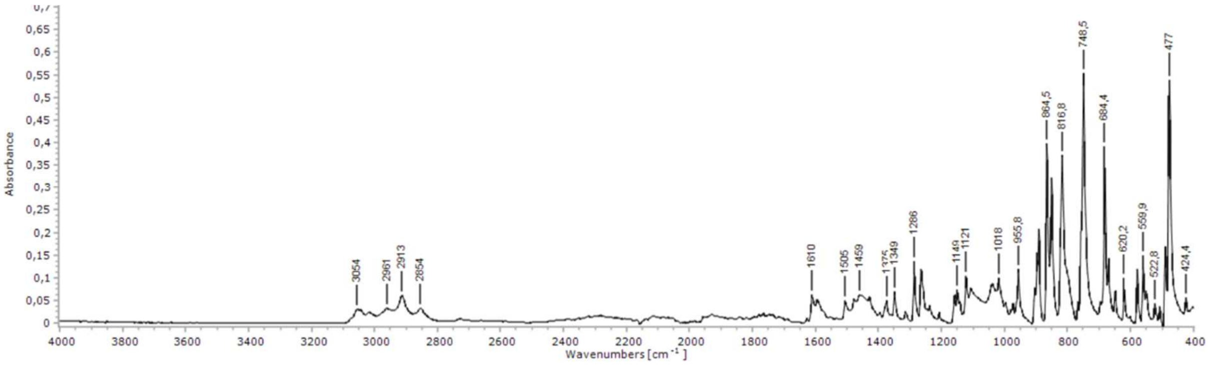

^{1}H NMR (500 MHz, Chloroform-*d*) of **3n**

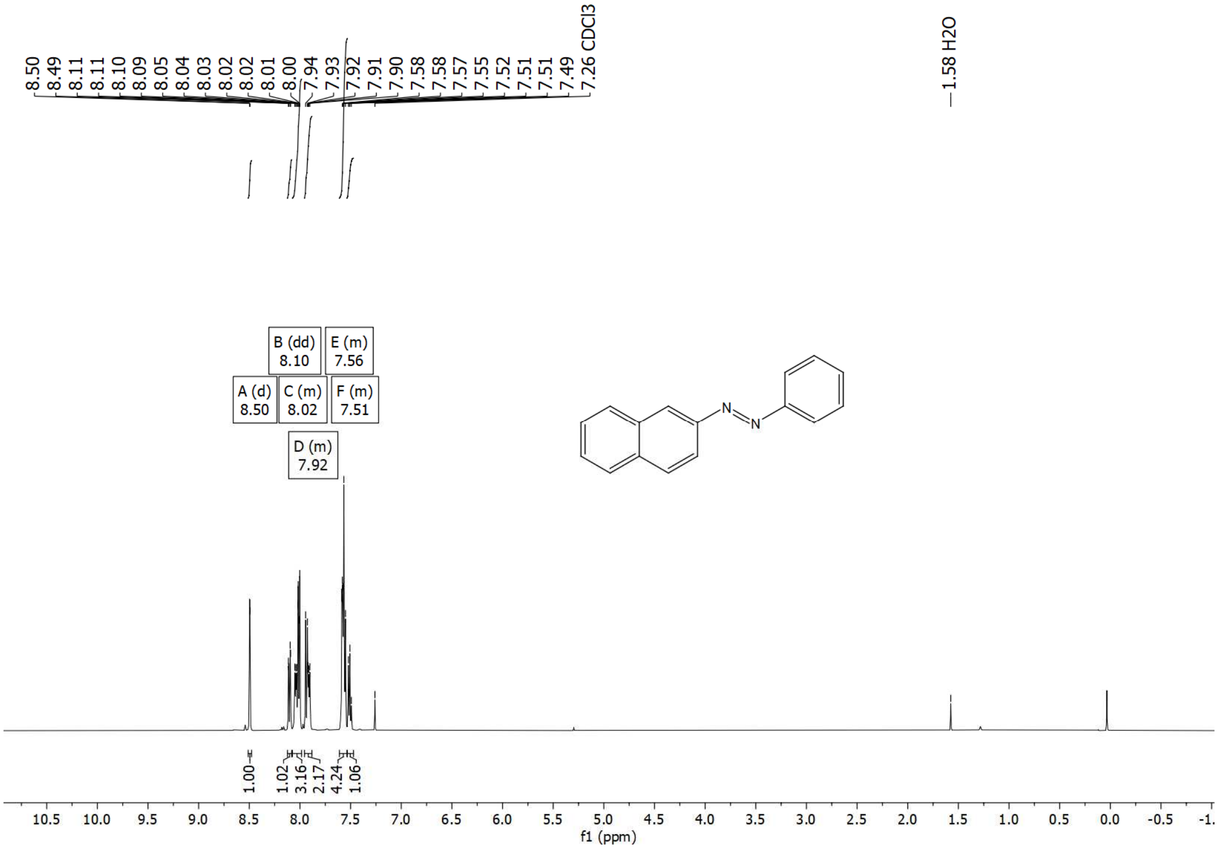

^{13}C APT NMR (126 MHz, $CDCl_3$) of **3n**

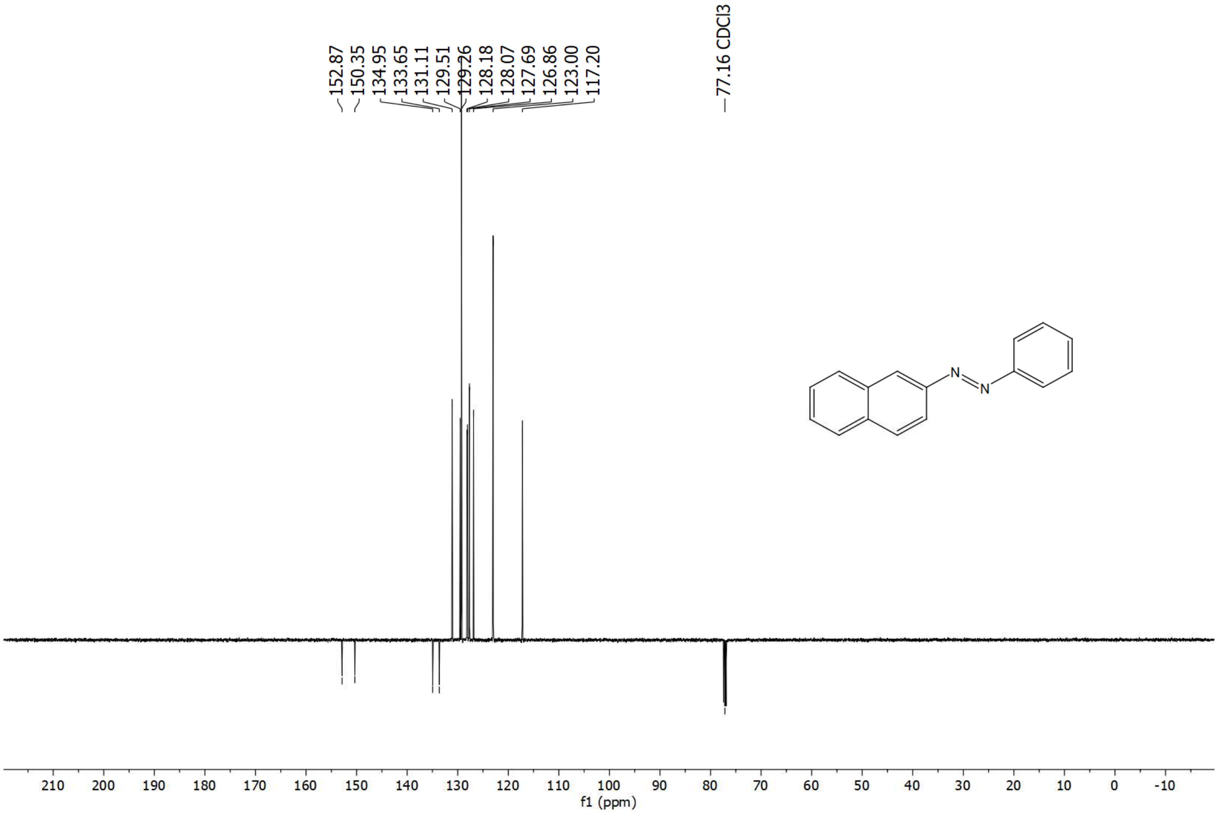

FT-IR (ATR) diamond of **3n**

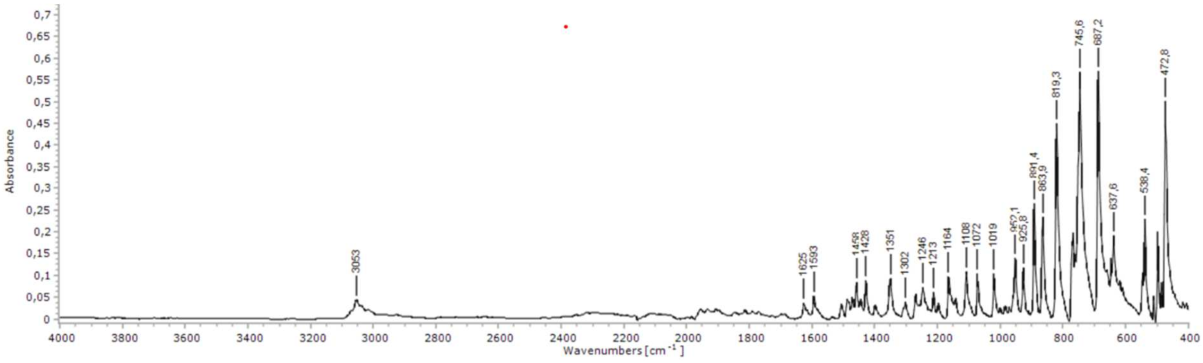

^{1}H NMR (500 MHz, Chloroform-*d*) of **3o**

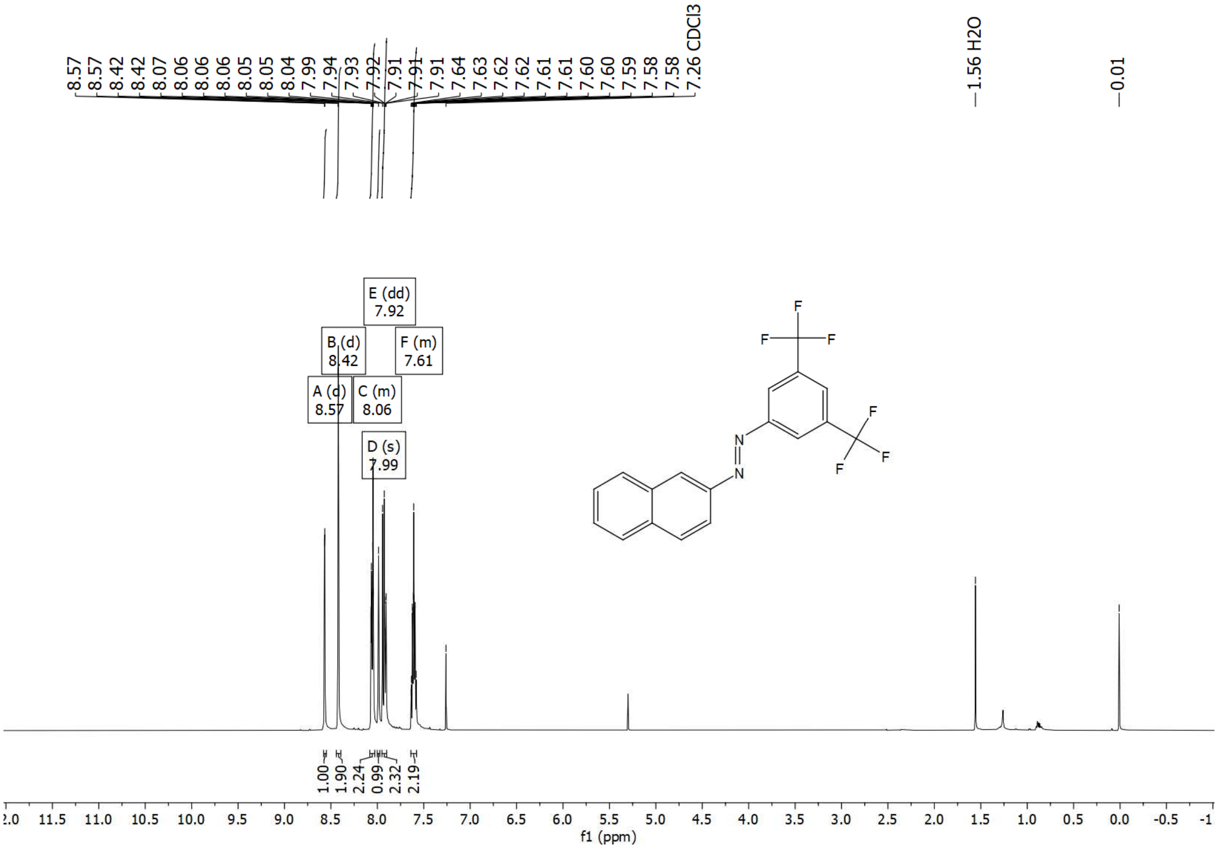

^{13}C APT NMR (126 MHz, $CDCl_3$) of **3o**

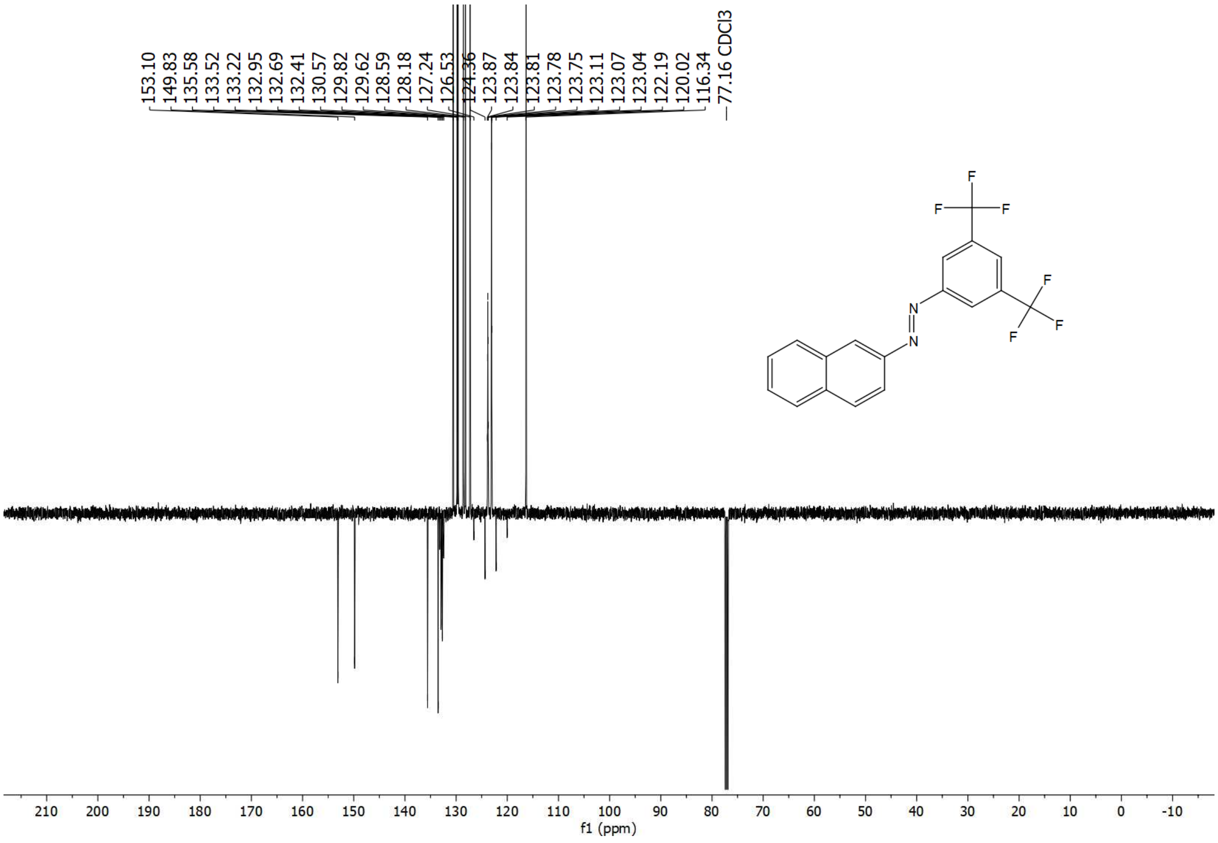

$^{19}F\{H\}$ NMR (376 MHz, $CDCl_3$) of **3o**

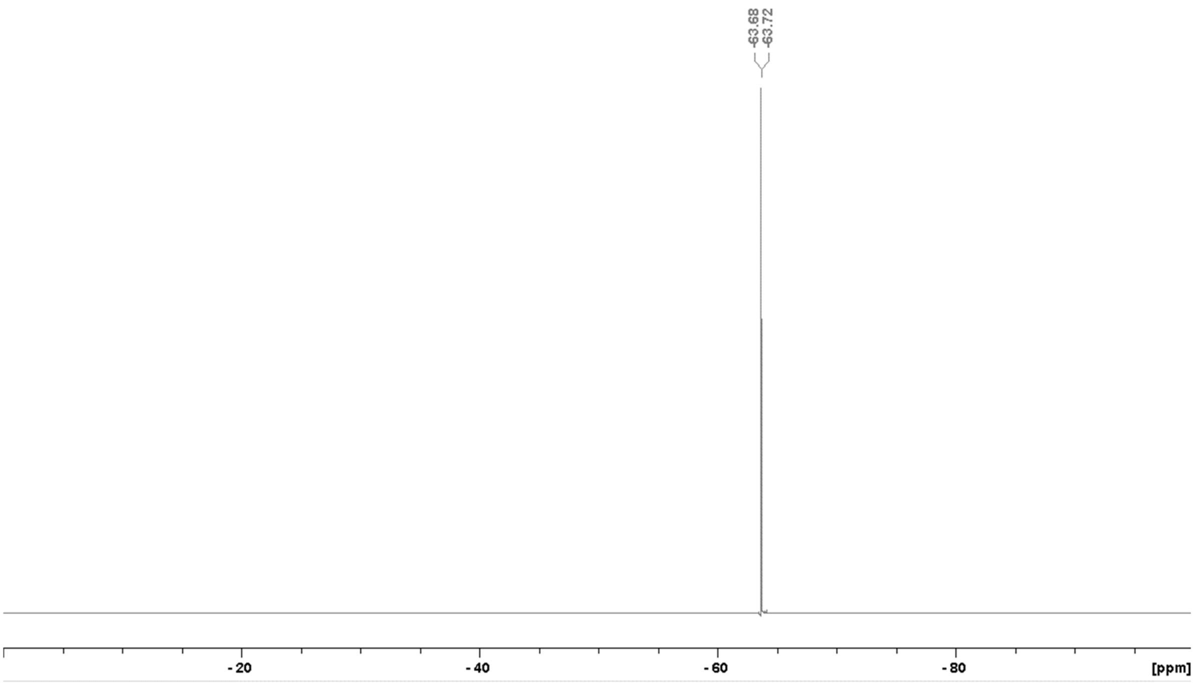

FT-IR (ATR) diamond of **3o**

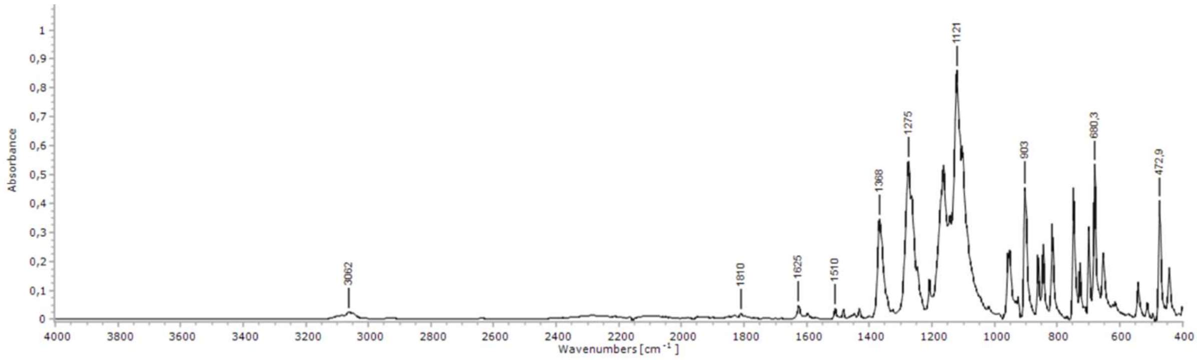

^{1}H NMR (500 MHz, Chloroform-*d*) of **3q**

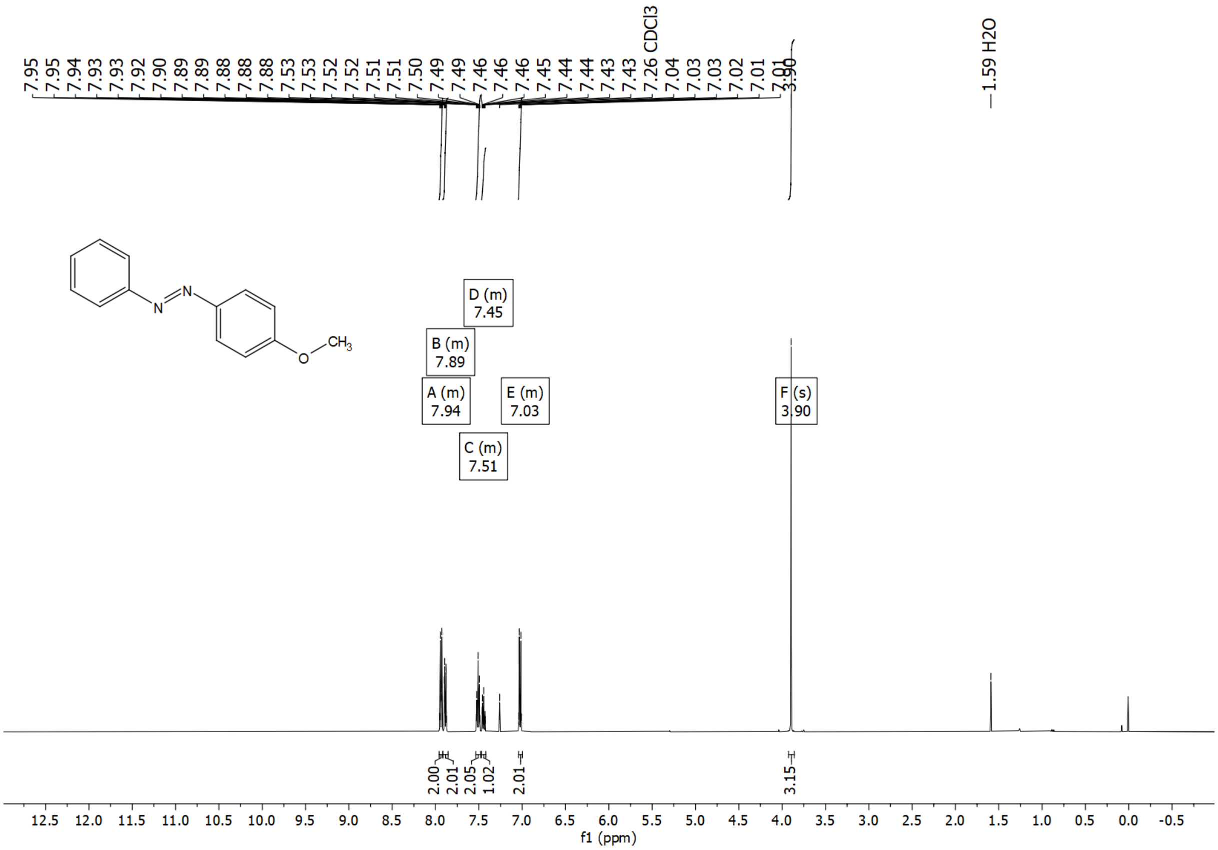

^{13}C APT NMR (126 MHz, $CDCl_3$) of **3q**

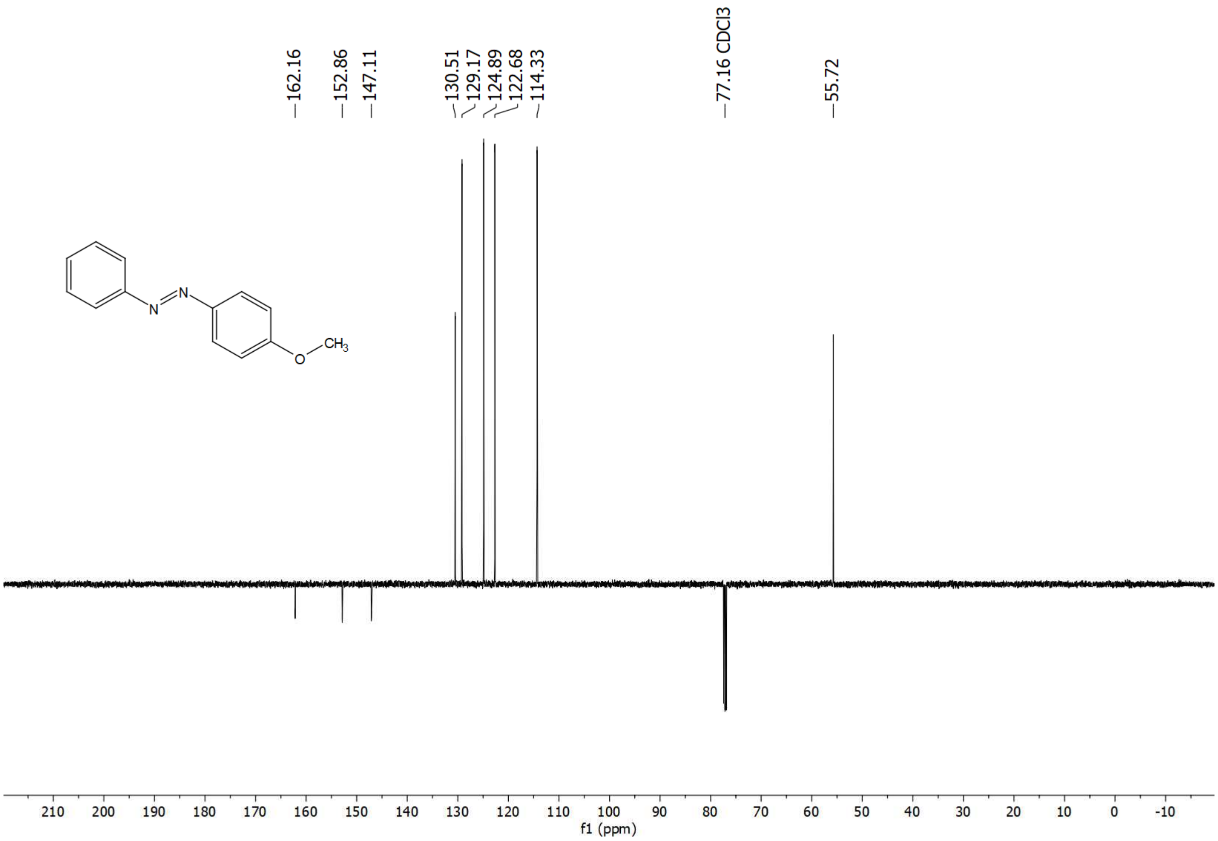

FT-IR (ATR) diamond of **3q**

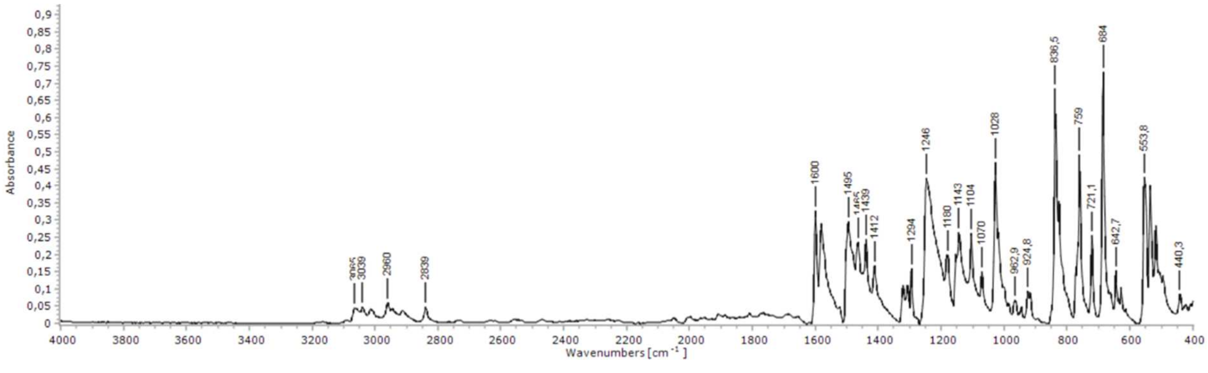

^{1}H NMR (500 MHz, Chloroform-*d*) of **3r**

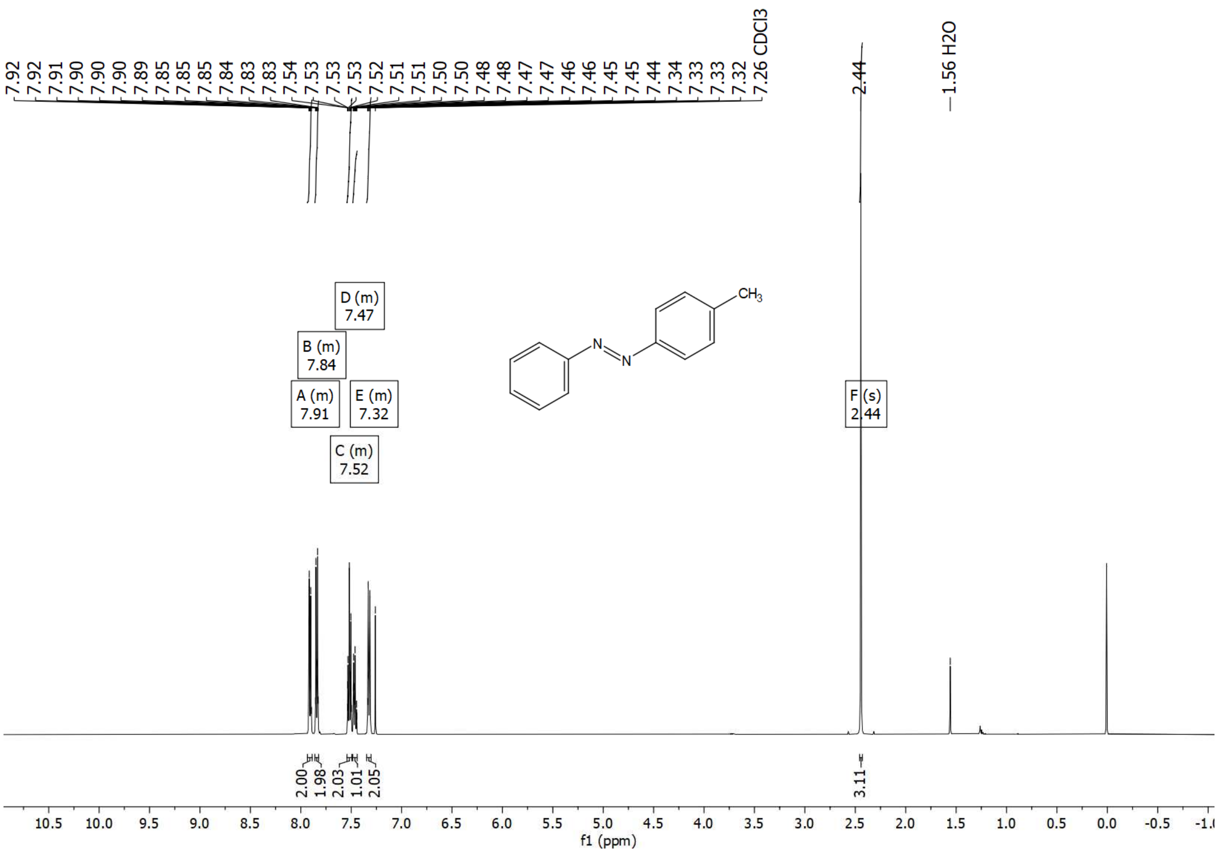

^{13}C APT NMR (126 MHz, $CDCl_3$) of **3r**

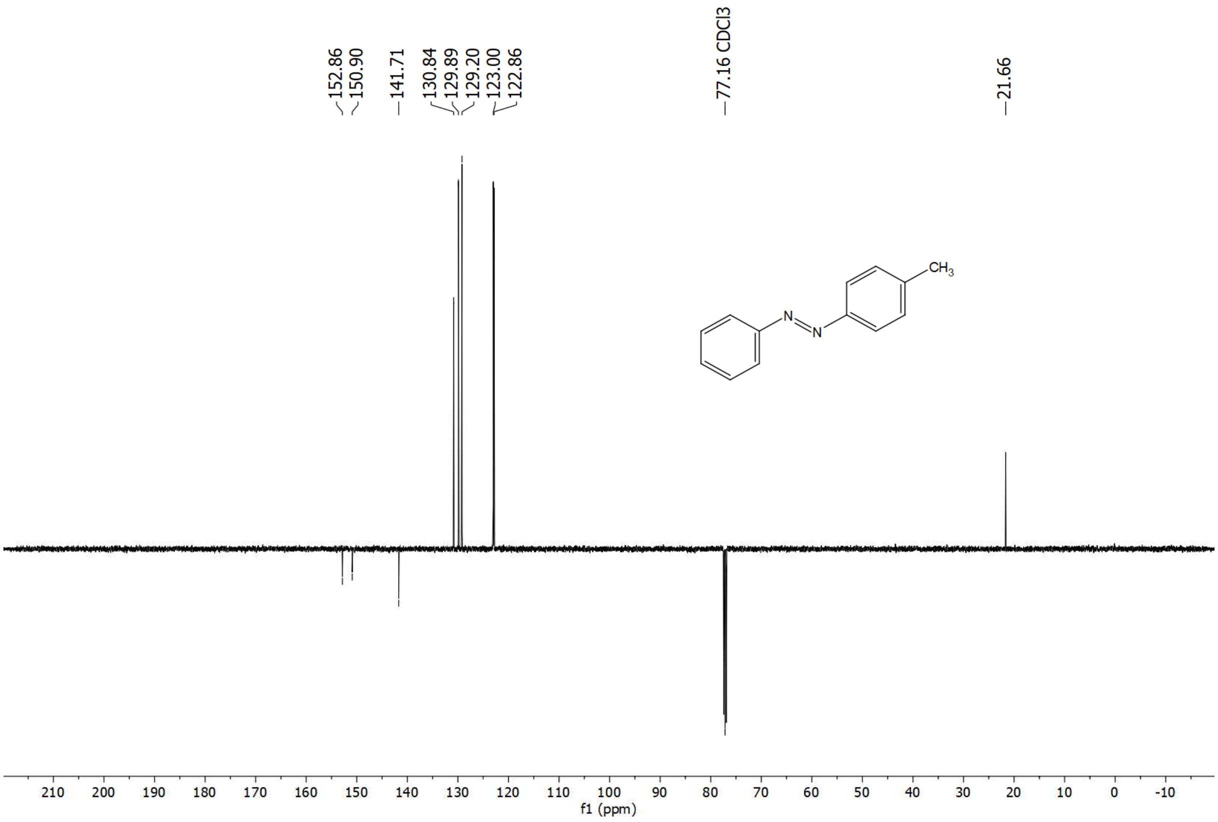

FT-IR (ATR) diamond of **3r**

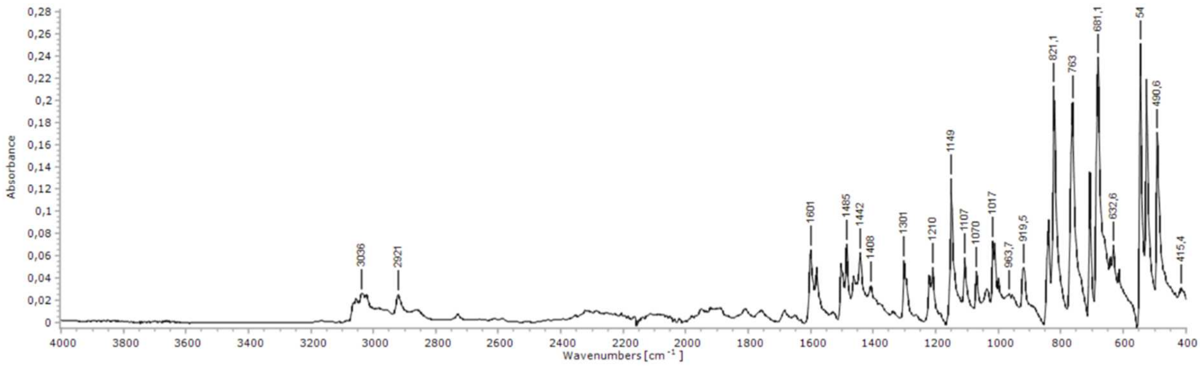

^{1}H NMR (500 MHz, Chloroform-*d*) of **3s**

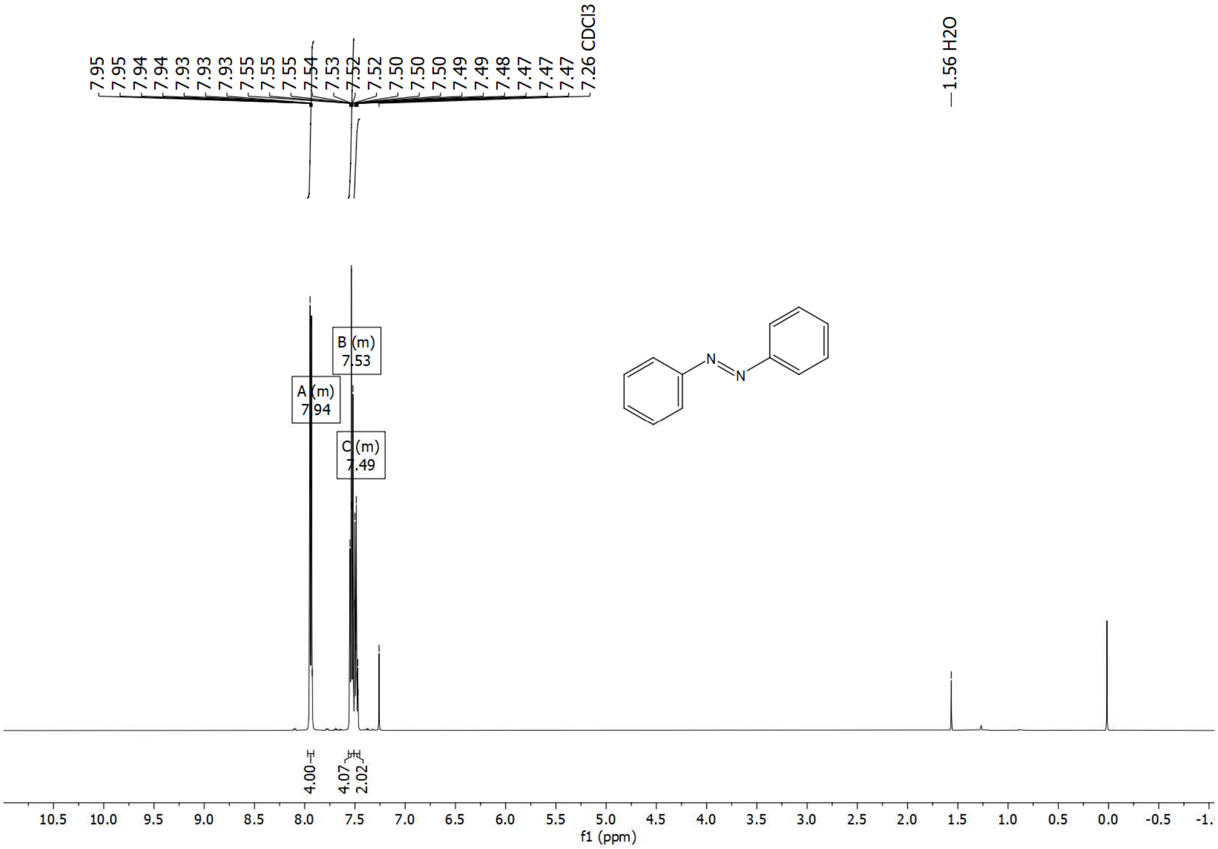

^{13}C APT NMR (126 MHz, $CDCl_3$) of **3s**

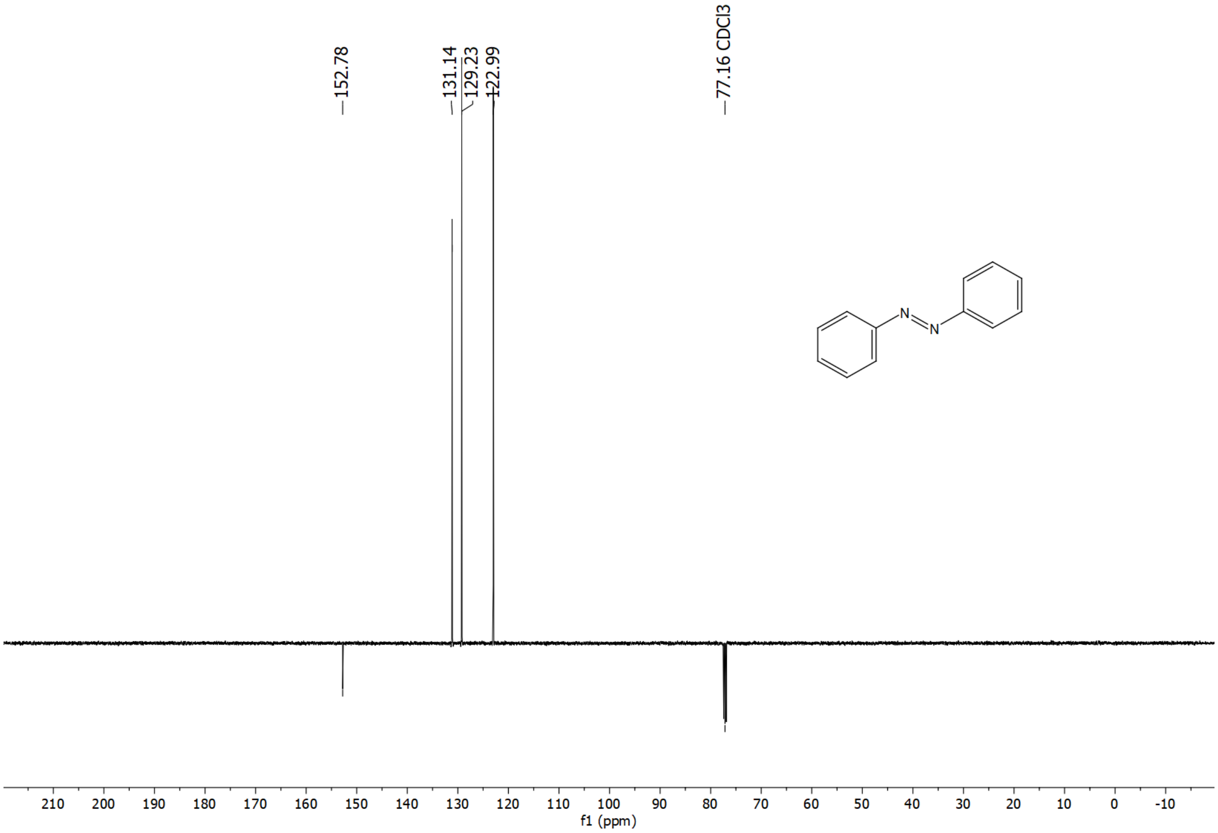

FT-IR (ATR) diamond of **3s**

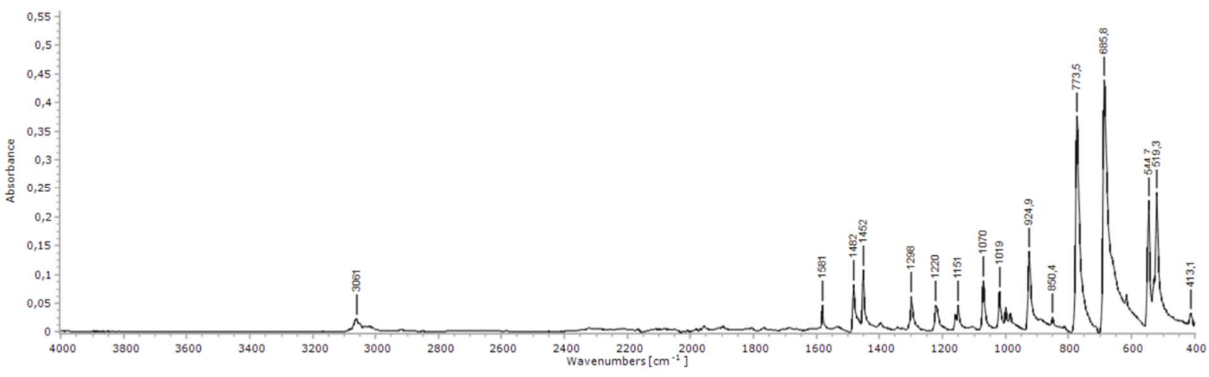

^{1}H NMR (500 MHz, Chloroform-*d*) of **3t**

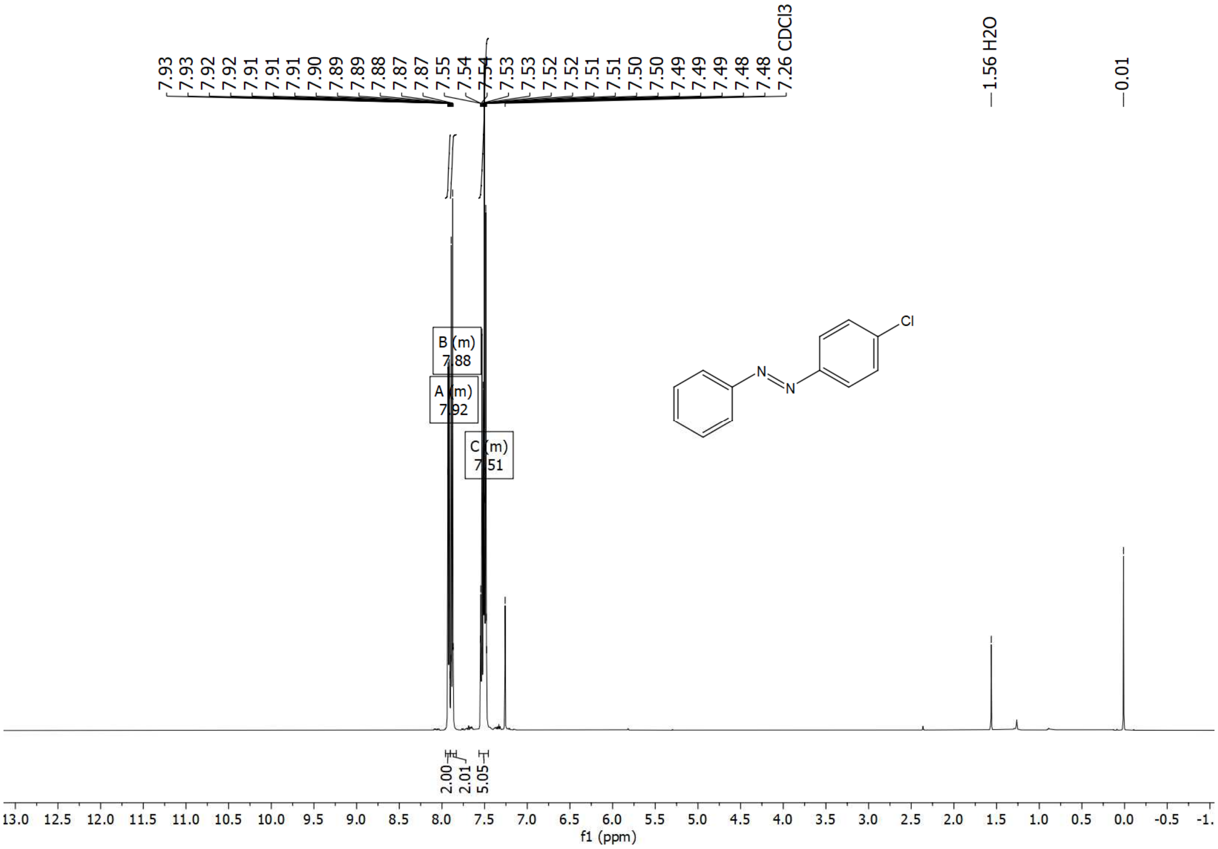

^{13}C APT NMR (126 MHz, $CDCl_3$) of **3t**

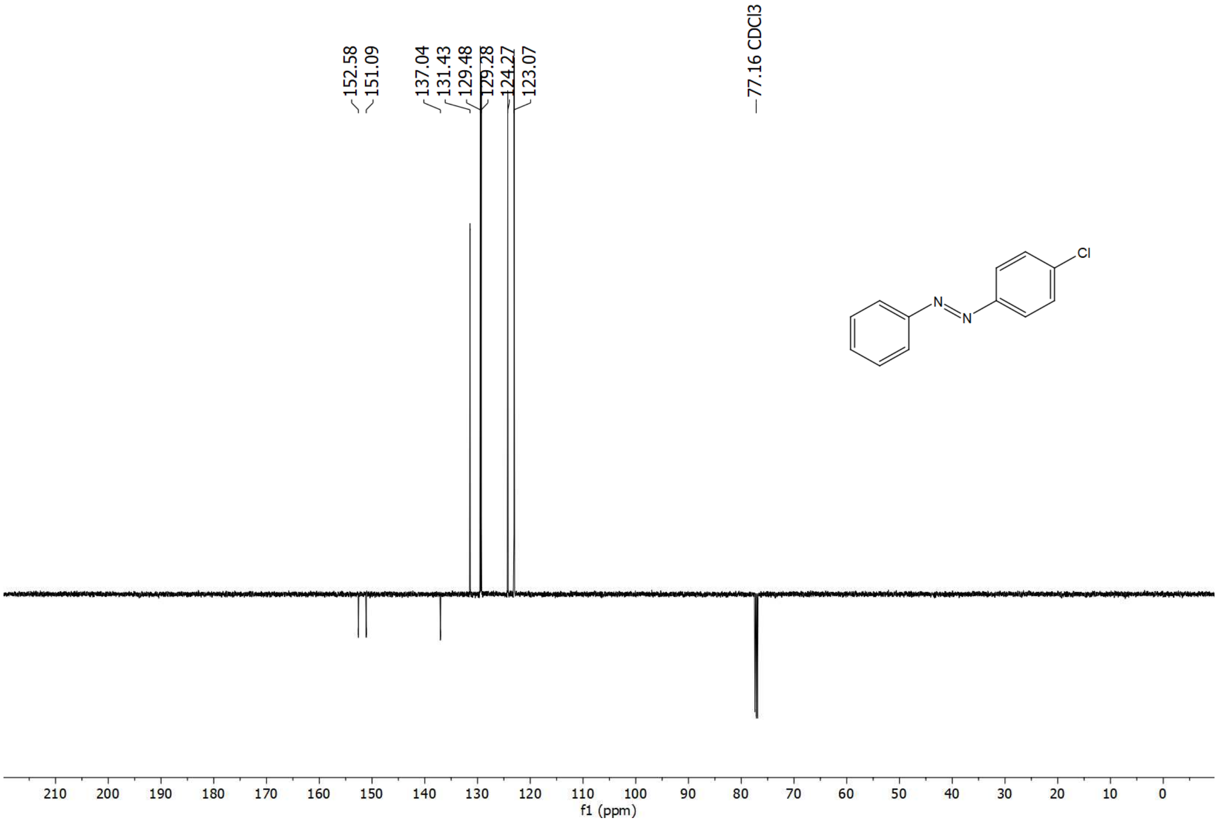

FT-IR (ATR) diamond of **3t**

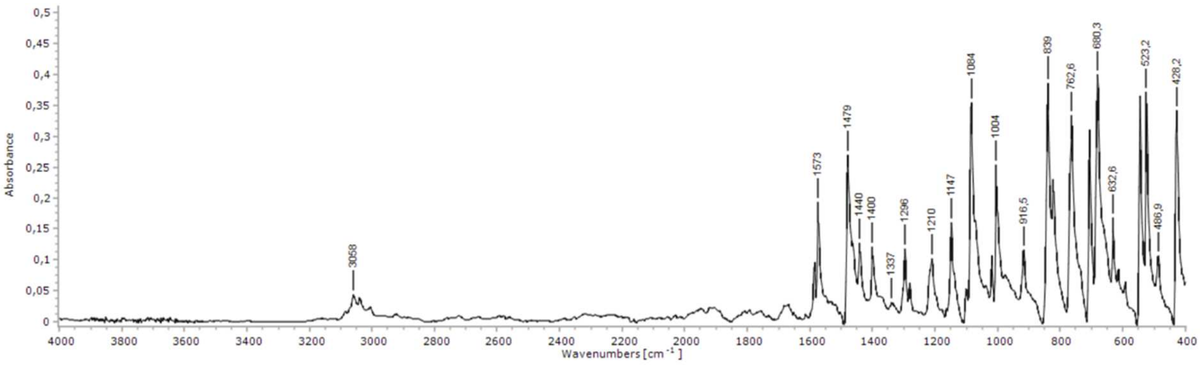

^{1}H NMR (500 MHz, Chloroform-*d*) of **3u**

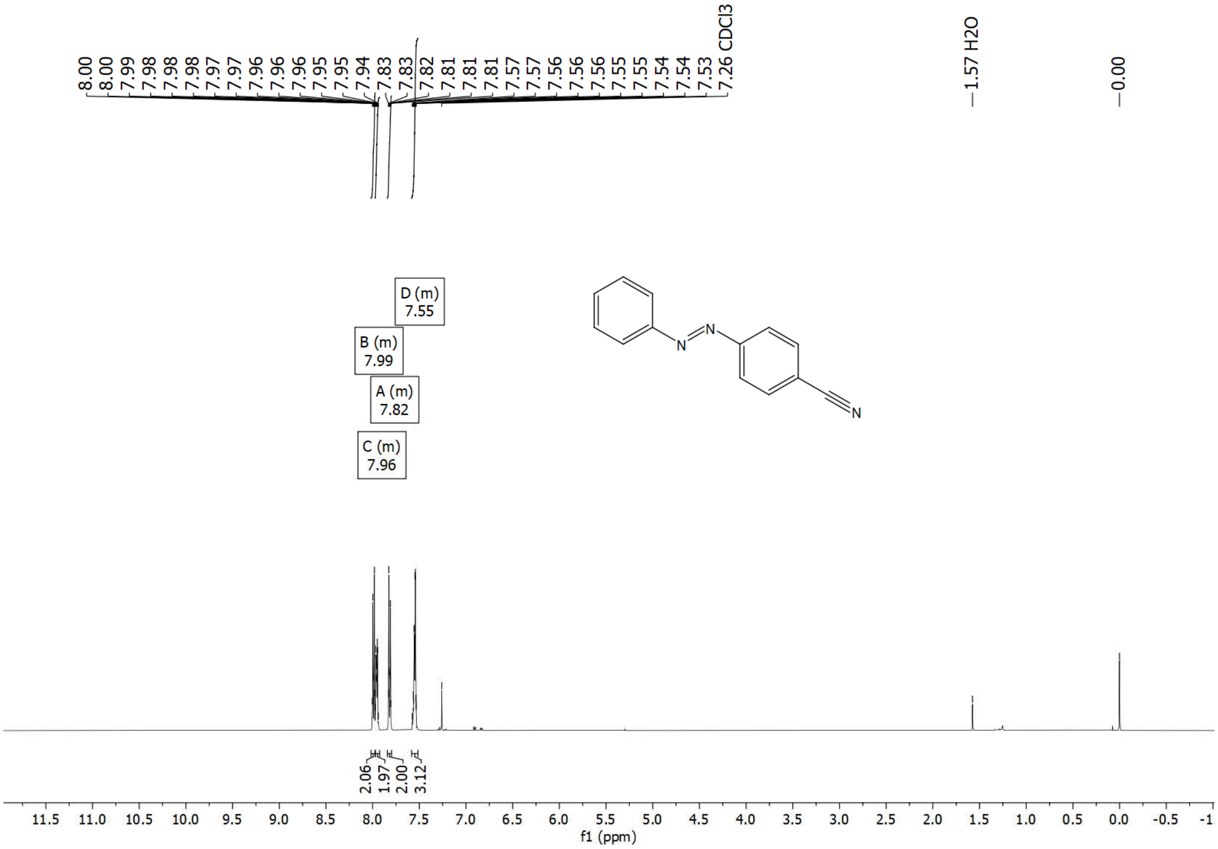

^{13}C APT NMR (126 MHz, $CDCl_3$) of **3u**

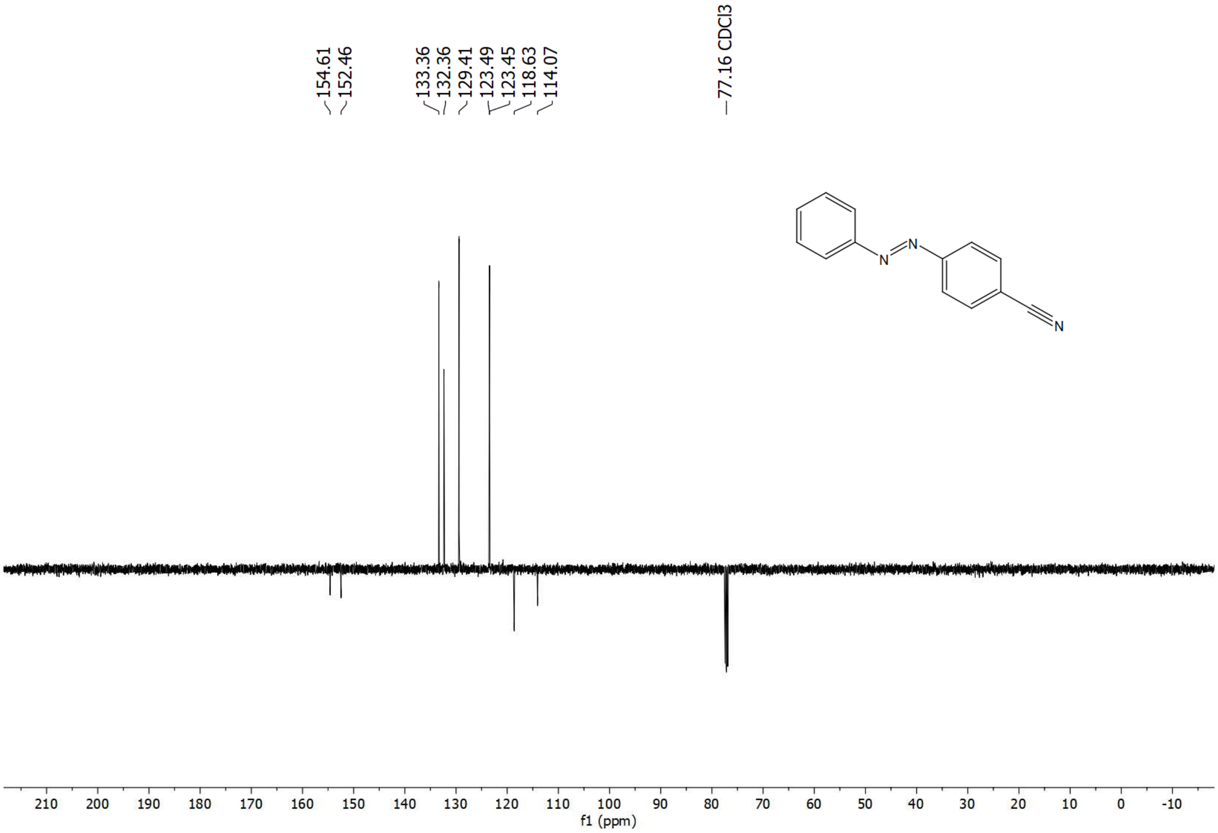

FT-IR (ATR) diamond of **3u**

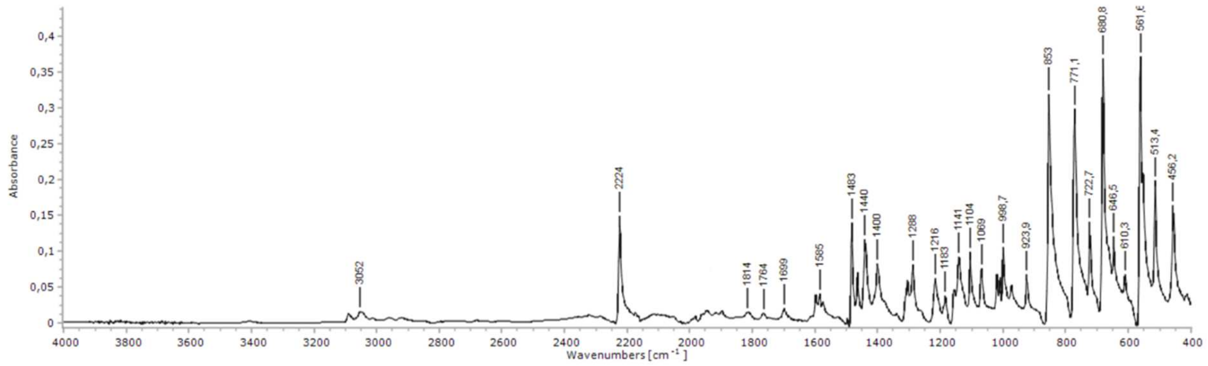

^{1}H NMR (500 MHz, Chloroform-*d*) of **3v**

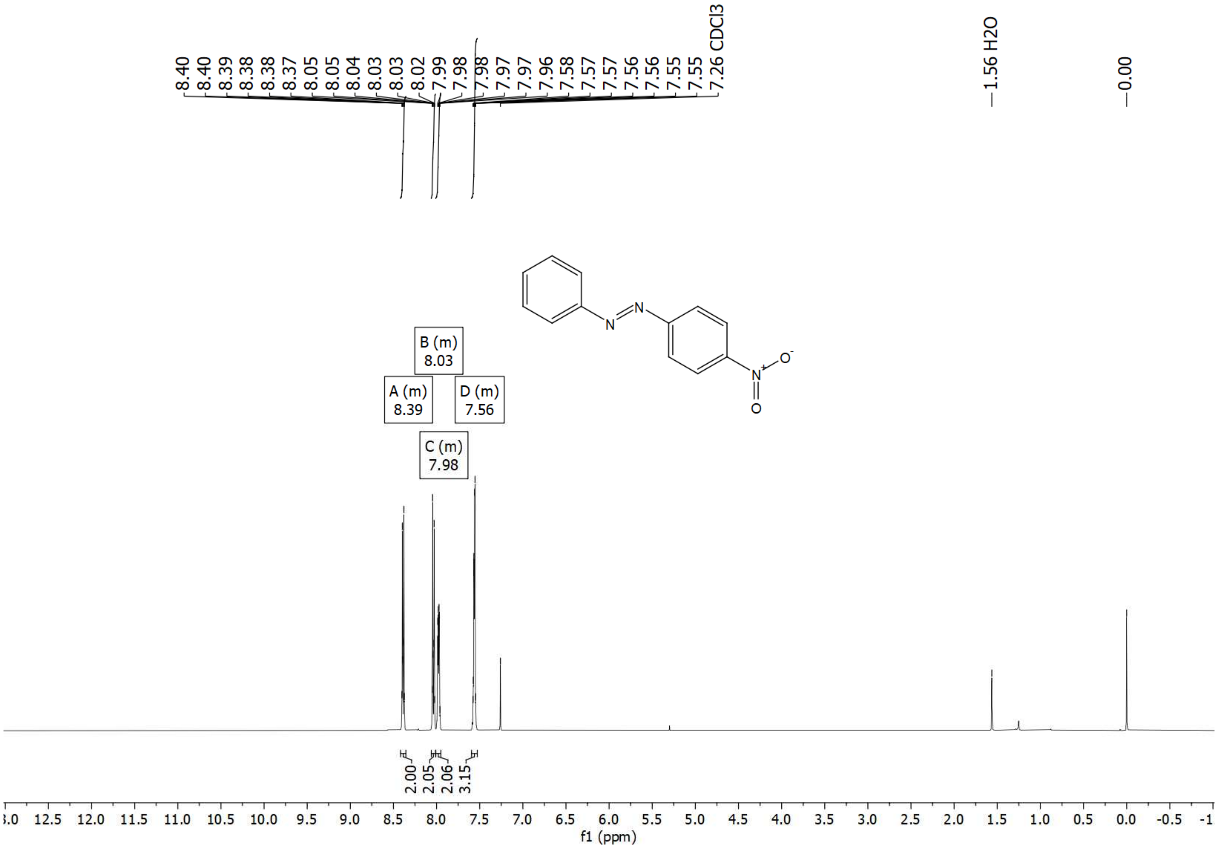

^{13}C APT NMR (126 MHz, $CDCl_3$) of **3v**

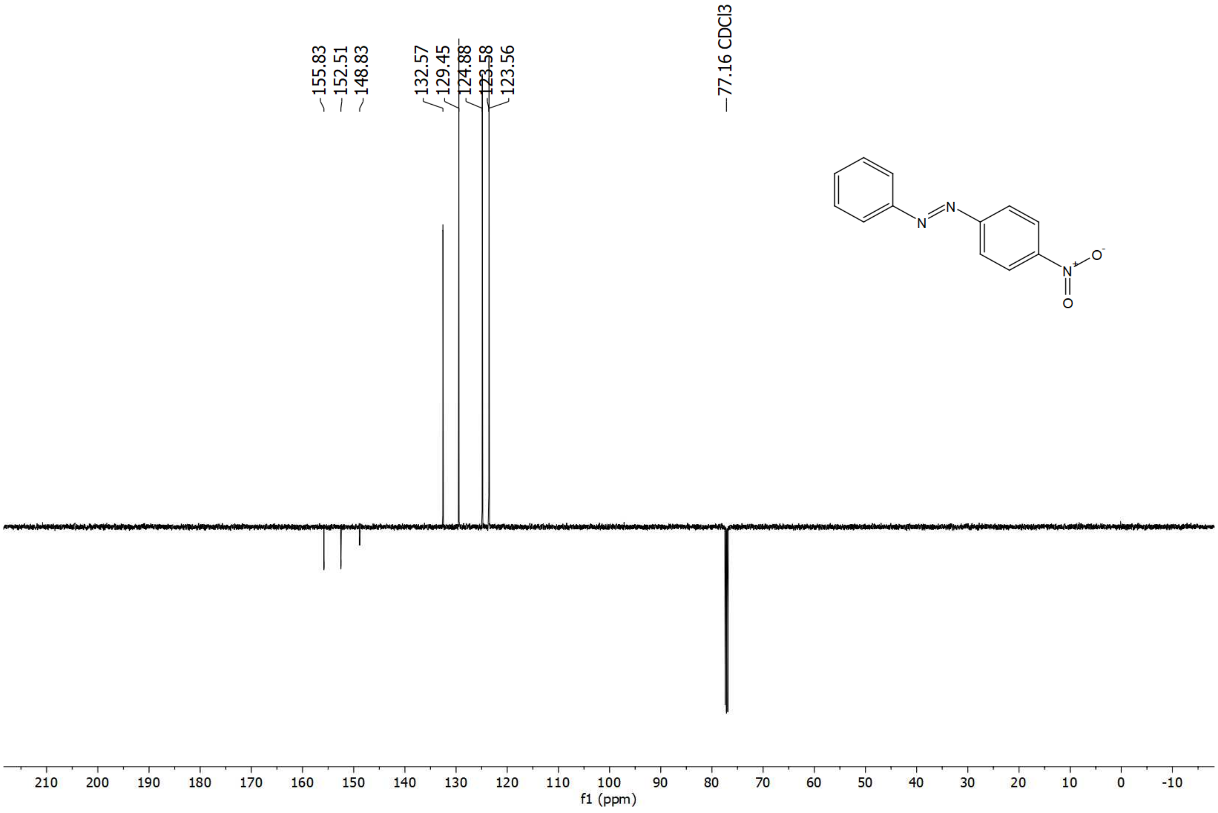

FT-IR (ATR) diamond of **3v**

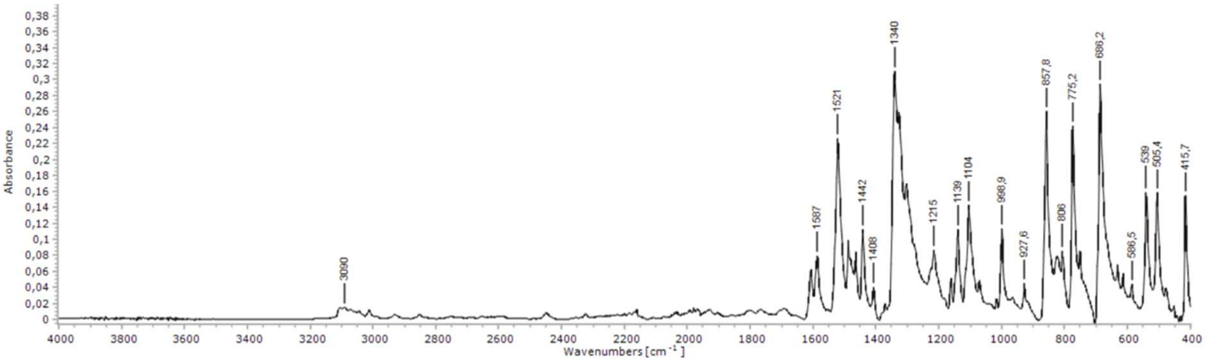

^{1}H NMR (500 MHz, Chloroform-*d*) of **3w**

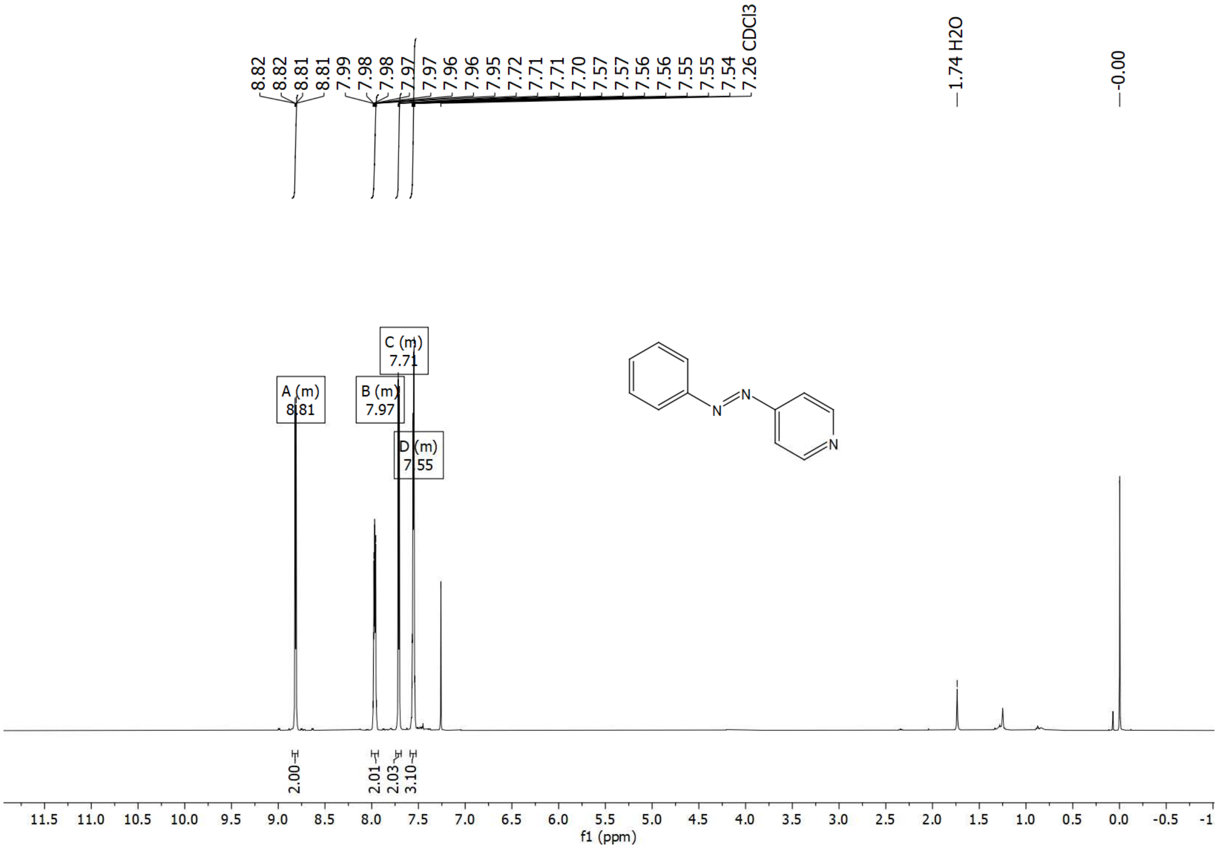

^{13}C APT NMR (126 MHz, $CDCl_3$) of **3w**

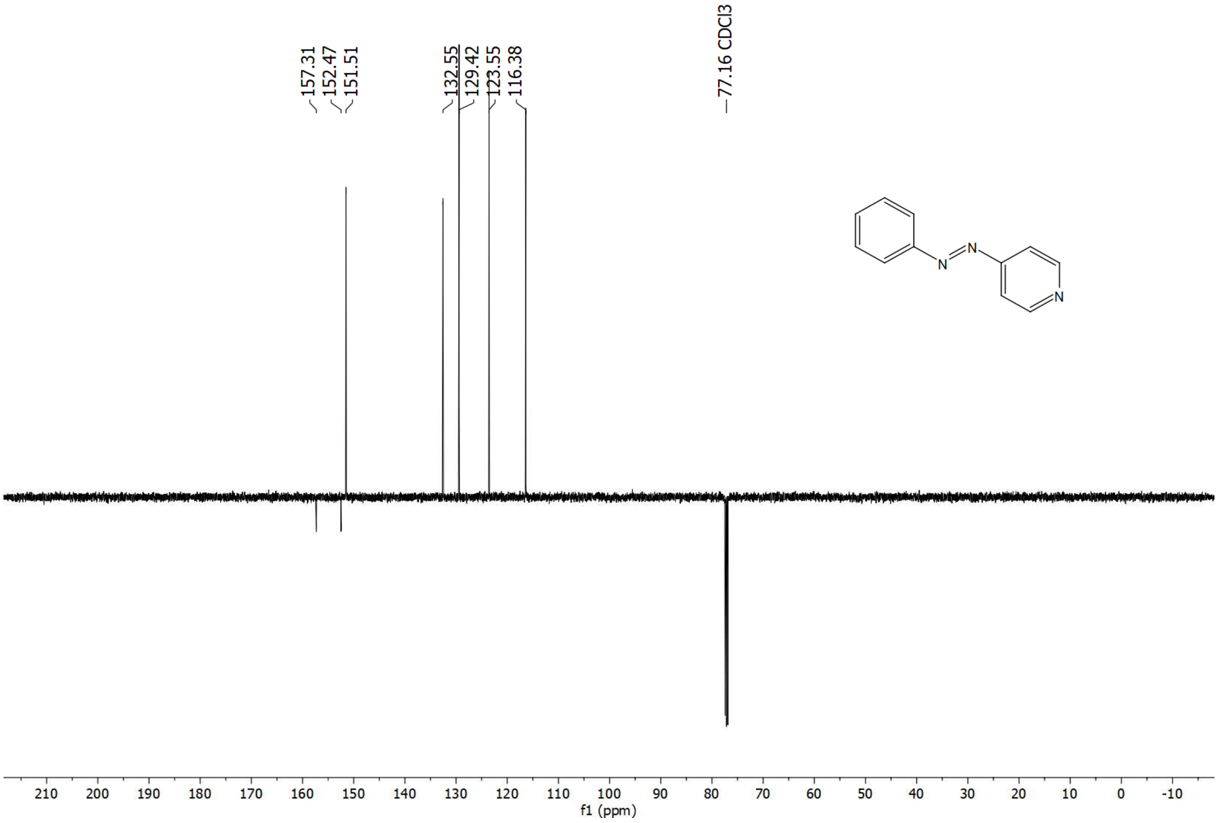

FT-IR (ATR) diamond of **3w**

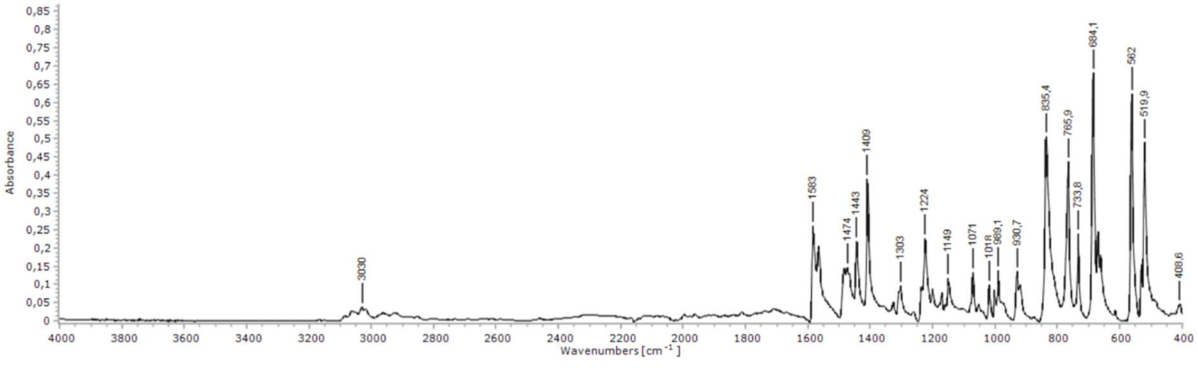

^{1}H NMR (500 MHz, Chloroform-d) of **3x**

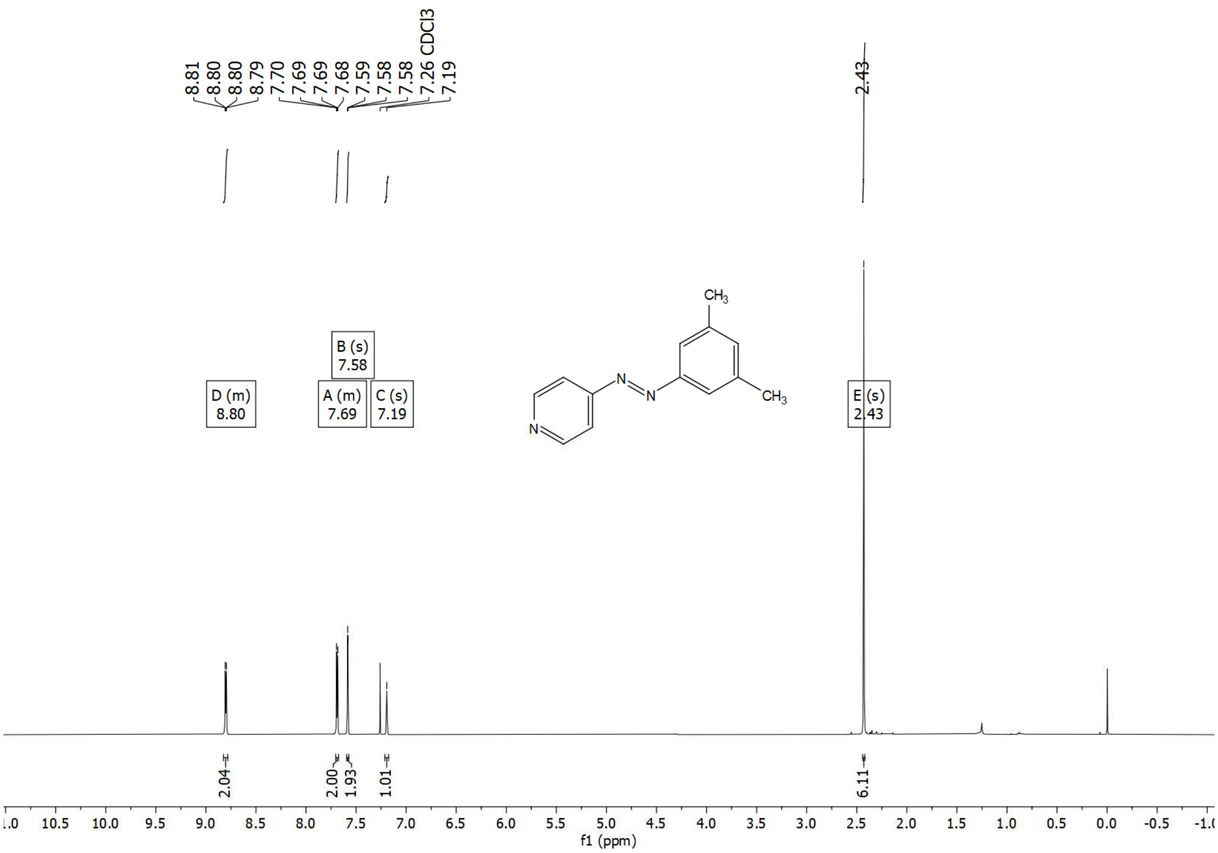

^{13}C APT NMR (126 MHz, $CDCl_3$) of **3x**

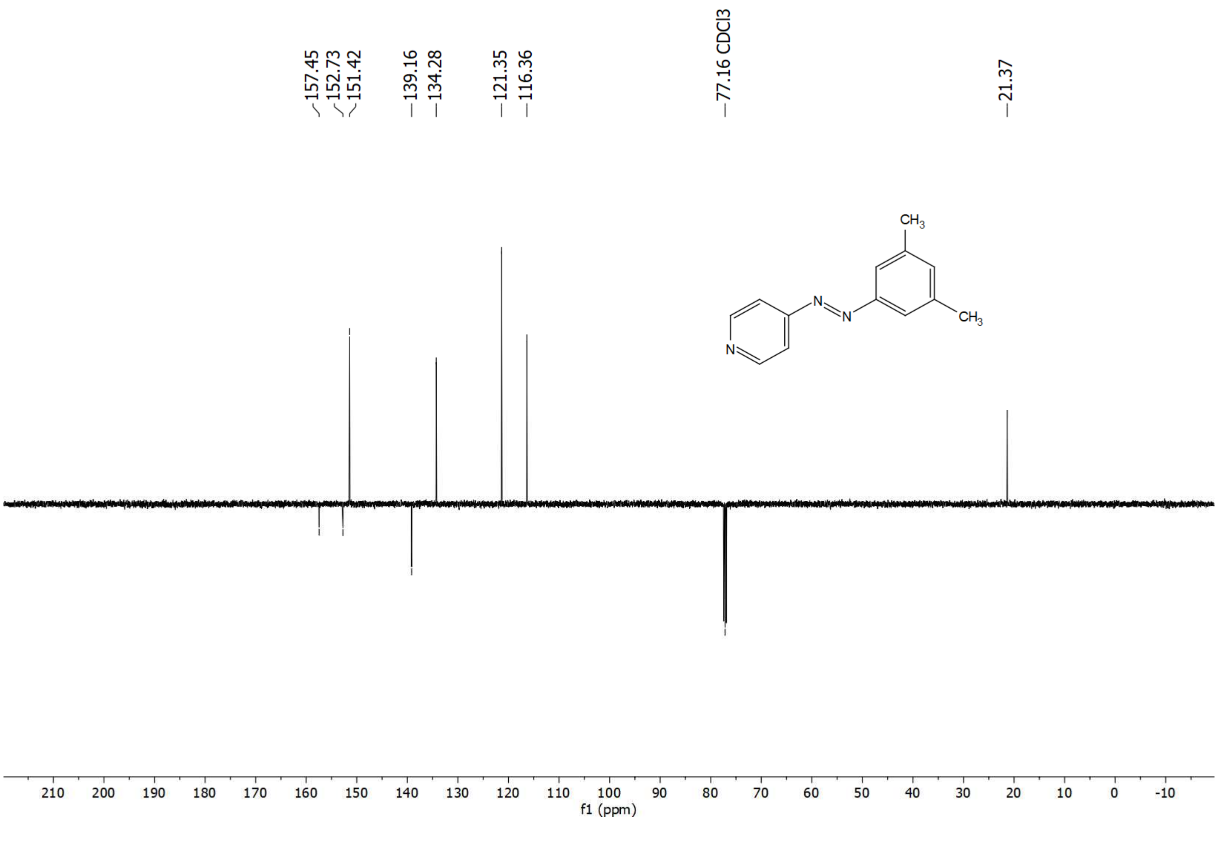

FT-IR (ATR) diamond of **3x**

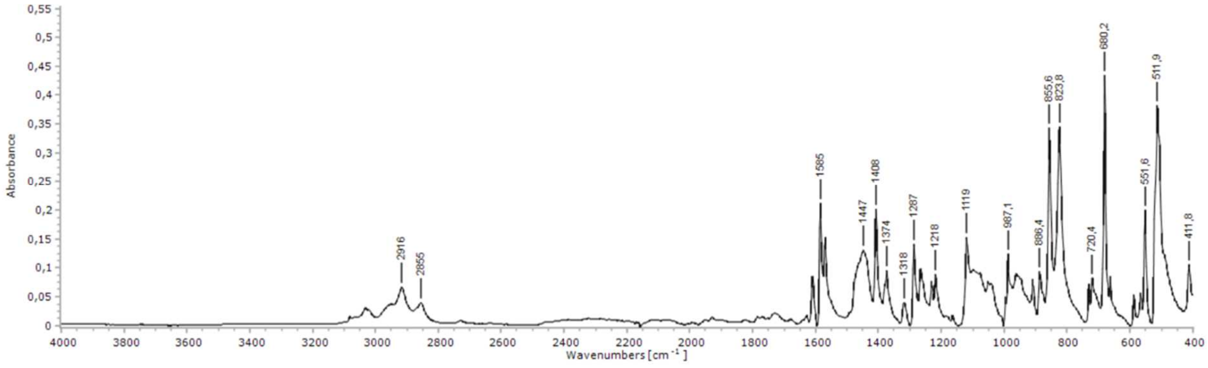

^{1}H NMR (500 MHz, Chloroform-*d*) of **3y**

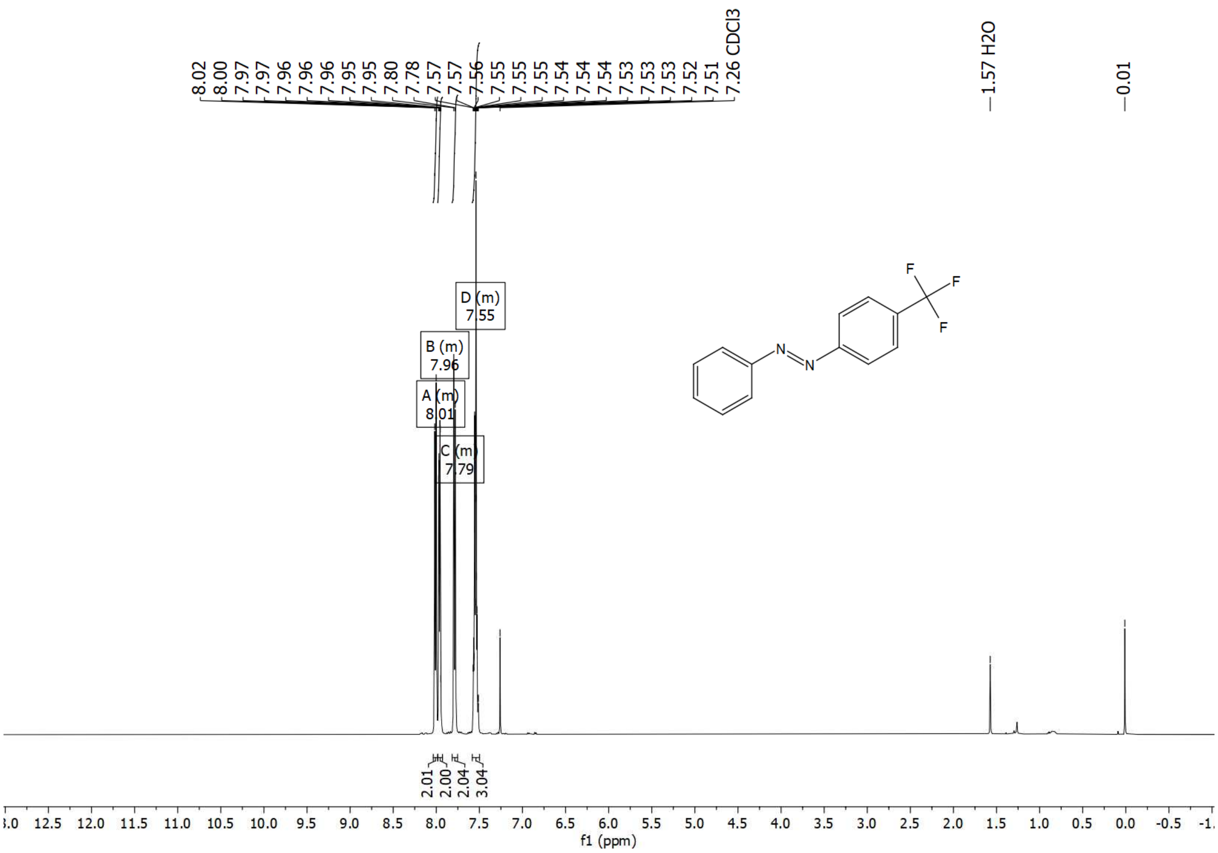

^{13}C APT NMR (126 MHz, $CDCl_3$) of **3y**

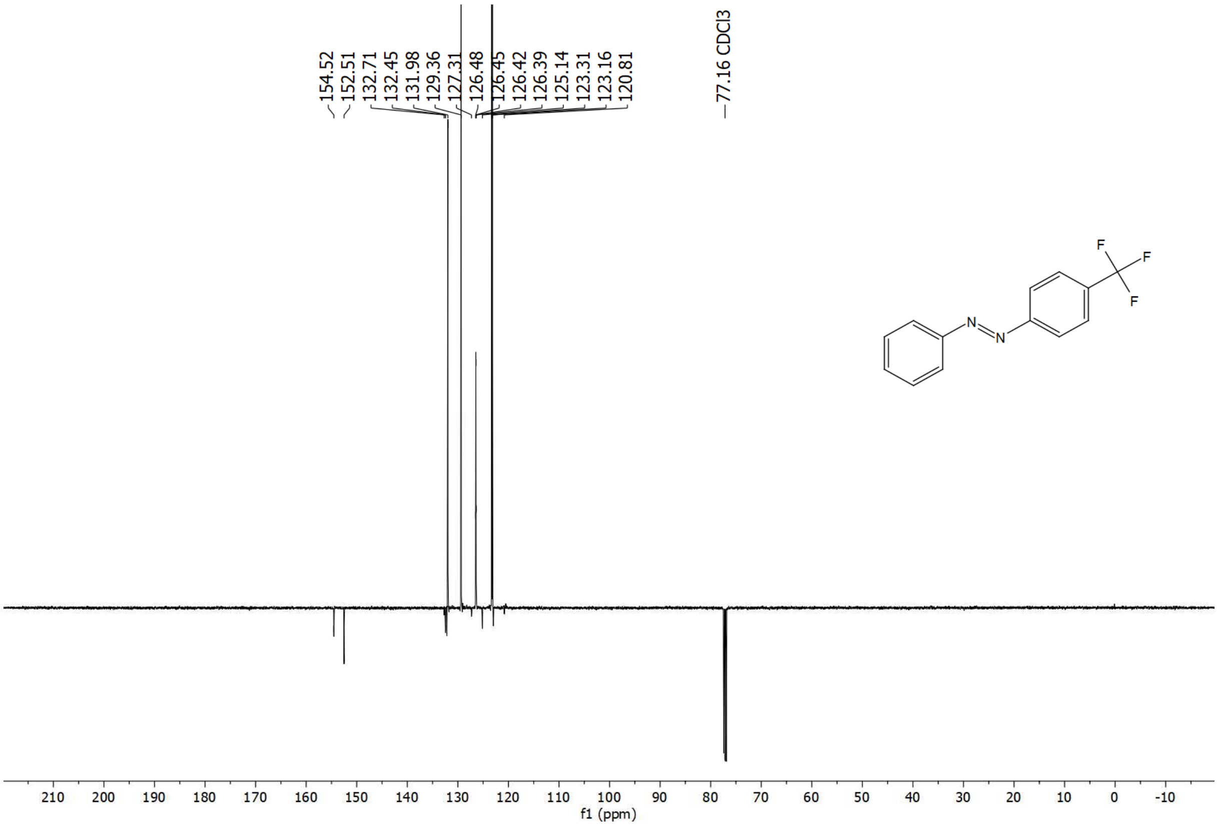

^{19}F{H} NMR (376 MHz, $CDCl_3$) of **3y**

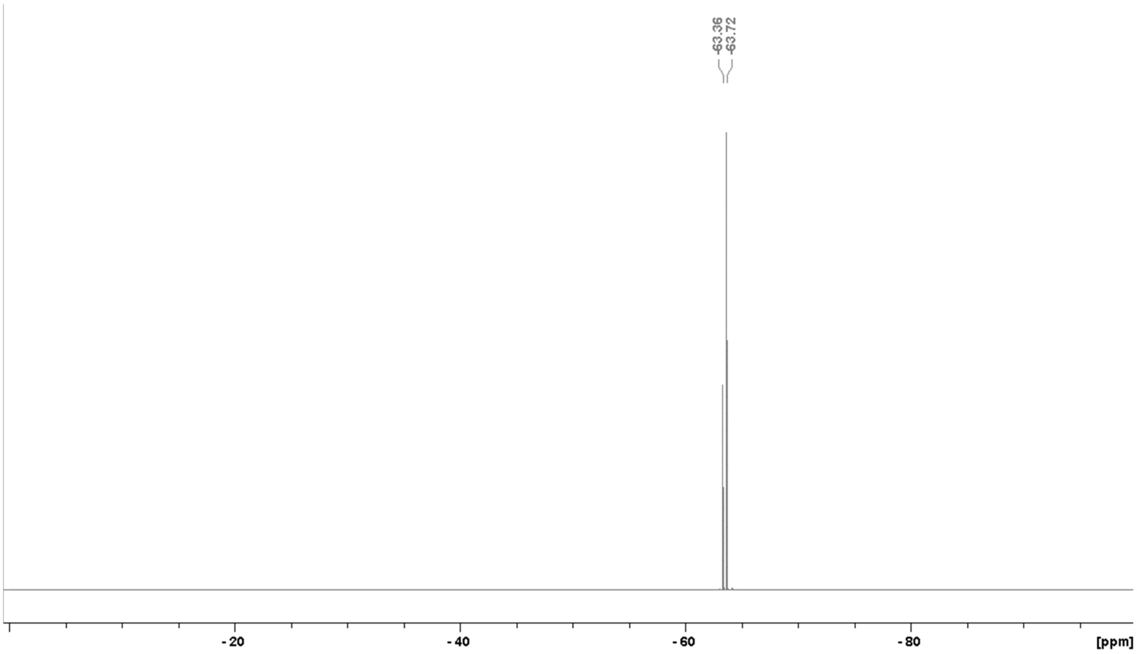

FT-IR (ATR) diamond of **3y**

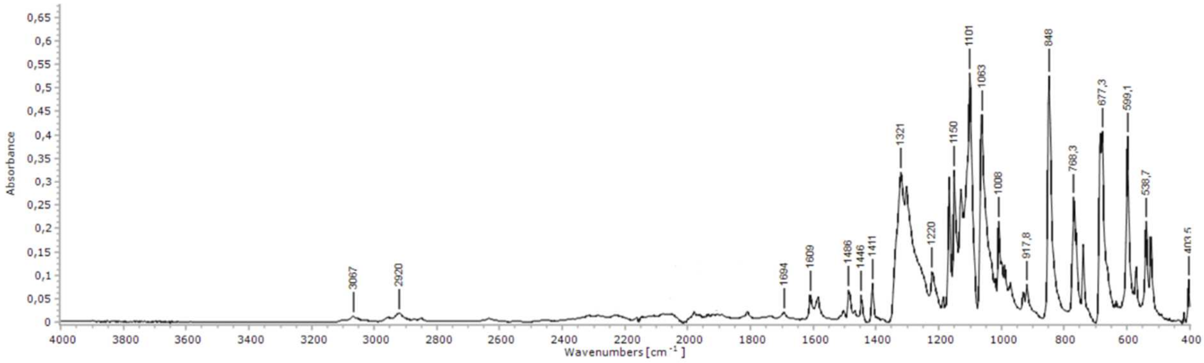

^{1}H NMR (500 MHz, Chloroform-*d*) of **3z**

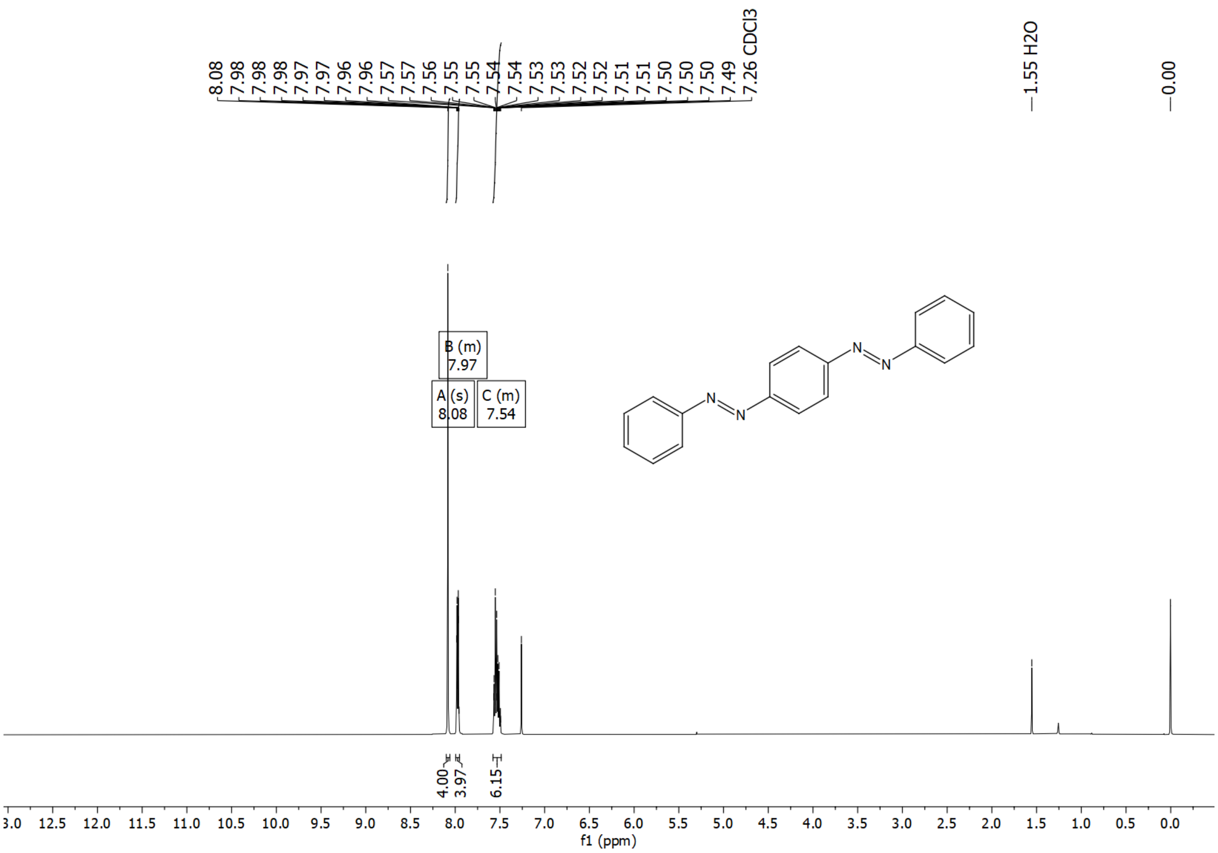

^{13}C APT NMR (126 MHz, $CDCl_3$) of **3z**

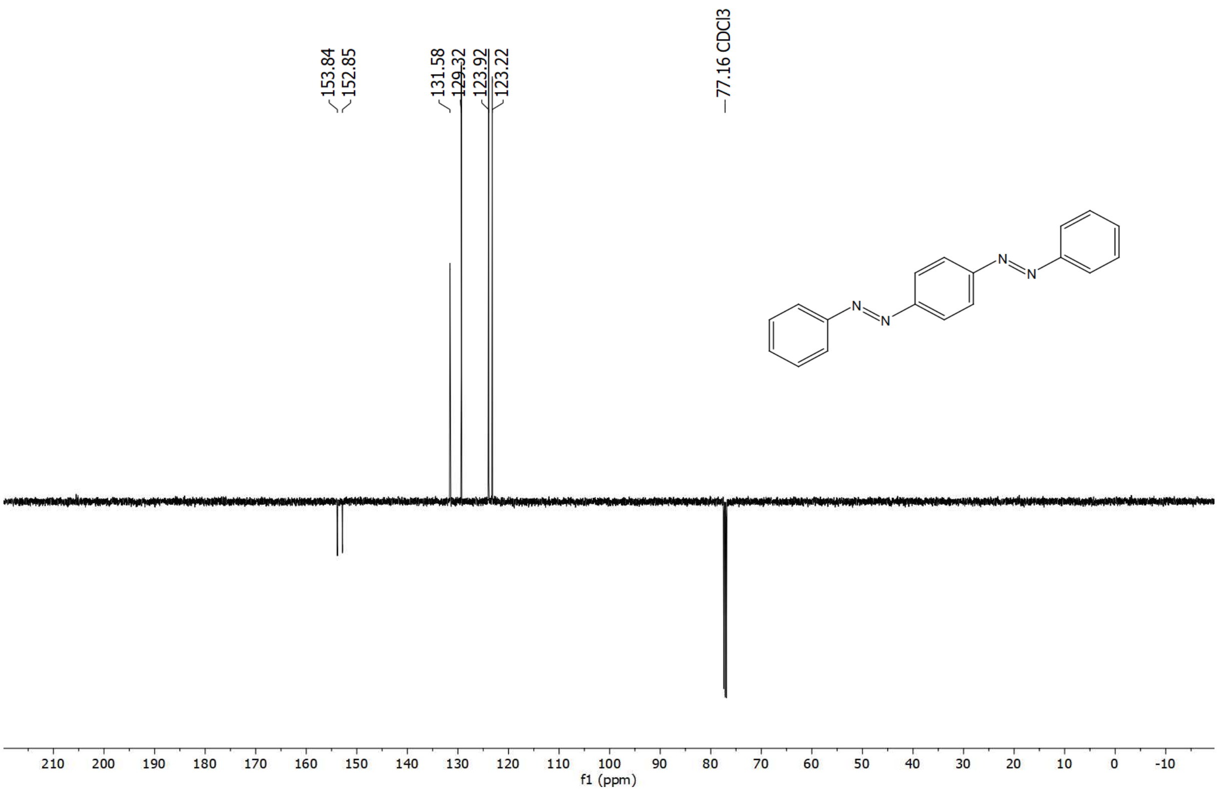

FT-IR (ATR) diamond of **3z**

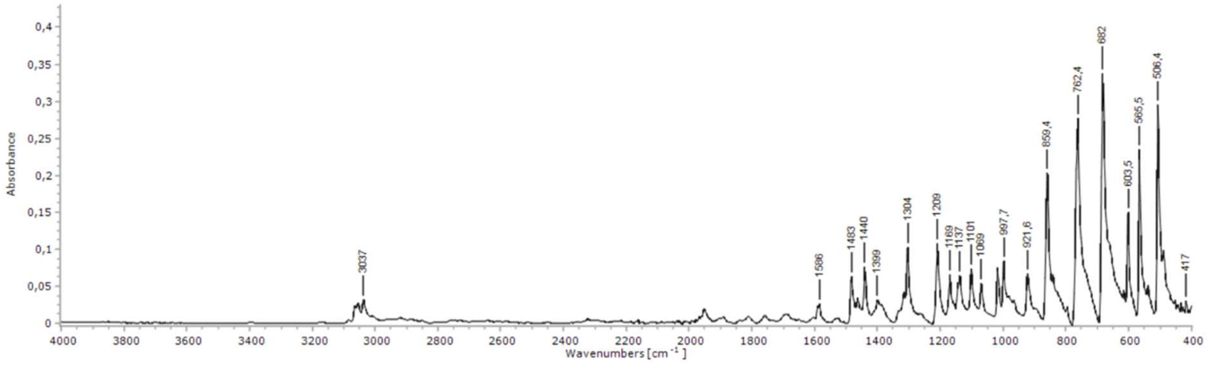

Coordinates

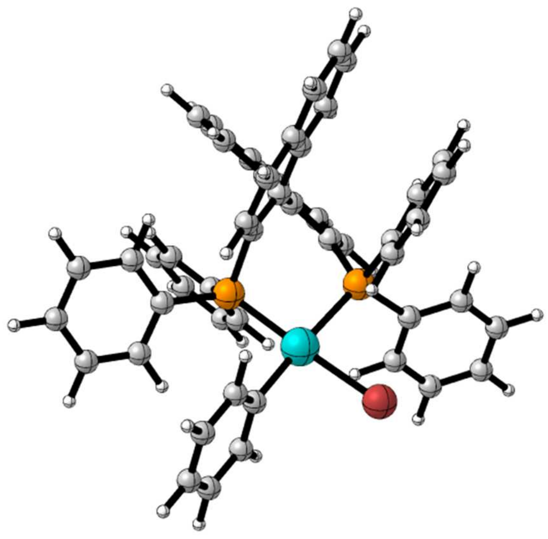

91

Int1 scf done: -5310.150003; Sum of electronic and thermal Free Energies: -5309.515523; imag. freq.: 0; Thermal correction to Gibbs Free Energy: 0.634481; scf(6-311+G**): -5313.3751809

C	4.0049100	-0.2283150	1.6050260
C	3.5961900	0.1515100	0.3209050
C	4.5328320	0.1100020	-0.7208470
C	5.8431970	-0.3246120	-0.4881040
C	6.2393160	-0.7134790	0.7959710
C	5.3164100	-0.6544310	1.8441300
Pd	1.6947500	0.7690110	-0.0376670
C	-0.4510050	-1.2145430	1.2812990
C	-1.7609560	-0.8784970	0.9631520
C	-2.7171180	-0.6639570	2.0153510
C	-2.2876500	-0.7158340	3.3794110
C	-0.9198400	-0.9749830	3.6597280
C	-0.0315880	-1.2238380	2.6456760
C	-3.2287350	-0.4897120	4.4177050
C	-4.5523150	-0.2363530	4.1284970
C	-4.9818820	-0.1867390	2.7813910
C	-4.0860740	-0.3845310	1.7520970
C	-2.1917600	-0.6455180	-0.4502550
C	-1.8150360	0.5215650	-1.1164310
C	-2.2953840	0.7577840	-2.4334950
C	-3.0884120	-0.1615240	-3.0780250
C	-3.4463610	-1.3798970	-2.4497320
C	-3.0083570	-1.6175150	-1.1078760
C	-3.3667670	-2.8457360	-0.4861280
C	-4.1102940	-3.7902100	-1.1596300

C	-4.5370030	-3.5546640	-2.4901900
C	-4.2121560	-2.3722110	-3.1182910
C	-0.5315770	3.0713510	-1.5615400
C	0.2965220	2.9743140	-2.6905510
C	0.3393380	4.0057370	-3.6269060
C	-0.4319550	5.1569900	-3.4346730
C	-1.2424430	5.2680440	-2.3027190
C	-1.2943610	4.2283910	-1.3692540
C	1.8725970	-2.8255660	0.6218500
C	1.3339870	-3.7977510	1.4786350
C	2.0687400	-4.9420330	1.7991760
C	3.3435890	-5.1321250	1.2601870
C	3.8770830	-4.1758870	0.3916020
C	3.1471280	-3.0310030	0.0726710
C	0.2719070	-2.0327340	-1.5468910
C	0.4432830	-1.2732090	-2.7099930
C	0.0475240	-1.7828480	-3.9482710
C	-0.5181540	-3.0569600	-4.0304220
C	-0.6816690	-3.8241900	-2.8723100
C	-0.2815070	-3.3190110	-1.6369120
C	-1.2993170	2.2627000	1.1513440
C	-0.4892910	2.4311740	2.2830570
C	-1.0481780	2.8655920	3.4861460
C	-2.4160070	3.1369420	3.5673830
C	-3.2274200	2.9737110	2.4406000
C	-2.6730950	2.5339010	1.2392040
H	0.5738340	2.2258870	2.2172190
H	-0.4132410	2.9859710	4.3598030
H	-4.2943310	3.1686840	2.5025840
H	-3.3129030	2.3782440	0.3755420
H	0.9241020	2.0976990	-2.8238890
H	0.9892160	3.9210380	-4.4937500
H	-1.8304310	6.1671280	-2.1378860
H	-1.9162650	4.3326280	-0.4868290
H	-2.0206860	1.6719750	-2.9455610
H	-3.4353650	0.0324720	-4.0900510
H	-4.5314280	-2.1827010	-4.1402860
H	-5.1190050	-4.3096870	-3.0117810
H	-4.3684340	-4.7250490	-0.6692480
H	-3.0378260	-3.0388060	0.5292560
H	-4.4284630	-0.3271370	0.7256580
H	-6.0247470	0.0193330	2.5555950
H	-5.2657690	-0.0689210	4.9308210
H	-2.8832640	-0.5213780	5.4481370
H	-0.5830530	-0.9744000	4.6933040
H	1.0092320	-1.4203690	2.8816240
H	0.8835250	-0.2827000	-2.6375010
H	0.1782760	-1.1820760	-4.8442390
H	-1.1252750	-4.8139680	-2.9308460

H	-0.4025160	-3.9242010	-0.7444850
H	3.5757410	-2.2942530	-0.5958110
H	4.8678000	-4.3114240	-0.0330260
H	1.6403850	-5.6839910	2.4680660
H	0.3412420	-3.6729540	1.8973220
H	-2.8516130	3.4666580	4.5068530
H	-0.3888080	5.9684120	-4.1563910
H	-0.8313730	-3.4528780	-4.9927180
H	4.2471440	0.4185660	-1.7236140
H	6.5552580	-0.3537140	-1.3106000
H	7.2568680	-1.0505550	0.9783050
H	5.6129730	-0.9475540	2.8492890
H	3.2999130	-0.2006460	2.4328690
H	3.9164710	-6.0200990	1.5140480
P	0.9010150	-1.3762570	0.0444870
P	-0.5386280	1.6579800	-0.3964000
Br	2.5626630	3.1231050	0.1313450

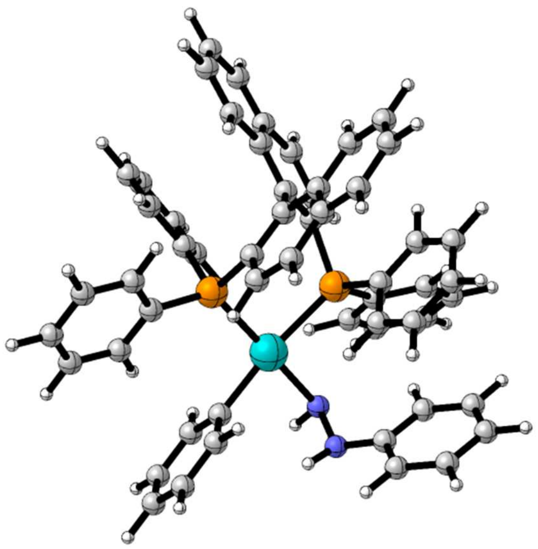

105

Int3 scf done: -3081.042913; Sum of electronic and thermal Free Energies: -3080.289249; imag. freq.: 0; Thermal correction to Gibbs Free Energy: 0.753664; scf(6-311+G**): -3081.6228773

C	2.0636750	3.6576180	-0.9536970
C	1.6266500	3.2240340	0.3102540
C	1.6304680	4.1638700	1.3554950
C	2.0342150	5.4882060	1.1447710
C	2.4564670	5.9025720	-0.1233620
C	2.4751650	4.9787430	-1.1722110
Pd	1.0757500	1.2787160	0.5893550
C	-1.2591030	0.1423230	-1.5052940
C	-1.6655500	-1.1373570	-1.1423840
C	-1.6687760	-2.1898820	-2.1218700

C	-1.1786660	-1.9285680	-3.4405010
C	-0.6940760	-0.6300480	-3.7471560
C	-0.7371490	0.3726000	-2.8132470
C	-1.1626200	-2.9730390	-4.4013360
C	-1.6259430	-4.2319340	-4.0847760
C	-2.1130980	-4.4950720	-2.7828080
C	-2.1253120	-3.5034550	-1.8247490
C	-2.0319670	-1.4795860	0.2680620
C	-1.0432570	-1.6133570	1.2442720
C	-1.4123370	-1.9965700	2.5630690
C	-2.7272390	-2.2066750	2.9030040
C	-3.7597650	-2.0439610	1.9463380
C	-3.4065580	-1.6927160	0.6044160
C	-4.4507570	-1.5285260	-0.3476100
C	-5.7705240	-1.6975950	0.0104480
C	-6.1168290	-2.0387320	1.3411840
C	-5.1291990	-2.2083030	2.2866990
C	1.6131770	-1.7343850	2.3720560
C	1.7575940	-0.8721020	3.4695380
C	2.4199020	-1.2954170	4.6221220
C	2.9695370	-2.5797150	4.6809770
C	2.8492100	-3.4363240	3.5834060
C	2.1726960	-3.0167150	2.4349510
C	-1.6239960	3.0052520	-1.2689420
C	-2.5032450	2.9101530	-2.3601490
C	-2.9827770	4.0620740	-2.9869700
C	-2.5986150	5.3247990	-2.5264800
C	-1.7383680	5.4273820	-1.4306660
C	-1.2552490	4.2766360	-0.8048050
C	-2.4041400	1.4531990	0.9443020
C	-2.0468090	1.3718710	2.2958880
C	-3.0300530	1.3767880	3.2873100
C	-4.3780630	1.4659770	2.9333460
C	-4.7402190	1.5580480	1.5855180
C	-3.7587910	1.5591020	0.5961220
C	1.2316480	-2.0888550	-0.5151130
C	1.9677590	-1.4557740	-1.5252160
C	2.4134310	-2.1786770	-2.6315910
C	2.1217610	-3.5396420	-2.7396120
C	1.3761820	-4.1758140	-1.7421280
C	0.9278940	-3.4530340	-0.6372420
H	2.1889470	-0.3974570	-1.4412940
H	2.9895810	-1.6768730	-3.4028770
H	1.1260710	-5.2289850	-1.8348740
H	0.3180200	-3.9432230	0.1159410
H	1.3617420	0.1371630	3.4111230
H	2.5264450	-0.6156130	5.4633310
H	3.2865090	-4.4308690	3.6158420
H	2.1053900	-3.6848920	1.5841700

H	-0.6442090	-2.1148150	3.3182070
H	-2.9894660	-2.4887180	3.9199040
H	-5.3827680	-2.4665550	3.3121140
H	-7.1617110	-2.1639530	1.6122220
H	-6.5531520	-1.5624830	-0.7314320
H	-4.1979650	-1.2568020	-1.3666030
H	-2.4873130	-3.7270430	-0.8282360
H	-2.4716370	-5.4900040	-2.5323900
H	-1.6118010	-5.0241480	-4.8284430
H	-0.7749030	-2.7593860	-5.3944950
H	-0.2829870	-0.4400750	-4.7354760
H	-0.3572870	1.3563870	-3.0688430
H	-0.9959170	1.2986890	2.5620320
H	-2.7415520	1.3039990	4.3325410
H	-5.7877940	1.6212540	1.3049900
H	-4.0493070	1.6372200	-0.4465050
H	-0.5882010	4.3721290	0.0425070
H	-1.4326090	6.4024450	-1.0614470
H	-3.6594640	3.9703610	-3.8327440
H	-2.8195140	1.9392480	-2.7265180
H	2.4656900	-4.1035530	-3.6026660
H	3.5001150	-2.9056260	5.5717440
H	-5.1453060	1.4625870	3.7029860
H	1.3034080	3.8683120	2.3512950
H	2.0210070	6.1958970	1.9716680
H	2.7700950	6.9302640	-0.2897120
H	2.8005600	5.2869140	-2.1641030
H	2.0679110	2.9631770	-1.7927320
H	-2.9692590	6.2208580	-3.0173630
P	-1.0686890	1.5310510	-0.3137970
P	0.7033990	-1.0866920	0.9179150
N	3.0055420	1.0830100	1.3994690
N	4.0424240	1.2886910	0.4642200
C	4.5990780	0.1813950	-0.1507270
C	5.2175230	0.3095950	-1.4131610
C	4.5867390	-1.0841670	0.4698750
C	5.8105970	-0.7936590	-2.0246770
H	5.2242960	1.2799470	-1.9067010
C	5.1755420	-2.1784560	-0.1584060
H	4.1016830	-1.1828200	1.4319660
C	5.7929930	-2.0512370	-1.4082890
H	6.2804260	-0.6716570	-2.9984250
H	5.1425840	-3.1481840	0.3335990
H	6.2431300	-2.9120520	-1.8950240
H	3.9384950	2.1028380	-0.1404850
H	3.0959840	1.8604990	2.0537800

105

TS4 scf done: -3081.010641; Sum of electronic and thermal Free Energies: -3080.257393; imag. freq.: 1 (-447.7160); Thermal correction to Gibbs Free Energy: 0.753248; scf(6-311+G**): -3081.5905594

C	3.2753430	2.8284390	-0.8422110
C	2.7185040	2.4860060	0.4223580
C	2.6326590	3.5238000	1.3970400
C	3.0788550	4.8099290	1.1187420
C	3.6250260	5.1357430	-0.1369890
C	3.7083480	4.1316170	-1.1061700
Pd	1.4020160	0.8966600	0.4905220
C	-1.2745450	0.4079140	-1.4772840
C	-2.0008140	-0.6992620	-1.0477050
C	-2.3882700	-1.7104000	-1.9948620
C	-1.9568260	-1.6076990	-3.3551710
C	-1.1468010	-0.5055850	-3.7354810
C	-0.8217400	0.4703800	-2.8293380
C	-2.3259710	-2.6141920	-4.2853150
C	-3.1064230	-3.6827670	-3.8990980
C	-3.5370730	-3.7885130	-2.5556950
C	-3.1789010	-2.8339450	-1.6271930
C	-2.3444250	-0.9148460	0.3930790
C	-1.3686600	-1.3347400	1.3004670
C	-1.7367550	-1.5858780	2.6513750
C	-3.0264060	-1.3983770	3.0873830
C	-4.0353500	-0.9470030	2.2000320
C	-3.6938880	-0.7188540	0.8287360
C	-4.7144140	-0.2657980	-0.0530060
C	-5.9981110	-0.0480800	0.3980390
C	-6.3302850	-0.2680750	1.7573850
C	-5.3655700	-0.7083160	2.6373200
C	1.2205480	-2.2163990	2.2344490
C	1.6447570	-1.4223680	3.3136540
C	2.2951840	-1.9947460	4.4075880
C	2.5506380	-3.3693170	4.4277090
C	2.1461690	-4.1642780	3.3515220
C	1.4824900	-3.5929750	2.2627180
C	-0.7592880	3.2391220	-1.3570630
C	-1.7607300	3.4195280	-2.3268960
C	-1.8793910	4.6337940	-3.0038590
C	-1.0016980	5.6856050	-2.7184730
C	-0.0079130	5.5147220	-1.7527010
C	0.1143450	4.2982670	-1.0757110
C	-1.8473690	2.0350900	0.9673990
C	-1.4617180	1.8499230	2.3014810
C	-2.3500370	2.1289280	3.3413950
C	-3.6330000	2.6012050	3.0555260
C	-4.0206490	2.8001930	1.7269800
C	-3.1316680	2.5243890	0.6890670
C	0.5187310	-2.4862440	-0.5925760

C	1.3201120	-2.1122510	-1.6792880
C	1.4228380	-2.9401210	-2.7979060
C	0.7260080	-4.1492600	-2.8391700
C	-0.0790130	-4.5280280	-1.7596770
C	-0.1874680	-3.6979870	-0.6447400
H	1.8633510	-1.1730590	-1.6440660
H	2.0468900	-2.6373040	-3.6338900
H	-0.6404770	-5.4574040	-1.7970100
H	-0.8430680	-3.9782500	0.1746230
H	1.4776600	-0.3487680	3.2842410
H	2.6177100	-1.3661380	5.2334920
H	2.3509550	-5.2318320	3.3552730
H	1.1869830	-4.2207260	1.4295980
H	-0.9843910	-1.9241940	3.3542890
H	-3.2843270	-1.5882910	4.1266700
H	-5.6067960	-0.8776700	3.6841060
H	-7.3447530	-0.0867850	2.1023010
H	-6.7606610	0.3025910	-0.2924790
H	-4.4694590	-0.0821740	-1.0933410
H	-3.5045210	-2.9400340	-0.5992200
H	-4.1467670	-4.6347500	-2.2499840
H	-3.3864830	-4.4461950	-4.6199810
H	-1.9788320	-2.5245350	-5.3119970
H	-0.7843220	-0.4451130	-4.7587650
H	-0.2001410	1.3028400	-3.1422770
H	-0.4632290	1.4777140	2.5142570
H	-2.0415450	1.9700430	4.3713580
H	-5.0197000	3.1606030	1.4983770
H	-3.4436960	2.6849140	-0.3372560
H	0.8883150	4.1777620	-0.3287330
H	0.6831320	6.3213720	-1.5247830
H	-2.6572840	4.7583480	-3.7531080
H	-2.4457970	2.6110570	-2.5618750
H	0.7991070	-4.7915550	-3.7128560
H	3.0708420	-3.8158260	5.2708740
H	-4.3296290	2.8116900	3.8626190
H	2.1997030	3.3060400	2.3719330
H	2.9900690	5.5775580	1.8856670
H	3.9664510	6.1449680	-0.3489450
H	4.0986510	4.3646350	-2.0955490
H	3.3044250	2.0864090	-1.6367000
H	-1.0930870	6.6302930	-3.2483380
P	-0.6087120	1.7046910	-0.3510370
P	0.4222010	-1.3414600	0.8303680
N	3.4362470	0.9238720	1.1576710
N	4.5791330	0.5896040	0.4406640
C	4.6155980	-0.6497640	-0.2072570
C	5.2457070	-0.7743740	-1.4585130
C	4.0783400	-1.7936040	0.4049760

C	5.3516360	-2.0246600	-2.0697060
H	5.6523270	0.1106360	-1.9439980
C	4.1781330	-3.0340960	-0.2209040
H	3.5952710	-1.6932200	1.3677440
C	4.8173020	-3.1641470	-1.4578980
H	5.8434970	-2.1049150	-3.0363780
H	3.7435180	-3.9051560	0.2620050
H	4.8850940	-4.1333490	-1.9440280
H	4.8944920	1.3631520	-0.1548660
H	3.6912040	1.2895920	2.0672730

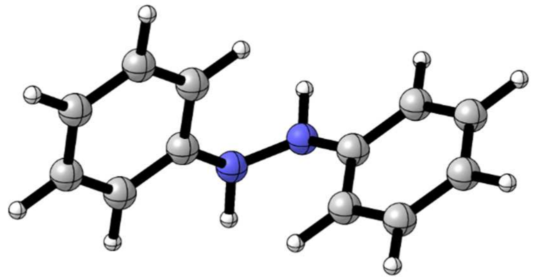

26

hydrazobenzene scf done: -574.061766; Sum of electronic and thermal Free Energies: -573.885065; imag. freq.:0; Thermal correction to Gibbs Free Energy: 0.176701; scf(6-311+G**): -574.1946011

C	-2.3712080	1.1586260	-1.2740750
C	-1.4115970	0.1693970	-1.0487930
C	-1.5423600	-0.6934160	0.0494910
C	-2.6408530	-0.5446450	0.9137690
C	-3.5925930	0.4448280	0.6757800
C	-3.4669090	1.3060120	-0.4200750
H	-2.2530050	1.8232570	-2.1262000
H	-0.5605020	0.0765820	-1.7147400
H	-2.7447990	-1.2110370	1.7678950
H	-4.4357120	0.5452290	1.3547720
H	-4.2076870	2.0795370	-0.6008360
N	-0.6334680	-1.7378680	0.2832390
N	0.6334140	-1.7378010	-0.2838890
C	1.5423280	-0.6934340	-0.0497740
C	1.4116100	0.1689480	1.0488520
C	2.6407860	-0.5443390	-0.9140350
C	2.3712350	1.1580830	1.2744830
H	0.5605410	0.0758700	1.7147950
C	3.5925430	0.4450370	-0.6756990
H	2.7446950	-1.2103990	-1.7684250
C	3.4669040	1.3057940	0.4204960
H	2.2530730	1.8223850	2.1268700
H	4.4356370	0.5456970	-1.3546830
H	4.2076910	2.0792470	0.6015310
H	0.6467400	-2.1356360	-1.2157780
H	-0.6468490	-2.1361230	1.2149450

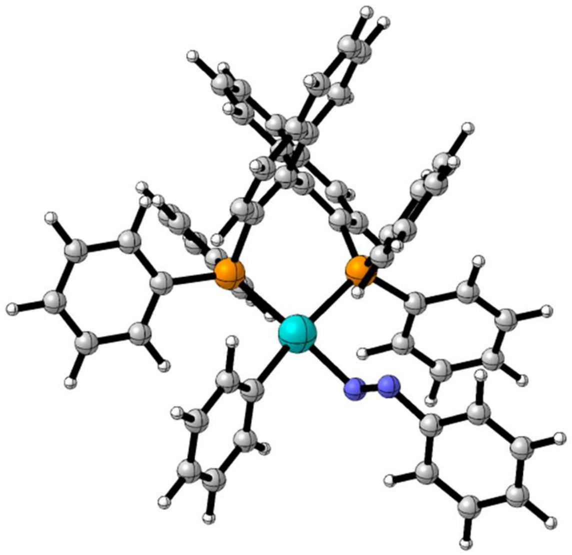

103

Int4 scf done: -3079.826807; Sum of electronic and thermal Free Energies: -3079.102175; imag. freq.:0; Thermal correction to Gibbs Free Energy: 0.724632; scf(6-311+G**): -3080.3995075

C	-1.6156810	3.4814350	1.6297080
C	-1.8511500	2.9254950	0.3616530
C	-2.6234840	3.6704550	-0.5433500
C	-3.1133220	4.9399640	-0.2107180
C	-2.8611920	5.4814240	1.0541690
C	-2.1190050	4.7398750	1.9790310
Pd	-1.1370800	1.0641330	-0.1430510
C	1.7989270	0.4505490	1.2302840
C	2.2067060	-0.8464500	0.9344260
C	2.5694050	-1.7403620	2.0008540
C	2.4259890	-1.3143080	3.3594420
C	1.9324710	-0.0094360	3.6208230
C	1.6375430	0.8467230	2.5914160
C	2.7597320	-2.2064070	4.4121400
C	3.2349830	-3.4716470	4.1427650
C	3.3844060	-3.8957050	2.8011580
C	3.0518020	-3.0563840	1.7591080
C	2.2121700	-1.3826440	-0.4645260
C	1.0188630	-1.7388000	-1.0958490
C	1.0563380	-2.3099310	-2.3970660
C	2.2460910	-2.4865640	-3.0619850
C	3.4738780	-2.1027530	-2.4672830
C	3.4596840	-1.5624990	-1.1416140
C	4.6984100	-1.1855880	-0.5524260
C	5.8834470	-1.3266040	-1.2409250
C	5.8928120	-1.8520680	-2.5566430
C	4.7109640	-2.2325810	-3.1537190
C	-1.8571140	-2.1237250	-1.4144690

C	-2.2966880	-1.4637550	-2.5738350
C	-3.2841280	-2.0294350	-3.3794710
C	-3.8620070	-3.2520600	-3.0235570
C	-3.4430990	-3.9049370	-1.8623280
C	-2.4445860	-3.3447090	-1.0605750
C	1.7378080	3.2731200	0.5111100
C	2.8897990	3.4512740	1.2926060
C	3.3559350	4.7358740	1.5808730
C	2.6828510	5.8551670	1.0822930
C	1.5430990	5.6836780	0.2921690
C	1.0712330	4.4009780	0.0092950
C	2.0687660	1.4294310	-1.5892670
C	1.3681760	1.1116720	-2.7591320
C	2.0408160	1.0131110	-3.9789570
C	3.4181820	1.2365710	-4.0373080
C	4.1207160	1.5672960	-2.8739760
C	3.4488820	1.6699950	-1.6572830
C	-0.6841380	-2.0890760	1.2544120
C	-1.2618500	-1.3932560	2.3259150
C	-1.3319020	-1.9864190	3.5878910
C	-0.8276040	-3.2740830	3.7871570
C	-0.2537080	-3.9725250	2.7202780
C	-0.1805240	-3.3818820	1.4591630
H	-1.6577640	-0.3951680	2.1657220
H	-1.7755600	-1.4378640	4.4144890
H	0.1553280	-4.9669490	2.8745980
H	0.2904270	-3.9153180	0.6385520
H	-1.8845240	-0.4920420	-2.8291880
H	-3.6208620	-1.5028840	-4.2683640
H	-3.9012450	-4.8459870	-1.5695640
H	-2.1438120	-3.8524850	-0.1508300
H	0.1291120	-2.5997030	-2.8779730
H	2.2548890	-2.9140980	-4.0617290
H	4.7054940	-2.6355920	-4.1636670
H	6.8335570	-1.9530290	-3.0911280
H	6.8182890	-1.0276810	-0.7742080
H	4.7016480	-0.7731610	0.4505580
H	3.1585280	-3.4039820	0.7384030
H	3.7554970	-4.8951160	2.5899320
H	3.4906560	-4.1455950	4.9559280
H	2.6328750	-1.8691990	5.4380800
H	1.7887600	0.3043790	4.6517540
H	1.2591830	1.8388670	2.8143310
H	0.2971060	0.9375880	-2.7060600
H	1.4887000	0.7565710	-4.8791780
H	5.1929860	1.7371460	-2.9134100
H	4.0003980	1.9311600	-0.7597630
H	0.1772350	4.2776020	-0.5915440
H	1.0092050	6.5468020	-0.0956350

H	4.2465210	4.8599170	2.1917050
H	3.4266400	2.5915960	1.6808690
H	-0.8731660	-3.7307410	4.7723520
H	-4.6475160	-3.6830070	-3.6384970
H	3.9444560	1.1529760	-4.9844130
H	-2.8558100	3.2540090	-1.5219060
H	-3.6997190	5.5015370	-0.9360560
H	-3.2445430	6.4643050	1.3183430
H	-1.9214650	5.1454410	2.9697750
H	-1.0213320	2.9364380	2.3606720
H	3.0443150	6.8546240	1.3097360
P	1.1324890	1.6228020	-0.0218460
P	-0.6142480	-1.2655620	-0.3745810
N	-3.1006110	0.6221250	-0.4482550
N	-3.7181090	0.0394650	0.4496570
C	-5.0819740	-0.3257740	0.1309540
C	-5.6446390	-1.3423950	0.9067370
C	-5.8264150	0.2858860	-0.8849590
C	-6.9494960	-1.7711390	0.6514690
H	-5.0432410	-1.7909840	1.6932240
C	-7.1337220	-0.1342470	-1.1262670
H	-5.3671750	1.0806350	-1.4646260
C	-7.6968790	-1.1670810	-0.3645250
H	-7.3838460	-2.5706950	1.2463850
H	-7.7200610	0.3444730	-1.9071870
H	-8.7165130	-1.4911180	-0.5572640

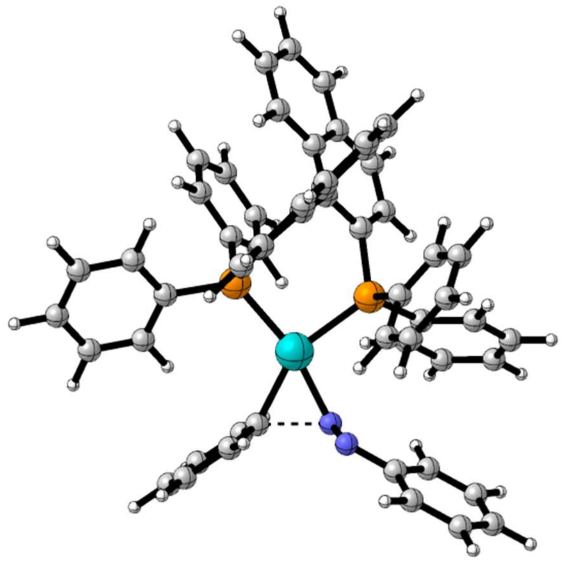

103

TS6 scf done: -3079.794446; Sum of electronic and thermal Free Energies: -3079.069678; imag. freq.:1 (-439.6789); Thermal correction to Gibbs Free Energy: 0.724767; scf(6-311+G**): -3080.3666678

0 1

C	-2.5767720	3.3580670	1.2077020

C	-2.3730820	2.7769130	-0.0564130
C	-2.4695610	3.5732710	-1.2079090
C	-2.6805470	4.9524230	-1.0929680
C	-2.8283440	5.5357890	0.1686590
C	-2.7837540	4.7295790	1.3187780
Pd	-1.0775160	1.0997460	-0.2065120
C	1.7183030	0.3578050	1.3879430
C	2.1044680	-0.9249830	1.0031250
C	2.3929740	-1.9137370	2.0071770
C	2.2100570	-1.5942000	3.3903970
C	1.7424480	-0.2999590	3.7378490
C	1.5097600	0.6436730	2.7702480
C	2.4818000	-2.5762770	4.3794290
C	2.9316860	-3.8302090	4.0262300
C	3.1203500	-4.1497940	2.6605060
C	2.8512870	-3.2200540	1.6788930
C	2.2055430	-1.3383230	-0.4345650
C	1.0643930	-1.6293410	-1.1869880
C	1.2058230	-2.0739250	-2.5306500
C	2.4437260	-2.2016640	-3.1133600
C	3.6222190	-1.8958000	-2.3879590
C	3.5034390	-1.4763730	-1.0242730
C	4.6951630	-1.1801370	-0.3053950
C	5.9317140	-1.2882410	-0.9033720
C	6.0441230	-1.6940720	-2.2558820
C	4.9101360	-1.9903600	-2.9800940
C	-1.7672140	-1.9053020	-1.8097910
C	-2.1318130	-1.0634480	-2.8732200
C	-3.0108710	-1.5073130	-3.8616540
C	-3.5577070	-2.7921360	-3.7842890
C	-3.2185580	-3.6274020	-2.7163850
C	-2.3258540	-3.1885320	-1.7353280
C	1.6420250	3.2367300	0.9884940
C	2.7911760	3.3962610	1.7805610
C	3.1290800	4.6501900	2.2911160
C	2.3261320	5.7618710	2.0117720
C	1.1824970	5.6111970	1.2244120
C	0.8389920	4.3544590	0.7189790
C	2.2758500	1.6064880	-1.2575210
C	1.7049850	1.3967620	-2.5196970
C	2.5029830	1.3856470	-3.6652540
C	3.8806820	1.5893090	-3.5581720
C	4.4556900	1.8107850	-2.3029670
C	3.6576020	1.8233000	-1.1597580
C	-0.8626010	-2.2774910	0.9191690
C	-1.6452960	-1.7704880	1.9673980
C	-1.8823080	-2.5473000	3.1030570
C	-1.3361850	-3.8300730	3.2024640
C	-0.5528840	-4.3375820	2.1613040

C	-0.3175780	-3.5650640	1.0236440
H	-2.0711610	-0.7740190	1.8894850
H	-2.4896710	-2.1468080	3.9104250
H	-0.1130840	-5.3278820	2.2414650
H	0.3047320	-3.9550240	0.2238010
H	-1.7385830	-0.0511350	-2.9115500
H	-3.2847990	-0.8438240	-4.6777420
H	-3.6530410	-4.6208390	-2.6412750
H	-2.0788250	-3.8427740	-0.9059310
H	0.3193220	-2.3075360	-3.1091280
H	2.5304480	-2.5350390	-4.1448380
H	4.9823400	-2.3011360	-4.0197400
H	7.0247400	-1.7694440	-2.7181290
H	6.8282740	-1.0534310	-0.3356140
H	4.6216500	-0.8573350	0.7271300
H	2.9906460	-3.4870090	0.6380590
H	3.4726160	-5.1396710	2.3822240
H	3.1386140	-4.5740820	4.7909090
H	2.3269120	-2.3175830	5.4242890
H	1.5702210	-0.0641610	4.7852920
H	1.1494850	1.6251030	3.0595860
H	0.6337390	1.2314520	-2.5941880
H	2.0490390	1.2097310	-4.6369640
H	5.5280080	1.9606900	-2.2131740
H	4.1160420	1.9903050	-0.1908900
H	-0.0576480	4.2411270	0.1192830
H	0.5481230	6.4664980	1.0085130
H	4.0185280	4.7596280	2.9065410
H	3.4174540	2.5396000	2.0108460
H	-1.5138150	-4.4306740	4.0907730
H	-4.2550630	-3.1345670	-4.5443540
H	4.5056380	1.5723120	-4.4471110
H	-2.3770850	3.1119930	-2.1877520
H	-2.7382790	5.5672980	-1.9883470
H	-2.9991110	6.6053450	0.2598800
H	-2.9148290	5.1787010	2.3008200
H	-2.5820140	2.7250040	2.0912910
H	2.5898810	6.7377360	2.4112130
P	1.1560090	1.6481900	0.1997470
P	-0.6194360	-1.2186470	-0.5506240
N	-3.1482650	1.0914480	-0.3992380
N	-3.7391640	0.6734760	0.6399790
C	-4.8910550	-0.1206440	0.4089660
C	-5.6468200	-0.4597420	1.5432920
C	-5.2638420	-0.6286020	-0.8498670
C	-6.7691370	-1.2794540	1.4228750
H	-5.3345450	-0.0696590	2.5084500
C	-6.3839320	-1.4480420	-0.9615930
H	-4.6582710	-0.3874940	-1.7156160

C	-7.1434070	-1.7772330	0.1697950
H	-7.3506000	-1.5328770	2.3058740
H	-6.6620810	-1.8448870	-1.9351030
H	-8.0127020	-2.4228980	0.0753290

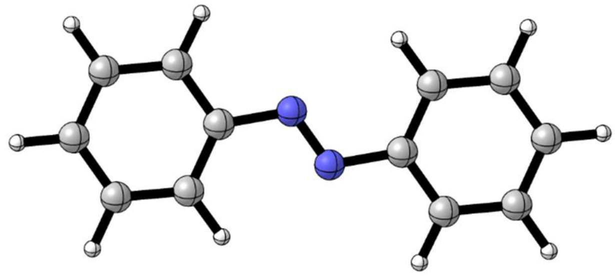

24

azobenzene scf done: -572.845479; Sum of electronic and thermal Free Energies: -572.693052; imag. freq.:0; Thermal correction to Gibbs Free Energy: 0.152427; scf(6-311+G**): -572.969005

C	3.6735360	1.3053950	-0.0000750
C	2.2947390	1.1217700	-0.0000830
C	1.7700760	-0.1834980	-0.0000070
C	2.6341890	-1.2876450	0.0000680
C	4.0162650	-1.0951680	0.0000830
C	4.5380170	0.2008470	0.0000120
H	4.0827550	2.3123050	-0.0001380
H	1.6160440	1.9674870	-0.0001530
H	2.2036850	-2.2850030	0.0001170
H	4.6829990	-1.9530490	0.0001470
H	5.6137290	0.3549450	0.0000180
N	0.3879350	-0.4951890	-0.0000140
N	-0.3879330	0.4951760	-0.0000060
C	-1.7700730	0.1834860	-0.0000040
C	-2.2947420	-1.1217810	-0.0000780
C	-2.6341820	1.2876370	0.0000720
C	-3.6735410	-1.3053990	-0.0000700
H	-1.6160520	-1.9675000	-0.0001410
C	-4.0162570	1.0951650	0.0000810
H	-2.2036710	2.2849920	0.0001260
C	-4.5380160	-0.2008470	0.0000090
H	-4.0827650	-2.3123070	-0.0001290
H	-4.6829880	1.9530490	0.0001410
H	-5.6137290	-0.3549400	0.0000130

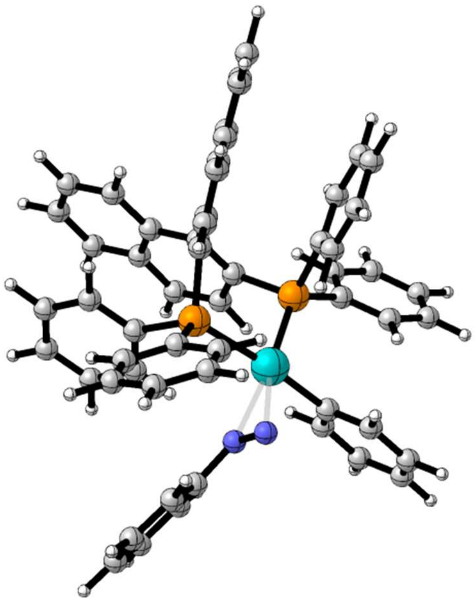

103

TS7 scf done: -3079.780117; Sum of electronic and thermal Free Energies: -3079.054493; imag. freq.:1 -309.3300); Thermal correction to Gibbs Free Energy: 0.725624; scf(6-311+G**): -3080.3534504

C	-1.7605850	3.5531790	1.3192180
C	-1.6576850	3.0790220	0.0056760
C	-2.0371580	3.9213950	-1.0472210
C	-2.4986600	5.2185110	-0.7902320
C	-2.5936270	5.6868100	0.5243620
C	-2.2308170	4.8455270	1.5800790
Pd	-1.1341290	1.1519050	-0.3659840
C	1.4147640	0.2627400	1.4142470
C	1.8871930	-1.0037000	1.0833130
C	2.0651790	-1.9901090	2.1161510
C	1.6843120	-1.6819100	3.4605800
C	1.1278330	-0.4049420	3.7410560
C	0.9983850	0.5344820	2.7525720
C	1.8409870	-2.6610400	4.4748270
C	2.3665550	-3.9027070	4.1849310
C	2.7441470	-4.2126380	2.8580640
C	2.5883200	-3.2847810	1.8491130
C	2.1196560	-1.4174680	-0.3357140
C	1.0377310	-1.6744140	-1.1808120
C	1.2760810	-2.1423430	-2.5011890
C	2.5547670	-2.3104070	-2.9762670
C	3.6767200	-2.0183270	-2.1612040
C	3.4569720	-1.5847370	-0.8148980
C	4.5893070	-1.2932000	-0.0049680
C	5.8680880	-1.4178180	-0.5025340
C	6.0821090	-1.8407320	-1.8377430
C	5.0066000	-2.1356560	-2.6468610
C	-1.7624910	-1.9560220	-1.9557460

C	-1.9004700	-1.2793570	-3.1788910
C	-2.7372750	-1.7812820	-4.1744400
C	-3.4632880	-2.9565400	-3.9540060
C	-3.3483000	-3.6223420	-2.7321300
C	-2.5036790	-3.1244930	-1.7358040
C	1.5870370	3.1332960	0.9283090
C	2.4841000	3.1777270	2.0055440
C	2.9900840	4.4010420	2.4549170
C	2.6181620	5.5903200	1.8249270
C	1.7445750	5.5497700	0.7339070
C	1.2350240	4.3316680	0.2874990
C	2.1654800	1.4610680	-1.2374960
C	1.6677730	1.2877430	-2.5331980
C	2.5372840	1.2836600	-3.6260180
C	3.9086140	1.4605330	-3.4294520
C	4.4088880	1.6490280	-2.1369360
C	3.5415460	1.6562150	-1.0463700
C	-0.9963730	-2.1908290	0.8360090
C	-1.7218490	-1.6046670	1.8803090
C	-2.0017180	-2.3342810	3.0373760
C	-1.5563970	-3.6521580	3.1589620
C	-0.8306030	-4.2422900	2.1188320
C	-0.5500770	-3.5149470	0.9634040
H	-2.0612480	-0.5787680	1.7803200
H	-2.5630220	-1.8688950	3.8432010
H	-0.4692790	-5.2622250	2.2150190
H	0.0303400	-3.9693880	0.1656560
H	-1.3743210	-0.3434860	-3.3377700
H	-2.8405090	-1.2411950	-5.1115280
H	-3.9238570	-4.5248550	-2.5439190
H	-2.4400370	-3.6423900	-0.7856730
H	0.4362510	-2.3626440	-3.1492500
H	2.7172590	-2.6596970	-3.9930360
H	5.1588160	-2.4586120	-3.6739400
H	7.0953690	-1.9301080	-2.2200790
H	6.7196640	-1.1840380	0.1309270
H	4.4357270	-0.9584290	1.0150980
H	2.8682270	-3.5463100	0.8356730
H	3.1519450	-5.1934840	2.6283650
H	2.4852220	-4.6444970	4.9701030
H	1.5364240	-2.4122040	5.4885810
H	0.7975720	-0.1841510	4.7530040
H	0.5553110	1.4971860	2.9852750
H	0.5983720	1.1569640	-2.6756120
H	2.1412930	1.1398290	-4.6278130
H	5.4750470	1.7832610	-1.9778130
H	3.9369410	1.8087860	-0.0471830
H	0.5566350	4.3152530	-0.5569880
H	1.4505440	6.4670270	0.2313950

H	3.6799480	4.4181030	3.2947510
H	2.8019450	2.2655550	2.4975730
H	-1.7669750	-4.2178130	4.0627370
H	-4.1268390	-3.3399680	-4.7246870
H	4.5868540	1.4527200	-4.2785800
H	-1.9822530	3.5650870	-2.0718660
H	-2.7877770	5.8604790	-1.6199530
H	-2.9516250	6.6940160	0.7235250
H	-2.3038410	5.1956660	2.6078130
H	-1.4667180	2.9195210	2.1526420
H	3.0092010	6.5410430	2.1774700
P	0.9963440	1.5589670	0.1794490
P	-0.6798060	-1.2103000	-0.6757870
N	-3.1516880	0.9450950	-1.6649580
N	-3.3168060	0.6185410	-0.4772170
C	-4.5576600	0.0127400	-0.0273980
C	-4.9146960	0.1730430	1.3121030
C	-5.3153390	-0.7927840	-0.8808770
C	-6.0642630	-0.4523280	1.7987510
H	-4.2912760	0.7942080	1.9513570
C	-6.4513870	-1.4343480	-0.3852890
H	-4.9915820	-0.9097000	-1.9111620
C	-6.8309830	-1.2622370	0.9518340
H	-6.3605550	-0.3149020	2.8358700
H	-7.0393850	-2.0758390	-1.0379160
H	-7.7158540	-1.7642920	1.3349310

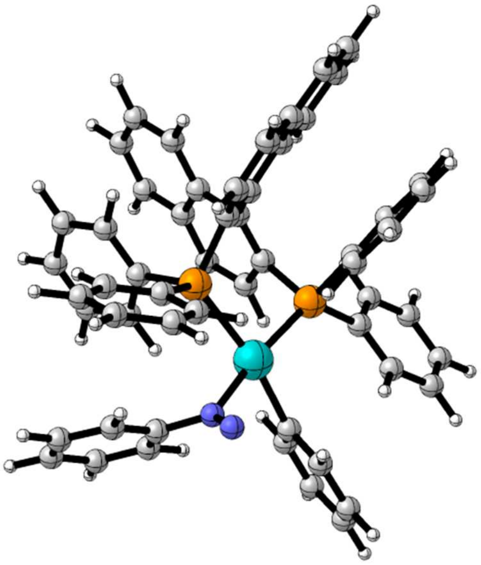

103

Int7 scf done-3079.767156; Sum of electronic and thermal Free Energies: -3079.038258; imag. freq.:0; Thermal correction to Gibbs Free Energy: 0.728898; scf(6-311+G**): -3080.3896553 geometry taken from gas phase calculations

C	2.2422720	3.2778550	-1.3517160
C	2.2496670	2.7587180	-0.0472860
C	2.8074930	3.5439570	0.9699040

C	3.3142840	4.8214100	0.7021690
C	3.2923860	5.3289300	-0.6015470
C	2.7638180	4.5459800	-1.6318100
Pd	1.3750670	0.9454480	0.4165620
C	-1.3828890	0.5110180	-1.3682870
C	-1.9437360	-0.7313420	-1.0903050
C	-2.2462900	-1.6360450	-2.1654870
C	-1.8855940	-1.2866860	-3.5049770
C	-1.2365620	-0.0457930	-3.7376020
C	-0.9996280	0.8260320	-2.7066350
C	-2.1674340	-2.1888140	-4.5631950
C	-2.7976660	-3.3900920	-4.3200990
C	-3.1609330	-3.7378530	-2.9979940
C	-2.8837630	-2.8883880	-1.9483510
C	-2.1913680	-1.1944850	0.3113820
C	-1.1372480	-1.6205520	1.1214630
C	-1.4164000	-2.1211010	2.4222640
C	-2.6992330	-2.1536930	2.9134660
C	-3.7876600	-1.6887270	2.1353290
C	-3.5334880	-1.2244320	0.8062320
C	-4.6326090	-0.7644590	0.0295530
C	-5.9101980	-0.7548470	0.5443670
C	-6.1568350	-1.2058900	1.8643760
C	-5.1149400	-1.6647510	2.6397810
C	1.5877660	-2.3247520	1.8299760
C	1.8394750	-1.7301050	3.0777280
C	2.6133720	-2.3876680	4.0329000
C	3.1536930	-3.6459460	3.7502810
C	2.9174630	-4.2386360	2.5072930
C	2.1418820	-3.5810430	1.5498540
C	-1.1875870	3.3410720	-0.7492270
C	-2.1897280	3.5626390	-1.7066540
C	-2.5077480	4.8613290	-2.1092560
C	-1.8352900	5.9528570	-1.5521840
C	-0.8473210	5.7391020	-0.5878910
C	-0.5236430	4.4421680	-0.1882250
C	-1.9539830	1.6341920	1.3498780
C	-1.4505350	1.3164120	2.6170490
C	-2.2913440	1.3248350	3.7316730
C	-3.6400900	1.6556020	3.5871650
C	-4.1448770	1.9890680	2.3264770
C	-3.3048230	1.9865570	1.2150760
C	0.7749310	-2.2643680	-0.9615880
C	1.4844240	-1.6777720	-2.0161230
C	1.6216700	-2.3494120	-3.2317110
C	1.0433990	-3.6080370	-3.4048050
C	0.3282410	-4.1971850	-2.3571240
C	0.1933170	-3.5286910	-1.1420570
H	1.9190710	-0.6937330	-1.8802660

H	2.1731440	-1.8828220	-4.0433490
H	-0.1420920	-5.1665030	-2.4946900
H	-0.3862190	-3.9758900	-0.3393570
H	1.4635390	-0.7336050	3.2878510
H	2.8159510	-1.9038750	4.9839700
H	3.3476240	-5.2091450	2.2742740
H	1.9919240	-4.0388170	0.5789230
H	-0.6018280	-2.4741420	3.0428490
H	-2.8895930	-2.5287600	3.9162460
H	-5.2919640	-2.0120400	3.6548850
H	-7.1681740	-1.1884490	2.2612640
H	-6.7351130	-0.3937630	-0.0639250
H	-4.4510890	-0.4074820	-0.9782620
H	-3.1552990	-3.1758090	-0.9394500
H	-3.6550660	-4.6866190	-2.8065720
H	-3.0131050	-4.0713310	-5.1386900
H	-1.8765940	-1.9114670	-5.5736290
H	-0.9287440	0.2069370	-4.7493390
H	-0.5027640	1.7698860	-2.9061350
H	-0.3993450	1.0620820	2.7226220
H	-1.8912980	1.0694680	4.7091230
H	-5.1946530	2.2414880	2.2078520
H	-3.7018680	2.2529030	0.2408620
H	0.2568990	4.2875060	0.5476500
H	-0.3125910	6.5787740	-0.1534180
H	-3.2827820	5.0182760	-2.8550890
H	-2.7236820	2.7248110	-2.1436860
H	1.1384260	-4.1263870	-4.3550550
H	3.7676630	-4.1536630	4.4892720
H	-4.2973250	1.6551280	4.4525120
H	2.8562670	3.1492790	1.9843260
H	3.7333940	5.4175150	1.5106610
H	3.6881180	6.3194810	-0.8124640
H	2.7433790	4.9267080	-2.6512680
H	1.8082990	2.7002070	-2.1667360
H	-2.0798960	6.9631440	-1.8693610
P	-0.7967300	1.6778780	-0.0750910
P	0.6246900	-1.3470930	0.6132300
N	3.5579890	0.6507940	2.3302990
N	3.2837620	0.4417850	1.1783110
C	4.1527900	-0.3208990	0.2762580
C	4.3074410	0.1070920	-1.0395110
C	4.7610950	-1.4844540	0.7425280
C	5.0999830	-0.6453770	-1.9085240
H	3.8195430	1.0225440	-1.3583720
C	5.5201250	-2.2517610	-0.1432320
H	4.6196540	-1.7763510	1.7775670
C	5.6936720	-1.8328160	-1.4667890
H	5.2496450	-0.3079590	-2.9309910

H	5.9742900	-3.1779790	0.1995090
H	6.2902190	-2.4297010	-2.1517280

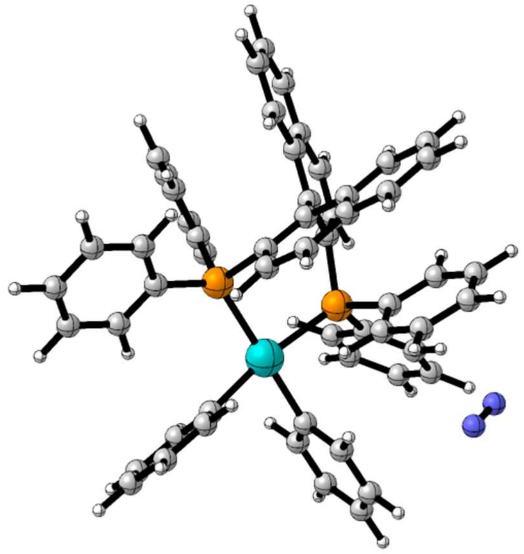

103

int5+N2 scf done: -3079.878431; Sum of electronic and thermal Free Energies: -3079.155961; imag. freq.:0; Thermal correction to Gibbs Free Energy: 0.722470; scf(6-311+G**): -3080.455782

C	2.1452240	3.2493630	-1.7449900
C	2.1739780	2.8336660	-0.4033550
C	2.7162400	3.7235590	0.5382140
C	3.1745900	4.9926660	0.1649030
C	3.1200140	5.3958660	-1.1735860
C	2.6105720	4.5121170	-2.1297630
Pd	1.3655560	1.0344760	0.1366660
C	-1.5483010	0.4829590	-1.2981100
C	-1.9830200	-0.7911150	-0.9486200
C	-2.3160150	-1.7401060	-1.9774110
C	-2.1665450	-1.3743610	-3.3529160
C	-1.6584190	-0.0868700	-3.6673210
C	-1.3544610	0.8088100	-2.6740590
C	-2.5051710	-2.3074630	-4.3675480
C	-2.9784990	-3.5610030	-4.0447520
C	-3.1184810	-3.9316280	-2.6864620
C	-2.7895710	-3.0479260	-1.6805640
C	-2.1066120	-1.2343110	0.4769750
C	-0.9867380	-1.5842260	1.2327410
C	-1.1674640	-2.1052970	2.5431200
C	-2.4172040	-2.2146830	3.1036520
C	-3.5709130	-1.8098190	2.3886910
C	-3.4166600	-1.3368650	1.0468430
C	-4.5832680	-0.9441820	0.3331820
C	-5.8284790	-1.0034530	0.9202050
C	-5.9752700	-1.4625020	2.2525170

C	-4.8670850	-1.8594290	2.9681950
C	1.8292390	-1.9723660	1.8567450
C	1.9190190	-1.3391160	3.1082020
C	2.7975510	-1.8098090	4.0814830
C	3.6266340	-2.9025800	3.8059210
C	3.5648990	-3.5176490	2.5547300
C	2.6673210	-3.0586930	1.5858250
C	-1.4294520	3.3320030	-0.7622460
C	-2.5858740	3.4874170	-1.5439220
C	-2.9961470	4.7565190	-1.9555800
C	-2.2604280	5.8867560	-1.5846410
C	-1.1153310	5.7400550	-0.7984710
C	-0.7007280	4.4705910	-0.3896350
C	-1.9142840	1.6411830	1.4454140
C	-1.2632130	1.3969430	2.6608450
C	-1.9804360	1.3925250	3.8589290
C	-3.3553480	1.6383160	3.8497260
C	-4.0092700	1.8952180	2.6403470
C	-3.2919870	1.9027720	1.4451800
C	0.8300620	-2.2641580	-0.8950480
C	1.1758540	-1.6583540	-2.1092390
C	1.1850190	-2.4014090	-3.2902620
C	0.8512330	-3.7567370	-3.2659200
C	0.5099520	-4.3691770	-2.0561490
C	0.4919040	-3.6254160	-0.8762900
H	1.4088900	-0.5988860	-2.1250450
H	1.4411910	-1.9169380	-4.2284430
H	0.2387450	-5.4211150	-2.0345640
H	0.1957480	-4.0981520	0.0561270
H	1.3044820	-0.4674380	3.3173610
H	2.8516990	-1.3108730	5.0455780
H	4.2194540	-4.3535920	2.3227780
H	2.6400060	-3.5405170	0.6164850
H	-0.3048720	-2.4180170	3.1175490
H	-2.5302660	-2.6064120	4.1117640
H	-4.9658140	-2.2139110	3.9914690
H	-6.9624660	-1.5006610	2.7051350
H	-6.7054200	-0.6921310	0.3587590
H	-4.4839100	-0.5833170	-0.6844230
H	-2.8902180	-3.3531740	-0.6456920
H	-3.4794040	-4.9249460	-2.4333380
H	-3.2368600	-4.2678140	-4.8286960
H	-2.3800400	-2.0130960	-5.4069410
H	-1.5089060	0.1818590	-4.7103360
H	-0.9592160	1.7839940	-2.9382620
H	-0.1934450	1.2055840	2.6582360
H	-1.4659030	1.1931880	4.7953060
H	-5.0796170	2.0806660	2.6272840
H	-3.8079230	2.1046850	0.5124170

H	0.1969780	4.3673170	0.2081330
H	-0.5317290	6.6098460	-0.5098230
H	-3.8908800	4.8613610	-2.5640050
H	-3.1670260	2.6192350	-1.8387840
H	0.8490330	-4.3341170	-4.1864050
H	4.3258040	-3.2603590	4.5569810
H	-3.9172780	1.6289180	4.7798810
H	2.7830840	3.4304640	1.5839410
H	3.5806950	5.6639610	0.9195960
H	3.4773610	6.3799470	-1.4676990
H	2.5677100	4.8078750	-3.1765250
H	1.7428210	2.5872070	-2.5097580
H	-2.5780930	6.8742970	-1.9090800
P	-0.8992900	1.7072650	-0.0842130
P	0.7225340	-1.2226150	0.6043000
N	4.1976760	-3.2536430	-2.2259840
N	3.9743310	-4.0681640	-1.5136210
C	3.3408170	0.4690940	0.2581220
C	4.0174650	0.5882390	1.4833640
C	4.0586840	-0.0697950	-0.8209810
C	5.3409060	0.1637790	1.6374890
H	3.5063820	1.0117760	2.3450990
C	5.3853020	-0.4964070	-0.6787440
H	3.5898820	-0.1644520	-1.7966380
C	6.0331090	-0.3868850	0.5542090
H	5.8294360	0.2596960	2.6054870
H	5.9101920	-0.9143590	-1.5356310
H	7.0619290	-0.7203750	0.6688930

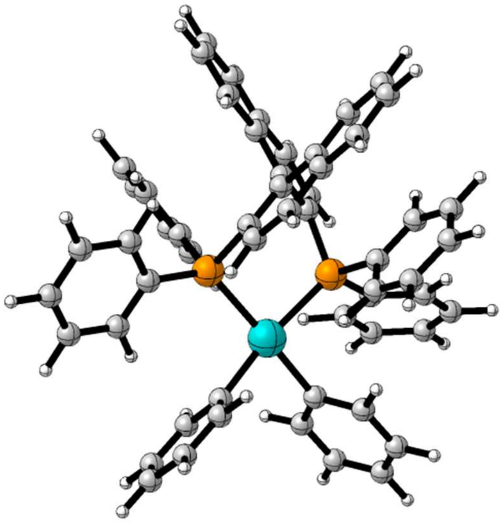

101

int5 scf done: -2970.348549; Sum of electronic and thermal Free Energies: -2969.627304; imag. freq.:0; Thermal correction to Gibbs Free Energy: 0.721245; scf(6-311+G**): -2970.8937971

C	-3.2295790	2.3271900	1.7284540
C	-3.0741060	1.8556090	0.4143380
C	-3.7947880	2.5108670	-0.5971760
C	-4.6146650	3.6106820	-0.3167700
C	-4.7445180	4.0752630	0.9961510
C	-4.0516210	3.4219220	2.0200270
Pd	-1.7536800	0.3461700	0.0183060
C	1.3491440	0.9294450	1.1765300
C	2.1853460	-0.1212810	0.8172900
C	2.9749900	-0.7825890	1.8211010
C	2.8418100	-0.3961500	3.1923720
C	1.9207550	0.6303770	3.5281160
C	1.2008840	1.2725210	2.5537050
C	3.6104560	-1.0590920	4.1847370
C	4.4916400	-2.0623780	3.8431970
C	4.6275170	-2.4478180	2.4887130
C	3.8832120	-1.8304120	1.5057090
C	2.2581030	-0.6344330	-0.5871360
C	1.2374390	-1.4262130	-1.1164490
C	1.3803850	-1.9616650	-2.4258920
C	2.4825330	-1.6799770	-3.1968680
C	3.5155180	-0.8425170	-2.7085990
C	3.4127450	-0.3293830	-1.3766940
C	4.4578630	0.5062520	-0.8934150
C	5.5381100	0.8230100	-1.6878190
C	5.6316650	0.3204340	-3.0091110
C	4.6397800	-0.4967660	-3.5053620
C	-1.2251260	-2.9674320	-1.1414200
C	-1.8682430	-2.6305690	-2.3434750
C	-2.5392210	-3.5994350	-3.0872910
C	-2.6002040	-4.9177320	-2.6254200
C	-1.9874870	-5.2555680	-1.4173130
C	-1.3020170	-4.2866350	-0.6787100
C	0.2160900	3.5100460	0.5639110
C	1.3451140	4.0795020	1.1770300
C	1.3726480	5.4399650	1.4875390
C	0.2754110	6.2522670	1.1837060
C	-0.8452170	5.6955130	0.5639250
C	-0.8757900	4.3334820	0.2550710
C	0.9589970	1.9051340	-1.6372600
C	0.3084920	1.3243750	-2.7335540
C	0.8326620	1.4606810	-4.0205540
C	2.0101870	2.1842640	-4.2216430
C	2.6578940	2.7767970	-3.1329780
C	2.1320760	2.6440290	-1.8489770
C	0.0718560	-2.3195500	1.3933450
C	-0.4989480	-1.7712320	2.5494160
C	-0.1792550	-2.2862920	3.8067230
C	0.7202320	-3.3487410	3.9187300

C	1.3041390	-3.8921170	2.7699980
C	0.9852200	-3.3779810	1.5139820
H	-1.1766290	-0.9275990	2.4585080
H	-0.6215620	-1.8464460	4.6965670
H	2.0228850	-4.7023300	2.8541390
H	1.4626440	-3.7847770	0.6271840
H	-1.8549220	-1.6013100	-2.6893330
H	-3.0358910	-3.3196790	-4.0124540
H	-2.0475260	-6.2731030	-1.0400320
H	-0.8475190	-4.5636200	0.2652990
H	0.6034720	-2.5986210	-2.8302330
H	2.5665130	-2.0918020	-4.1997850
H	4.6993360	-0.8873820	-4.5184250
H	6.4861980	0.5821210	-3.6273400
H	6.3223180	1.4682970	-1.3007930
H	4.3924600	0.9051500	0.1127540
H	3.9897360	-2.1483790	0.4753150
H	5.3175390	-3.2437720	2.2214710
H	5.0773730	-2.5617210	4.6103250
H	3.4885880	-0.7586280	5.2227330
H	1.7896480	0.9013390	4.5728860
H	0.5000440	2.0516330	2.8349610
H	-0.6066050	0.7618280	-2.5679880
H	0.3235570	0.9976330	-4.8617250
H	3.5774200	3.3354570	-3.2831550
H	2.6396860	3.1119790	-1.0122930
H	-1.7534950	3.9143980	-0.2199540
H	-1.7061970	6.3138960	0.3255190
H	2.2522040	5.8642300	1.9652160
H	2.2057760	3.4642390	1.4186890
H	0.9783660	-3.7438780	4.8975340
H	-3.1371910	-5.6712980	-3.1953740
H	2.4237250	2.2862530	-5.2214670
H	-3.7109220	2.1748030	-1.6285210
H	-5.1515920	4.1040970	-1.1250300
H	-5.3806640	4.9287190	1.2185100
H	-4.1462970	3.7674040	3.0479180
H	-2.6949160	1.8446050	2.5445860
H	0.2957860	7.3108560	1.4293930
P	0.1747010	1.7507980	0.0180810
P	-0.3834430	-1.6125490	-0.2313690
C	-3.4507140	-0.8187890	-0.0160780
C	-3.7670150	-1.7369720	0.9982690
C	-4.3113840	-0.7717310	-1.1253880
C	-4.8758970	-2.5868370	0.9005290
H	-3.1384120	-1.8150730	1.8807860
C	-5.4182770	-1.6192240	-1.2365090
H	-4.1213730	-0.0635850	-1.9289530
C	-5.7062700	-2.5371920	-0.2219410

H	-5.0848240	-3.2923450	1.7029230
H	-6.0569320	-1.5610120	-2.1163020
H	-6.5648250	-3.1996880	-0.3036910

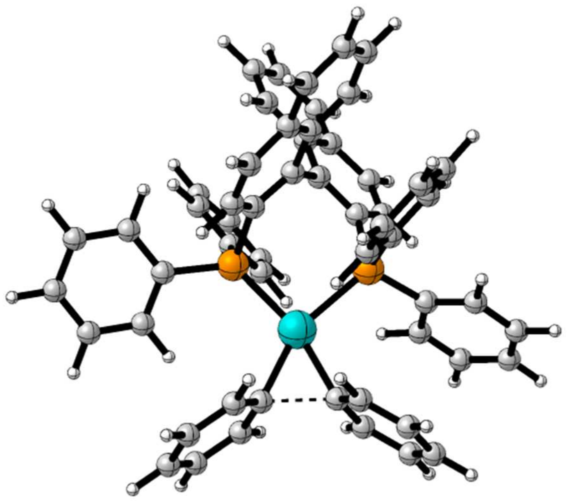

101

TS8 scf done: -2970.327215; Sum of electronic and thermal Free Energies: -2969.606631; imag. freq.:1 (-401.1223); Thermal correction to Gibbs Free Energy: 0.720585; scf(6-311+G**): -2970.8722614

C	-3.7063620	1.9065850	1.5223980
C	-3.4799890	1.3998900	0.2233990
C	-3.9988490	2.1443890	-0.8591800
C	-4.6835930	3.3427700	-0.6553780
C	-4.8863380	3.8351410	0.6400980
C	-4.3900350	3.1049160	1.7266860
Pd	-1.6917560	0.3314920	-0.0775230
C	1.3171450	0.9870570	1.2095670
C	2.1640450	-0.0673280	0.8748170
C	2.9157270	-0.7354040	1.9041560
C	2.7380950	-0.3550240	3.2722850
C	1.8101040	0.6738590	3.5802140
C	1.1271030	1.3230560	2.5837230
C	3.4756740	-1.0194510	4.2871450
C	4.3710450	-2.0187960	3.9717960
C	4.5511660	-2.3990290	2.6210210
C	3.8364060	-1.7811360	1.6168080
C	2.3150120	-0.5661760	-0.5302630
C	1.3480610	-1.3863600	-1.1182870
C	1.5801870	-1.9086690	-2.4206330
C	2.7137050	-1.5905660	-3.1294420
C	3.6967180	-0.7309910	-2.5794110
C	3.5061940	-0.2309900	-1.2519540
C	4.5085600	0.6166980	-0.7029040
C	5.6290390	0.9563520	-1.4294240

C	5.8074570	0.4689520	-2.7474820
C	4.8583830	-0.3582740	-3.3072730
C	-1.1332020	-2.9138600	-1.3029800
C	-1.6290700	-2.5472810	-2.5667360
C	-2.3212310	-3.4640940	-3.3559710
C	-2.5670610	-4.7544630	-2.8760570
C	-2.1134890	-5.1159840	-1.6059120
C	-1.3980100	-4.2038830	-0.8245450
C	0.0603990	3.5268560	0.6309450
C	1.1291550	4.2108020	1.2346370
C	0.9854210	5.5419990	1.6270780
C	-0.2277800	6.2083870	1.4185460
C	-1.2950570	5.5346950	0.8210360
C	-1.1536040	4.1993740	0.4331430
C	1.0760820	2.0279600	-1.5715290
C	0.5313070	1.4415860	-2.7219010
C	1.1540140	1.5978320	-3.9614400
C	2.3266700	2.3501310	-4.0630570
C	2.8683890	2.9506070	-2.9225180
C	2.2449460	2.7938330	-1.6850290
C	0.0976100	-2.4112210	1.2869370
C	-0.5023230	-1.9102260	2.4497380
C	-0.2058390	-2.4682290	3.6942840
C	0.6961210	-3.5309360	3.7858960
C	1.3000220	-4.0350390	2.6294780
C	1.0044600	-3.4767900	1.3861050
H	-1.1842310	-1.0692850	2.3712290
H	-0.6663220	-2.0624230	4.5912440
H	2.0162160	-4.8491630	2.6991670
H	1.4928520	-3.8558310	0.4927370
H	-1.4751840	-1.5347150	-2.9305430
H	-2.6891580	-3.1641530	-4.3339020
H	-2.3171940	-6.1100490	-1.2161710
H	-1.0589840	-4.4993820	0.1621230
H	0.8479730	-2.5697170	-2.8679540
H	2.8650890	-1.9961980	-4.1271170
H	4.9825950	-0.7395250	-4.3181760
H	6.6923610	0.7491370	-3.3126940
H	6.3792710	1.6091340	-0.9908370
H	4.3782950	1.0056100	0.3008360
H	3.9763870	-2.0968270	0.5897820
H	5.2511480	-3.1926580	2.3731150
H	4.9337180	-2.5185480	4.7558160
H	3.3199550	-0.7217340	5.3215190
H	1.6471750	0.9438840	4.6209600
H	0.4209260	2.1041800	2.8448540
H	-0.3785740	0.8538210	-2.6326960
H	0.7276380	1.1266190	-4.8432240
H	3.7845990	3.5301060	-2.9943630

H	2.6784160	3.2610040	-0.8073710
H	-1.9902600	3.6782060	-0.0179740
H	-2.2447000	6.0387200	0.6641140
H	1.8189820	6.0590180	2.0959890
H	2.0715850	3.7011250	1.4114850
H	0.9388950	-3.9580140	4.7552420
H	-3.1214100	-5.4662610	-3.4820650
H	2.8191840	2.4664400	-5.0248110
H	-3.8623360	1.7834010	-1.8750470
H	-5.0597610	3.8965520	-1.5131850
H	-5.4243080	4.7658780	0.7994490
H	-4.5320380	3.4737970	2.7403740
H	-3.3383540	1.3575000	2.3851510
H	-0.3391270	7.2448690	1.7265570
P	0.1714760	1.7967600	0.0136880
P	-0.3047170	-1.6118120	-0.3095650
C	-3.6142860	-0.5224020	0.0355560
C	-3.9432230	-1.2273180	1.2139680
C	-4.1856990	-0.9791740	-1.1703990
C	-4.7653590	-2.3526360	1.1814810
H	-3.5490580	-0.8939380	2.1697760
C	-5.0135310	-2.1008630	-1.2035770
H	-3.9765540	-0.4541650	-2.0983810
C	-5.3078590	-2.8011270	-0.0291420
H	-4.9821890	-2.8838610	2.1059900
H	-5.4208100	-2.4373250	-2.1542930
H	-5.9491270	-3.6781510	-0.0555810

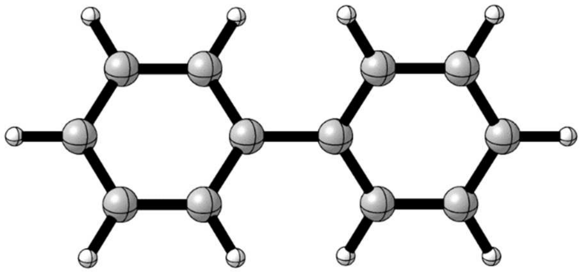

22
biphenyl scf done: -463.382222; Sum of electronic and thermal Free Energies: -463.234847; imag. freq.:0; Thermal correction to Gibbs Free Energy: 0.147375; scf(6-311+G**): -463.4815728

C	-2.8593070	-1.1404030	0.3954320
C	-1.4637230	-1.1396060	0.3960180
C	-0.7422360	0.0000040	0.0000000
C	-1.4637250	1.1396120	-0.3960220
C	-2.8593090	1.1404060	-0.3954370
C	-3.5640090	0.0000010	-0.0000020
H	-3.3966120	-2.0303220	0.7132530
H	-0.9268130	-2.0236150	0.7287480
H	-0.9268160	2.0236220	-0.7287530
H	-3.3966160	2.0303230	-0.7132610

H	-4.6508030	-0.0000000	-0.0000040
C	0.7422360	0.0000040	-0.0000000
C	1.4637250	1.1396120	0.3960210
C	1.4637230	-1.1396060	-0.3960190
C	2.8593090	1.1404060	0.3954370
H	0.9268170	2.0236220	0.7287530
C	2.8593070	-1.1404030	-0.3954320
H	0.9268130	-2.0236150	-0.7287480
C	3.5640090	0.0000010	0.0000020
H	3.3966160	2.0303230	0.7132600
H	3.3966120	-2.0303220	-0.7132530
H	4.6508030	-0.0000010	0.0000030

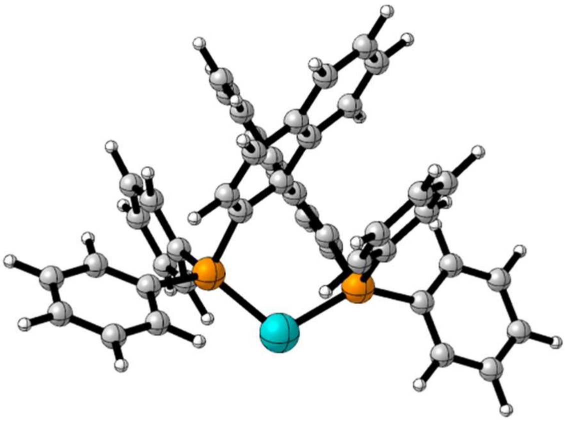

79

"Pd(BINAP)" scf done: -2506.937139; Sum of electronic and thermal Free Energies: -2506.385036; imag. freq.:0; Thermal correction to Gibbs Free Energy: 0.552103; scf(6-311+G**): -2507.409987 geometry taken from gas phase calculations

Pd	-0.1067710	-0.7966260	-2.3218780
C	1.1999050	1.1752750	-0.3403220
C	0.3174210	1.1314500	0.7390770
C	-0.1583160	2.3581740	1.3220020
C	0.1781180	3.6106630	0.7183540
C	0.9908730	3.6085460	-0.4442990
C	1.4964080	2.4353690	-0.9420650
C	-0.3015180	4.8178630	1.2898040
C	-1.0648910	4.8048220	2.4370400
C	-1.3884390	3.5707810	3.0479260
C	-0.9587680	2.3818960	2.4985210
C	-0.1900500	-0.1493510	1.3334010
C	-1.2390560	-0.8707710	0.7511420
C	-1.7154400	-2.0425810	1.4024410
C	-1.1700350	-2.4833650	2.5845990
C	-0.1136500	-1.7738850	3.2055460
C	0.3750050	-0.5855840	2.5764170
C	1.4243050	0.1293840	3.2195300
C	1.9544660	-0.3041920	4.4142180
C	1.4729700	-1.4860130	5.0283430
C	0.4616280	-2.2048360	4.4306570

C	-3.4368960	-1.4841780	-1.0487800
C	-3.3042680	-2.7705780	-1.5982460
C	-4.4084570	-3.6150560	-1.7189650
C	-5.6727150	-3.1733610	-1.3160480
C	-5.8202920	-1.8861930	-0.7931900
C	-4.7100190	-1.0480710	-0.6559690
C	3.5384320	0.2135960	-1.7345250
C	4.3512330	1.0817070	-0.9871630
C	5.6436510	1.3891450	-1.4149470
C	6.1451730	0.8300250	-2.5950720
C	5.3435820	-0.0307540	-3.3486490
C	4.0464530	-0.3293270	-2.9230100
C	2.2264120	-1.5891370	0.0447580
C	1.4731810	-2.7720760	0.0407770
C	1.7095350	-3.7688580	0.9870770
C	2.7102750	-3.5981690	1.9464760
C	3.4763170	-2.4300740	1.9479590
C	3.2386720	-1.4336080	1.0011360
C	-2.4780860	1.2558410	-0.8046830
C	-2.1832950	2.1354460	-1.8556350
C	-2.5846150	3.4711590	-1.7950870
C	-3.2833240	3.9406090	-0.6805950
C	-3.5751520	3.0719170	0.3745290
C	-3.1696700	1.7393360	0.3154170
H	-1.6158410	1.7675680	-2.7066010
H	-2.3382460	4.1465010	-2.6101170
H	-4.0895040	3.4400680	1.2574300
H	-3.3574680	1.0815220	1.1590400
H	-2.3247390	-3.0992820	-1.9390790
H	-4.2852290	-4.6091730	-2.1407840
H	-6.8019950	-1.5302290	-0.4910410
H	-4.8446800	-0.0501930	-0.2542980
H	-2.5278150	-2.6035800	0.9553750
H	-1.5519970	-3.3844940	3.0590970
H	0.0840190	-3.1156880	4.8896470
H	1.9036570	-1.8225440	5.9675820
H	2.7548060	0.2600690	4.8856600
H	1.8089790	1.0287190	2.7519240
H	-1.2295630	1.4461700	2.9725400
H	-1.9892960	3.5582130	3.9535760
H	-1.4185480	5.7358570	2.8717790
H	-0.0454630	5.7579370	0.8064500
H	1.2223470	4.5540680	-0.9292980
H	2.1302090	2.4615550	-1.8222720
H	0.6857160	-2.8873520	-0.6996500
H	1.1049560	-4.6718310	0.9808790
H	4.2492620	-2.2863530	2.6978710
H	3.8393140	-0.5309190	1.0186470
H	3.4142750	-0.9864920	-3.5157080

H	5.7228810	-0.4627820	-4.2710240
H	6.2594400	2.0657090	-0.8275650
H	3.9689450	1.5329720	-0.0763020
H	-3.5841900	4.9832120	-0.6240500
H	-6.5376070	-3.8230270	-1.4205960
H	2.8897960	-4.3675590	2.6925130
H	7.1515630	1.0706110	-2.9273030
P	1.8386120	-0.3206430	-1.2373060
P	-1.8932040	-0.4761810	-0.9505140

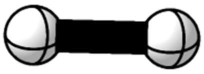

2
hydrogen scf done-1.175073; Sum of electronic and thermal Free Energies: -1.176429; imag. freq.:0; Thermal correction to Gibbs Free Energy: -0.001356; scf(6-311+G**): -1.1792595

H	0.0000000	0.0000000	0.3714210
H	0.0000000	0.0000000	-0.3714210

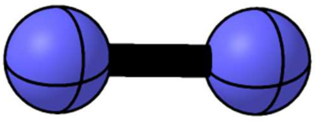

2
nitrogen scf done: -109.524945; Sum of electronic and thermal Free Energies: -109.537797; imag. freq.:0; Thermal correction to Gibbs Free Energy: -0.012851; scf(6-311+G**): -109.5546632

N	0.0000000	0.0000000	0.5524040
N	0.0000000	0.0000000	-0.5524040

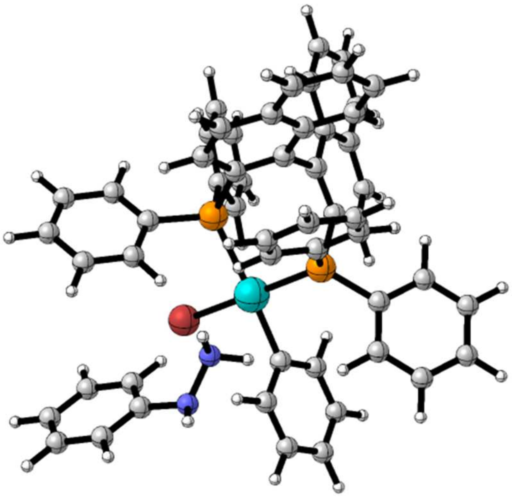

107
Int2a scf done: -5653.073386; Sum of electronic and thermal Free Energies: -5652.313626; imag. freq.:0; Thermal correction to Gibbs Free Energy: 0.759759; scf(6-311+G**): -5656.44275055 geometry taken from gas phase calculations

C	1.5867340	2.5383960	-2.8084460
C	2.0578360	1.8451940	-1.6875290
C	3.2728320	2.2387430	-1.1129640
C	3.9842050	3.3277400	-1.6311350
C	3.4971680	4.0286690	-2.7386520
C	2.2998650	3.6213600	-3.3334260
Pd	0.9886210	0.2996720	-0.9147420
C	-2.1044300	1.0453590	-0.7536730
C	-2.8614340	0.0253340	-0.1904550
C	-3.9833180	-0.5114370	-0.9119740
C	-4.2533910	-0.0519470	-2.2394130
C	-3.4023170	0.9296370	-2.8119100
C	-2.3680520	1.4677790	-2.0911410
C	-5.3513020	-0.5938100	-2.9566210
C	-6.1696130	-1.5435990	-2.3847080
C	-5.9095060	-1.9970820	-1.0703870
C	-4.8389460	-1.5020330	-0.3573070
C	-2.5016230	-0.6018800	1.1196230
C	-1.4322120	-1.4948290	1.2029920
C	-1.1701530	-2.1539520	2.4347410
C	-1.9225170	-1.8936340	3.5555340
C	-2.9766170	-0.9478420	3.5203230
C	-3.2845450	-0.3067430	2.2783050
C	-4.3446850	0.6408960	2.2492960
C	-5.0533390	0.9461780	3.3903390
C	-4.7410330	0.3161360	4.6203390
C	-3.7256920	-0.6129970	4.6795160
C	0.8952750	-2.9879880	0.3747070
C	2.0044500	-2.5916030	1.1373660
C	2.9036560	-3.5416840	1.6187690
C	2.7085580	-4.8962100	1.3322050
C	1.6155750	-5.2936240	0.5575930
C	0.7094900	-4.3430960	0.0794180
C	-0.6376390	3.5141360	-0.3460720
C	-1.8488660	4.1795440	-0.5903930
C	-1.8737250	5.5672430	-0.7457630
C	-0.6909260	6.3050400	-0.6504290
C	0.5156040	5.6490190	-0.3934170
C	0.5433920	4.2629720	-0.2408920
C	-0.7212330	1.7220900	1.8270070
C	0.1265620	0.9113010	2.5899260
C	0.0480150	0.9239580	3.9830790
C	-0.8747080	1.7527630	4.6251000
C	-1.7160100	2.5731260	3.8683070
C	-1.6368140	2.5631450	2.4767120
C	-1.2591310	-2.3769900	-1.5385010
C	-1.0733470	-1.9295500	-2.8538120
C	-1.8865520	-2.4159400	-3.8784030
C	-2.8859320	-3.3510700	-3.6004670

C	-3.0705230	-3.8049840	-2.2915320
C	-2.2642990	-3.3164630	-1.2645840
H	-0.2907790	-1.2109940	-3.0703590
H	-1.7394480	-2.0580040	-4.8937520
H	-3.8568380	-4.5198200	-2.0664240
H	-2.4362790	-3.6425260	-0.2426920
H	2.1896150	-1.5379360	1.3240780
H	3.7732130	-3.2175510	2.1822020
H	1.4717830	-6.3430590	0.3141530
H	-0.1245360	-4.6577030	-0.5387820
H	-0.3528470	-2.8630810	2.4927160
H	-1.7012020	-2.4020880	4.4910320
H	-3.4779150	-1.1022170	5.6185350
H	-5.3054600	0.5672430	5.5142110
H	-5.8558980	1.6776390	3.3490520
H	-4.5843260	1.1322600	1.3123860
H	-4.6431200	-1.8709770	0.6424620
H	-6.5529720	-2.7495790	-0.6226200
H	-7.0104070	-1.9485090	-2.9411570
H	-5.5353080	-0.2420610	-3.9688720
H	-3.5819710	1.2527860	-3.8342540
H	-1.7296800	2.2181850	-2.5457220
H	0.8379590	0.2658890	2.0864050
H	0.6946410	0.2715110	4.5643300
H	-2.4418310	3.2132870	4.3612590
H	-2.2902740	3.2071310	1.8977730
H	1.4893860	3.7645720	-0.0666590
H	1.4432860	6.2100470	-0.3278260
H	-2.8180190	6.0688350	-0.9405850
H	-2.7756240	3.6200140	-0.6642360
H	-3.5246560	-3.7204510	-4.3982420
H	3.4178330	-5.6360530	1.6936370
H	-0.9435640	1.7560080	5.7093760
H	3.6864080	1.7028670	-0.2660560
H	4.9258600	3.6174310	-1.1695770
H	4.0507990	4.8731120	-3.1416430
H	1.9152190	4.1491390	-4.2036910
H	0.6543180	2.2401870	-3.2823520
H	-0.7097290	7.3839550	-0.7788320
P	-0.5614200	1.7123780	-0.0020110
P	-0.2327900	-1.6697120	-0.2012250
Br	2.4786380	-1.2213630	-2.2314040
N	3.2640660	0.9453250	2.1719090
H	3.0096030	0.7197250	3.1350210
N	4.6676800	1.1267880	2.0437650
H	5.0955470	1.5711320	2.8522660
C	5.3893800	-0.0099350	1.6133280
C	6.6381110	-0.3033300	2.1824390
C	4.9035740	-0.8083970	0.5691190

C	7.3919310	-1.3788250	1.7061000
H	7.0195090	0.3111810	2.9966350
C	5.6576440	-1.8842820	0.1072480
H	3.9524950	-0.5839280	0.1049590
C	6.9040090	-2.1805980	0.6701190
H	8.3581390	-1.5938430	2.1558790
H	5.2573940	-2.4830650	-0.7060630
H	7.4891180	-3.0191650	0.3027950
H	2.7959730	1.8100870	1.9120840

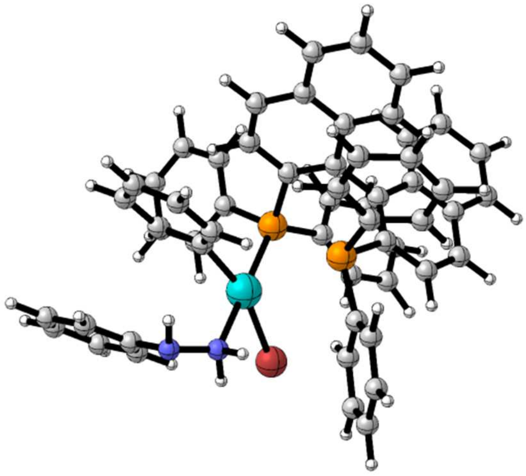

107
Int2b scf done: -5653.078497; Sum of electronic and thermal Free Energies: -5652.315846; imag. freq.:0; Thermal correction to Gibbs Free Energy: 0.762650; scf(6-311+G**): -5656.443956; geometry taken from gas phase calculations

C	-2.9347460	-0.4288240	2.1191940
C	-1.9683750	-0.9819070	1.2647700
C	-1.3194040	-2.1550920	1.6725100
C	-1.6085150	-2.7499930	2.9064350
C	-2.5544770	-2.1754830	3.7604470
C	-3.2192720	-1.0143100	3.3575670
Pd	-1.7682350	-0.2043390	-0.5924660
Br	-2.2768110	0.7462700	-2.9430330
P	1.3704800	-1.7188600	-0.6031380
C	2.3592390	-0.2357900	-1.0983220
C	2.5843410	0.7341520	-0.1236920
C	3.4567570	1.8363220	-0.3931220
C	4.0443950	1.9631040	-1.6916180
C	3.7469610	0.9862320	-2.6775830
C	2.9362980	-0.0854250	-2.3877780
C	4.9092530	3.0569150	-1.9574170
C	5.1998100	3.9824390	-0.9786150
C	4.6270020	3.8535900	0.3094940
C	3.7708620	2.8116630	0.5914880
C	2.0111820	0.5678150	1.2523620
C	0.6975570	0.9052900	1.5778780

C	0.2067810	0.6277670	2.8874570
C	1.0083600	0.0657060	3.8472500
C	2.3575920	-0.2651750	3.5643880
C	2.8682210	-0.0079870	2.2531550
C	4.2168020	-0.3665220	1.9742140
C	5.0123290	-0.9432530	2.9382930
C	4.5036950	-1.1934050	4.2357040
C	3.2021860	-0.8604780	4.5392000
P	-0.5312400	1.4923350	0.3339150
C	-1.6711530	2.6142910	1.2359940
C	-2.9471220	2.7896650	0.6770110
C	-3.8602490	3.6648860	1.2650940
C	-3.5115150	4.3720700	2.4191390
C	-2.2406240	4.2069560	2.9763680
C	-1.3221680	3.3366080	2.3852480
C	1.0526170	-2.5873940	-2.1932340
C	1.4377840	-3.9162140	-2.4321170
C	1.0221570	-4.5787820	-3.5920040
C	0.2175000	-3.9268470	-4.5298650
C	-0.1764640	-2.6041550	-4.2980270
C	0.2336320	-1.9445420	-3.1397150
C	2.7158110	-2.7341480	0.1570900
C	2.4784610	-3.2966600	1.4185340
C	3.4700730	-4.0390290	2.0651580
C	4.7113130	-4.2252040	1.4547570
C	4.9610360	-3.6625460	0.1979390
C	3.9710110	-2.9183480	-0.4434990
N	-2.8058840	-1.9460440	-1.3472850
C	0.3461070	2.6756740	-0.7636320
C	0.5804250	2.3767840	-2.1095800
C	1.2315780	3.2957970	-2.9308550
C	1.6649310	4.5178760	-2.4147980
C	1.4358180	4.8218520	-1.0707340
C	0.7752510	3.9089380	-0.2503610
H	0.2493810	1.4340050	-2.5189390
H	1.4055510	3.0463110	-3.9736060
H	1.7787080	5.7659530	-0.6568660
H	0.5993470	4.1616420	0.7895410
H	-3.2208030	2.2342710	-0.2161230
H	-4.8461190	3.7862980	0.8249180
H	-1.9615980	4.7549310	3.8725000
H	-0.3400750	3.2143060	2.8307020
H	-0.8277530	0.8363510	3.1243370
H	0.6037310	-0.1485560	4.8331940
H	2.7976860	-1.0510510	5.5305680
H	5.1405170	-1.6531810	4.9867190
H	6.0335910	-1.2223940	2.6961670
H	4.6139460	-0.1988070	0.9807040
H	3.3265320	2.7231920	1.5770990

H	4.8582250	4.5866500	1.0776390
H	5.8676270	4.8125740	-1.1924700
H	5.3410180	3.1495880	-2.9512380
H	4.1832620	1.0903400	-3.6684840
H	2.7322280	-0.8305680	-3.1497410
H	1.5243360	-3.1277760	1.9093590
H	3.2772000	-4.4544550	3.0504040
H	5.9291780	-3.7987730	-0.2775780
H	4.1741050	-2.4717420	-1.4128760
H	-0.1052840	-0.9275810	-2.9722400
H	-0.8097350	-2.0796880	-5.0086880
H	1.3315010	-5.6072720	-3.7605070
H	2.0627480	-4.4373210	-1.7139700
H	2.1817450	5.2290590	-3.0532380
H	-4.2259430	5.0469830	2.8827330
H	5.4862650	-4.7969850	1.9585460
H	-0.5609470	-2.5941450	1.0337530
H	-1.0857850	-3.6579250	3.2016270
H	-2.7760950	-2.6313820	4.7221410
H	-3.9739970	-0.5650280	3.9989320
H	-3.4814220	0.4613520	1.8273930
H	-3.2189370	-1.6541380	-2.2377330
H	-0.1041300	-4.4448530	-5.4293900
H	-2.1078470	-2.6470290	-1.6019540
N	-3.8181420	-2.6392390	-0.5864420
H	-3.3368350	-3.0536420	0.2098740
C	-4.8571340	-1.7948770	-0.1192420
C	-5.1504670	-0.5586630	-0.7081450
C	-5.6233170	-2.2465010	0.9665130
C	-6.1914700	0.2205030	-0.1921100
H	-4.5712280	-0.1747650	-1.5402970
C	-6.6538530	-1.4610980	1.4709160
H	-5.3853090	-3.2018370	1.4278440
C	-6.9441040	-0.2161530	0.8974150
H	-6.4023470	1.1821940	-0.6525980
H	-7.2272990	-1.8170900	2.3227080
H	-7.7463610	0.3984280	1.2956450

References

1. Gaussian 16, Revision C.01, Frisch, M. J.; Trucks, G. W.; Schlegel, H. B.; Scuseria, G. E.; Robb, M. A.; Cheeseman, J. R.; Scalmani, G.; Barone, V.; Petersson, G. A.; Nakatsuji, H.; Li, X.; Caricato, M.; Marenich, A. V.; Bloino, J.; Janesko, B. G.; Gomperts, R.; Mennucci, B.; Hratchian, H. P.; Ortiz, J. V.; Izmaylov, A. F.; Sonnenberg, J. L.; Williams-Young, D.; Ding, F.; Lipparini, F.; Egidi, F.; Goings, J.; Peng, B.; Petrone, A.; Henderson, T.; Ranasinghe, D.; Zakrzewski, V. G.; Gao, J.; Rega, N.; Zheng, G.; Liang, W.; Hada, M.; Ehara, M.; Toyota, K.; Fukuda, R.; Hasegawa, J.; Ishida, M.; Nakajima, T.; Honda, Y.; Kitao, O.; Nakai, H.; Vreven, T.; Throssell, K.; Montgomery, J. A., Jr.; Peralta, J. E.; Ogliaro, F.; Bearpark, M. J.; Heyd, J. J.; Brothers, E. N.; Kudin, K. N.; Staroverov, V. N.; Keith, T. A.; Kobayashi, R.; Normand, J.; Raghavachari, K.; Rendell, A. P.; Burant, J. C.; Iyengar, S. S.; Tomasi, J.; Cossi, M.; Millam, J. M.; Klene, M.; Adamo, C.; Cammi, R.; Ochterski, J. W.; Martin, R. L.; Morokuma, K.; Farkas, O.; Foresman, J. B.; Fox, D. J. Gaussian, Inc., Wallingford CT, 2016.
2. S. Grimme, S. Ehrlich and L. Goerigk, Effect of the damping function in dispersion corrected density functional theory, J. Comput. Chem., 2011, 32, 1456.
3. D. Andrae, U. Haussermann, M. Dolg, H. Stoll and H. Preuss, H. Energy-adjusted ab initio pseudopotentials for the second and third row transition elements, Theor. Chim. Acta, 1990, 77, 123.
4. A. V. Marenich, C. J. Cramer and G. D. Truhlar, Universal solvation model based on solute electron density and a continuum model of the solvent defined by the bulk dielectric constant and atomic surface tensions, J. Phys. Chem., B 2009, 113, 6378.
5. CYLview, 1.0b; C. Y. Legault, Université de Sherbrooke, 2009 (http://www.cylview.org)
6. Izquierdo, J., Jaina, A. D., Abdulkadirb, S. A. & Schiltz, G. E. Palladium-Catalyzed Coupling Reactions on Functionalized 2-Trifluoromethyl-4-chromenone Scaffolds: Synthesis of Highly Functionalized Trifluoromethyl Heterocycles. *Synthess*, **51**, 1342-1352 (2019).
7. Hennings, D.D., Iwasa, S. & Rawal, V. H. Anion Accelerated Palladium Catalyzed Intramolecular Coupling of Phenols with Aryl Halides. *J. Org. Chem.* **62**, 2-3 (1997).
8. Bajracharya, G. B. & Daugulis, O. Direct Transition MetalFree Intramolecular Arylation of Phenols. *Org. Lett.* **10**, 4625-4628 (2008).
9. Hosangadi, B. D. & Dave, R. H. An Efficient General Method for Esterification of Aromatic Carboxylic Acids. *Tetrahedron Lett.* **37**, 6375-6378 (1996).
10. Palencia, H., Garcia-Jimenez, F. & Takacs, J. M. Suzuki-Miyaura Coupling with High Turnover Number Using an N-Acyl-N-Heterocyclic Carbene Palladacycle Precursor. *Tetrahedron Lett.* **45**, 3849-3853 (2004).
11. Killander, D. & Sterner, O. Reagent-Controlled Cyclization-Deprotection Reaction to Yield Either Fluorenes or Benzochromenes. *Eur. J. Org. Chem.* **29**, 6507-6512 (2014).
12. Ikawa, T., Yamamoto, Y., Heguri, A., Fukumoto, Y., Murakami, T., Takagi, A., Masuda, Y., Yahata, K., Aoyama, H., Shigeta, Y., Tokiwa, H. & Akai, S. Could London Dispersion Force Control Regioselective (2 + 2) Cyclodime;rizations of Benzynes? YES: Application to the Synthesis of Helical Biphenylenes *J. Am. Chem. Soc.* **143**, 10853-10859 (2021).

13. Sheldrick, G. M. SHELXT – Integrated space-group and crystal-structure determination *Acta Cryst.* **A71**, 3-8 (2015).
